# Regioselective Arylation of Amidoaryne Precursors via Ag‐Mediated Intramolecular Oxy‐Argentation

**DOI:** 10.1002/advs.202308829

**Published:** 2024-02-25

**Authors:** Yong‐Ju Kwon, Ye‐Jin Kong, Min‐Jung Lee, Eun‐Hye Lim, Jaesung Kwak, Won‐Suk Kim

**Affiliations:** ^1^ Department of Chemistry and Nanoscience Ewha Womans University Seoul 03760 Republic of Korea; ^2^ Infectious Diseases Therapeutic Research Center Korea Research Institute of Chemical Technology (KRICT) Daejeon 34114 Republic of Korea; ^3^ Division of Medicinal Chemistry and Pharmacology KRICT School University of Science and Technology Daejeon 34114 Republic of Korea

**Keywords:** amidobenzyne precursors, aryne, bimetallic cross‐coupling, oxy‐argentation, regioselective arylation

## Abstract

An unprecedented silver‐mediated intramolecular oxy‐argentation of 3‐amidoaryne precursors that quickly generates a heteroarylsilver species is developed. AgF acts as both a stoichiometric fluoride source and a reagent for the formation of a benzoxazolylsilver intermediate via aryne generation. Pd‐catalyzed coupling reactions of (hetero)aryl iodides with a silver species, generated in situ, allow for the synthesis of various C7‐arylated benzoxazoles. As a result, an aryl group is selectively introduced into the meta‐position of 3‐amidobenzyne precursors. Mechanistic studies have indicated the presence of a benzoxazolylsilver intermediate and revealed that the reaction proceeds via an intramolecular oxy‐argentation process, which is initiated by a direct fluoride attack on the silyl group.

## Graphical abstract

Ag‐mediated/Pd‐catalyzed cross‐coupling via intramolecular oxy‐argentation of aryne to synthesize heterobiaryl compounds has first been realized. The newly developed method for subjecting a heteroarylsilver intermediate formed via aryne generation to coupling reactions opens up numerous opportunities to expand the scope of aryne chemistry.

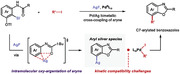

## 1 Introduction

**Figure 1:**
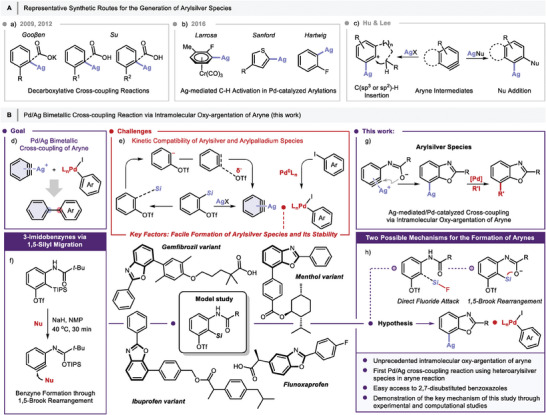
A) Representative synthetic routes for the generation of arylsilver species. B) Pd/Ag Bimetallic cross‐coupling reaction via intramolecular oxy‐argentation of aryne (this work).

Biaryl scaffolds are important building blocks found in a variety of natural products, pharmaceuticals, organic electronic devices, and fine chemicals.^[^ ^1^ ^]^ Therefore, over the past decades, numerous synthetic methods have been developed to synthesize biaryl compounds via transition‐metal catalyzed cross‐coupling reactions.^[^ ^2^ ^]^ Among them, the recent development of Pd/Ag bimetallic system for the synthesis of biaryl compounds has greatly expanded the scope of traditional cross‐coupling reactions, by allowing the use of unreactive functional groups, such as carboxylic acids or C─H bonds as organonucleophiles.^[^ ^3^ ^]^ The key features of Pd/Ag bimetallic systems for biaryl synthesis are a facile generation of organosilver intermediates followed by their transmetalation to palladium catalysts, and the transmetalation step should be faster than the decomposition of the resulting organosilver species.^[^ ^3a^ ^]^ After the seminal work by Gooβen et al.,^[^ ^4^ ^]^ who presented the first example of Pd/Ag‐catalyzed decarboxylative cross‐coupling reactions (**Figure** **1**A‐a), Su et al.^[^ ^5^ ^]^ reported the synthesis of unsymmetrical biaryl compounds from two different aryl carboxylic acids via Ag‐mediated Pd‐catalyzed cross‐coupling method (Figure 1A‐b). In 2016, Larrosa proposed the first example of Ag‐mediated C─H activation in Pd‐catalyzed arylations.^[^ ^6^ ^]^ From the aforementioned strategy, (hetero)aryl‐silver(I) salts have been shown to offer elegant bimetallic routes in the construction of biaryl compounds. Particularly, in the same year, Sanford^[^ ^7^ ^]^ and Hartwig^[^ ^8^ ^]^ independently demonstrated exquisite experimental evidence of the stoichiometric C─H activation by Ag salts. In this way, Pd/Ag bimetallic process has been investigated according to the synthetic method of Ag species.

Meanwhile, an arylsilver species can also be generated in situ from a highly reactive aryne intermediate.^[^ ^9^ ^]^ It has been shown that a benzyne coordinates to the silver cation, thereby affording the benzyne‐silver π‐complex. This complex displays a distinctive reactivity at the benzyne carbon due to the increased electrophilic character in the silver‐coordinated complex.^[^ ^10^ ^]^ Alternatively, it has been reported that the addition of suitable nucleophiles with silver counter cation to the aryne intermediate generates the arylsilver intermediate.^[^ ^11^ ^]^ For example, Hu^[^ ^11a,b^ ^]^ and Lee et al.,^[^ ^11^, ^12^ ^]^ have elaborated on the Ag^+^‐activated aryne reactions, utilizing the benzyne intermediates generated in situ from Kobayashi and triyne or tetrayne precursors, respectively. Representatively, Ag^+^‐catalyzed C(sp^3^ or sp^2^)−H insertion by aryne intermediates effectively furnished the fused and bridged 5‐membered carbocycles. Furthermore, addition of nucleophiles to Ag^+^‐activated aryne species resulted in the nucleophile‐added Ag^+^‐aryne intermediates, which provided bis‐functionalized products after trapping the halogenation reagents (Figure 1A‐c).

While these examples demonstrate the versatility of silver‐mediated benzyne functionalization, the scope of reactions is solely limited to the reactivity of the arylsilver species. Under these circumstances, we envisioned that the arylsilver intermediates generated by the benzyne precursors could be involved in the palladium‐catalyzed cross‐coupling reactions for the synthesis of biaryl compounds (Figure 1B‐d). However, Pd/Ag bimetallic cross‐coupling reactions, which utilize arylsilver species generated from benzyne intermediates remain challenging for the catalytic cycle due to the requisite simultaneous formation of the organosilver and organopalladium intermediates. Thus, the incorporation of benzyne reaction into palladium‐catalyzed reaction to synthesize biaryl compounds using Pd/Ag bimetallic systems has not yet been explored. The approach to realize this goal was to develop a straightforward and distinct aryne reaction that can achieve a bimetallic cross‐coupling reaction. Several challenges were considered to address this aim and establish the hypothetical processes in the desired direction (Figure 1B‐e). Firstly, the kinetic compatibility between the arylsilver and the arylpalladium species was considered in the transmetalation step. Secondly, to circumvent the formation of byproducts, the reagents and catalyst system should not tackle each other. Thirdly, the aryne species should be immediately captured with a silver reagent instead of a palladium catalyst or a palladium complex to prevent any undesired transformations. The key factors covering all these issues are related to the facile formation of arylsilver species through the coordination of silver(I) with intermediates obtained from well‐designed aryne precursors and the stability of the arylsilver species before the transmetalation step.^[^ ^3^, ^13^ ^]^

Recently, we demonstrated novel 3‐amino‐2‐(trialkylsilyl)phenyl triflates as aminobenzyne precursors.^[^ ^14^ ^]^ These are base‐activated aminoaryne precursors induced via an 1,3‐aza‐Brook rearrangement, and the resulting aminoaryne intermediates were used for nucleophilic addition and cycloadditions to furnish various aniline derivatives. Interestingly, when the reaction was performed using an amidoaryne precursor and NaH in *N*‐methyl‐2‐pyrrolidone (NMP) at 40 °C, *O*‐silylimidate was obtained from the imidobenzyne intermediate formed through 1,5‐Brook rearrangement (Figure 1B‐f). Therefore, these observations led us to design a model study to efficiently generate arylsilver intermediates using amidoaryne precursors. Additionally, we hypothesized that the model precursor would be transformed via intramolecular cyclization to the biologically potent benzoxazole,^[^ ^15^ ^]^ a ubiquitous structural motif in a myriad of medicines and natural products (Figure 1B‐g). Herein, we report a new synthetic toolbox for Ag‐mediated/Pd‐catalyzed cross‐coupling via the intramolecular oxy‐argentation of aryne, to synthesize heterobiaryl compounds. Furthermore, to rationalize the observed results, we investigated two possible mechanistic scenarios for the formation of aryne intermediates: direct fluoride attack or 1,5‐Brook rearrangement (Figure 1B‐h). Indeed, our experimental and theoretical analyses revealed the key mechanism in this study; thus represents the first example of bimetallic cross‐coupling reaction via intramolecular oxy‐argentation of arynes for the synthesis of heterobiaryl compounds.

## 2 Results and Discussion

**Scheme 1:**
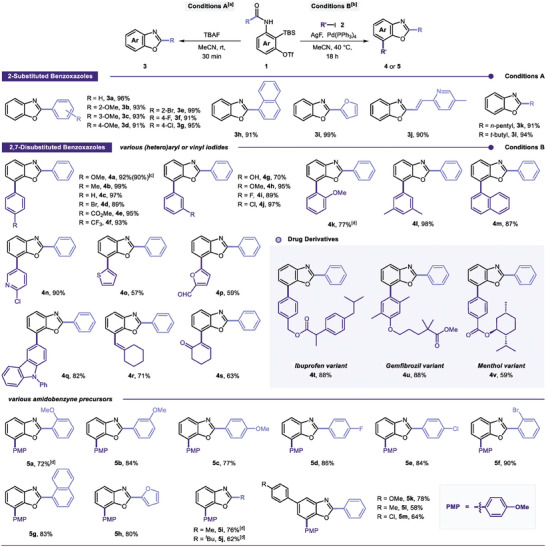
Substrate scope for the synthesis of 2‐mono, 2,7‐di, and 2,5,7‐trisubstituted benzoxazoles. a) Conditions A: **1** (0.2 mmol), TBAF (0.44 mmol), MeCN (6.7 mL), rt, 30 min. b) Conditions B: **1** (0.26 mmol), **2** (0.2 mmol), AgF (0.8 mmol), Pd(PPh_3_)_4_ (10 mol%), MeCN (2.0 mL), 40 °C, 18 h. c) 1.0 mmol scale of **2a** was used. d) 1.5 equiv. of **1** was used.

With these potential challenges in mind, we commenced our study by evaluating a series of fluoride bases for the proposed reaction of **1a**, a model precursor having an amido group (**Table** **1**). Pleasingly, using TBAF (2.2 equiv.) and MeCN at room temperature, the desired intramolecular cyclized product, benzoxazole **3a** was produced in 96% yield (entry 1). Notably, the replacement of TBAF with CsF or AgF, both led to a significant decrease in reaction time in the competent yields, thus, suggesting that the fluoride ion source affects the benzyne generation rate (entries 2–3). In addition, when the amidoaryne precursor **1b** containing TIPS group was used, **3a** was obtained in 54% yield (entry 4). From these initial results, we subsequently investigated the bimetallic cross‐coupling reaction conditions (entries 5–11). Pleasingly, after extensive screening of various reaction parameters (see the Supporting Information for details), we were able to obtain 2,7‐disubstituted benzoxazole **4a** in 92% yield (entry 5). However, by reducing the amount of Pd catalyst to 5 mol%, the yield of **4a** was also reduced to 82% (entry 6). In addition, the use of **1a** (1.0 equiv.) and **2a** (1.3 equiv.) resulted in the desired cross‐coupled benzoxazole **4a** in 81% yield (entry 7). Here, the reason for the decrease in yield was that **3a** was provided by protodeargentation of the arylsilver species formed from the limiting substance **1a**. When SPhos or RuPhos were used as the ligand, **4a** was afforded in 13% and 14% yields, respectively (entries 8–9). Conversely, the use of CsF instead of AgF did not provide the desired coupled product **4a** and only benzoxazole **3a** was observed (entry 10). This suggests that the generation of arylsilver(I) species is essential to obtain the cross‐coupled product **4a**. Lastly, when the amidoaryne precursor **1b** was used under the standard conditons B, the desired product **4a** was obtained in 42% yield (entry 11). In addition, the reaction did not occur in the absence of Pd catalyst contrary to the standard conditions (entry 12). Therefore, these results demonstrate that the final optimized conditions consist of **2a** (1.0 equiv.), **1a** (1.3 equiv.), AgF (4.0 equiv.), Pd(PPh_3_)_4_ (10 mol%), and MeCN (0.1 m) at 40 °C. Following confirmation of the optimized conditions, we next probed the scope for the synthesis of 2‐mono, 2,7‐di‐, and 2,5,7‐trisubstituted benzoxazoles (**Scheme** **1**). The observed excellent intramolecular cyclization toward the *ortho*‐position to the original amido group led us to first examine the reaction of various 3‐amidobenzyne precursors. This protocol turned out to be highly efficient under mild conditions, affording the desired 2‐substituted benzoxazoles in excellent yields. The position and electronic properties of the substituents were not critical to the efficiency of this cyclization, as demonstrated by the smooth formations of **3a**–**3 g**. The amidobenzyne precursor with the bulky naphthalene substituent also underwent smooth cyclization and generated the corresponding benzoxazole **3** **h** with high efficiency (91%). Intriguingly, the reaction of amidobenzyne precursors bearing heterocycles such as furan and pyridine was also facile to give the desired products **3i** and **3j** with high yields. Moreover, the substrates bearing alkyl chains were all readily cyclized through the intramolecular benzyne process (**3k** and **3l**).

**Table 1:** Optimization studies.

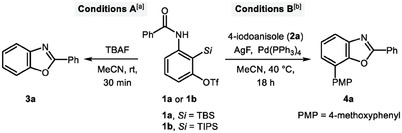		
Entry^a)^, ^b)^ ^)^	Variation from the standard conditions A	yield of 3a (%)^c^ ^)^
1	None	96
2	CsF, 6 h	93
3	AgF, 20 h	92
4	**1b** (1.0 equiv.), AgF, 20 h	54
Entry	Variation from the standard conditions B	yield of **4a** (%)^c^ ^)^
5	None	92
6	Pd(PPh_3_)_4_ (5 mol%)	82
7	**1a** (1.0 equiv.), and **2a** (1.3 equiv.)	81
8	Pd(OAc)_2_ (10 mol%), and SPhos (20 mol%)	13
9	Pd(OAc)_2_ (10 mol%), and Ruphos (20 mol%)	14
10	CsF instead of AgF	0
11	**1b** (1.3 equiv.), and **2a** (1.0 equiv.)	42
12	without Pd(PPh_3_)_4_	0

^a)^ Reaction conditions: **1a** (0.2 mmol), TBAF (0.44 mmol), MeCN (6.7 mL), rt, 30 min;

^b)^ Conditions B: **1a** (0.26 mmol), **2a** (0.2 mmol), AgF (0.8 mmol), Pd(PPh_3_)_4_ (10 mol%), MeCN (2.0 mL), 40 °C, 18 h;

^c)^ Isolated yield.

Next, to investigate the generality of the bimetallic cross‐coupling reaction, a range of substrates with additional substituents were further examined under the optimized conditions B. To our delight, the presently developed protocol was effective over a broad range of (hetero)aryl or alkenyl iodides and furnished 2,7‐disubstituted benzoxazoles **4a**–**4v** in good to excellent yields. Furthermore, when 1.0 mmol of **2a** was used, the reaction furnished **4a** in 90% yield, demonstrating the feasibility of the present protocol. Notably, when the sterically hindered 2‐methoxyphenyl iodide was used, 1.5 equiv. of **1a** was required to complete the reaction and **4k** was obtained in 77% yield. Halo‐substituted phenyl iodides also proved to be effective coupling partners, furnishing the coupled products **4d**, **4i**, and **4j** in 89%, 89%, and 97% yields, respectively. When the arylsilver species generated in situ from **1a** under the optimized conditions were reacted with various heteroaryl iodides (**4n**–**4q**) or alkenyl iodides (**4r** and **4s**), the coupled products were obtained in yields ranging from 57% to 90%. Additionally, the present method was suitable for the synthesis of benzoxazole‐based drug derivatives containing key moieties such as ibuprofen (**4t**), gemfibrozil (**4u**), and menthol (**4v**).

We further investigated the scope of the Pd/Ag bimetallic cross‐coupling reaction via intramolecular oxy‐argentation of aryne using **2a** and various 3‐amidobenzyne precursors. A wide range of functionalized aryne precursors coupled with **2a** provided the coupled products in yields ranging from 62% to 90% (**5a**–**5m**). For **5a**, **5i**, and **5j**, the reaction was not completed under the optimized conditions. However, when 1.5 equiv. of amidobenzyne precursors was used, **5a**, **5i**, and **5j** were obtained in yields of 72%, 76%, and 62%, respectively. Moreover, an analogous pathway was operative for the 5‐arylated 3‐amidobenzyne precursors to provide the desired 2,5,7‐trisubstituted benzoxazole products (**5k**–**5m**), in moderate yields.

**Scheme 2:**
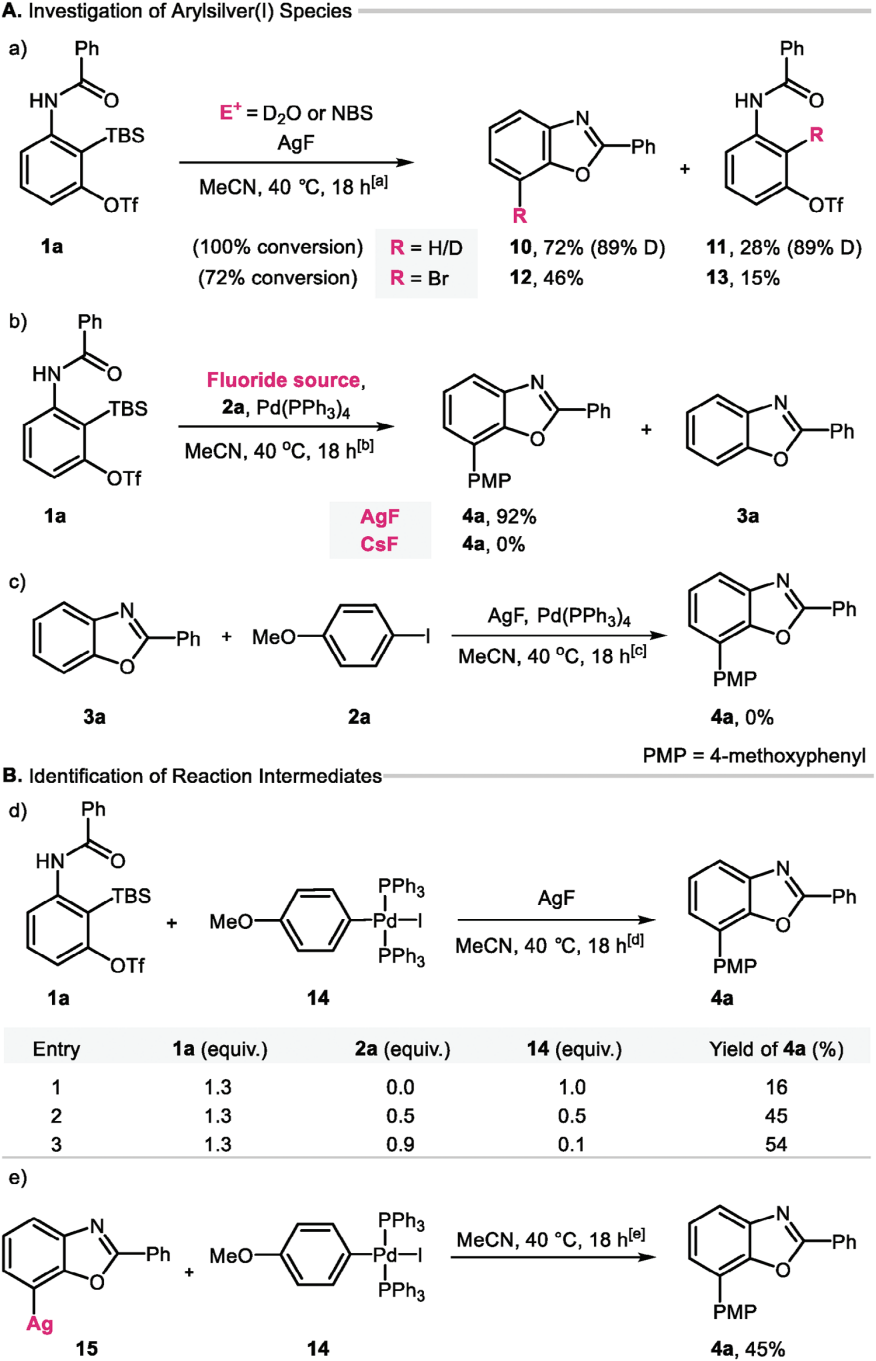
Mechanistic studies. a) **1a** (0.39 mmol), AgF (1.2 mmol), D_2_O (6.0 mmol) or NBS (0.9 mmol), MeCN (3.0 mL), 40 °C, 18 h. b) **1a** (0.39 mmol), **2a** (0.3 mmol), AgF (1.2 mmol) or CsF (1.2 mmol), Pd(PPh_3_)_4_ (10 mol%), MeCN (3.0 mL), 40°C, 18 h. c) **3a** (0.39 mmol), **2a** (0.3 mmol), AgF (1.2 mmol), Pd(PPh_3_)_4_ (10 mol%), MeCN (3.0 mL), 40°C, 18 h. d) **1a** (0.39 mmol), **2a** (0.0–0.9 equiv.), **14** (0.1–1.0 equiv.), AgF (1.2 mmol), MeCN (3.0 mL), 40°C, 18 h. e) **15** (0.13 mmol), **2a** (0.1 mmol), MeCN (1.0 mL), 40°C, 18 h.

Next, a series of control experiments were performed to gain insight into the reaction mechanisms and the role of AgF in Pd/Ag bimetallic cross‐coupling reaction via intramolecular oxy‐argentation of amidoaryne (**Scheme** **2**). First, a deuterium‐labeling experiment and a bromination reaction were performed to investigate the formation of the benzoxazolylsilver intermediate when 3‐aminobenzyne precursor **1a** was treated with AgF in MeCN at 40 °C for 18 h. When D_2_O or NBS was used, the corresponding products **10** and **12** were obtained in yields of 72% (89% D incorporation) and 46%, respectively. Interestingly, byproducts **11** and **13**, furnished by a direct attack of fluoride ions to silicon, were also observed, thereby indicating that the benzoxazolylsilver species is the reactive intermediate in this reaction (Scheme 2A‐a). In addition, as previously mentioned, when 4‐iodoanisole **2a** reacts with the amidobenzyne precursor **1a** under the standard conditions B, the desired product **4a** was afforded in 92% yield. Instead, when CsF was added as the fluoride source, under otherwise identical conditions, the 2,7‐disubstituted product **4a** was not detected but instead rather likely to afford the protonated substrate **3a** (Scheme 2A‐b). Accordingly, these results suggest that AgF acts as both a stoichiometric fluoride source and a reagent for the formation of the benzoxazolylsilver intermediate via aryne generation, giving the desired product **4a** in the presence of aryl iodide and palladium catalyst.

Furthermore, the palladium‐catalyzed C─H activation/C─C bond forming reaction using benzoxazole **3a** was not successful under the standard conditions B (Scheme 2A‐c).^[^ ^16^ ^]^ This result ruled out the involvement of a C─H activation pathway involving **3a**, indicating that the heteroarylsilver species is the reactive intermediate formed directly from the 3‐amidobenzyne precursor under the optimized conditions.

To elucidate this arylsilver pathway and its feasibility of incorporating it into the cross‐coupling reaction, we attempted a stoichiometric experiment using the palladium oxidative addition complex **14** (Scheme 2B‐d). Intriguingly, when 1.0 equiv. of **14** and 1.3 equiv. of **1a** were treated with AgF in MeCN at 40 °C for 18 h, only a negligible amount of **4a** was obtained, mainly due to the competitive side reaction between the palladium complex **14** and AgF (entry 1).^[^ ^17^ ^]^ Therefore, when the reaction was conducted using reduced amounts of **14**, it led to an increase in product yields, suggesting that efficient generation of arylsilver species from preferential reaction of AgF with the aminobenzyne precursor is essential for the reaction (entries 2 and 3). Consequently, we conducted further direct coupling reactions by taking benzoxazolylsilver species **15** ^[^ ^18^ ^]^ and the palladium oxidative addition complex **14** as the feasible intermediates in this reaction (Scheme 2). Indeed, the participation of each metallic complex afforded the 2,7‐disubstituted product **4a** in a yield of 45%.

**Figure 2:**
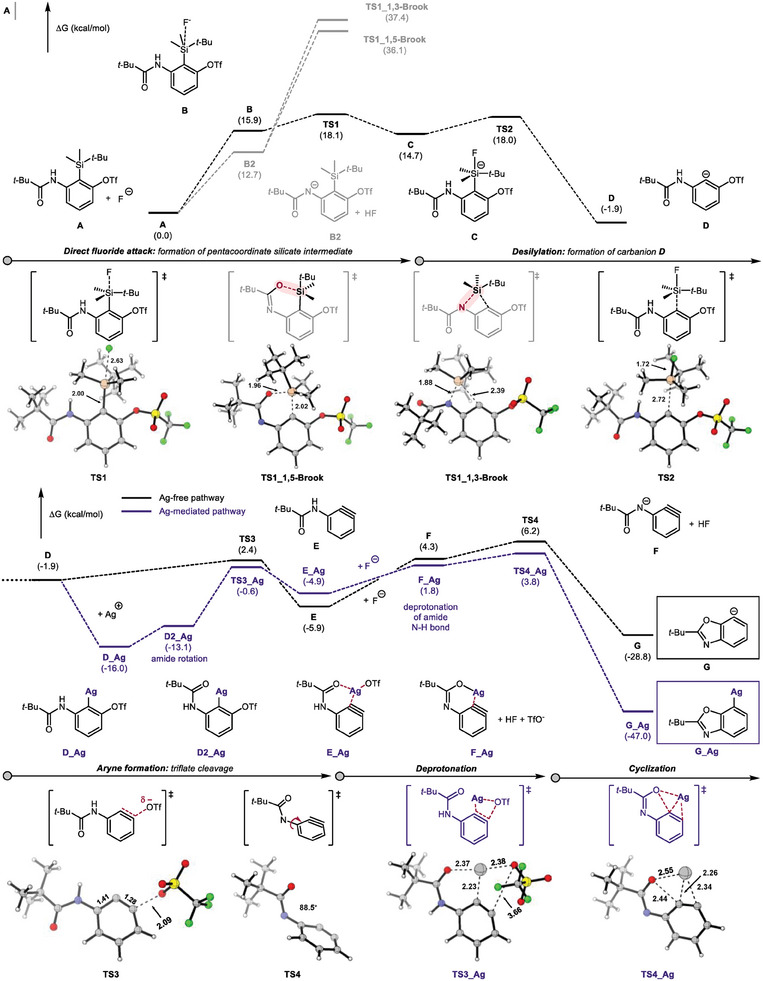
Calculated potential energy surface of arylsilver intermediate formation A. DFT studies. All calculations were performed at the SMD(MeCN)/M06/SDD(Ag)/6‐311++G**//SMD(MeCN)/B3LYP‐D3/LANL2DZ(Ag)/6‐31G** level of theory. Energies are presented in kcal/mol.

To further understand the formation of the arylsilver species via an aryne intermediate, we performed density functional theory (DFT) studies to calculate the Gibbs free energies of the intermediates and transition states along the possible pathways (**Figure** **2**). First, we analyzed the pathway where fluoride directly attack the TBS group, affording carbanion intermediate **D**.^[^ ^19^ ^]^ The formation of the pentacoordinate silicate intermediate **C** was computed as 14.7 kcal mol^−1^ with a barrier of 18.1 kcal/mol. The carbon‐silicone bond for **C** was cleaved with a barrier of 3.3 kcal/mol, which resulted in the formation of the carbanion species **D**.

Further calculations were performed to explore alternative pathways for the desilylation of **A**. When fluoride deprotonates the amide N─H bond of **A**, two possible pathways emerge: the 1,3‐aza‐Brook rearrangement, where the TBS group migrates to the nitrogen, and the 1,5‐Brook rearrangement, where the TBS group migrates to the amide oxyanion. However, our calculations indicated that the barriers for **TS_1,3‐Brook** and **TS_1,5‐Brook** are computed as 37.4 kcal/mol and 36.1 kcal molmol^−1^, respectively. These results clearly demonstrate that desilylation through either 1,3‐ or 1,5‐Brook rearrangement is not feasible under our reaction conditions.

Regarding the formation of aryne, it proceeds through a downhill process facilitated by the triflate cleavage transition state, **TS3**. Subsequent deprotonation by fluoride (**F**) followed by rotation of the amide (**TS4**), leads to the generation of the benzoxazole anion **G** with the formation of the C─O bond. Our calculation showed that desilylation of **A** by fluoride can afford the desired benzoxazolyl anion without the need for the silver cation at the ambient temperature. Indeed, when **1l** was reacted with stoichiometric TBAF, the desired product **3l** was obtained in 94% yield (Scheme 1).

Next, we investigated the role of the silver ion during the formation of benzoxazole and found that the coordination of silver to **D** is an exergonic process of 14.1 kcal/mol. The formation of aryne (**E_Ag**) from **D_Ag** involves amide rotation to produce **D2_Ag**, which has an intramolecular amide coordination with silver. A subsequent triflate cleavage step generates **E_Ag** via **TS3_A** where triflate migrates to silver. Deprotonation of the amide N─H bond in **E_Ag** by fluoride generates **F_Ag**, and the simultaneous formation of the C─O and Ag─C bonds by **TS4_Ag**, resulting in the arylsilver intermediate **G_Ag**. The formation of **G_Ag** from **D_Ag** is computed as being highly exergonic by 31 kcal mol^−1^ with a free energy barrier of 19.8 kcal mol^−1^ (**D_Ag** → **TS4_Ag**). These results strongly support the experimental evidence for the formation of the arylsilver intermediate.

**Figure 3:**
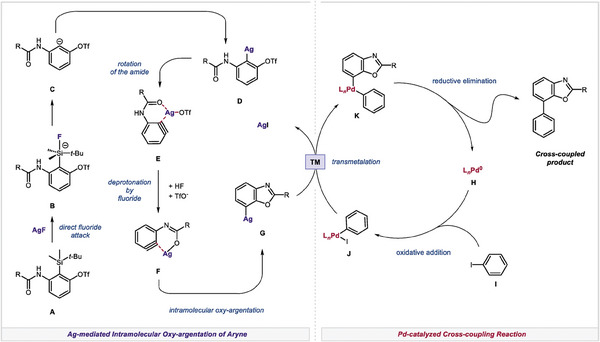
Plausible reaction mechaism.

Based on data generated by both experimental and computational analyses, we propose the reaction mechanism as depicted in **Figure** **3**. Under the reaction conditions, a fluoride anion directly attacks the TBS group of **A** and generates the carbanion **C**. The resulting carbanion is coordinated to a silver ion, followed by silver‐assisted aryne formation, and the production of the silver‐bound aryne intermediate **E**. The subsequent deprotonation and intramolecular oxy‐argentation produces the arylsilver intermediate **G**. This intermediate undergoes transmetalation with the arylpalladium species **J** to generate diarylpalladium intermediate **K**. The following reductive elimination affords the desired product and the Pd(0) species **H**. Notably, the formation of the arylsilver intermediate is computed as being a highly exergonic process, which provides a sufficient concentration of the arylsilver species for the transmetalation step in the Pd catalytic cycles. Therefore, the rate‐determining step is involved in the Pd catalytic cycle rather than the arylsilver formation. Indeed, we observed the high extent of the arylsilver intermediate compared to the initial palladium loading throughout the reaction, supporting our proposed mechanism (see Figure S1, Supporting Information for details).

## 3 Conclusion

In summary, we developed the first Pd/Ag bimetallic cross‐coupling reaction in aryne chemistry using heteroarylsilver species formed by intramolecular oxy‐argentation of 3‐amidoaryne precursors. The reaction displayed a broad scope with respect to both (hetero)aryl or alkenyl iodides and amidobenzyne precursors, affording 2‐mono, 2,7‐di‐, or 2,5,7‐trisubstituted benzoxazoles. Mechanistic investigation revealed that heteroarylsilver species is likely to be involved in this process as a reactive intermediate and suggested a dual role of AgF as a stoichiometric fluoride source and a reagent for the formation of the silver intermediate. Furthermore, the observed intramolecular oxy‐argentation of amidobenzyne precursor was fully rationalized by computational studies, suggesting that the direct fluoride attack to silyl group is more feasible than 1,3‐ or 1,5‐Brook rearrangement during desilylation in the formation of aryne intermediate. Further investigation of the application of Pd/Ag bimetallic cross‐coupling reaction in aryne chemistry is ongoing in our laboratory.

## 4 Experimental Section

### General Procedure for the Synthesis of 2‐Monosubstituted Benzoxazoles 3a–3l

***Conditions A***. To a solution of **1** (0.25 mmol, 1.0 equiv.) in anhydrous MeCN (8.3 mL) was added dropwise TBAF (0.55 mmol, 2.2 equiv.) and stirred at room temperature for 30 min. After completion of reaction, the reaction mixture was quenched with H_2_O and extracted with diethyl ether (3 × 10 mL). the organic phase was collected, dried over anhydrous MgSO_4_, filtered, and concentrated under reduced pressure. The crude product was purified by flash column chromatography on silica gel to obtain desired products **3**.

### General Procedure for the Synthesis of 2,7‐Di and 2,5,7‐Trisubstituted Benzoxazoles 4a–5m

***Conditions B***. A sealed tube was charged with **1** (0.39 mmol, 1.3 equiv.), (hetero)aryl or alkenyl iodide **2** (0.3 mmol, 1.0 equiv.), AgF (1.2 mmol, 4.0 equiv.) and Pd(pph_3_)_4_ (0.03 mmol, 10 mol%). The sealed tube and contents were placed under vacuum and back‐filled with argon under a Schlenk line three times. Degassed anhydrous MeCN (3.0 mL, 0.1 m) was added under argon, and the reaction mixture was stirred in an oil bath at 40 °C for 18 h. After completion of the reaction as monitored by TLC analysis, the reaction mixture was cooled to room temperature, celite‐filtered with diethyl ether (3×10 mL), and concentrated under reduced pressure. The crude product was purified by flash column chromatography on silica gel to obtain desired products **4** or **5**.

## Conflict of Interest

The authors declare no conflict of interest.

## Supporting information

Supporting Information

## Data Availability

The data that support the findings of this study are available in the supplementary material of this article.
