# Supplementary material for: Regioselective Arylation of Amidoaryne Precursors via Ag‐Mediated Intramolecular Oxy‐Argentation
Source: Adv Sci (Weinh). 2024 Feb 25;11(17):2308829. doi: 10.1002/advs.202308829 (PMC11077674; doi:10.1002/advs.202308829)
Supplement: Supplementary file 1 — Supporting Information [file ADVS-11-2308829-s001.pdf]

ADVANCED SCIENCE
Open Access

Supporting Information

for *Adv. Sci.*, DOI 10.1002/advs.202308829

Regioselective Arylation of Amidoaryne Precursors via Ag-Mediated Intramolecular Oxy-Argentation

Yong-Ju Kwon, Ye-Jin Kong, Min-Jung Lee, Eun-Hye Lim, Jaesung Kwak and Won-Suk Kim**

Supporting Information

Regioselective Arylation of Amidoaryne Precursors via Ag-Mediated Intramolecular Oxy-argentation

Yong-Ju Kwon,[a] Ye-Jin Kong,[a] Min-Jung Lee,[a] Eun-Hye Lim,[a]
Jaesung Kwak*[b][c] & Won-Suk Kim*[a]

[a]Department of Chemistry and Nanoscience, Ewha Womans University, Seoul 03760, Republic of Korea.
[b]Infectious Diseases Therapeutic Research Center, Korea Research Institute of Chemical Technology (KRICT), Daejeon, 34114, Republic of Korea
[c]Division of Medicinal Chemistry and Pharmacology, KRICT School, University of Science and Technology, Daejeon 34114, Republic of Korea

jkwak@krict.re.kr, wonsukk@ewha.ac.kr

Table of Contents

1. General Experimental Information **S2**
2. Preparation of Starting Materials **S3**
3. Comprehensive Data for Reaction Optimization **S10**
4. Substrate Scope **S12**
5. Mechanistic Studies **S25**
6. Computational Studies **S30**
7. References **S32**
Appendix 1. ^{1}H, ^{13}C, and ^{11}B NMR Spectra **S33**
Appendix 2. Spectra Cartesian Coordinates for Optimized Structures **S103**

1. General Experimental Information

Unless otherwise indicated, all chemical reagents were purchased from commercial suppliers and were used without further purification. All reactions were carried out in oven-dried glassware equipped with a magnetic stir bar. Reactions were monitored by thin layer chromatography (TLC) with 0.25-mm pre-coated silica gel plates (Kieselgel 60 F_{254}). Products were detected by viewing under a UV light, by staining with a ninhydrin solution composed of ninhydrin, acetic acid, and EtOH, a $KMnO_4$ solution composed of potassium carbonate, sodium hydroxide, and water, and an anisaldehyde solution composed of acetic acid, sulfuric acid, and MeOH. Flash column chromatography was performed on silica gel (70-230 mesh). Yields refer to chromatographically and spectroscopically pure compounds unless otherwise noted. ^{1}H, ^{13}C, and ^{11}B spectra were recorded on a Bruker AM-300 spectrometer at the National Research Facilities and Equipment Center (Nano-Bio Energy Materials Center) at Ewha Womans University. Chemical shifts are reported as δ values relative to internal $SiMe_4$ or chloroform-*d* (δ 0.00 or δ 7.26 for ^{1}H and δ 77.0 for ^{13}C), dichloromethane-d_2 (δ 5.32 for ^{1}H and δ 54.0 for ^{13}C) or pyridine-d_5 (δ 8.74, δ 7.58, and δ 7.22 for ^{1}H and δ 150.3, δ 135.9, and δ 123.9 for ^{13}C). HRMS data were obtained by electron ionization and fast atom bombardment with a double-focusing high-resolution magnetic sector mass analyzer at the Korea Basic Science Institute Mass Spectrometry Service Center. Optical rotation of $[\alpha]_D$ values of enantiomerically pure compounds were measured using a A. KRÜSS Optronic GmbH P8000-PT polarimeter at room temperature in CH_2Cl_2.

2. Preparation of Starting Materials
2.1. Preparation of Aryne Precursors 1

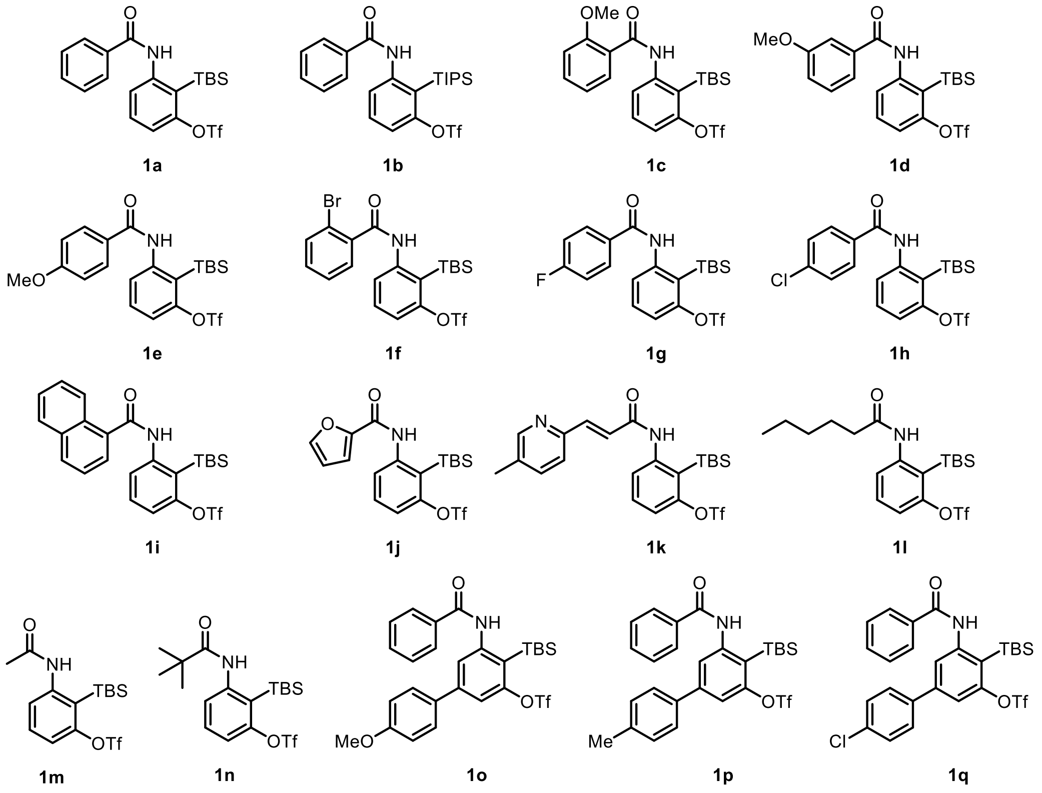

Figure S1. Amidoaryne Precursors **1** (**1a–1q**)

1j[1] and **1k**[1] were synthesized according to the literature methods and the others were synthesized following the general procedure.

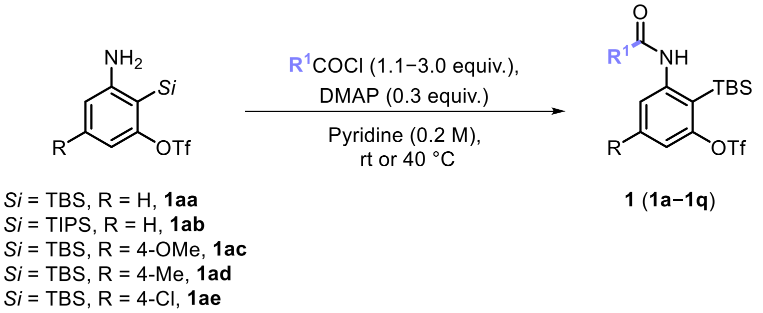

Scheme S1. Synthetic Method for Amidobenzyne Precursor **1**

General Procedure for the Synthesis of Amidobenzyne Precursors:

A pyridine (0.2 M) solution containing aniline derivative (1.0 equiv.) and 4-(dimethylamino)pyridine (0.3 equiv.) was placed in an ice bath at 0 °C, and acyl chloride (1.1–3.0 equiv.) was added at the same temperature. Then the reaction mixture was stirred at room temperature or in an oil bath at 40 °C. After completion of the reaction as monitored by TLC analysis, NaCl(sat.) and $CuSO_4$(sat.) were added to the reaction mixture and extracted with diethyl ether (3 x 10 mL). The organic phase was collected, dried over anhydrous $MgSO_4$, filtered, and concentrated under reduced pressure. The crude product was purified by silica gel column chromatography to obtain the desired product **1**.

3-Benzamido-2-(*tert*-butyldimethylsilyl)phenyl trifluoromethanesulfonate (1a)

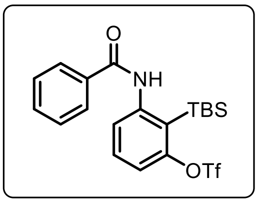

The general procedure was used employing **1aa** (100.0 mg, 0.281 mmol) and benzoyl chloride (0.04 mL, 0.365 mmol). The reaction mixture was stirred for 2 h at room temperature. Flash column chromatography on silica gel using hexane/diethyl ether (8:1) provided pure **1a** (124.1 mg, 0.270 mmol, 96%) as a white solid. **m.p**: 134−136 °C; **R*f*** 0.30 (hexane/ethyl acetate = 5:1); **^{1}H NMR** (300 MHz, $CDCl_3$) δ 8.01 (brs, 1H), 7.85–7.80 (m, 3H), 7.59–7.48 (m, 4H), 7.30 (dd, *J* = 8.4 and 0.9 Hz, 1H), 0.94 (s, 9H), 0.42 (s, 6H); **^{13}C{^{1}H} NMR** (75 MHz, $CDCl_3$) δ 165.9, 156.3, 144.2, 134.4, 132.2, 131.5, 129.0, 127.0, 125.0, 122.0, 118.5 (q, *J* = 320.6 Hz). 115.7, 26.5, 18.9, -1.4; **HRMS** (FAB) *m/z* $[M+H]^+$ calcd for $C_{20}H_{25}F_3NO_4SSi$ 460.1226, found 460.1229.

3-Benzamido-2-(triisopropylsilyl)phenyl trifluoromethanesulfonate (1b)

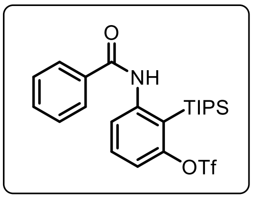

The general procedure was used employing **1ab**[1] (100.0 mg, 0.252 mmol) and benzoyl chloride (0.04 mL, 0.328 mmol). The reaction mixture was stirred for 2 h at room temperature. Flash column chromatography on silica gel using hexane/diethyl ether (8:1) provided pure **1b** (119.9 mg, 0.239 mmol, 95%) as a white solid. **m.p**: 118−120 °C; **R*f*** 0.34 (hexane/ethyl acetate = 3:1); **^{1}H NMR** (300 MHz, $CDCl_3$) δ 8.02 (s, 1H), 7.84 (dt, *J* = 7.0 and 1.6 Hz, 3H), 7.66–7.41 (m, 4H), 7.29 (dd, *J* = 8.4 and 1.0 Hz, 1H), 1.62 (hept, *J* = 7.5 Hz, 3H), 1.14 (s, 9H); **^{13}C NMR** (75 MHz, $CDCl_3$) δ 165.9, 156.4, 144.6, 134.6, 132.3, 131.3, 129.0, 126.9, 125.6, 121.7, 118.5 (q, *J* = 318.8 Hz), 116.7 (q, *J* = 3.0 Hz), 19.1, 13.5; **HRMS** (FAB) *m/z* $[M+H]^+$ calcd for $C_{23}H_{31}F_3NO_4SSi$ 502.1617, found 502.1699.

2-(*tert*-Butyldimethylsilyl)-3-(2-methoxybenzamido)phenyl trifluoromethanesulfonate (1c)

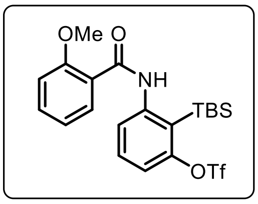

The general procedure was used employing **1aa** (100.0 mg, 0.281 mmol) and 2-methoxybenzoyl chloride (0.05 mL, 0.365 mmol). The reaction mixture was stirred for 6 h at room temperature. Flash column chromatography on silica gel using hexane/diethyl ether (8:1) provided pure **1c** (136.1 mg, 0.278 mmol, 99%) as a white solid. **m.p**: 114−116 °C; **R*f*** 0.15 (hexane/diethyl ether = 3:1); **^{1}H NMR** (300 MHz, $CDCl_3$) δ 9.31 (brs, 1H), 8.25 (dd, *J* = 7.9 and 1.9 Hz, 1H), 7.59–7.45 (m, 3H), 7.35 (d, *J* = 8.4 Hz, 1H), 7.14–7.05 (m, 2H), 3.96 (s, 3H), 0.95 (s, 9H), 0.39 (s, 6H); **^{13}C{^{1}H} NMR** (75 MHz, $CDCl_3$) δ 164.4, 157.1, 156.4, 144.3, 133.5, 132.5, 130.9, 128.0, 124.7, 121.5, 121.0, 118.4 (q, *J* = 320.6 Hz), 116.1 (d, *J* = 2.5 Hz), 111.3, 55.7, 26.5, 18.4, -1.7; **HRMS** (FAB) *m/z* $[M+H]^+$ calcd for $C_{21}H_{27}F_3NO_5SSi$ 490.1331, found 490.1328.

2-(*tert*-Butyldimethylsilyl)-3-(3-methoxybenzamido)phenyl trifluoromethanesulfonate (1d)

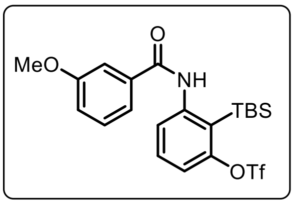

The general procedure was used employing **1aa** (100.0 mg, 0.281 mmol) and 3-methoxybenzoyl chloride (0.05 mL, 0.365 mmol). The reaction mixture was stirred for 4 h at room temperature. Flash column chromatography on silica gel using hexane/diethyl ether (8:1) provided pure **1d** (129.3 mg, 0.264 mmol, 94%) as a white solid. **m.p**: 134−136 °C; **R*f*** 0.17 (hexane/diethyl ether = 3:1); **^{1}H NMR** (300 MHz, $CDCl_3$) δ 8.01 (brs, 1H), 7.80 (dd, *J* = 8.1 and 0.9 Hz, 1H), 7.50 (t, *J* = 8.3 Hz, 1H), 7.44–7.26 (m, 4H), 7.12 (ddd, *J* = 8.1, 2.6 and 1.2 Hz, 1H), 3.87 (s, 3H), 0.95 (s, 9H), 0.43 (s, 6H); **^{13}C{^{1}H} NMR** (75 MHz, $CDCl_3$) δ 165.7, 160.1, 156.3, 144.2, 135.9, 131.5, 130.0, 124.9, 121.9,

118.6, 118.5 (q, J = 320.5 Hz), 118.4, 115.7 (d, J = 2.3 Hz), 112.4, 55.4, 26.5, 18.9, -1.4; **HRMS** (FAB) *m/z* $[M+H]^+$ calcd for $C_{21}H_{27}F_3NO_5SSi$ 490.1331, found 490.1334.

2-(*tert*-Butyldimethylsilyl)-3-(4-methoxybenzamido)phenyl trifluoromethanesulfonate (1e)

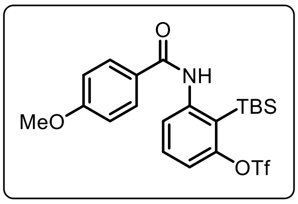

The general procedure was used employing **1aa** (100.0 mg, 0.281 mmol) and 4-methoxybenzoyl chloride (0.05 mL, 0.365 mmol). The reaction mixture was stirred for 17 h at room temperature. Flash column chromatography on silica gel using hexane/diethyl ether (5:1) provided pure **1e** (136.2 mg, 0.278 mmol, 99%) as a white solid. **m.p**: 154−156 °C; $\mathbf{R_f}$ 0.10 (hexane/diethyl ether = 3:1); **^{1}H NMR** (300 MHz, $CDCl_3$) δ 7.94 (brs, 1H), 7.81 (d, J = 8.9 Hz, 2H), 7.75 (d, J = 8.0 Hz, 1H), 7.48 (t, J = 8.3 Hz, 1H), 7.28 (dd, J = 8.4 and 0.9 Hz, 1H), 6.99 (d, J = 8.8 Hz, 2H), 3.88 (s, 3H), 0.95 (s, 9H), 0.43 (s, 6H); **^{13}C{^{1}H} NMR** (75 MHz, $CDCl_3$) δ 165.4, 162.7, 156.3, 144.4, 131.4, 128.9, 126.4, 125.2, 122.1, 118.5 (q, J = 320.4 Hz), 115.5 (d, J = 2.4 Hz), 114.1, 55.4, 26.5, 18.8, -1.4; **HRMS** (FAB) *m/z* $[M+H]^+$ calcd for $C_{21}H_{27}F_3NO_5SSi$ 490.1331, found 490.1329.

3-(2-Bromobenzamido)-2-(*tert*-butyldimethylsilyl)phenyl trifluoromethanesulfonate (1f)

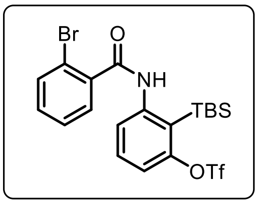

The general procedure was used employing **1aa** (100.0 mg, 0.281 mmol) and 2-bromobenzoyl chloride (0.04 mL, 0.309 mmol). The reaction mixture was stirred for 9 h in an oil bath at 40 °C. Flash column chromatography on silica gel using hexane/diethyl ether (10:1) provided pure **1f** (142.2 mg, 0.264 mmol, 94%) as a white solid. **m.p**: 88−90 °C; $\mathbf{R_f}$ 0.33 (hexane/diethyl ether = 3:1); **^{1}H NMR** (300 MHz, $CDCl_3$) δ 8.02 (d, J = 8.1 Hz, 1H), 7.80 (brs, 1H), 7.65 (dd, J = 7.9 and 1.3 Hz, 1H), 7.56–7.28 (m, 5H), 0.90 (s, 9H), 0.40 (s, 6H); **^{13}C{^{1}H} NMR** (75 MHz, $CDCl_3$) δ 165.8, 156.2, 143.8, 137.5, 133.7, 131.9, 131.7, 129.1, 127.9, 123.9, 121.4, 118.4 (q, J = 320.4 Hz), 115.8 (d, J = 2.4 Hz), 119.1, 26.4, 18.7, -1.4; **HRMS** (FAB) *m/z* $[M+H]^+$ calcd for $C_{20}H_{24}BrF_3NO_4SSi$ 538.0331, found 538.0328.

2-(*tert*-Butyldimethylsilyl)-3-(4-fluorobenzamido)phenyl trifluoromethanesulfonate (1g)

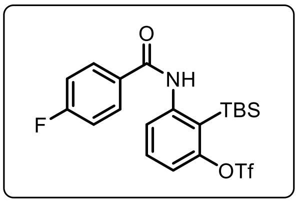

The general procedure was used employing **1aa** (100.0 mg, 0.281 mmol) and 4-fluorobenzoyl chloride (0.04 mL, 0.365 mmol). The reaction mixture was stirred for 2 h at room temperature. Flash column chromatography on silica gel using hexane/diethyl ether (10:1) provided pure **1g** (127.5 mg, 0.267 mmol, 95%) as a white solid. **m.p**: 138−140 °C; $\mathbf{R_f}$ 0.21 (hexane/diethyl ether = 3:1); **^{1}H NMR** (300 MHz, $CDCl_3$) δ 7.98 (s, 1H), 7.88–7.83 (m, 2H), 7.73 (d, J = 8.0 Hz, 1H), 7.49 (t, J = 8.3 Hz, 1H), 7.31 (d, J = 8.7 Hz, 1H), 7.22–7.15 (m, 2H), 0.94 (s, 9H), 0.42 (s, 6H); **^{13}C{^{1}H} NMR** (75 MHz, $CDCl_3$) δ 165.1 (d, J = 253.6 Hz), 164.9, 156.3, 144.0, 131.5, 130.6 (d, J = 3.2 Hz), 129.4 (d, J = 9.1 Hz), 125.1, 122.2, 118.5 (q, J = 320.5 Hz), 116.1 (d, J = 22.0 Hz), 115.9 (d, J = 2.4 Hz), 26.4, 18.8, -1.4; **HRMS** (FAB) *m/z* $[M+H]^+$ calcd for $C_{20}H_{24}F_4NO_4SSi$ 478.1131, found 478.1127.

2-(*tert*-Butyldimethylsilyl)-3-(4-chlorobenzamido)phenyl trifluoromethanesulfonate (1h)

The general procedure was used employing **1aa** (100.0 mg, 0.281 mmol) and 4-chlorobenzoyl chloride (0.05 mL, 0.365 mmol). The reaction mixture was stirred for 4 h in an oil bath at 40 °C. Flash column chromatography on silica gel using

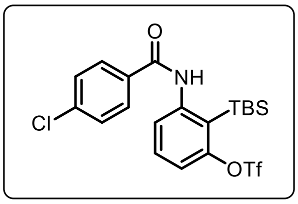

hexane/diethyl ether (10:1) provided pure **1h** (131.9 mg, 0.267 mmol, 95%) as a white solid. **m.p**: 152−154 °C; **R*f*** 0.26 (hexane/diethyl ether = 3:1); **^{1}H NMR** (300 MHz, $CDCl_3$) δ 7.94 (brs, 1H), 7.78 (d, *J* = 8.5 Hz, 3H), 7.54–7.49 (m, 3H), 7.31 (d, *J* = 8.4 Hz, 1H), 0.94 (s, 9H), 0.42 (s, 6H); **^{13}C{^{1}H} NMR** (75 MHz, $CDCl_3$) δ 164.9, 156.3, 143.9, 138.6, 132.8, 131.5, 129.3, 128.4, 125.1, 122.2, 118.5 (q, *J* = 320.5 Hz), 116.0 (d, *J* = 2.4 Hz), 26.4, 18.8, -1.4; **HRMS** (FAB) *m/z* $[M+H]^+$ calcd for $C_{20}H_{24}ClF_3NO_4SSi$ 494.0836, found 494.0840.

3-(1-Naphthamido)-2-(*tert*-butyldimethylsilyl)phenyl trifluoromethanesulfonate (1i)

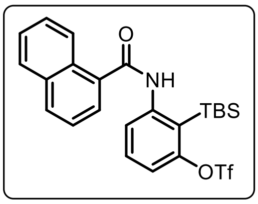

The general procedure was used employing **1aa** (100.0 mg, 0.281 mmol) and 1-naphthoyl chloride (0.06 mL, 0.365 mmol). The reaction mixture was stirred for 8 h at room temperature. Flash column chromatography on silica gel using hexane/diethyl ether (15:1) provided pure **1i** (139.1 mg, 0.273 mmol, 97%) as a white solid. **m.p**: 84−86 °C; **R*f*** 0.36 (hexane/diethyl ether = 3:1); **^{1}H NMR** (300 MHz, $CDCl_3$) δ 8.37–8.33 (m, 1H), 8.11 (d, *J* = 8.1 Hz, 1H), 8.04 (brs, 1H), 8.00 (d, *J* = 8.3 Hz, 1H), 7.92–7.88 (m, 1H), 7.72 (d, *J* = 7.0 Hz, 1H), 7.58–7.49 (m, 4H), 7.30 (d, *J* = 8.7 Hz, 1H), 0.86 (s, 9H), 0.33 (s, 6H); **^{13}C{^{1}H} NMR** (75 MHz, $CDCl_3$) δ 167.4, 156.3, 144.4, 133.8, 133.7, 131.6, 131.6, 129.9, 128.4, 127.4, 126.7, 125.1, 125.0, 124.6, 123.6, 120.9, 118.5 (q, *J* = 320.5 Hz), 115.4 (d, *J* = 2.5 Hz), 26.4, 18.8, -1.5; **HRMS** (FAB) *m/z* $[M+H]^+$ calcd for $C_{24}H_{27}F_3NO_4SSi$ 510.1382, found 510.1380.

2-(*tert*-Butyldimethylsilyl)-3-(furan-2-carboxamido)phenyl trifluoromethanesulfonate (1j)

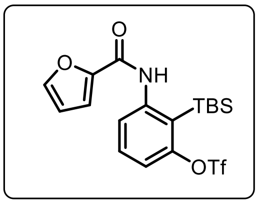

The general procedure was used employing **1aa** (100.0 mg, 0.281 mmol) and 2-furoyl chloride (0.08 mL, 0.843 mmol). The reaction mixture was stirred for 18 h at room temperature. Flash column chromatography on silica gel using hexane/diethyl ether (8:1) provided pure **1j** (106.1 mg, 0.236 mmol, 84%) as a white solid. **m.p**: 76−78 °C; **R*f*** 0.20 (hexane/diethyl ether = 3:1); **^{1}H NMR** (300 MHz, $CDCl_3$) δ 8.30 (brs, 1H), 7.97 (dd, *J* = 8.2 and 0.9 Hz, 1H), 7.57 (dd, *J* = 1.8 and 0.8 Hz, 1H), 7.49 (t, *J* = 8.3 Hz, 1H), 7.28 (ddd, *J* = 6.0, 2.5 and 1.0 Hz, 2H), 6.59 (dd, *J* = 3.5 and 1.8 Hz, 1H), 1.00 (s, 9H), 0.53 (s, 6H); **^{13}C{^{1}H} NMR** (75 MHz, $CDCl_3$) 156.3, 156.1, 147.5, 144.6, 143.5, 131.5, 124.0, 121.5, 118.5 (q, *J* = 320.5 Hz), 115.5 (d, *J* = 2.4 Hz), 116.1, 112.7, 26.5, 18.9, -1.5; **HRMS** (EI) *m/z* $[M]^+$ calcd for $C_{18}H_{22}F_3NO_5SSi$ 449.0940, found 449.0937.

(*E*)-2-(tert-Butyldimethylsilyl)-3-[3-(5-methylpyridin-2-yl)acrylamido]phenyl trifluoromethanesulfonate (1k)

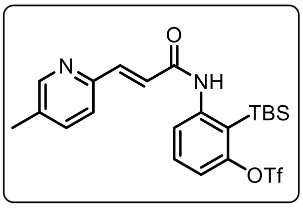

A DMF (0.94 mL) solution containing **1aa** (100.0 mg, 0.281 mmol), (*E*)-3-(5-methylpyridin-2-yl)acryloyl chloride (78.0 mg, 0.562 mmol) and K_2CO_3 (905.0 mg, 0.562 mmol) was placed in an oil bath at 80 °C and stirred for 1 h. After completion of the reaction as monitored by TLC analysis, NaCl(sat.) and water were added to the reaction mixture and extracted with diethyl ether (3 x 10 mL). The organic phase was collected, dried over anhydrous $MgSO_4$, filtered, and concentrated under reduced pressure. Flash column chromatography on silica gel using hexane/ethyl acetate = 4:1) provided pure **1k** (123.7 mg, 0.247 mmol, 88%) as a white solid. **^{1}H NMR** (300 MHz, $CDCl_3$) δ 8.50 (s, 1H), 7.91 (s, 1H), 7.76 (d, *J* = 15.0 Hz, 1H), 7.57–7.46 (m, 3H), 7.29 (t, *J* = 8.7 Hz, 2H), 7.01 (d, *J* = 14.9 Hz, 1H), 2.39 (s, 3H), 0.98 (s, 9H), 0.54 (s, 6H); **^{13}C{^{1}H} NMR** (75 MHz, $CDCl_3$) δ 163.9, 156.2, 150.7, 149.9, 144.1, 142.1, 137.1, 134.4, 131.4, 124.8, 124.5, 122.6, 121.5, 118.4 (q, *J* = 320.6 Hz), 115.6, 26.5, 18.8, 18.4, −1.2; Data are consistent

with those reported in the literature.[1]

2-(*tert*-Butyldimethylsilyl)-3-hexanamidophenyl trifluoromethanesulfonate (1l)

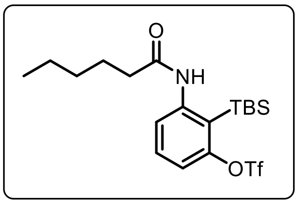

The general procedure was used employing **1aa** (100.0 mg, 0.281 mmol) and hexanoyl chloride (0.05 mL, 0.365 mmol). The reaction mixture was stirred for 11 h in an oil bath at 40 °C. Flash column chromatography on silica gel using hexane/diethyl ether (10:1) provided pure **1l** (117.5 mg, 0.259 mmol, 92%) as a colorless oil. **R*f*** 0.28 (hexane/diethyl ether = 3:1); **^{1}H NMR** (300 MHz, $CDCl_3$) δ 7.89 (d, *J* = 8.2 Hz, 1H), 7.44 (t, *J* = 8.3 Hz, 1H), 7.35 (brs, 1H), 7.22 (d, *J* = 8.4 Hz, 1H), 2.35–2.30 (m, 2H), 1.79–1.69 (m, 2H), 1.38–1.33 (m, 4H), 0.97 (s, 9H), 0.94–0.89 (m, 3H), 0.51 (s, 6H); **^{13}C{^{1}H} NMR** (75 MHz, $CDCl_3$) δ 171.0, 156.2, 144.3, 131.5, 123.6, 120.6, 118.5 (q, *J* = 320.6 Hz), 116.3, 115.1 (d, *J* = 1.5 Hz), 37.7, 31.4, 26.5, 24.9, 22.4, 18.8, 13.8, -1.2; **HRMS** (FAB) *m/z* $[M+H]^+$ calcd for $C_{19}H_{31}F_3NO_4SSi$ 454.1695, found 454.1697.

3-Acetamido-2-(tert-butyldimethylsilyl)phenyl trifluoromethanesulfonate (1m)

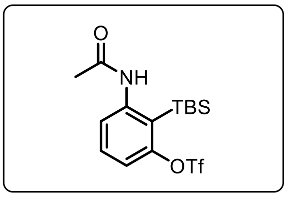

The general procedure was used employing **1aa** (100.0 mg, 0.281 mmol) and acetyl chloride (0.04 mL, 0.563 mmol). The reaction mixture was stirred for 18 h at room temperature. Flash column chromatography on silica gel using hexane/diethyl ether (15:1) provided pure **1m** (100.5 mg, 0.253 mmol, 90%) as a white solid. **m.p**: 68−71 °C; **R*f*** 0.28 (hexane/diethyl ether = 3:1); **^{1}H NMR** (300 MHz, $CDCl_3$) δ 7.83 (d, *J* = 8.1 Hz, 1H), 7.47 (t, *J* = 8.3 Hz, 1H), 7.40 (s, 1H), 7.25 (d, *J* = 8.4 Hz, 1H), 2.18 (s, 3H), 0.98 (s, 10H), 0.52 (s, 6H); **^{13}C{^{1}H} NMR** (75 MHz, $CDCl_3$) δ 168.0, 156.3, 144.1, 131.6, 124.1, 121.0, 118.5 (q, *J* = 318.0 Hz), 115.5, 26.5, 24.5, 18.9, -1.3; **HRMS** (FAB) *m/z* $[M+H]^+$ calcd for $C_{15}H_{23}F_3NO_4SSi$ 398.0991, found 398.1071.

2-(*tert*-Butyldimethylsilyl)-3-pivalamidophenyl trifluoromethanesulfonate (1n)

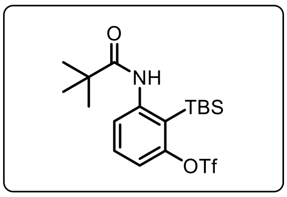

The general procedure was used employing **1aa** (100.0 mg, 0.281 mmol) and pivaloyl chloride (0.07 mL, 0.563 mmol). The reaction mixture was stirred for 18 h at room temperature. Flash column chromatography on silica gel using hexane/diethyl ether (15:1) provided pure **1n** (122.2 mg, 0.278 mmol, 99%) as a white solid. **^{1}H NMR** (300 MHz, $CDCl_3$) δ 7.58 (d, *J* = 8.1 Hz, 1H), 7.43 (t, *J* = 8.3 Hz, 1H), 7.36 (brs, 1H), 7.28−7.25 (m, 1H), 1.33 (s, 9H), 0.97 (s, 9H), 0.48 (s, 6H); ^{13}C{^{1}H} NMR (75 MHz, $CDCl_3$) δ 177.0, 156.3, 144.7, 131.0, 126.3, 122.8, 118.4 (q, *J* = 320.8 Hz), 115.5 (d, *J* = 2.3 Hz), 39.4, 27.4, 26.5, 18.7, −1.2; Data are consistent with those reported in the literature.[1]

5-Benzamido-4-(*tert*-butyldimethylsilyl)-4'-methoxy-[1,1'-biphenyl]-3-yl trifluoromethanesulfonate (1o)

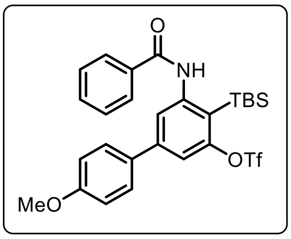

The general procedure was used employing **1ac** (100.2 mg, 0.217 mmol) and benzoyl chloride (0.04 mL, 0.326 mmol). The reaction mixture was stirred for 9 h at room temperature. Flash column chromatography on silica gel using hexane/diethyl ether (10:1) provided pure **1o** (101.8 mg, 0.180 mmol, 83%) as a white solid. **m.p**: 126−128 °C; **R*f*** 0.31 (hexane/diethyl ether = 3:1); **^{1}H NMR** (300 MHz, $CDCl_3$) δ 8.06 (brs, 1H), 8.04 (d, *J* = 1.5 Hz, 1H), 7.88–7.84 (m, 2H), 7.61–7.48 (m, 6H), 7.03–6.92 (m, 2H), 3.84 (s, 3H), 0.97 (s, 9H), 0.44 (s, 6H); **^{13}C{^{1}H} NMR** (75 MHz, $CDCl_3$) δ 165.9, 160.1, 156.9, 144.4, 144.37, 134.4, 132.2, 130.8, 129.0, 128.2, 126.9, 122.8,

119.2, 118.5 (q, J = 320.5 Hz), 113.7 (d, J = 1.9 Hz), 114.4, 55.3, 26.5, 18.9, -1.5; **HRMS** (FAB) *m/z* $[M+H]^+$ calcd for $C_{27}H_{31}F_3NO_5SSi$ 566.1644, found 566.1647.

5-Benzamido-4-(*tert*-butyldimethylsilyl)-4'-methyl-[1,1'-biphenyl]-3-yl trifluoromethanesulfonate (1p)

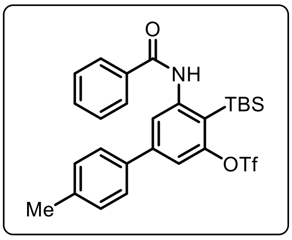

The general procedure was used employing **1ad** (100.0 mg, 0.224 mmol) and benzoyl chloride (0.03 mL, 0.291 mmol). The reaction mixture was stirred for 9 h at room temperature. Flash column chromatography on silica gel using hexane/diethyl ether (10:1) provided pure **1p** (87.4 mg, 0.159 mmol, 71%) as a white solid. **m.p**: 130−132 °C; $\mathbf{R_{\mathit{f}}}$ 0.28 (hexane/diethyl ether = 3:1); ^{1}**H NMR** (300 MHz, $CDCl_3$) δ 8.06 (d, J = 1.5 Hz, 2H), 7.87–7.84 (m, 2H), 7.58–7.48 (m, 6H), 7.28–7.24 (m, 2H), 2.39 (s, 3H), 0.97 (s, 9H), 0.44 (s, 6H); ^{13}**C{^{1}H} NMR** (75 MHz, $CDCl_3$) δ 166.0, 156.8, 144.7, 144.4, 138.6, 135.6, 134.4, 132.3, 129.7, 129.0, 127.0, 126.9, 123.2, 119.7, 118.5 (q, J = 320.6 Hz), 114.0 (d, J = 2.3 Hz), 26.5, 21.1, 18.9, -1.5; **HRMS** (FAB) *m/z* $[M+H]^+$ calcd for $C_{27}H_{31}F_3NO_4SSi$ 550.1695, found 550.1698.

5-Benzamido-4-(*tert*-butyldimethylsilyl)-4'-chloro-[1,1'-biphenyl]-3-yl trifluoromethanesulfonate (1q)

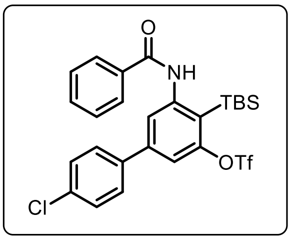

The general procedure was used employing **1ae** (100.2 mg, 0.215 mmol) and benzoyl chloride (0.03 mL, 0.280 mmol). The reaction mixture was stirred for 9 h at room temperature. Flash column chromatography on silica gel using hexane/diethyl ether (20:1) provided pure **1q** (88.4 mg, 0.155 mmol, 72%) as a white solid. **m.p**: 132−134 °C; $\mathbf{R_{\mathit{f}}}$ 0.45 (hexane/diethyl ether = 3:1); ^{1}**H NMR** (300 MHz, $CDCl_3$) δ 8.10 (brs, 1H), 8.07 (s, 1H), 7.86 (d, J = 6.9 Hz, 2H), 7.62–7.41 (m, 8H), 0.97 (s, 9H), 0.45 (s, 6H); ^{13}**C{^{1}H} NMR** (75 MHz, $CDCl_3$) δ 166.0, 156.8, 144.6, 143.4, 136.9, 134.8, 134.3, 132.3, 129.2, 129.0, 128.4, 126.9, 123.1, 120.5, 118.5 (q, J = 320.6 Hz), 113.9 (d, J = 2.0 Hz), 26.5, 18.9, -1.5; **HRMS** (FAB) *m/z* $[M+H]^+$ calcd for $C_{26}H_{28}ClF_3NO_4SSi$ 570.1149, found 570.1154.

2.2. Preparation of (Hetero)Aryl or Alkenyl Iodides

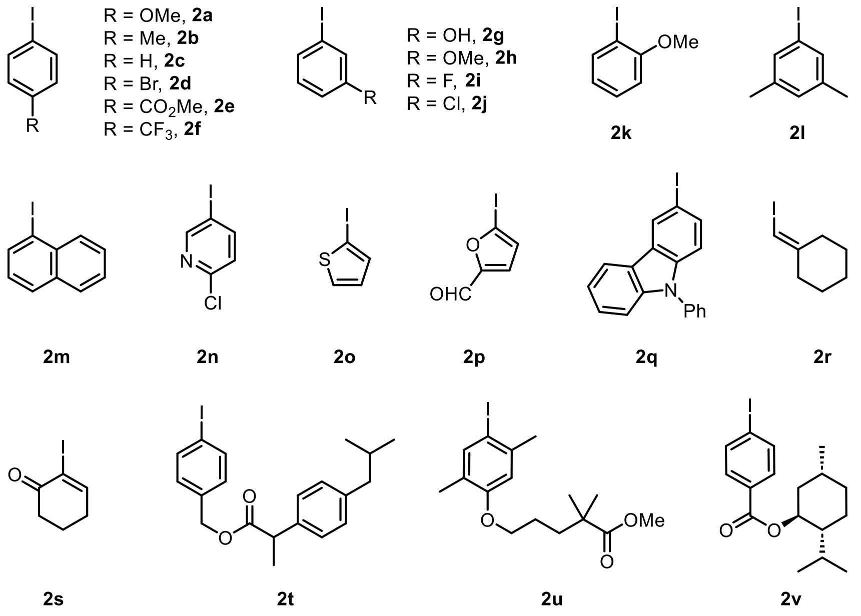

Figure S2. (Hetero)aryl or Alkenyl Iodides Utilized in This Paper

2a−**2p** were purchased from commercial suppliers and **2r**[2], **2s**[3], **2t**−**2v**[4] were synthesized according to the literature methods.

3. Comprehensive Data for Reaction Optimization

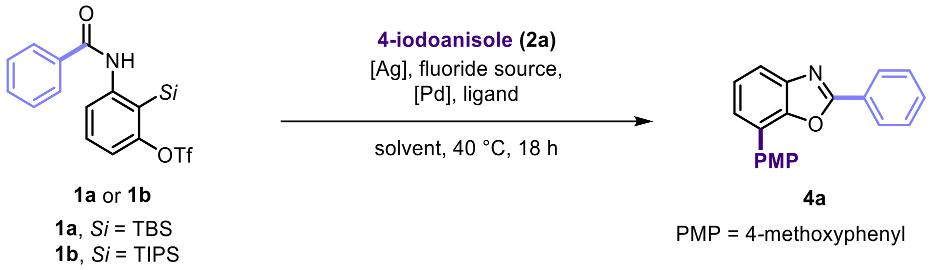

General Procedure for Reaction Optimization:

A sealed tube was charged with **1a** or **1b** (0.39 mmol, 1.3 equiv.), 4-iodoanisole **2a** (0.3 mmol, 1.0 equiv.), silver salts, fluoride source, Pd source and ligand. The sealed tube and contents were placed under vacuum and back-filled with argon under a Schlenk line three times. Degassed anhydrous solvent (3.0 mL, 0.1 M) then added under argon, and the reaction mixture was stirred in an oil bath at 40 °C for 18 h. After completion of the reaction as monitored by TLC analysis, the reaction mixture was cooled to room temperature, celite-filtered with diethyl ether (3 × 10 mL) and concentrated under reduced pressure. The crude product was purified by flash chromatography on silica gel to obtain desired product **4a**.

Table S1. Solvent Screening[a]

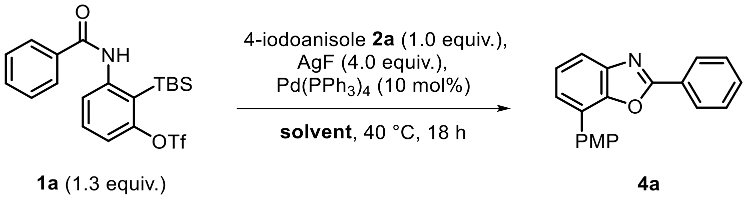

entry	solvent	conversion (%) of **2a**[b]	conversion (%) of **1a**[b]	yield (%) of **4a**[b]
1	EtCN	40	>99	7
2	PhCN	18	99	2
3	PhMe	18	90	4
4	THF	21	82	11
5	1,4-dioxane	49	93	0
6	DME	65	83	1

[a]Reactions were performed on a 0.1 mmol scale following the general procedure. [b]Yields were determined by ^{1}H NMR analysis using ethylene carbonate as an internal standard.

Table S2. Catalyst and Ligand Screening[a]

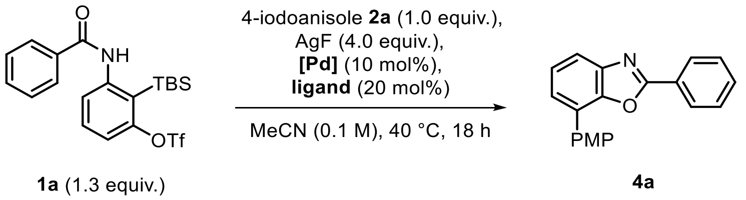

entry	[Pd]	ligand	conversion (%) of **2a**[b]	conversion (%) of **1a**[b]	yield (%) of **4a**[b]
1	$Pd(PPh_3)_2Cl_2$	-	81	>99	65
2	$Pd(PPh_3)_2Cl_2$	PPh_3	91	>99	78
3	$Pd(PhCN)Cl_2$	PPh_3	73	>99	54
4	$PdCl_2$	PPh_3	82	>99	63
5	$Pd_2(dba)_3$	PPh_3	85	>99	60

6	$Pd_2(dba)_3{\cdot}CHCl_3$	PPh_3	83	>99	54
7	$Pd(OAc)_2$	PPh_3	74	>99	44
8	$Pd(MeCN)_2Cl_2$	PPh_3	78	>99	49
9	$Pd(MeCN)_2Cl_2$	P(*o*-tol)$_3$	49	>99	32
10	$Pd(MeCN)_2Cl_2$	dppp	22	>99	12
11	$Pd(MeCN)_2Cl_2$	dppe	15	>99	0
12	$Pd(MeCN)_2Cl_2$	Ad_2BnP	9	>99	0
13	$Pd(MeCN)_2Cl_2$	dtbpy	22	>99	0
14	$Pd(MeCN)_2Cl_2$	SPhos	22	>99	10
15	$Pd(MeCN)_2Cl_2$	Ruphos	57	>99	13
16	$Pd(MeCN)_2Cl_2$	XPhos	34	>99	15

[a]Reactions were performed on a 0.1 mmol scale following the general procedure. [b]Yields were determined by ^{1}H NMR analysis using ethylene carbonate as an internal standard.

Table S3. Silver Salt and Fluoride Source Screening[a]

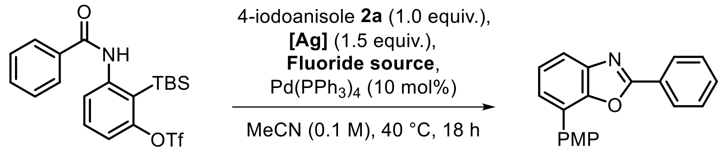

1a (1.3 equiv.) **4a**

entry	[Ag]	fluoride source	conversion (%) of **2a**[b]	conversion (%) of **1a**[b]	yield (%) of **4a**[b]
1	$AgNO_3$	TBAF (3.0 equiv)	60	>99	0
2	$AgNO_3$	CsF (3.0 equiv)	42	87	10
3	$AgClO_4$	CsF (3.0 equiv)	85	83	53
4	$AgSbF_6$	CsF (3.0 equiv)	29	89	18
5	$AgBF_4$	CsF (3.0 equiv)	25	89	5
6	AgOAc	CsF (2.5 equiv)	8	>99	2

[a]Reactions were performed on a 0.1 mmol scale following the general procedure. [b]Yields were determined by ^{1}H NMR analysis using ethylene carbonate as an internal standard.

Table S4. Other Screening[a]

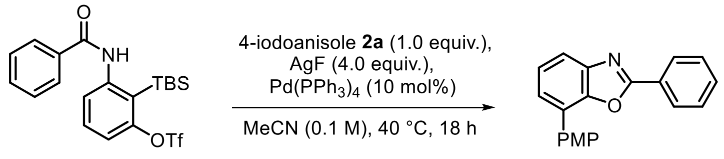

1a (1.3 equiv.) **4a**

entry	variation from the standard conditions B	conversion (%) of **2a**[b]	conversion (%) of **1a**[b]	yield (%) of **4a**[b]
1	30 °C	99	>99	81
2	50 °C	>99	>99	65
3	MeCN (0.2 M)	>99	>99	80
4	MeCN (0.05 M)	>99	>99	84
5	**1a** (1.0 equiv.) and **2a** (1.0 equiv.)	>99	>99	83
6	PMP-Br (1.0 equiv.)	25	>99	1

[a]Reactions were performed on a 0.1 mmol scale following the general procedure. [b]Yields were determined by ^{1}H NMR analysis using ethylene carbonate as an internal standard.

4. Substrate Scope
4.1. Synthesis of 2-Monosubstituted Benzoxazoles (Scheme 1, Conditions A)

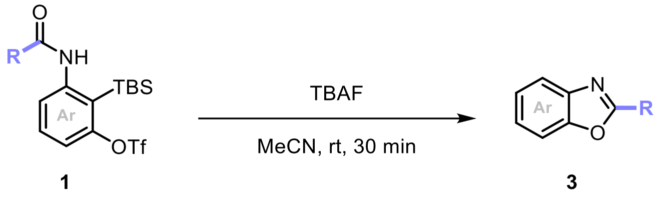

General Procedure for the Synthesis of 2-Monosubstituted Benzoxazoles:

To a solution of **1** (0.25 mmol, 1.0 equiv.) in anhydrous MeCN (8.3 mL, 0.03 M) was added dropwise TBAF (0.55 mmol, 2.2 equiv.) and stirred at room temperature for 30 min. After completion of reaction, the reaction mixture was quenched with H_2O and extracted with diethyl ether (3 x 10 mL). The organic phase was collected, dried over anhydrous $MgSO_4$, filtered, and concentrated under reduced pressure. The crude product was purified by flash column chromatography on silica gel to obtain desired products **3**.

2-Phenylbenzo[*d*]oxazole (3a)

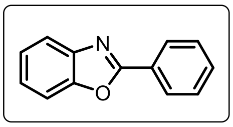

The general procedure was used employing **1a** (114.9 mg, 0.25 mmol). Flash column chromatography on silica gel using hexane/diethyl ether (20:1) provided pure **3a** (46.9 mg, 0.24 mmol, 96%) as a white solid. **^{1}H NMR** (300 MHz, $CDCl_3$) δ 8.27–8.24 (m, 2H), 7.79–7.77 (m, 1H), 7.59–7.56 (m, 1H), 7.54–7.49 (m, 3H), 7.37–7.33 (m, 2H); **^{13}C{^{1}H} NMR** (75 MHz, $CDCl_3$) δ 163.0, 150.7, 142.1, 131.5, 128.9, 127.6, 127.1, 125.1, 124.5, 120.0, 110.5; Data are consistent with those reported in the literature.[5]

2-(2-Methoxyphenyl)benzo[*d*]oxazole (3b)

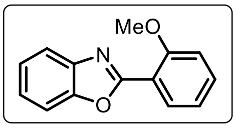

The general procedure was used employing **1c** (122.4 mg, 0.25 mmol). Flash column chromatography on silica gel using hexane/diethyl ether (20:1) provided pure **3b** (52.5 mg, 0.233 mmol, 93%) as a white solid. **^{1}H NMR** (300 MHz, $CDCl_3$) δ 8.14 (dd, *J* = 7.7 and 1.8 Hz, 1H), 7.84–7.81 (m, 1H), 7.60–7.57 (m, 1H), 7.49 (ddd, *J* = 8.3, 7.4 and 1.8 Hz, 1H), 7.36–7.32 (m, 2H), 7.12–7.06 (m, 2H), 4.01 (s, 3H); **^{13}C{^{1}H} NMR** (75 MHz, $CDCl_3$) δ 161.5, 158.4, 150.2, 142.0, 132.7, 131.2, 124.9, 124.2, 120.6, 120.1, 116.0, 112.0, 110.4, 56.1; Data are consistent with those reported in the literature.[5]

2-(3-Methoxyphenyl)benzo[*d*]oxazole (3c)

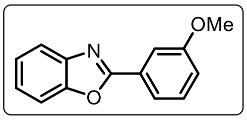

The general procedure was used employing **1d** (122.4 mg, 0.25 mmol). Flash column chromatography on silica gel using hexane/diethyl ether (20:1) provided pure **3c** (52.5 mg, 0.233 mmol, 93%) as a white solid. **^{1}H NMR** (300 MHz, $CDCl_3$) δ 7.85 (ddd, *J* = 7.7, 1.6 and 1.0 Hz, 1H), 7.80–7.76 (m, 2H), 7.58 (ddd, *J* = 6.0, 3.3 and 0.7 Hz, 1H), 7.42 (t, *J* = 8.0 Hz, 1H), 7.38–7.32 (m, 2H), 7.08 (ddd, *J* = 8.3, 2.7 and 1.0 Hz, 1H), 3.91 (s, 3H); **^{13}C{^{1}H} NMR** (75 MHz, $CDCl_3$) δ 163.3, 160.3, 151.1, 142.4, 130.4, 128.7, 125.5, 125.0, 120.5, 120.4, 118.7, 112.2, 111.0, 55.9; Data are consistent with those reported in the literature.[5]

2-(4-Methoxyphenyl)benzo[*d*]oxazole (3d)

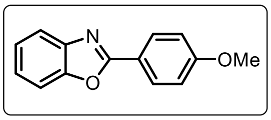

The general procedure was used employing **1e** (122.4 mg, 0.25 mmol). Flash column chromatography on silica gel using hexane/diethyl ether (20:1) provided pure **3d** (51.4 mg,

0.228 mmol, 91%) as a white solid. **^{1}H NMR** (300 MHz, $CDCl_3$) δ 8.19–8.14 (m, 2H), 7.74–7.71 (m, 1H), 7.54–7.50 (m, 1H), 7.33–7.25 (m, 2H), 7.01–6.96 (m, 2H), 3.83 (s, 3H); **^{13}C{^{1}H} NMR** (75 MHz, $CDCl_3$) δ 163.0, 162.2, 150.5, 142.2, 129.3, 124.5, 124.3, 119.6, 119.5, 114.2, 110.3, 55.3; Data are consistent with those reported in the literature.[5]

2-(2-Bromophenyl)benzo[*d*]oxazole (3e)

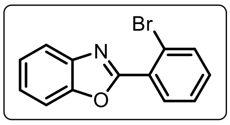

The general procedure was used employing **1f** (134.6 mg, 0.25 mmol). Flash column chromatography on silica gel using hexane/diethyl ether (20:1) provided pure **3e** (68.0 mg, 0.248 mmol, 99%) as a white solid. **^{1}H NMR** (300 MHz, $CDCl_3$) δ 8.08 (dd, *J* = 7.8 and 1.8 Hz, 1H), 7.87–7.84 (m, 1H), 7.77 (dd, *J* = 8.0 and 1.3 Hz, 1H), 7.63–7.60 (m, 1H), 7.48–7.32 (m, 4H); **^{13}C{^{1}H} NMR** (75 MHz, $CDCl_3$) δ 161.5, 150.6, 141.6, 134.6, 132.1, 131.9, 128.3, 127.4, 125.5, 124.6, 121.9, 120.5, 110.7; Data are consistent with those reported in the literature.[6]

2-(4-Fluorophenyl)benzo[*d*]oxazole (3f)

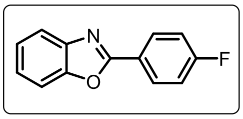

The general procedure was used employing **1g** (119.4 mg, 0.25 mmol). Flash column chromatography on silica gel using hexane/diethyl ether (20:1) provided pure **3f** (48.6 mg, 0.228 mmol, 91%) as a white solid. **^{1}H NMR** (300 MHz, $CDCl_3$) δ 8.28–8.24 (m, 2H), 7.79–7.74 (m, 1H), 7.59–7.55 (m, 1H), 7.39–7.33 (m, 2H), 7.24–7.18 (m, 2H); **^{13}C{^{1}H} NMR** (75 MHz, $CDCl_3$) δ 164.8 (d, *J* = 252.6 Hz), 162.1, 150.7, 142.0, 129.8 (d, *J* = 8.8 Hz), 125.1, 124.6, 123.5 (d, *J* = 3.2 Hz), 119.9, 116.1 (d, *J* = 22.2 Hz), 110.5; Data are consistent with those reported in the literature.[5]

2-(4-Chlorophenyl)benzo[*d*]oxazole (3g)

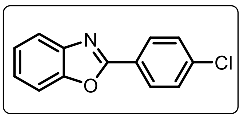

The general procedure was used employing **1h** (123.5 mg, 0.25 mmol). Flash column chromatography on silica gel using hexane/diethyl ether (20:1) provided pure **3g** (54.7 mg, 0.238 mmol, 95%) as a white solid. **^{1}H NMR** (300 MHz, $CDCl_3$) δ 8.19–8.14 (m, 2H), 7.79–7.73 (m, 1H), 7.59–7.53 (m, 1H), 7.50–7.46 (m, 2H), 7.38–7.32 (m, 2H); **^{13}C{^{1}H} NMR** (75 MHz, $CDCl_3$) δ 162.0, 150.7, 141.9, 137.7, 129.2, 128.8, 125.6, 125.3, 124.7, 120.0, 110.6; Data are consistent with those reported in the literature.[5]

2-(Naphthalen-1-yl)benzo[*d*]oxazole (3h)

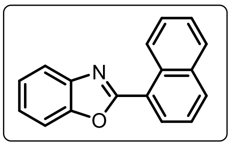

The general procedure was used employing **1i** (127.4 mg, 0.25 mmol). Flash column chromatography on silica gel using hexane/diethyl ether (20:1) provided pure **3h** (55.9 mg, 0.228 mmol, 91%) as a white solid. **^{1}H NMR** (300 MHz, $CDCl_3$) δ 9.47 (dd, *J* = 8.6 and 1.1 Hz, 1H), 8.42 (dd, *J* = 7.4 and 1.3 Hz, 1H), 8.01 (d, *J* = 8.3 Hz, 1H), 7.94–7.85 (m, 2H), 7.73–7.67 (m, 1H), 7.65–7.55 (m, 3H), 7.42–7.36 (m, 2H); **^{13}C{^{1}H} NMR** (75 MHz, $CDCl_3$) δ 162.8, 150.1, 142.3, 133.9, 132.3, 130.7, 129.3, 128.6, 127.9, 126.4, 126.3, 125.3, 124.9, 124.5, 123.6, 120.3, 110.5; Data are consistent with those reported in the literature.[5]

2-(Furan-2-yl)benzo[*d*]oxazole (3i)

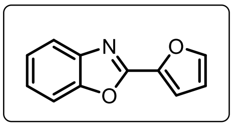

The general procedure was used employing **1j** (112.4 mg, 0.25 mmol). Flash column chromatography on silica gel using hexane/diethyl ether (20:1) provided pure **3i** (45.9 mg, 0.248 mmol, 99%) as a white solid. **^{1}H NMR** (300 MHz, $CDCl_3$) δ 7.78–7.75 (m, 1H), 7.67 (dd, *J* = 1.8

and 0.8 Hz, 1H), 7.58–7.55 (m, 1H), 7.37–7.34 (m, 2H), 7.28 (dd, J = 3.5 and 0.8 Hz, 1H), 6.62 (dd, J = 3.5 and 1.7 Hz, 1H); **^{13}C{^{1}H} NMR** (75 MHz, $CDCl_3$) δ 155.3, 150.1, 145.7, 142.6, 141.6, 125.3, 124.8, 120.1, 114.2, 112.2, 110.5; Data are consistent with those reported in the literature.[7]

(*E*)-2-[2-(5-Methylpyridin-2-yl)vinyl]benzo[*d*]oxazole (3j)

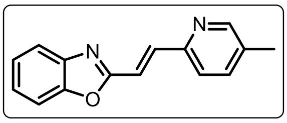

The general procedure was used employing **1k** (125.2 mg, 0.25 mmol). Flash column chromatography on silica gel using hexane/diethyl ether (2:1) provided pure **3j** (53.2 mg, 0.225 mmol, 90%) as a white solid. **m.p**: 150−152 °C; **R*f*** 0.08 (hexane/diethyl ether = 3:1); **^{1}H NMR** (300 MHz, $CDCl_3$) δ 8.51 (d, J = 2.2 Hz, 1H), 7.82–7.73 (m, 2H), 7.58–7.51 (m, 3H), 7.38–7.31 (m, 3H), 2.38 (s, 3H); **^{13}C{^{1}H} NMR** (75 MHz, $CDCl_3$) δ 162.6, 150.8, 150.75, 150.5, 142.2, 138.3, 137.1, 133.7, 125.4, 124.5, 123.3, 120.0, 116.8, 110.4, 18.5; **HRMS** (EI) *m/z* $[M]^+$ calcd for $C_{15}H_{12}N_2O$ 236.0950, found 236.0947.

2-Pentylbenzo[*d*]oxazole (3k)

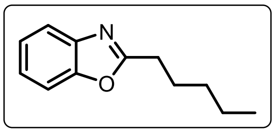

The general procedure was used employing **1l** (113.4 mg, 0.25 mmol). Flash column chromatography on silica gel using hexane/diethyl ether (20:1) provided pure **3k** (43.2 mg, 0.228 mmol, 91%) as a colorless oil. **^{1}H NMR** (300 MHz, $CDCl_3$) δ 7.70–7.64 (m, 1H), 7.50–7.44 (m, 1H), 7.32–7.26 (m, 2H), 2.95–2.90 (m, 2H), 1.94–1.84 (m, 2H), 1.42–1.36 (m, 2H), 0.93–0.89 (m, 3H); **^{13}C{^{1}H} NMR** (75 MHz, $CDCl_3$) δ 167.3, 150.7, 141.3, 124.3, 124.0, 119.5, 110.2, 31.3, 28.6, 26.4, 22.3, 13.9; Data are consistent with those reported in the literature.[5]

2-(*tert*-Butyl)benzo[*d*]oxazole (3l)

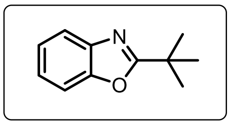

The general procedure was used employing **1n** (109.9 mg, 0.25 mmol). Flash column chromatography on silica gel using hexane/diethyl ether (20:1) provided pure **3l** (41.2 mg, 0.235 mmol, 94%) as a colorless oil. **^{1}H NMR** (300 MHz, $CDCl_3$) δ 7.73–7.67 (m, 1H), 7.52–7.46 (m, 1H), 7.32–7.26 (m, 2H), 1.50 (s, 9H); **^{13}C{^{1}H} NMR** (75 MHz, $CDCl_3$) δ 173.5, 150.8, 141.2, 124.4, 123.9, 119.7, 110.3, 34.1, 28.4; Data are consistent with those reported in the literature.[5]

4.2. Synthesis of 2,7-Di and 2,5,7-Trisubstituted Benzoxazoles (Scheme 1, Conditions B)

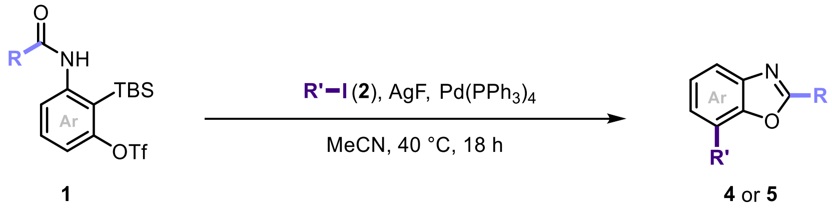

General Procedure for the Synthesis of 2,7-Di and 2,5,7-Trisubstituted Benzoxazoles:

A sealed tube was charged with **1** (0.39 mmol, 1.3 equiv.), (hetero)aryl or alkenyl iodide **2** (0.3 mmol, 1.0 equiv.), AgF (1.2 mmol, 4.0 equiv.) and $Pd(PPh_3)_4$ (0.03 mmol, 10 mol%). The sealed tube and contents were placed under vacuum and back-filled with argon under a Schlenk line three times. Degassed anhydrous MeCN (3.0 mL, 0.1 M) was added under argon, and the reaction mixture was stirred in an oil bath at 40 ºC for 18 h. After completion of the reaction as monitored by TLC analysis, the reaction mixture was cooled to room temperature, celite-filtered with diethyl ether (3 × 10 mL) and concentrated under reduced pressure. The crude product was purified by flash column chromatography on silica gel to obtain desired products **4** or **5**.

7-(4-Methoxyphenyl)-2-phenylbenzo[*d*]oxazole (4a)

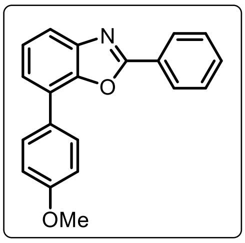

The general procedure was used employing **1a** (179.2 mg, 0.39 mmol), 4-iodoanisole **2a** (70.2 mg, 0.3 mmol). Flash column chromatography on silica gel using hexane/diethyl ether (20:1) provided pure **4a** (83.2 mg, 0.276 mmol, 92%) as a white solid. **m.p**: 144−146 °C; $\mathbf{R_f}$ 0.44 (hexane/diethyl ether = 3:1); **^{1}H NMR** (300 MHz, $CDCl_3$) δ 8.28–8.24 (m, 2H), 7.88–7.83 (m, 2H), 7.69 (dd, *J* = 7.8 and 1.3 Hz, 1H), 7.54–7.47 (m, 4H), 7.39 (t, *J* = 7.8 Hz, 1H), 7.10–7.05 (m, 2H), 3.88 (s, 3H); **^{13}C{^{1}H} NMR** (75 MHz, $CDCl_3$) δ 163.0, 159.5, 148.0, 142.7, 131.5, 129.3, 128.9, 128.0, 127.6, 127.1, 125.0, 124.7, 123.6, 118.3, 114.3, 55.3; **HRMS** (EI) *m/z* $[M]^+$ calcd for $C_{20}H_{15}NO_2$ 301.1103, found 301.1106.

Large-scale preparation of 7-(4-Methoxyphenyl)-2-phenylbenzo[*d*]oxazole (4a)

The general procedure was used employing **1a** (597.4 mg, 1.3 mmol), 4-iodoanisole **2a** (234.0 mg, 1.0 mmol). Flash column chromatography on silica gel using hexane/diethyl ether (20:1) provided pure **4a** (271.2 mg, 0.90 mmol, 90%) as a white solid.

7-(4-Methylphenyl)-2-phenylbenzo[*d*]oxazole (4b)

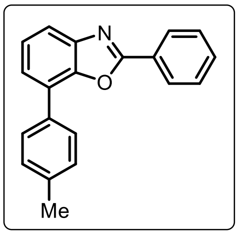

The general procedure was used employing **1a** (179.2 mg, 0.39 mmol), 4-iodotoluene **2b** (65.4 mg, 0.3 mmol). Flash column chromatography on silica gel using hexane/diethyl ether (30:1) provided pure **4b** (84.8 mg, 0.297 mmol, 99%) as a white solid. **m.p**: 100−102 °C; $\mathbf{R_f}$ 0.69 (hexane/diethyl ether = 2:1); **^{1}H NMR** (300 MHz, $CDCl_3$) δ 8.28–8.25 (m, 2H), 7.82–7.79 (m, 2H), 7.72 (dd, *J* = 7.8 and 1.2 Hz, 1H), 7.53–7.48 (m, 4H), 7.43–7.34 (m, 3H), 2.44 (s, 3H); **^{13}C{^{1}H} NMR** (75 MHz, $CDCl_3$) δ 163.0, 148.1, 142.7, 137.8, 132.6, 131.4, 129.5, 128.8, 128.0, 127.6, 127.0, 124.9, 123.9, 118.6, 21.2; **HRMS** (EI) *m/z* $[M]^+$ calcd for $C_{20}H_{15}NO$ 285.1154, found 285.1153.

7-(Phenyl)-2-phenylbenzo[*d*]oxazole (4c)

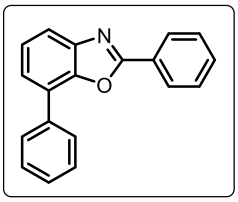

The general procedure was used employing **1a** (179.2 mg, 0.39 mmol), iodobenzene **2c** (0.034 mL, 0.3 mmol). Flash column chromatography on silica gel using hexane/diethyl ether (30:1) provided pure **4c** (79.0 mg, 0.291 mmol, 97%) as a white solid. **m.p**: 112−114 °C; **R*f*** 0.49 (hexane/diethyl ether = 3:1); **^{1}H NMR** (300 MHz, $CDCl_3$) δ 8.28–8.25 (m, 2H), 7.93–7.90 (m, 2H), 7.74 (dd, *J* = 7.8 and 1.2 Hz, 1H), 7.58–7.40 (m, 8H); **^{13}C{^{1}H} NMR** (75 MHz, $CDCl_3$) δ 163.1, 148.1, 142.7, 135.6, 131.5, 128.8, 128.8, 128.2, 128.0, 127.6, 127.0, 125.0, 125.0, 124.1, 118.9; **HRMS** (EI) *m/z* $[M]^+$ calcd for $C_{19}H_{13}NO$ 271.0997, found 271.0998.

7-(4-Bromophenyl)-2-phenylbenzo[*d*]oxazole (4d)

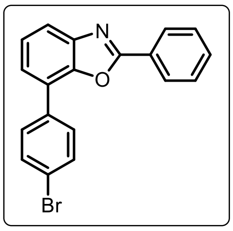

The general procedure was used employing **1a** (179.2 mg, 0.39 mmol), 1-bromo-4-iodobenzene **2d** (84.9 mg, 0.3 mmol). Flash column chromatography on silica gel using hexane/diethyl ether (30:1) provided pure **4d** (93.5 mg, 0.267 mmol, 89%) as a white solid. **m.p**: 132−134 °C; **R*f*** 0.21 (hexane/diethyl ether = 10:1); **^{1}H NMR** (300 MHz, $CDCl_3$) δ 8.25–8.20 (m, 2H), 7.77–7.72 (m, 3H), 7.67–7.62 (m, 2H), 7.54–7.45 (m, 4H), 7.39 (t, *J* = 7.7 Hz, 1H); **^{13}C{^{1}H} NMR** (75 MHz, $CDCl_3$) δ 163.1, 147.9, 142.8, 134.4, 131.9, 131.6, 129.6, 128.9, 127.6, 126.8, 125.1, 123.8, 123.7, 122.2, 119.4; **HRMS** (EI) *m/z* $[M]^+$ calcd for $C_{19}H_{12}BrNO$ 349.0102, found 349.0098.

Methyl 4-(2-phenylbenzo[*d*]oxazol-7-yl)benzoate (4e)

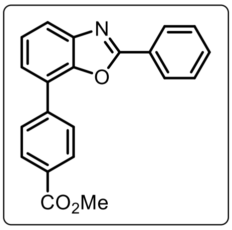

The general procedure was used employing **1a** (179.2 mg, 0.39 mmol), methyl 4-iodobenzoate **2e** (78.6 mg, 0.3 mmol). Flash column chromatography on silica gel using hexane/diethyl ether (15:1) provided pure **4e** (93.9 mg, 0.285 mmol, 95%) as a white solid. **m.p**: 144−146 °C; **R*f*** 0.26 (hexane/diethyl ether = 3:1); **^{1}H NMR** (300 MHz, $CDCl_3$) δ 8.26–8.19 (m, 4H), 8.00–7.96 (m, 2H), 7.77 (dd, *J* = 7.9 and 1.2 Hz, 1H), 7.57–7.50 (m, 4H), 7.43 (t, *J* = 7.8 Hz, 1H), 3.97 (s, 3H); **^{13}C{^{1}H} NMR** (75 MHz, $CDCl_3$) δ 166.7, 163.2, 148.1, 142.9, 140.0, 131.7, 130.0, 129.4, 128.9, 128.0, 127.6, 126.8, 125.1, 124.2, 123.7, 119.9, 52.2; **HRMS** (EI) *m/z* $[M]^+$ calcd for $C_{21}H_{15}NO_3$ 329.1052, found 329.1055.

2-Phenyl-7-[4-(trifluoromethyl)phenyl]benzo[*d*]oxazole (4f)

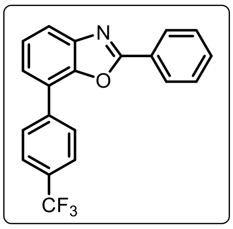

The general procedure was used employing **1a** (179.2 mg, 0.39 mmol), methyl 4-iodobenzotrifluoride **2f** (0.044 mL, 0.3 mmol). Flash column chromatography on silica gel using hexane/diethyl ether (30:1) provided pure **4f** (94.7 mg, 0.279 mmol, 93%) as a white solid. **m.p**: 154−156 °C; **R*f*** 0.48 (hexane/diethyl ether = 3:1); **^{1}H NMR** (300 MHz, $CDCl_3$) δ 8.26–8.19 (m, 2H), 7.98 (d, *J* = 8.0 Hz, 2H), 7.79–7.74 (m, 3H), 7.56–7.46 (m, 4H), 7.41 (t, *J* = 7.8 Hz, 1H); **^{13}C{^{1}H} NMR** (75 MHz, $CDCl_3$) δ 163.2, 148.1, 142.9, 139.1, 139.09, 131.7, 129.9 (q, *J* = 32.5 Hz), 128.9, 128.4, 127.6, 126.7, 125.7 (q, *J* = 3.8 Hz), 125.1, 124.2, 124.15 (q, *J* = 272.0 Hz), 123.4, 120.0; **HRMS** (EI) *m/z* $[M]^+$ calcd for $C_{20}H_{12}F_3NO$ 339.0871, found 339.0873.

3-(2-Phenylbenzo[*d*]oxazol-7-yl)phenol (4g)

The general procedure was used employing **1a** (179.2 mg, 0.39 mmol), 3-iodophenol **2g** (66.0 mg, 0.3 mmol). Flash column chromatography on silica gel using hexane/diethyl ether (5:1) provided pure **4g** (60.3 mg, 0.210 mmol, 70%) as a

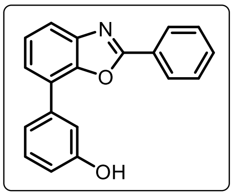

white solid. **m.p**: 178−180 °C; **R*f*** 0.13 (hexane/diethyl ether = 3:1); **^{1}H NMR** (300 MHz, CD_2Cl_2) δ 8.29–8.25 (m, 2H), 7.73 (dd, *J* = 7.9 and 1.2 Hz, 1H), 7.60–7.40 (m, 8H), 6.95 (ddd, *J* = 8.0, 2.5 and 1.1 Hz, 1H), 5.02 (brs, 1H); **^{13}C{^{1}H} NMR** (75 MHz, CD_2Cl_2) δ 163.7, 156.9, 148.7, 143.1, 137.6, 132.3, 130.7, 129.5, 128.2, 127.4, 125.7, 125.2, 124.8, 121.1, 119.5, 115.7, 115.6; **HRMS** (EI) *m/z* $[M]^+$ calcd for $C_{19}H_{13}NO_2$ 287.0946, found 287.0944.

7-(3-Methoxyphenyl)-2-phenylbenzo[*d*]oxazole (4h)

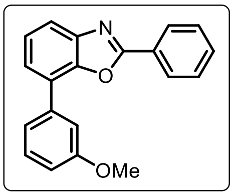

The general procedure was used employing **1a** (179.2 mg, 0.39 mmol), 3-iodoanisole **2h** (0.036 mL, 0.3 mmol). Flash column chromatography on silica gel using hexane/diethyl ether (20:1) provided pure **4h** (85.9 mg, 0.285 mmol, 95%) as a white solid. **m.p**: 56−58 °C; **R*f*** 0.37 (hexane/diethyl ether = 3:1); **^{1}H NMR** (300 MHz, $CDCl_3$) δ 8.29–8.23 (m, 2H), 7.74 (dd, *J* = 7.9 and 1.2 Hz, 1H), 7.55–7.39 (m, 8H), 7.00–6.96 (m, 1H), 3.90 (s, 3H); **^{13}C{^{1}H} NMR** (75 MHz, $CDCl_3$) δ 163.1, 159.9, 148.1, 142.8, 136.9, 131.5, 129.8, 128.9, 127.6, 127.0, 125.0, 124.9, 124.2, 120.6, 119.1, 113.9, 113.6, 55.3; **HRMS** (EI) *m/z* $[M]^+$ calcd for $C_{20}H_{15}NO_2$ 301.1103, found 301.1104.

7-(3-Fluorophenyl)-2-phenylbenzo[*d*]oxazole (4i)

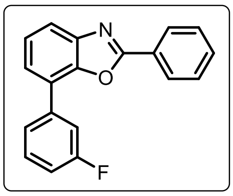

The general procedure was used employing **1a** (179.2 mg, 0.39 mmol), 3-fluoroiodobenzene **2i** (0.035 mL, 0.3 mmol). Flash column chromatography on silica gel using hexane/diethyl ether (30:1) provided pure **4i** (77.3 mg, 0.267 mmol, 89%) as a white solid. **m.p**: 94−96 °C; **R*f*** 0.46 (hexane/diethyl ether = 3:1); **^{1}H NMR** (300 MHz, $CDCl_3$) δ 8.27–8.23 (m, 2H), 7.76 (dd, *J* = 7.8 and 1.2 Hz, 1H), 7.71–7.61 (m, 2H), 7.55–7.46 (m, 5H), 7.42 (t, *J* = 7.8 Hz, 1H), 7.16–7.09 (m, 1H); **^{13}C{^{1}H} NMR** (75 MHz, $CDCl_3$) δ 164.7, 163.2, 161.5, 148.0, 142.9, 137.7 (d, *J* = 8.1 Hz), 131.7, 130.3 (d, *J* = 8.5 Hz), 128.9, 127.7, 126.9, 125.1, 124.1, 123.8 (d, *J* = 2.9 Hz), 119.6, 115.0 (d, *J* = 4.9 Hz); **HRMS** (EI) *m/z* $[M]^+$ calcd for $C_{19}H_{12}FNO$ 289.0903, found 289.0905.

7-(3-Chlorophenyl)-2-phenylbenzo[*d*]oxazole (4j)

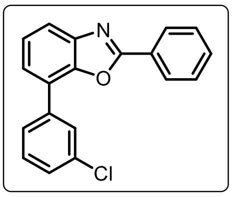

The general procedure was used employing **1a** (179.2 mg, 0.39 mmol), 3-chloroiodobenzene **2j** (0.037 mL, 0.3 mmol). Flash column chromatography on silica gel using hexane/diethyl ether (30:1) provided pure **4j** (89.0 mg, 0.291 mmol, 97%) as a white solid. **m.p**: 94−96 °C; **R*f*** 0.55 (hexane/diethyl ether = 3:1); **^{1}H NMR** (300 MHz, $CDCl_3$) δ 8.25–8.20 (m, 2H), 7.86 (t, *J* = 1.9 Hz, 1H), 7.77–7.71 (m, 2H), 7.52–7.35 (m, 7H); **^{13}C{^{1}H} NMR** (75 MHz, $CDCl_3$) δ 163.1, 147.9, 142.8, 137.3, 134.7, 131.6, 130.0, 128.9, 128.1, 128.0, 127.6, 126.8, 126.2, 125.0, 124.0, 123.4, 119.6; **HRMS** (EI) *m/z* $[M]^+$ calcd for $C_{19}H_{12}ClNO$ 305.0607, found 305.0603.

7-(2-Methoxyphenyl)-2-phenylbenzo[*d*]oxazole (4k)

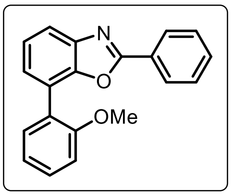

The general procedure was used employing **1a** (206.8 mg, 0.45 mmol), 2-iodoanisole **2k** (0.039 mL, 0.3 mmol). Flash column chromatography on silica gel using hexane/diethyl ether (20:1) provided pure **4k** (69.6 mg, 0.231 mmol, 77%) as a white solid. **m.p**: 100−102 °C; **R*f*** 0.48 (hexane/diethyl ether = 3:1); **^{1}H NMR** (300 MHz, $CDCl_3$) δ 8.23–8.20 (m, 2H), 7.76 (dd, *J* = 7.5 and 1.6 Hz, 1H), 7.55–7.38 (m, 7H), 7.14–7.07 (m, 2H), 3.85 (s, 3H); **^{13}C{^{1}H} NMR** (75 MHz,

$CDCl_3$) δ 162.9, 156.9, 148.8, 142.1, 131.4, 131.2, 129.6, 128.8, 127.5, 127.3, 126.5, 124.9, 124.4, 122.4, 120.7, 118.8, 111.3, 55.6; **HRMS** (EI) *m/z* $[M]^+$ calcd for $C_{20}H_{15}NO_2$ 301.1103, found 301.1104.

7-(3,5-Dimethylphenyl)-2-phenylbenzo[*d*]oxazole (4l)

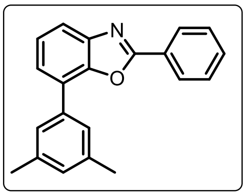

The general procedure was used employing **1a** (179.2 mg, 0.39 mmol), 1-iodo-3,5-dimethylbenzene **2l** (0.043 mL, 0.3 mmol). Flash column chromatography on silica gel using hexane/diethyl ether (30:1) provided pure **4l** (88.0 mg, 0.294 mmol, 98%) as a white solid. **m.p**: 86−88 °C; **R*f*** 0.70 (hexane/diethyl ether = 3:1); **^{1}H NMR** (300 MHz, $CDCl_3$) δ 8.28–8.24 (m, 2H), 7.73 (dd, *J* = 7.8 and 1.2 Hz, 1H), 7.55–7.50 (m, 6H), 7.41 (t, *J* = 7.7 Hz, 1H), 7.09 (s, 1H), 2.45 (s, 6H); **^{13}C{^{1}H} NMR** (75 MHz, $CDCl_3$) δ 163.1, 148.2, 142.7, 138.3, 135.6, 131.5, 129.7, 128.9, 127.6, 127.1, 126.1, 125.4, 125.0, 124.3, 118.7, 21.5; **HRMS** (EI) *m/z* $[M]^+$ calcd for $C_{21}H_{17}NO$ 299.1310, found 299.1311.

7-(Naphthalen-1-yl)-2-phenylbenzo[*d*]oxazole (4m)

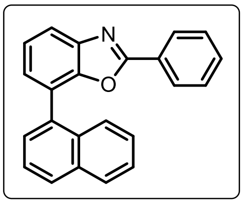

The general procedure was used employing **1a** (179.2 mg, 0.39 mmol), 1-iodonaphthalene **2m** (76.2 mg, 0.3 mmol). Flash column chromatography on silica gel using hexane/diethyl ether (30:1) provided pure **4m** (83.9 mg, 0.261 mmol, 87%) as a white solid. **m.p**: 164−166 °C; **R*f*** 0.58 (hexane/ethyl acetate = 3:1); **^{1}H NMR** (300 MHz, $CDCl_3$) δ 8.08 (dd, *J* = 8.0 and 1.7 Hz, 2H), 7.94 (d, *J* = 7.5 Hz, 2H), 7.85 (dd, *J* = 7.1 and 1.9 Hz, 1H), 7.77 (dd, *J* = 8.5 and 1.2 Hz, 1H), 7.63–7.34 (m, 9H); **^{13}C{^{1}H} NMR** (75 MHz, $CDCl_3$) δ 163.2, 149.0, 142.3, 133.8, 133.75, 131.5, 131.4, 128.7, 128.6, 128.3, 127.8, 127.6, 127.1, 126.9, 126.2, 126.0, 125.8, 125.4, 124.7, 124.3, 119.2; **HRMS** (EI) *m/z* $[M]^+$ calcd for $C_{23}H_{15}NO$ 321.1154, found 321.1157.

7-(6-Chloropyridin-3-yl)-2-phenylbenzo[*d*]oxazole (4n)

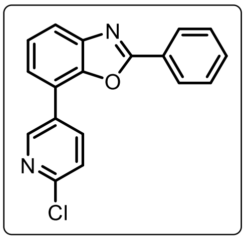

The general procedure was used employing **1a** (179.2 mg, 0.39 mmol), 2-chloro-5-iodopyridine **2n** (71.8 mg, 0.3 mmol). Flash column chromatography on silica gel using hexane/diethyl ether (10:1) provided pure **4n** (82.8 mg, 0.270 mmol, 90%) as a white solid. **m.p**: 156−158 °C; **R*f*** 0.24 (hexane/diethyl ether = 3:1); **^{1}H NMR** (300 MHz, $CDCl_3$) δ 9.00 (dd, *J* = 2.6 and 0.7 Hz, 1H), 8.27–8.24 (m, 2H), 8.17 (dd, *J* = 8.3 and 2.6 Hz, 1H), 7.81 (dd, *J* = 7.6 and 1.4 Hz, 1H), 7.58–7.44 (m, 6H); **^{13}C{^{1}H} NMR** (75 MHz, $CDCl_3$) δ 163.4, 150.9, 149.0, 148.0, 143.0, 137.8, 131.9, 130.4, 129.0, 127.7, 126.6, 125.4, 124.5, 123.7, 120.4, 120.2; **HRMS** (EI) *m/z* $[M]^+$ calcd for $C_{18}H_{11}ClN_2O$ 306.0560, found 306.0560.

2-Phenyl-7-(thiophen-2-yl)benzo[*d*]oxazole (4o)

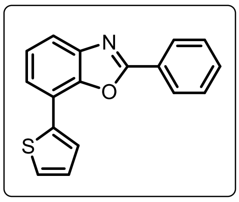

The general procedure was used employing **1a** (179.2 mg, 0.39 mmol), 2-iodothiophene **2o** (63.0 mg, 0.3 mmol). Flash column chromatography on silica gel using hexane/diethyl ether (30:1) provided pure **4o** (47.4 mg, 0.171 mmol, 57%) as a white solid. **m.p**: 140−142 °C; **R*f*** 0.55 (hexane/diethyl ether = 3:1); **^{1}H NMR** (300 MHz, $CDCl_3$) δ 8.32–8.29 (m, 2H), 7.81 (dd, *J* = 3.7 and 1.2 Hz, 1H), 7.67 (dd, *J* = 7.9 and 1.1 Hz, 1H), 7.60 (dd, *J* = 7.8 and 1.1 Hz, 1H), 7.57–7.52 (m, 3H), 7.42 (dd, *J* = 5.1 and 1.2 Hz, 1H), 7.35 (t, *J* = 7.8 Hz, 1H), 7.21 (dd, *J* = 5.1 and 3.6 Hz, 1H); **^{13}C{^{1}H} NMR** (75 MHz, $CDCl_3$) δ 163.2, 146.8, 142.7, 137.9, 131.7, 129.0, 128.0, 127.7, 126.9, 126.1, 125.7, 125.0, 122.5, 118.8, 118.7;

HRMS (EI) *m/z* $[M]^+$ calcd for $C_{17}H_{11}NOS$ 277.0561, found 277.0559.

5-(2-Phenylbenzo[*d*]oxazol-7-yl)furan-2-carbaldehyde (4p)

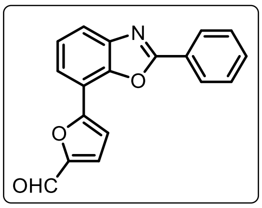

The general procedure was used employing **1a** (179.2 mg, 0.39 mmol), 5-iodo-2-furaldehyde **2p** (66.6 mg, 0.3 mmol). Flash column chromatography on silica gel using hexane/ethyl acetate (15:1) provided pure **4p** (51.3 mg, 0.177 mmol, 59%) as a white solid. **m.p**: 152−154 °C; $\mathbf{R_f}$ 0.23 (hexane/diethyl ether = 3:1); **^{1}H NMR** (300 MHz, $CDCl_3$) δ 9.74 (s, 1H), 8.35–8.22 (m, 2H), 7.94 (dd, *J* = 7.8 and 1.1 Hz, 1H), 7.79 (dd, *J* = 8.0 and 1.1 Hz, 1H), 7.57 (dd, *J* = 5.3 and 2.0 Hz, 3H), 7.50–7.39 (m, 2H), 7.34 (d, *J* = 3.7 Hz, 1H); **^{13}C{^{1}H} NMR** (75 MHz, $CDCl_3$) δ 177.4, 163.3, 154.0, 151.9, 146.9, 142.7, 132.0, 129.1, 127.7, 126.6, 125.1, 121.6, 121.1, 113.6, 111.8; **HRMS** (EI) *m/z* $[M]^+$ calcd for $C_{18}H_{11}NO_3$ 289.0739, found 289.0742.

2-Phenyl-7-(9-phenyl-9*H*-carbazol-3-yl)benzo[*d*]oxazole (4q)

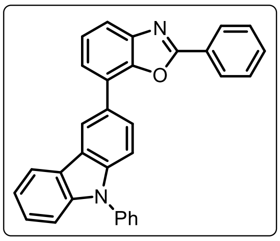

The general procedure was used employing **1a** (179.2 mg, 0.39 mmol), 3-iodo-9-phenylcarbazole **2q** (110.8 mg, 0.3 mmol). Flash column chromatography on silica gel using hexane/diethyl ether (15:1) provided pure **4q** (107.4 mg, 0.246 mmol, 82%) as a white solid. **m.p**: 88−90 °C; $\mathbf{R_f}$ 0.33 (hexane/diethyl ether = 3:1); **^{1}H NMR** (300 MHz, $CDCl_3$) δ 8.63 (d, *J* = 2.0 Hz, 1H), 8.29–8.25 (m, 2H), 8.20 (dt, *J* = 7.7 and 1.1 Hz, 1H), 7.93 (dd, *J* = 8.6 and 1.8 Hz, 1H), 7.71 (dd, *J* = 7.9 and 1.1 Hz, 1H), 7.62–7.39 (m, 13H), 7.33–7.28 (m, 1H); **^{13}C{^{1}H} NMR** (75 MHz, $CDCl_3$) δ 163.0, 148.2, 142.7, 141.3, 140.5, 137.4, 131.4, 129.9, 128.8, 127.5, 127.53, 127.46, 127.1, 127.0, 126.3, 126.2, 125.8, 125.1, 124.2, 123.8, 123.4, 120.3, 120.2, 120.0, 118.1, 110.0, 109.9; **HRMS** (EI) *m/z* $[M]^+$ calcd for $C_{31}H_{20}N_2O$ 436.1576, found 436.1573.

7-(Cyclohexylidenemethyl)-2-phenylbenzo[*d*]oxazole (4r)

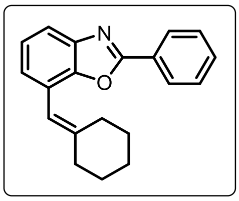

The general procedure was used employing **1a** (179.2 mg, 0.39 mmol), (iodomethylene)cyclohexane **2r** (66.6 mg, 0.3 mmol). Flash column chromatography on silica gel using hexane/diethyl ether (50:1) provided pure **4r** (61.7 mg, 0.213 mmol, 71%) as a white solid. **m.p**: 60−62 °C; $\mathbf{R_f}$ 0.58 (hexane/diethyl ether = 3:1); **^{1}H NMR** (300 MHz, $CDCl_3$) δ 8.30–8.24 (m, 2H), 7.62 (dd, *J* = 7.9 and 1.2 Hz, 1H), 7.55–7.50 (m, 3H), 7.29 (t, *J* = 7.7 Hz, 1H), 7.18 (dt, *J* = 7.6 and 1.0 Hz, 1H), 6.40 (s, 1H), 2.42–2.36 (m, 4H), 1.76–1.61 (m, 6H); **^{13}C{^{1}H} NMR** (75 MHz, $CDCl_3$) δ 162.7, 148.6, 146.7, 142.0, 131.4, 128.8, 127.6, 127.3, 125.7, 124.3, 122.4, 117.7, 114.9, 37.6, 30.4, 28.6, 27.8, 26.5; **HRMS** (EI) *m/z* $[M]^+$ calcd for $C_{20}H_{19}NO$ 289.1467, found 289.1467.

2-(2-Phenylbenzo[*d*]oxazol-7-yl)cyclohex-2-en-1-one (4s)

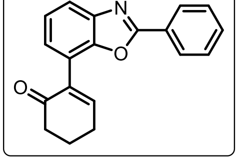

The general procedure was used employing **1a** (179.2 mg, 0.39 mmol), 2-iodocyclohex-2-en-1-one **2s** (66.6 mg, 0.3 mmol). Flash column chromatography on silica gel using hexane/diethyl ether (5:1) provided pure **4s** (54.7 mg, 0.189 mmol, 63%) as a white solid. **m.p**: 148−150 °C; $\mathbf{R_f}$ 0.15 (hexane/diethyl ether = 3:1); **^{1}H NMR** (300 MHz, $CDCl_3$) δ 8.22–8.18 (m, 2H), 7.71 (dd, *J* = 7.7 and 1.4 Hz, 1H), 7.53–7.48 (m, 3H), 7.36–7.26 (m, 3H), 2.73–2.63 (m, 4H), 2.23–2.19 (m, 2H); **^{13}C{^{1}H} NMR** (75 MHz, $CDCl_3$) δ 196.7, 162.8, 150.1, 148.6, 142.1, 135.6, 131.4, 128.8, 127.6, 127.1, 125.9, 124.3, 120.5, 119.3, 38.8, 26.6,

22.8; **HRMS** (EI) *m/z* $[M]^+$ calcd for $C_{19}H_{15}NO_2$ 289.1103, found 289.1105.

4-(2-Phenylbenzo[*d*]oxazol-7-yl)benzyl 2-(4-isobutylphenyl)propanoate (4t)

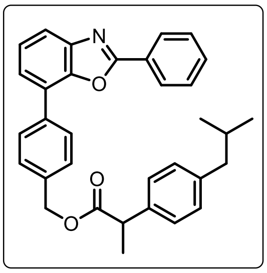

The general procedure was used employing **1a** (179.2 mg, 0.39 mmol), 4-iodobenzyl 2-(4-isobutylphenyl)propanoate **2t** (126.7 mg, 0.3 mmol). Flash column chromatography on silica gel using hexane/diethyl ether (20:1) provided pure **4t** (129.3 mg, 0.264 mmol, 88%) as a white solid. **m.p**: 76–78 °C; $\mathbf{R}_f$ 0.45 (hexane/diethyl ether = 3:1); ^{1}**H NMR** (300 MHz, $CDCl_3$) δ 8.27–8.24 (m, 2H), 7.85 (d, *J* = 8.3 Hz, 2H), 7.74 (dd, *J* = 7.8 and 1.3 Hz, 1H), 7.54–7.49 (m, 4H), 7.44–7.38 (m, 3H), 7.26–7.23 (m, 2H), 7.12–7.09 (m, 2H), 5.20 (d, *J* = 2.5 Hz, 2H), 3.80 (q, *J* = 7.1 Hz, 1H), 2.44 (d, *J* = 7.2 Hz, 2H), 1.83 (sept, *J* = 6.7 Hz, 1H), 1.55 (d, *J* = 7.2 Hz, 3H), 0.88 (d, *J* = 6.6 Hz, 6H); ^{13}**C{^{1}H} NMR** (75 MHz, $CDCl_3$) δ 174.5, 163.1, 148.1, 142.8, 140.6, 137.5, 135.9, 135.3, 131.6, 129.3, 128.9, 128.2, 127.6, 127.2, 127.0, 125.0, 124.5, 124.1, 119.1, 66.0, 45.1, 45.0, 30.2, 22.3, 18.4; **HRMS** (EI) *m/z* $[M]^+$ calcd for $C_{33}H_{31}NO_3$ 489.2304, found 489.2308.

Methyl 5-[2,5-dimethyl-4-(2-phenylbenzo[*d*]oxazol-7-yl)phenoxy]-2,2-dimethylpentanoate (4u)

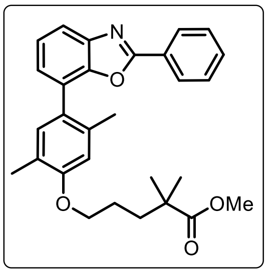

The general procedure was used employing **1a** (179.2 mg, 0.39 mmol), methyl 5-(4-iodo-2,5-dimethylphenoxy)-2,2-dimethylpentanoate **2u** (117.1 mg, 0.3 mmol). Flash column chromatography on silica gel using hexane/diethyl ether (10:1) provided pure **4u** (120.8 mg, 0.264 mmol, 88%) as a colorless oil. $\mathbf{R}_f$ 0.33 (hexane/diethyl ether = 3:1); ^{1}**H NMR** (300 MHz, $CDCl_3$) δ 8.23–8.19 (m, 2H), 7.74 (dd, *J* = 7.9 and 1.2 Hz, 1H), 7.51–7.48 (m, 3H), 7.39 (t, *J* = 7.7 Hz, 1H), 7.27–7.24 (m, 1H), 7.18 (s, 1H), 6.79 (s, 1H), 4.03 (t, *J* = 5.3 Hz, 2H), 3.69 (s, 3H), 2.26 (s, 6H), 1.82–1.74 (m, 4H), 1.25 (s, 6H); ^{13}**C{^{1}H} NMR** (75 MHz, $CDCl_3$) δ 178.3, 163.0, 156.9, 148.6, 141.9, 134.9, 132.4, 131.4, 128.8, 127.6, 127.3, 127.2, 126.7, 125.6, 124.6, 124.2, 118.4, 112.7, 68.0, 51.7, 42.1, 37.1, 25.2, 20.4, 15.7; **HRMS** (EI) *m/z* $[M]^+$ calcd for $C_{29}H_{31}NO_4$ 457.2253, found 457.2250.

(1*S*,2*R*,5*R*)-2-Isopropyl-5-methylcyclohexyl 4-(2-phenylbenzo[*d*]oxazol-7-yl)benzoate (4v)

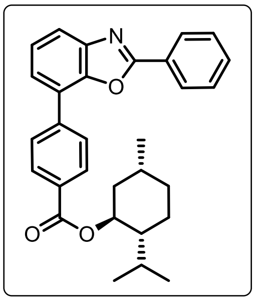

The general procedure was used employing **1a** (179.2 mg, 0.39 mmol), (*1S,2R,5R*)-2-isopropyl-5-methylcyclohexyl 4-iodobenzoate **2v** (115.9 mg, 0.3 mmol). Flash column chromatography on silica gel using hexane/diethyl ether (20:1) provided pure **4v** (80.4 mg, 0.177 mmol, 59%) as a white solid. **m.p**: 140−142 °C; $\mathbf{R}_f$ 0.53 (hexane/diethyl ether = 3:1); $[\alpha]_D^{25}$ +3.4° (c 0.15, CH_2Cl_2); ^{1}**H NMR** (300 MHz, $CDCl_3$) δ 8.28–8.21 (m, 4H), 7.99 (d, *J* = 8.4 Hz, 2H), 7.78 (dd, *J* = 7.9 and 1.2 Hz, 1H), 7.59–7.51 (m, 4H), 7.45 (t, *J* = 7.8 Hz, 1H), 5.00 (td, *J* = 10.8 and 4.4 Hz, 1H), 2.22–2.14 (m, 1H), 2.08–1.96 (m, 1H), 1.80–1.72 (m, 2H), 1.64–1.57 (m, 2H), 1.25–1.09 (m, 3H), 0.96 (d, *J* = 7.3 Hz, 6H), 0.84 (d, *J* = 6.9 Hz, 3H); ^{13}**C{^{1}H} NMR** (75 MHz, $CDCl_3$) δ 165.8, 163.2, 148.2, 142.9, 139.9, 131.7, 130.2, 130.1, 128.9, 128.0, 127.6, 126.8, 125.1, 124.3, 123.9, 119.8, 74.9, 47.3, 41.0, 34.3, 31.4, 26.5, 23.6, 22.0, 20.8, 16.5; **HRMS** (EI) *m/z* $[M]^+$ calcd for $C_{30}H_{31}NO_3$ 453.2304, found 453.2303.

2-(2-Methoxyphenyl)-7-(4-methoxyphenyl)benzo[*d*]oxazole (5a)

The general procedure was used employing **1c** (220.3 mg, 0.45 mmol), 4-iodoanisole **2a** (70.2 mg, 0.3 mmol). Flash column chromatography on silica gel using hexane/diethyl ether (10:1) provided pure **5a** (71.6 mg, 0.216 mmol, 72%) as

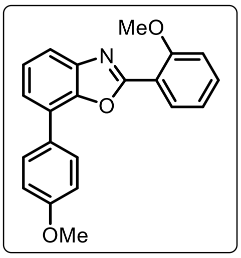

a white solid. **m.p**: 104−106 °C; **R*f*** 0.29 (hexane/diethyl ether = 2:1); **^{1}H NMR** (300 MHz, $CDCl_3$) δ 8.19–8.15 (m, 1H), 7.93–7.91 (m, 2H), 7.75 (dd, *J* = 7.8 and 1.2 Hz, 1H), 7.53–7.47 (m, 2H), 7.39 (t, *J* = 7.8 Hz, 1H), 7.13–7.05 (m, 4H), 4.03 (s, 3H), 3.88 (s, 3H); **^{13}C{^{1}H} NMR** (75 MHz, $CDCl_3$) δ 161.5, 159.4, 158.5, 147.5, 142.7, 132.7, 131.1, 129.2, 128.1, 124.7, 124.3, 123.1, 120.6, 118.4, 116.0, 114.1, 112.0, 56.1, 55.3; **HRMS** (EI) *m/z* $[M]^+$ calcd for $C_{21}H_{17}NO_3$ 331.1208, found 331.1206.

2-(3-Methoxyphenyl)-7-(4-methoxyphenyl)benzo[*d*]oxazole (5b)

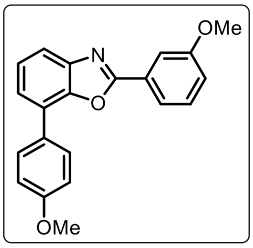

The general procedure was used employing **1d** (190.9 mg, 0.39 mmol), 4-iodoanisole **2a** (70.2 mg, 0.3 mmol). Flash column chromatography on silica gel using hexane/diethyl ether (10:1) provided pure **5b** (83.5 mg, 0.252 mmol, 84%) as a white solid. **m.p**: 122−124 °C; **R*f*** 0.32 (hexane/diethyl ether = 3:1); **^{1}H NMR** (300 MHz, $CDCl_3$) δ 7.86–7.77 (m, 4H), 7.68 (dd, *J* = 7.8 and 1.2 Hz, 1H), 7.48–7.35 (m, 3H), 7.08–7.03 (m, 3H), 3.88 (s, 3H), 3.87 (s, 3H); **^{13}C{^{1}H} NMR** (75 MHz, $CDCl_3$) δ 162.8, 159.8, 159.5, 147.9, 142.6, 129.9, 129.2, 128.2, 127.9, 125.0, 124.6, 123.6, 120.0, 118.2, 117.8, 114.2, 112.2, 55.4, 55.3; **HRMS** (EI) *m/z* $[M]^+$ calcd for $C_{21}H_{17}NO_3$ 331.1208, found 331.1212.

2,7-Bis(4-methoxyphenyl)benzo[*d*]oxazole (5c)

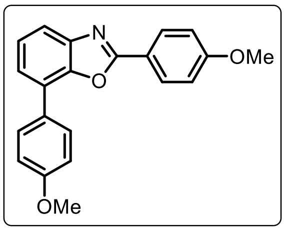

The general procedure was used employing **1e** (190.9 mg, 0.39 mmol), 4-iodoanisole **2a** (70.2 mg, 0.3 mmol). Flash column chromatography on silica gel using hexane/diethyl ether (10:1) provided pure **5c** (76.6 mg, 0.231 mmol, 77%) as a white solid. **m.p**: 128−130 °C; **R*f*** 0.28 (hexane/diethyl ether = 3:1); **^{1}H NMR** (300 MHz, $CDCl_3$) δ 8.21–8.16 (m, 2H), 7.87–7.82 (m, 2H), 7.65 (dd, *J* = 7.7 and 1.3 Hz, 1H), 7.45–7.33 (m, 2H), 7.08–7.03 (m, 2H), 7.02–6.97 (m, 2H), 3.87 (s, 3H), 3.85 (s, 3H); **^{13}C{^{1}H} NMR** (75 MHz, $CDCl_3$) δ 163.1, 162.2, 159.4, 147.9, 142.9, 129.3, 129.2, 128.1, 124.8, 124.4, 123.1, 119.6, 117.9, 114.2, 114.19, 55.3, 55.27; **HRMS** (EI) *m/z* $[M]^+$ calcd for $C_{21}H_{17}NO_3$ 331.1208, found 331.1212.

2-(4-Fluorophenyl)-7-(4-methoxyphenyl)benzo[*d*]oxazole (5d)

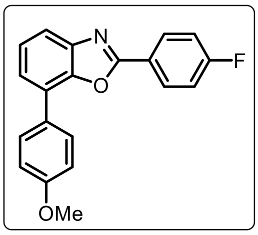

The general procedure was used employing **1g** (186.2 mg, 0.39 mmol), 4-iodoanisole **2a** (70.2 mg, 0.3 mmol). Flash column chromatography on silica gel using hexane/diethyl ether (30:1) provided pure **5d** (82.4 mg, 0.258 mmol, 86%) as a white solid. **m.p**: 150−152 °C; **R*f*** 0.50 (hexane/diethyl ether = 3:1); **^{1}H NMR** (300 MHz, $CDCl_3$) δ 8.27–8.22 (m, 2H), 7.86–7.81 (m, 2H), 7.67 (dd, *J* = 7.8 and 1.3 Hz, 1H), 7.49–7.36 (m, 2H), 7.25–7.17 (m, 2H), 7.10–7.05 (m, 2H), 3.89 (s, 3H); **^{13}C{^{1}H} NMR** (75 MHz, $CDCl_3$) δ 164.8 (d, *J* = 252.7 Hz), 162.1, 159.5, 148.0, 142.6, 129.8 (d, *J* = 8.9 Hz), 129.3, 127.9, 125.1, 124.7, 123.4 (d, *J* = 3.2 Hz), 123.7, 118.2, 116.1 (d, *J* = 22.1 Hz), 114.3, 55.3; **HRMS** (EI) *m/z* $[M]^+$ calcd for $C_{20}H_{14}FNO_2$ 319.1009, found 319.1012.

2-(4-Chlorophenyl)-7-(4-methoxyphenyl)benzo[*d*]oxazole (5e)

The general procedure was used employing **1h** (192.7 mg, 0.39 mmol), 4-iodoanisole **2a** (70.2 mg, 0.3 mmol). Flash column chromatography on silica gel using hexane/diethyl ether (30:1) provided pure **5e** (84.6 mg, 0.252 mmol, 84%) as

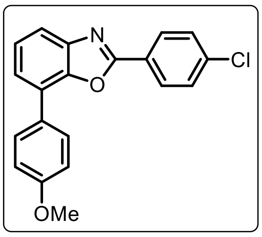

a white solid. **m.p**: 174−176 °C; **R$_f$** 0.56 (hexane/diethyl ether = 3:1); **^{1}H NMR** (300 MHz, $CDCl_3$) δ 8.18–8.13 (m, 2H), 7.84–7.79 (m, 2H), 7.66 (dd, *J* = 7.8 and 1.3 Hz, 1H), 7.48–7.44 (m, 3H), 7.37 (t, *J* = 7.7 Hz, 1H), 7.08–7.03 (m, 2H), 3.88 (s, 3H); **^{13}C{^{1}H} NMR** (75 MHz, $CDCl_3$) δ 161.9, 159.5, 148.0, 142.6, 137.7, 129.2, 129.17, 128.8, 127.8, 125.5, 125.1, 124.7, 123.8, 118.3, 114.2, 55.3; **HRMS** (EI) *m/z* [M]$^+$ calcd for $C_{20}H_{14}ClNO_2$ 335.0713, found 335.0711.

2-(2-Bromophenyl)-7-(4-methoxyphenyl)benzo[*d*]oxazole (5f)

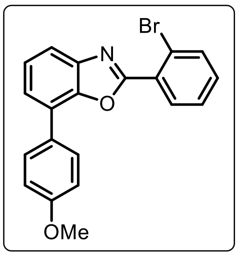

The general procedure was used employing **1f** (210.0 mg, 0.39 mmol), 4-iodoanisole **2a** (70.2 mg, 0.3 mmol). Flash column chromatography on silica gel using hexane/diethyl ether (20:1) provided pure **5f** (102.7 mg, 0.270 mmol, 90%) as a white solid. **m.p**: 108−110 °C; **R$_f$** 0.19 (hexane/diethyl ether = 10:1); **^{1}H NMR** (300 MHz, $CDCl_3$) δ 8.10 (dd, *J* = 7.7 and 1.8 Hz, 1H), 7.93–7.88 (m, 2H), 7.75 (dt, *J* = 8.0 and 1.4 Hz, 2H), 7.54 (dd, *J* = 7.7 and 1.2 Hz, 1H), 7.46–7.39 (m, 2H), 7.33 (td, *J* = 7.8 and 1.8 Hz, 1H), 7.06–7.01 (m, 2H), 3.85 (s, 3H); **^{13}C{^{1}H} NMR** (75 MHz, $CDCl_3$) δ 161.6, 159.5, 148.0, 142.2, 134.6, 132.2, 132.0, 129.3, 128.3, 127.6, 127.4, 125.1, 124.8, 123.8, 121.7, 118.6, 114.2, 55.3; **HRMS** (EI) *m/z* [M]$^+$ calcd for $C_{20}H_{14}BrNO_2$ 379.0208, found 379.0205.

7-(4-Methoxyphenyl)-2-(naphthalen-1-yl)benzo[*d*]oxazole (5g)

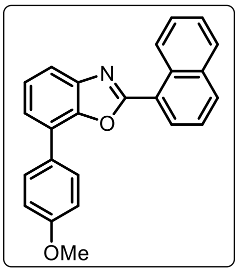

The general procedure was used employing **1i** (198.8 mg, 0.39 mmol), 4-iodoanisole **2a** (70.2 mg, 0.3 mmol). Flash column chromatography on silica gel using hexane/diethyl ether (30:1) provided pure **5g** (87.5 mg, 0.249 mmol, 83%) as a white solid. **m.p**: 148−150 °C; **R$_f$** 0.45 (hexane/diethyl ether = 3:1); **^{1}H NMR** (300 MHz, $CDCl_3$) δ 9.50 (dd, *J* = 8.7 and 1.2 Hz, 1H), 8.42 (dd, *J* = 7.4 and 1.3 Hz, 1H), 8.01 (d, *J* = 8.2 Hz, 1H), 7.93–7.87 (m, 3H), 7.80 (dd, *J* = 7.8 and 1.2 Hz, 1H), 7.70 (ddd, *J* = 8.5, 6.8 and 1.5 Hz, 1H), 7.61–7.52 (m, 3H), 7.43 (t, *J* = 7.8 Hz, 1H), 7.10–7.05 (m, 2H), 3.88 (s, 3H); **^{13}C{^{1}H} NMR** (75 MHz, $CDCl_3$) δ 162.8, 159.5, 147.4, 143.0, 133.9, 132.3, 130.7, 129.3, 128.6, 128.1, 127.9, 126.4, 126.3, 124.9, 124.89, 124.6, 123.7, 123.5, 118.5, 114.3, 55.3; **HRMS** (EI) *m/z* [M]$^+$ calcd for $C_{24}H_{17}NO_2$ 351.1259, found 351.1257.

2-(Furan-2-yl)-7-(4-methoxyphenyl)benzo[*d*]oxazole (5h)

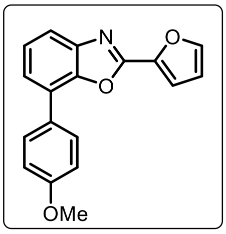

The general procedure was used employing **1j** (175.3 mg, 0.39 mmol), 4-iodoanisole **2a** (70.2 mg, 0.3 mmol). Flash column chromatography on silica gel using hexane/diethyl ether (15:1) provided pure **5h** (69.9 mg, 0.240 mmol, 80%) as a white solid. **m.p**: 174−176 °C; **R$_f$** 0.24 (hexane/diethyl ether = 3:1); **^{1}H NMR** (300 MHz, $CDCl_3$) δ 7.85–7.81 (m, 2H), 7.70–7.67 (m, 2H), 7.49 (dd, *J* = 7.8 and 1.3 Hz, 1H), 7.41 (t, *J* = 7.7 Hz, 1H), 7.29 (dd, *J* = 3.5 and 0.8 Hz, 1H), 7.09–7.05 (m, 2H), 6.61 (dd, *J* = 3.5 and 1.8 Hz, 1H), 3.89 (s, 3H); **^{13}C{^{1}H} NMR** (75 MHz, $CDCl_3$) δ 159.5, 155.3, 147.4, 145.7, 142.6, 142.2, 129.3, 127.8, 125.3, 124.7, 123.8, 118.4, 114.3, 112.2, 55.3; **HRMS** (EI) *m/z* [M]$^+$ calcd for $C_{18}H_{13}NO_3$ 291.0895, found 291.0894.

7-(4-Methoxyphenyl)-2-methylbenzo[d]oxazole (5i)

The general procedure was used employing **1m** (179.0 mg, 0.45 mmol), 4-iodoanisole **2a** (70.2 mg, 0.3 mmol). Flash

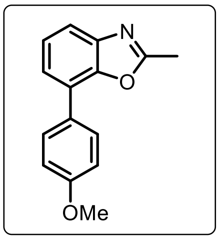

column chromatography on silica gel using hexane/diethyl ether (30:1) provided pure **5i** (54.6 mg, 0.228 mmol, 76%) as a white solid. **m.p**: 70−73 °C; **R$_f$** 0.23 (hexane/diethyl ether = 3:1); **^{1}H NMR** (300 MHz, $CDCl_3$) δ 7.88–7.76 (m, 2H), 7.62 (dd, *J* = 7.7 and 1.3 Hz, 1H), 7.47 (dd, *J* = 7.7, 1.2 Hz, 1H), 7.38 (t, *J* = 7.7 Hz, 1H), 7.13–7.02 (m, 2H), 3.90 (s, 3H), 2.71 (s, 3H); **^{13}C{^{1}H} NMR** (75 MHz, $CDCl_3$) δ 159.6, 129.4, 128.0, 124.7, 124.5, 123.3, 117.7, 114.3, 55.4, 14.7; **HRMS** (EI) *m/z* $[M]^+$ calcd for $C_{15}H_{13}NO_2$ 239.0946, found 239.0944.

2-(*tert*-Butyl)-7-(4-methoxyphenyl)benzo[*d*]oxazole (5j)

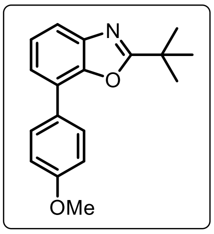

The general procedure was used employing **1n** (197.8 mg, 0.45 mmol), 4-iodoanisole **2a** (70.2 mg, 0.3 mmol). Flash column chromatography on silica gel using hexane/diethyl ether (30:1) provided pure **5j** (52.3 mg, 0.186 mmol, 62%) as a white solid. **m.p**: 112−114 °C; **R$_f$** 0.55 (hexane/diethyl ether = 3:1); **^{1}H NMR** (300 MHz, $CDCl_3$) δ 7.83–7.80 (m, 2H), 7.63 (dd, *J* = 7.8 and 1.2 Hz, 1H), 7.46 (dd, *J* = 7.8 and 1.3 Hz, 1H), 7.35 (t, *J* = 7.8 Hz, 1H), 7.07–7.04 (m, 2H), 3.89 (s, 3H), 1.53 (s, 9H); **^{13}C{^{1}H} NMR** (75 MHz, $CDCl_3$) δ 173.5, 159.4, 148.0, 141.9, 129.2, 128.1, 124.4, 124.3, 122.9, 118.0, 114.2, 55.3, 34.1, 28.5; **HRMS** (EI) *m/z* $[M]^+$ calcd for $C_{18}H_{19}NO_2$ 281.1416, found 281.1415.

5,7-Bis(4-methoxyphenyl)-2-phenylbenzo[*d*]oxazole (5k)

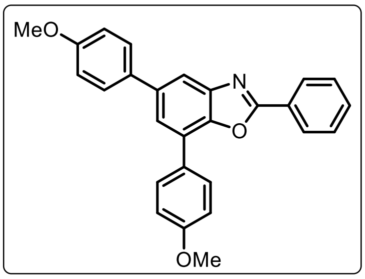

The general procedure was used employing **1o** (220.6 mg, 0.39 mmol), 4-iodoanisole **2a** (70.2 mg, 0.3 mmol). Flash column chromatography on silica gel using hexane/ethyl acetate (20:1) provided pure **5k** (95.3 mg, 0.234 mmol, 78%) as a white solid. **m.p**: 130−132 °C; **R$_f$** 0.46 (hexane/ethyl acetate = 3:1); **^{1}H NMR** (300 MHz, $CDCl_3$) δ 8.30–8.26 (m, 2H), 7.92–7.89 (m, 2H), 7.84 (d, *J* = 1.8 Hz, 1H), 7.67 (d, *J* = 1.7 Hz, 1H), 7.62–7.59 (m, 2H), 7.55–7.53 (m, 3H), 7.11–7.08 (m, 2H), 7.03–7.00 (m, 2H), 3.90 (s, 3H), 3.87 (s, 3H); **^{13}C{^{1}H} NMR** (75 MHz, $CDCl_3$) δ 163.5, 159.6, 159.2, 147.3, 143.4, 138.5, 133.7, 131.6, 129.4, 128.9, 128.5, 128.0, 127.6, 127.1, 124.6, 123.0, 116.3, 114.3, 114.29, 55.4; **HRMS** (EI) *m/z* $[M]^+$ calcd for $C_{27}H_{21}NO_3$ 407.1521, found 407.1518.

7-(4-Methoxyphenyl)-2-phenyl-5-(*p*-tolyl)benzo[*d*]oxazole (5l)

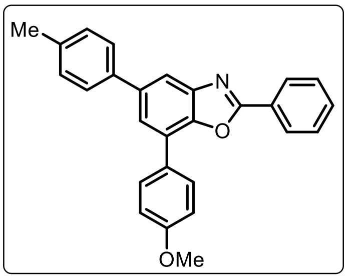

The general procedure was used employing **1p** (214.4 mg, 0.39 mmol), 4-iodoanisole **2a** (70.2 mg, 0.3 mmol). Flash column chromatography on silica gel using hexane/diethyl ether (20:1) provided pure **5l** (68.1 mg, 0.174 mmol, 58%) as a white solid. **m.p**: 130−132 °C; **R$_f$** 0.43 (hexane/diethyl ether = 3:1); **^{1}H NMR** (300 MHz, $CDCl_3$) δ 8.30–8.27 (m, 2H), 7.93–7.87 (m, 3H), 7.70 (d, *J* = 1.7 Hz, 1H), 7.59–7.51 (m, 5H), 7.29 (d, *J* = 7.8 Hz, 2H), 7.12–7.07 (m, 2H), 3.91 (s, 3H), 2.42 (s, 3H); **^{13}C{^{1}H} NMR** (75 MHz, $CDCl_3$) δ 163.5, 159.6, 147.5, 143.3, 138.8, 138.3, 137.1, 131.6, 129.6, 129.4, 128.9, 128.0, 127.6, 127.3, 127.1, 124.6, 123.2, 116.6, 114.3, 55.4, 21.1; **HRMS** (EI) *m/z* $[M]^+$ calcd for $C_{27}H_{21}NO_2$ 391.1572, found 391.1574.

5-(4-Chlorophenyl)-7-(4-methoxyphenyl)-2-phenylbenzo[*d*]oxazole (5m)

The general procedure was used employing **1q** (222.3 mg, 0.39 mmol), 4-iodoanisole **2a** (70.2 mg, 0.3 mmol). Flash column chromatography on silica gel using hexane/diethyl ether (30:1) provided pure **5m** (79.1 mg, 0.192 mmol, 64%) as

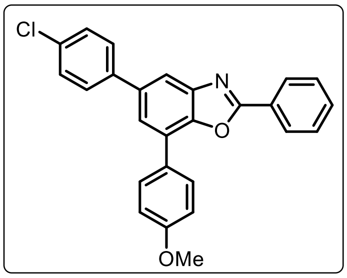

a white solid. **m.p**: 164−166 °C; **R*f*** 0.54 (hexane/diethyl ether = 3:1); **^{1}H NMR** (300 MHz, $CDCl_3$) δ 8.28–8.25 (m, 2H), 7.90–7.87 (m, 2H), 7.81 (d, *J* = 1.7 Hz, 1H), 7.63 (d, *J* = 1.7 Hz, 1H), 7.59–7.51 (m, 5H), 7.45–7.42 (m, 2H), 7.11–7.08 (m, 2H), 3.90 (s, 3H); **^{13}C{^{1}H} NMR** (75 MHz, $CDCl_3$) δ 163.7, 159.7, 147.7, 143.4, 139.6, 137.6, 133.4, 131.7, 129.4, 129.0, 128.9, 128.7, 127.7, 127.67, 126.9, 124.8, 123.1, 116.6, 114.4, 55.4; **HRMS** (EI) *m/z* $[M]^+$ calcd for $C_{26}H_{18}ClNO_2$ 411.1026, found 411.1025.

5. Mechanistic Studies

a) Deuteration of Arylsilver Species Using D_2O (Scheme 2)

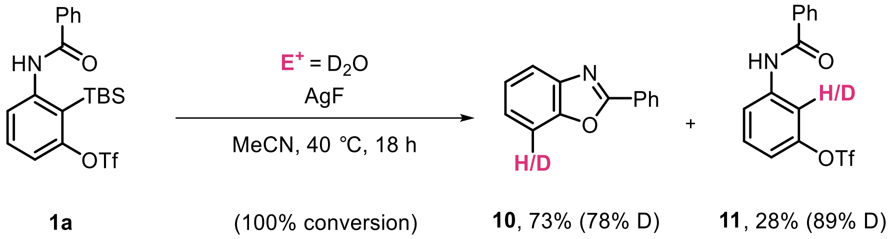

A sealed tube was charged with **1a** (179.2 mg, 0.39 mmol), AgF (152.2 mg, 1.20 mmol), and D_2O (0.11 mL, 6.00 mmol). The sealed tube and contents were placed under vacuum and back-filled with argon under a Schlenk line three times. Degassed anhydrous MeCN (3.0 mL, 0.1 M) was added under argon, and the reaction mixture was stirred in an oil bath at 40 °C for 18 h while being protected from light with aluminum foil. The reaction mixture was cooled to room temperature, celite-filtered with diethyl ether (3 × 10 mL) and concentrated under reduced pressure. The crude product was purified by flash column chromatography on silica gel using hexane/diethyl ether (20:1) to obtain pure **10** as a white solid (55.9 mg, 0.285 mmol, 73%) and **11** as a white solid (37.8 mg, 0.109 mmol, 28%). The level of deuterium incorporation was determined as 78% and 89%, respectively, based on ^{1}H NMR analysis.

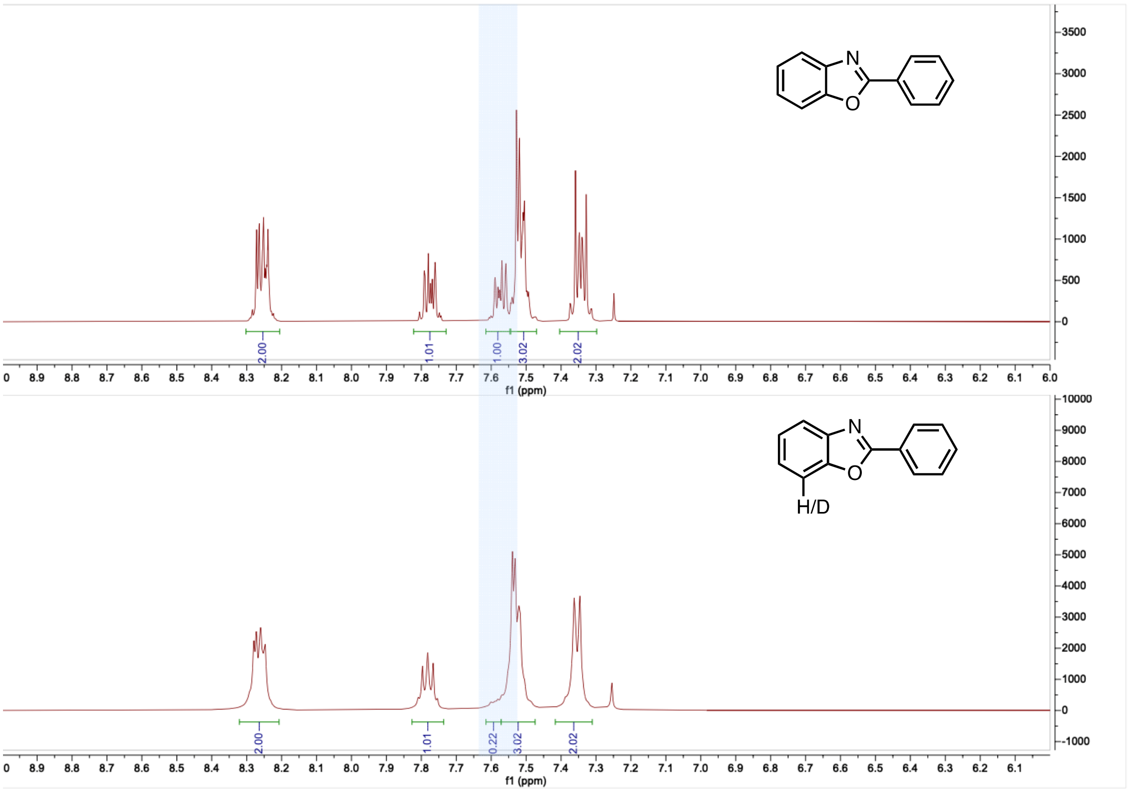

Figure S3. Comparison with ^{1}H NMR Data of **3a** and **10**

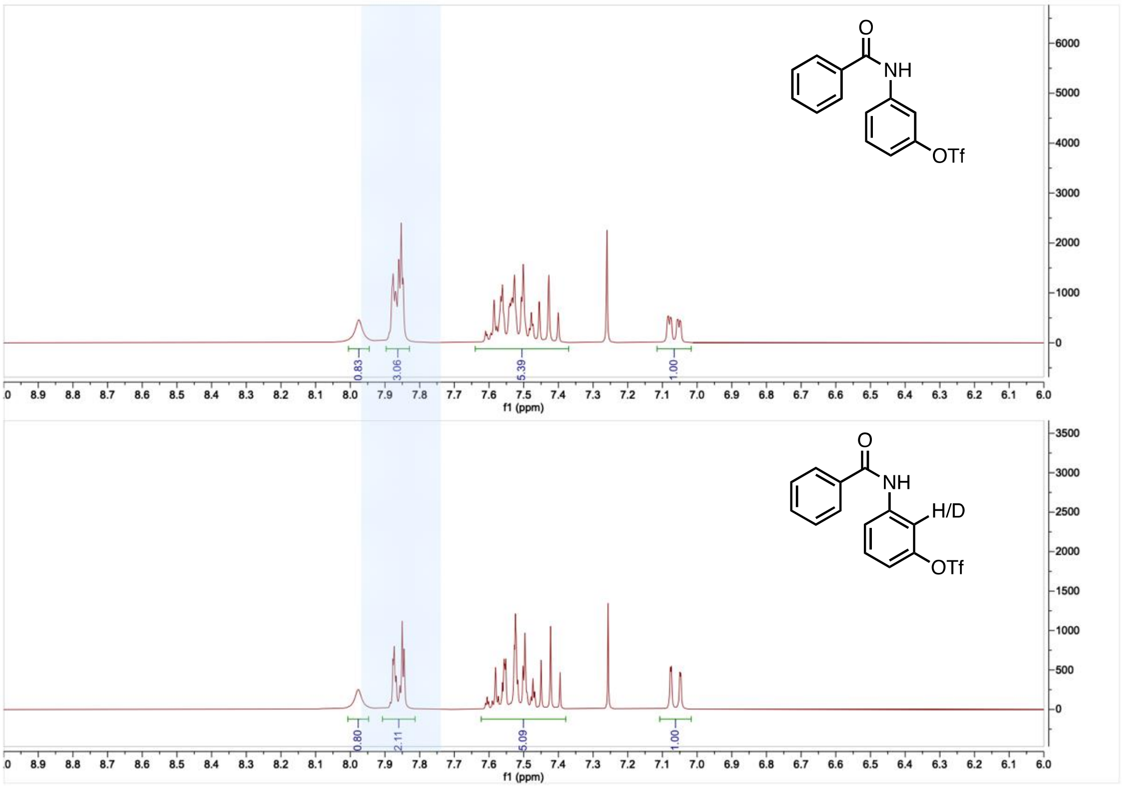

Figure S4. Comparison with ^{1}H NMR Data of 3-Benzamidophenyl Trifluoromethanesulfonate and **11**

b) Bromination of Arylsilver Species Using NBS (Scheme 2)

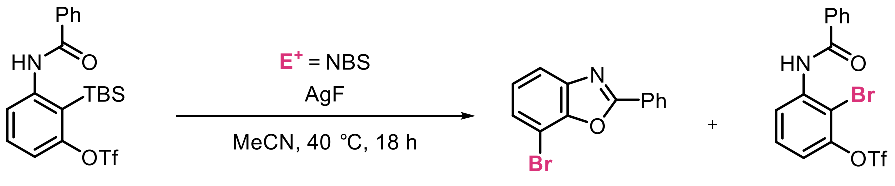

1a (72% conversion) **12**, 46% **13**, 15%

A sealed tube was charged with **1a** (179.2 mg, 0.39 mmol), AgF (152.2 mg, 1.20 mmol), and NBS (160.2 mg, 0.90 mmol). The sealed tube and contents were placed under vacuum and back-filled with argon under a Schlenk line three times. Degassed anhydrous MeCN (3.0 mL, 0.1 M) was added under argon, and the reaction mixture was stirred in an oil bath at 40 °C for 18 h while being protected from light with aluminum foil. The reaction mixture was cooled to room temperature, celite-filtered with diethyl ether (3 × 10 mL) and concentrated under reduced pressure. The crude product was purified by flash column chromatography on silica gel using hexane/diethyl ether (20:1) to obtain pure **12** (37.8 mg, 0.179 mmol, 46%) and **13** (24.6 mg, 0.058 mmol, 15%), respectively, as white solids.

7-Bromo-2-phenylbenzo[*d*]oxazole (12)

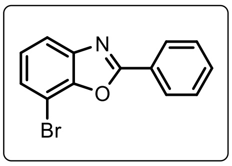

^{1}H NMR (300 MHz, $CDCl_3$) δ 8.24–8.19 (m, 2H), 7.64 (dd, J = 7.9 and 1.0 Hz, 1H), 7.49–7.40 (m, 4H), 7.16 (t, J = 8.0 Hz, 1H); **^{13}C{^{1}H} NMR** (75 MHz, $CDCl_3$) δ 162.9, 148.7, 142.6, 131.7, 128.7, 128.0, 127.7, 126.3, 125.5, 118.9, 102.4; Data are consistent with those reported in the literature.[8]

3-Benzamido-2-bromophenyl trifluoromethanesulfonate (13)

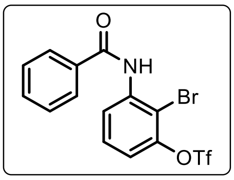

m.p: 80−82 °C; **R*f*** 0.28 (hexane/diethyl ether = 3:1); **^{1}H NMR** (300 MHz, $CDCl_3$) δ 8.65 (dd, *J* = 8.4 and 1.4 Hz, 1H), 8.54 (brs, 1H), 7.96–7.93 (m, 2H), 7.63–7.45 (m, 4H), 7.16 (dd, *J* = 8.3 and 1.4 Hz, 1H); **$^{13}C\{^1H\}$ NMR** (75 MHz, $CDCl_3$) δ 165.4, 147.0, 138.3, 134.0, 132.7, 129.3, 129.1, 127.1, 120.8, 118.6 (q, *J* = 320.7 Hz), 117.6, 107.7; **HRMS** (EI) *m/z* $[M]^+$ calcd for $C_{14}H_9BrF_3NO_4S$ 422.9388, found 422.9385.

c) Reaction of 2-phenyl Benzoxazole

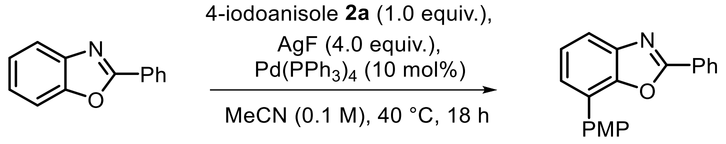

3a **4a**, n.d.

A sealed tube was charged with **3a** (76.1 mg, 0.39 mmol), 4-iodoanisole **2a** (70.2 mg, 0.3 mmol), AgF (152.2 mg, 1.2 mmol) and $Pd(PPh_3)_4$ (34.7 mg, 0.03 mmol). The sealed tube and contents were placed under vacuum and back-filled with argon under a Schlenk line three times. Degassed anhydrous MeCN (3.0 mL, 0.1 M) was added under argon, and the reaction mixture was stirred in an oil bath at 40 °C for 18 h. The reaction mixture was cooled to room temperature, celite-filtered with diethyl ether (3 × 10 mL) and concentrated under reduced pressure. As a result of ^{1}H NMR analysis, **4a** was not detected in the crude product.

d) Arylation Using an Arylpalladium Species 14

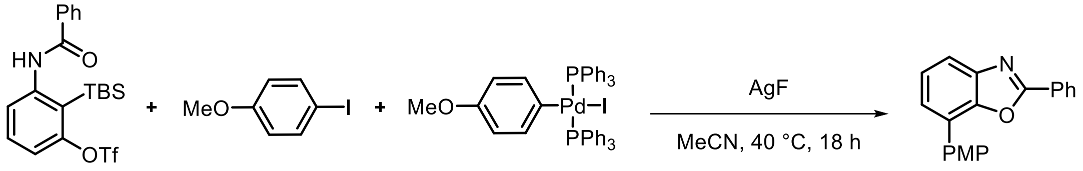

1a	2a	14	4a
1.3 equiv.	0.0 equiv.	1.0 equiv.	16%
1.3 equiv.	0.5 equiv.	0.5 equiv.	45%
1.3 equiv.	0.9 equiv.	0.1 equiv.	54%

A sealed tube was charged with **1a** (179.2 mg, 0.39 mmol), specified equivalents of 4-iodoanisole **2a**, AgF (152.2 mg, 1.20 mmol), and specified equivalents of **14**[9]. The sealed tube and contents were placed under vacuum and back-filled with argon under a Schlenk line three times. Degassed anhydrous MeCN (3.0 mL, 0.1 M) was added under argon, and the reaction mixture was stirred in an oil bath at 40 °C for 18 h. The reaction mixture was cooled to room temperature, celite-filtered with diethyl ether (3 × 10 mL) and concentrated under reduced pressure. The crude product was purified by flash column chromatography on silica gel using hexane/diethyl ether (20:1) to obtain pure **4a** as a white solid.

e) Synthesis of Arylsilver Species

An arylsilver species was synthesized according to the literature.[10]

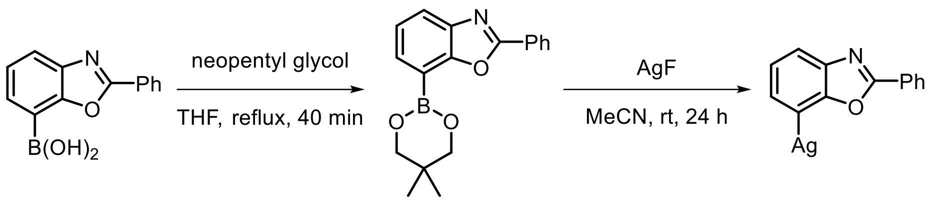

15a **15b** **15**

Scheme S2. Synthesis of Arylsilver Species **15**

7-(5,5-Dimethyl-1,3,2-dioxaborinan-2-yl)-2-phenylbenzo[*d*]oxazole (15b)

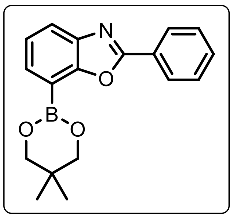

A solution of **15a** (301.2 mg, 1.26 mmol) and neopentyl glycol (157.3 mg, 1.51 mmol) in THF (12.6 mL) was refluxed for 40 min under a Dean Stark apparatus. After completion of the reaction as monitored by TLC analysis, the reaction mixture was concentrated under reduced pressure. The crude product was purified by flash column chromatography on silica gel (hexane:diethyl ether = 3:1) to obtain pure **15b** (383.9 mg, 1.25 mmol, 99%) as a white solid. **m.p**: 94–96 °C; **R*f*** 0.18 (hexane/diethyl ether = 3:1); **^{1}H NMR** (300 MHz, $CDCl_3$) δ 8.32–8.29 (m, 2H), 7.85 (dd, *J* = 7.9 and 1.3 Hz, 1H), 7.76 (dd, *J* = 7.4 and 1.4 Hz, 1H), 7.50–7.45 (m, 3H), 7.34 (t, *J* = 7.6 Hz, 1H), 3.84 (s, 4H), 1.03 (s, 6H); **^{13}C{^{1}H} NMR** (75 MHz, $CDCl_3$) δ 163.0, 154.8, 141.4, 131.2, 131.1, 128.6, 127.5, 127.2, 123.9, 122.1, 72.3, 31.7, 21.7; **^{11}B NMR** (96 MHz, $CDCl_3$) δ 26.7; **HRMS** (EI) *m/z* $[M]^+$ calcd for $C_{18}H_{18}NO_3B$ 307.1380, found 307.1376.

(2-Phenylbenzo[d]oxazol-7-yl)silver (15)

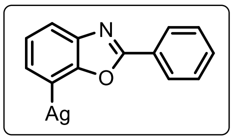

A sealed tube was charged with **15b** (100.1 mg, 0.326 mmol) and AgF (41.4 mg, 0.326 mmol). The sealed tube and contents were placed under vacuum and back-filled with argon under a Schlenk line three times. Degassed anhydrous MeCN (3.3 mL) was added under argon, and the reaction mixture was stirred for 24 h at room temperature while being protected from light with aluminum foil. During the reaction, AgF was dissolved and **15** was precipitated as a solid. The solid was collected by filtration and washed with diethyl ether (50 mL) to obtain pure **15** as a bright violet solid (45.3 mg, 0.15 mmol, 46%). **^{1}H NMR** (300 MHz, pyridine-*d_5*) δ 8.30–8.27 (m, 3H), 7.81 (dd, *J* = 7.8 and 1.3 Hz, 1H), 7.47–7.34 (m, 4H).

Note: Due to the low solubility and thermal instability, the title compound ***15*** *was not amenable to further characterization. Even in the only soluble pyridine-d_5, the title compound was decomposed over time.*

f) Stoichiometric Reaction of Arylpalladium Species 14 and Arylsilver Species 15

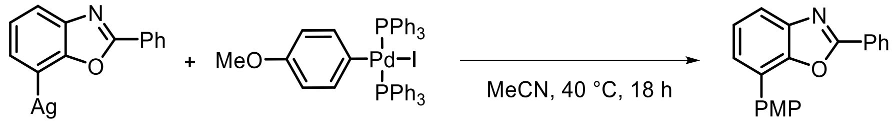

15 **14** **4a**, 45%

A sealed tube was charged with (2-phenylbenzo[d]oxazol-7-yl)silver **15** (39.3 mg, 0.13 mmol) and palladium oxidative addition complex **14** (86.5 mg, 0.100 mmol). The sealed tube and contents were placed under vacuum and back-filled with argon under a Schlenk line three times. Degassed anhydrous MeCN (3.0 mL, 0.1 M) was added under argon, and the reaction mixture was stirred in an oil bath at 40 °C for 18 h. The reaction mixture was cooled to room temperature, celite-filtered with diethyl ether (3 × 10 mL) and concentrated under reduced pressure. The crude product was purified by flash

column chromatography on silica gel using hexane/diethyl ether (15:1) to obtain pure **4a** as a white solid (13.5 mg, 0.045 mmol, 45%).

General Procedure for Quenching Experiments

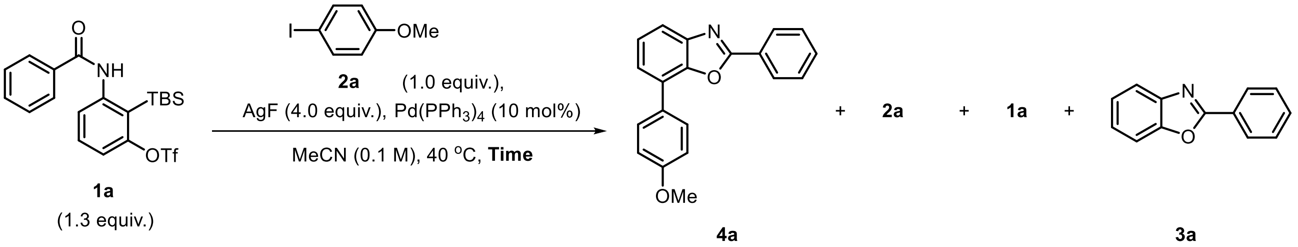

A sealed tube was charged with **1a** (0.13 mmol, 1.3 equiv.), 4-iodoanisole **2a** (0.1 mmol, 1.0 equiv.), AgF (0.4 mmol, 4.0 equiv.) and $Pd(PPh_3)_4$ (0.01 mmol, 10 mol%). The sealed tube and contents were placed under vacuum and back-filled with argon under a Schlenk line three times. Degassed anhydrous solvent (1.0 mL, 0.1 M) then added under argon, and the reaction mixture was stirred in an oil bath at 40 °C. After being stirred for the indicated period, the reaction mixture was cooled to room temperature and quenched with 1N HCl. The reaction mixture was extracted with diethyl ether (3 × 10 mL). The organic phase was collected, dried over anhydrous $MgSO_4$, filtered, and concentrated under reduced pressure. The yields were determined by ^{1}H NMR analysis using ethylene carbonate as an internal standard.

Table S5. Quenching Experiments[a]

Entry	Time (h)	4a (%)[b]	2a (%)[b]	1a (%)[b]	3a (%)[b]
1	2	32	61	67	24
2	6	51	40	41	30
3	10	70	26	20	34
4	14	82	11	13	28
5	18	94	0	4	24

[a]Reactions were performed on a 0.1 mmol scale following the general procedure. [b]Yields were determined by ^{1}H NMR analysis using ethylene carbonate as an internal standard.

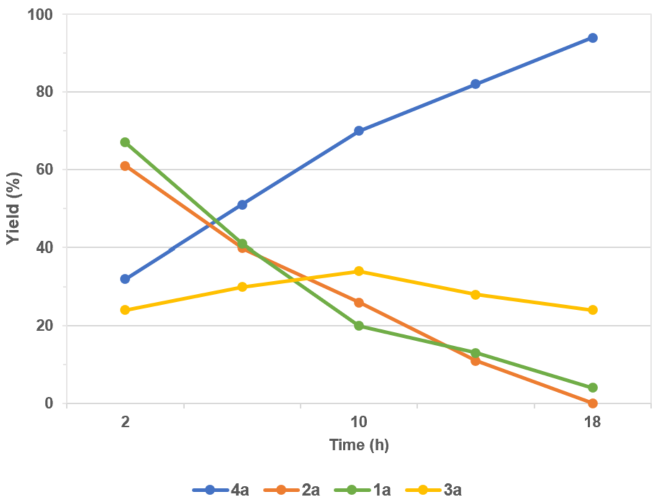

Figure S5. Analysis of the reaction intermediates by HCl quenching

6. Computational Studies
6.1. General Procedure (Figure 2)

All density functional theory (DFT) calculations were carried out using the Gaussian 16.[11] The structures of stationary points were optimized at the SMD(MeCN)/B3LYP/6-31G**/LANL2DZ(Ag) level of theory.[12-16] Frequency calculations were performed at the same level of theory to verify whether the stationary points are minima or transition states, and to obtain thermal correction for the Gibbs free energy. Additionally, intrinsic reaction coordinate (IRC) calculations were conducted to validate the connectivity of transition states between reactants and products. Single-point corrections to energy were carried out at the SMD(MeCN)/M06/6-311++G**/SDD(Ag) level of theory,[17-19] with an addition of 1.89 kcal/mol to the final energies to account for 1 mol/L standard state for the liquid. Figures of three-dimensional molecular structures in Supporting Information were prepared using CYLview20.[20]

6.2. Estimation of the Solvation Energy of Ag Atom

For the thermodynamic estimation of dissociation of AgF in the MeCN solution, we calculated the solvation energy of silver cation using the cluster-continuum model.[21-23] Solution structure of Ag ion in MeCN is experimentally determined as $Ag(MeCN)_4^+$.[24] Therefore, we used 1:4 stoichiometry for computing the gas-phase clustering free energy ($\Delta G^{\circ}_{g,bind}$) at 1 atm and the solvation free energy of the cluster (Figure S5).

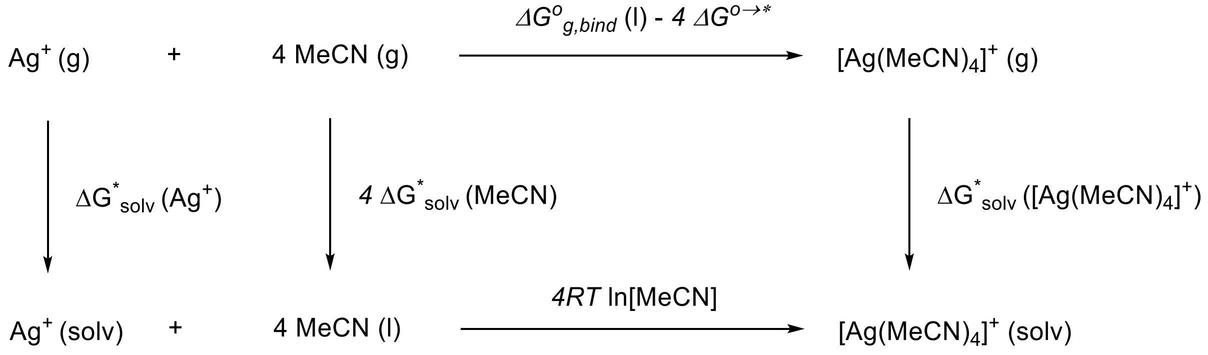

Figure S5. Thermodynamic Cycle Used within the Cluster-continuum Model

The solvation free energy of Ag+ ion in the cluster-continuum model can be calculated from eq 1.

$$\Delta G^{*}_{solv}(Ag^{+}) = \Delta G^{\circ}_{g,bind}([Ag(MeCN)_4]^{+}) + \Delta G^{*}_{solv}([Ag(MeCN)_4]^{+}) - 4\Delta G^{*}_{solv}(MeCN) - 4RTln[MeCN] - 4\Delta G^{\circ\rightarrow *} \quad (1)$$

Where $\Delta G^{\circ}_{g,bind}([Ag(MeCN)_4]^{+})$ is the gas-phase clustering free energy from Ag^{+} and 4 MeCN molecules at 1 atm, $\Delta G^{*}_{solv}([Ag(MeCN)_4]^{+})$ is the solvation free energy of the cluster, $\Delta G^{*}_{solv}(MeCN)$ is the solvation free energy of a single MeCN molecule, and $\Delta G^{\circ\rightarrow *}$ is the correction term for the free-energy change of the transition of an ideal gas from 1 atm to 1 mol/L at 298.15 K.

Using the cluster-continuum model, we determined the solvation free energy of silver ion to be -119 kcal/mol (Table S5). Using this result, we computed the dissociation of AgF in MeCN is computed and found that the process is exergonic

by 11.3 kcal/mol. This result is consistent with the solubility of AgF in MeCN, and we assumed that AgF is fully dissociated in the reaction mixture.

Table S6. Calculated Data Used to Derive the Solvation Free Energy of the Silver Ion (eq 1).

		**Results (kcal/mol)**
1.	$\Delta G^{\circ}_{g,bind}([Ag(MeCN)_4]^+)$	-86.15
2.	$\Delta G^{*}_{solv}([Ag(MeCN)_4]^+)$	-45.34
3.	$\Delta G^{*}_{solv}(MeCN)$	-6.76
4.	RTln[MeCN]	1.75
5.	Solvation free energy of Ag^+ in MeCN	-119.00

7. References

[1] Y.-K. Jeon, W.-S. Kim, *Org. Lett.* **2021**, *23*, 7545–7549.

[2] Y.-Y. Lin, Y.-J. Lin, C.-H. Wang, J.-H. Cheng, C.-F. Lee, *J. Org. Chem.***2012**, *77*, 6100−6106.

[3] K. Li, A. Alexakis, *Angew. Chem., Int. Ed.* **2006**, *45*, 7600−7603.

[4] Y.-Y. Che, Y. Yue, L.-Z. Lin, B. Pei, X. Deng, C. Feng, *Angew. Chem. Int. Ed.* **2020**, *59*, 16414–16419.

[5] L. Tang, X. Guo, Y. Yang, Z. Zha, Z. Wang, *Chem. Commun.* **2014**, *50*, 6145−6148.

[6] A. Patra, A. James, T. K. Das, A. T. Biju, *J. Org. Chem.* **2018**, *83*, 14820−14826.

[7] K. Amaike, K. Muto, J. Yamaguchi, K. Itami, *J. Am. Chem. Soc.* **2012**, *134*, 13573−13576.

[8] F. Abdellaoui, C. Youssef, H. B. Ammar, T. Roisnel, J.-F. Soulé, H. Doucet, *ACS Catal*. **2016**, *6*, 4248–4252.

[9] D. Whitaker, J. Burés, I. Larrosa, *J. Am. Chem. Soc.* **2016**, *138*, 8384−8387.

[10] A. Baur, K. A. Bustin, E. Aguilera, J. L. Petersen, J. M. Hoover, *Org. Chem. Front.* **2017**, *4*, 519−524.

[11] Gaussian 16, Revision A.03, M. J. Frisch, G. W. Trucks, H. B. Schlegel, G. E. Scuseria, M. A. Robb, J. R. Cheeseman, G. Scalmani, V. Barone, G. A. Petersson, H. Nakatsuji, X. Li, M. Caricato, A. V. Marenich, J. Bloino, B. G. Janesko, R. Gomperts, B. Mennucci, H. P. Hratchian, J. V. Ortiz, A. F. Izmaylov, J. L. Sonnenberg, Williams, F. Ding, F. Lipparini, F. Egidi, J. Goings, B. Peng, A. Petrone, T. Henderson, D. Ranasinghe, V. G. Zakrzewski, J. Gao, N. Rega, G. Zheng, W. Liang, M. Hada, M. Ehara, K. Toyota, R. Fukuda, J. Hasegawa, M. Ishida, T. Nakajima, Y. Honda, O. Kitao, H. Nakai, T. Vreven, K. Throssell, J. A. Montgomery Jr., J. E. Peralta, F. Ogliaro, M. J. Bearpark, J. J. Heyd, E. N. Brothers, K. N. Kudin, V. N. Staroverov, T. A. Keith, R. Kobayashi, J. Normand, K. Raghavachari, A. P. Rendell, J. C. Burant, S. S. Iyengar, J. Tomasi, M. Cossi, J. M. Millam, M. Klene, C. Adamo, R. Cammi, J. W. Ochterski, R. L. Martin, K. Morokuma, O. Farkas, J. B. Foresman, D. J. Fox, Gaussian, Inc., Wallingford, CT, 2016.

[12] C. Lee, W. Yang, R. G. Parr, *Phys. Rev. B* **1988**, *37*, 785−789.

[13] A. D. Becke, *J. Chem. Phys.* **1993**, *98*, 5648−5652.

[14] P. J. Hay, W. R. Wadt, *J. Chem. Phys.* **1985**, *82*, 299−310.

[15] L. E. Roy, P. J. Hay, R. L. Martin, *J. Chem. Theory Comput.* **2008**, *4*, 1029−1931.

[16] A. V. Marenich, C. J. Cramer, D. G. Truhlar, *J. Phys. Chem. B* **2009**, *113*, 6378−6396.

[17] Y. Zhao, D. G. Truhlar, *Theor. Chem. Acc.* **2008**, *120*, 215−241.

[18] Y. Zhao, D. G. Truhlar, *Acc. Chem. Res.* **2008**, *41*, 157−167.

[19] D. Andrae, U. Häußermann, M. Dolg, H. Stoll, H. Preuß, *Theor. Chim. Acta.* **1990**, *77*, 123–141.

[20] C. Y. Legault, *CYLview20*, Université de Sherbrooke, 2020 (http://www.cylview.org)

[21] J. R. Pliego, J. M. Riveros, *J. Phys. Chem. A* **2001**, *105*, 7241−7247.

[22] V. S. Bryantsev, M. S. Diallo, W. A. Goddard, *J. Phys. Chem. B.* **2008**, *112*, 9709−9719.

[23] L. Tomaník, E. Muchová, P. Slavíček, *Phys. Chem. Chem. Phys.* **2020**, *22*, 22357−22368.

[24] T. Ichikawa, H. Yoshida, A. S. W. Li, L. Kevan, *J. Am. Chem. Soc.* **1984**, *106*, 4324−4327.

Appendix 1: ^{1}H, ^{13}C, and ^{29}Si NMR Spectra

Figure S1, **1a**, ^{1}H NMR-300 MHz in $CDCl_3$ and ^{13}C NMR-75 MHz in $CDCl_3$

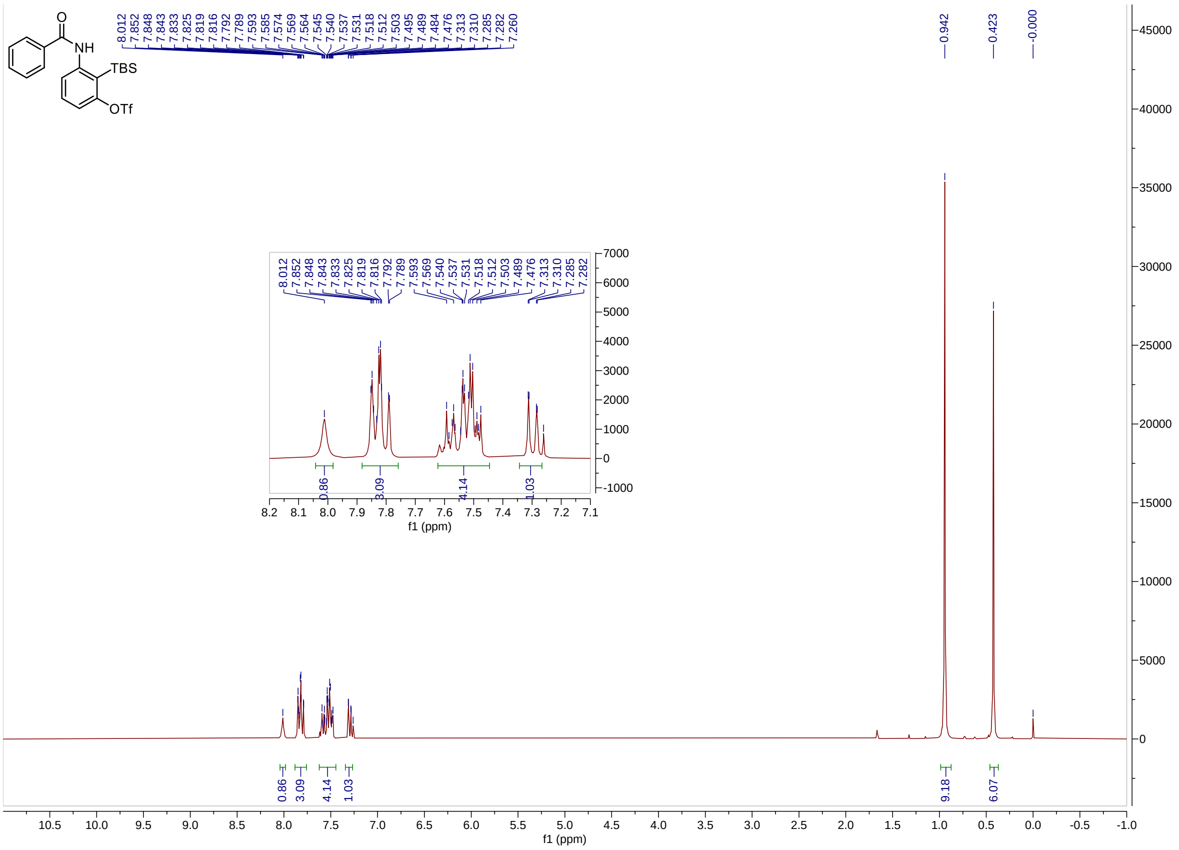

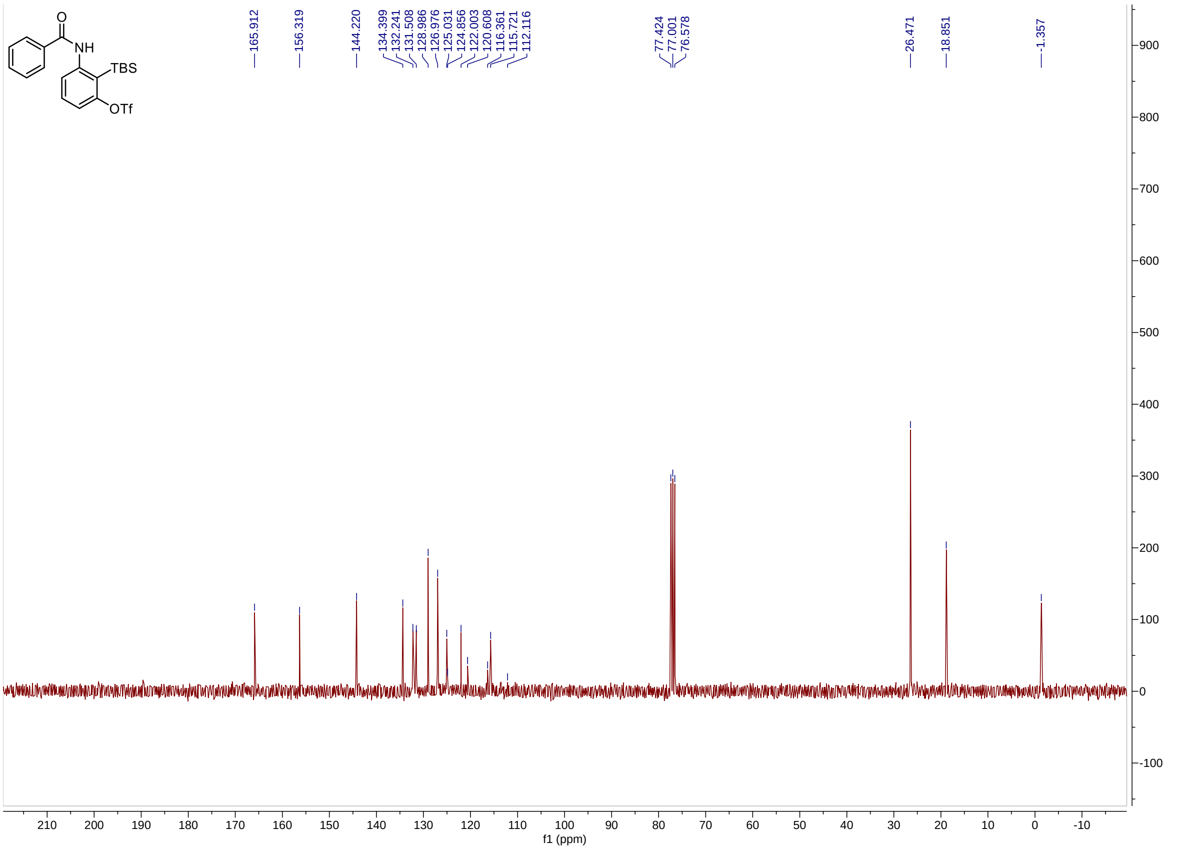

Figure S1, **1b**, ^{1}H NMR-300 MHz in $CDCl_3$ and ^{13}C NMR-75 MHz in $CDCl_3$

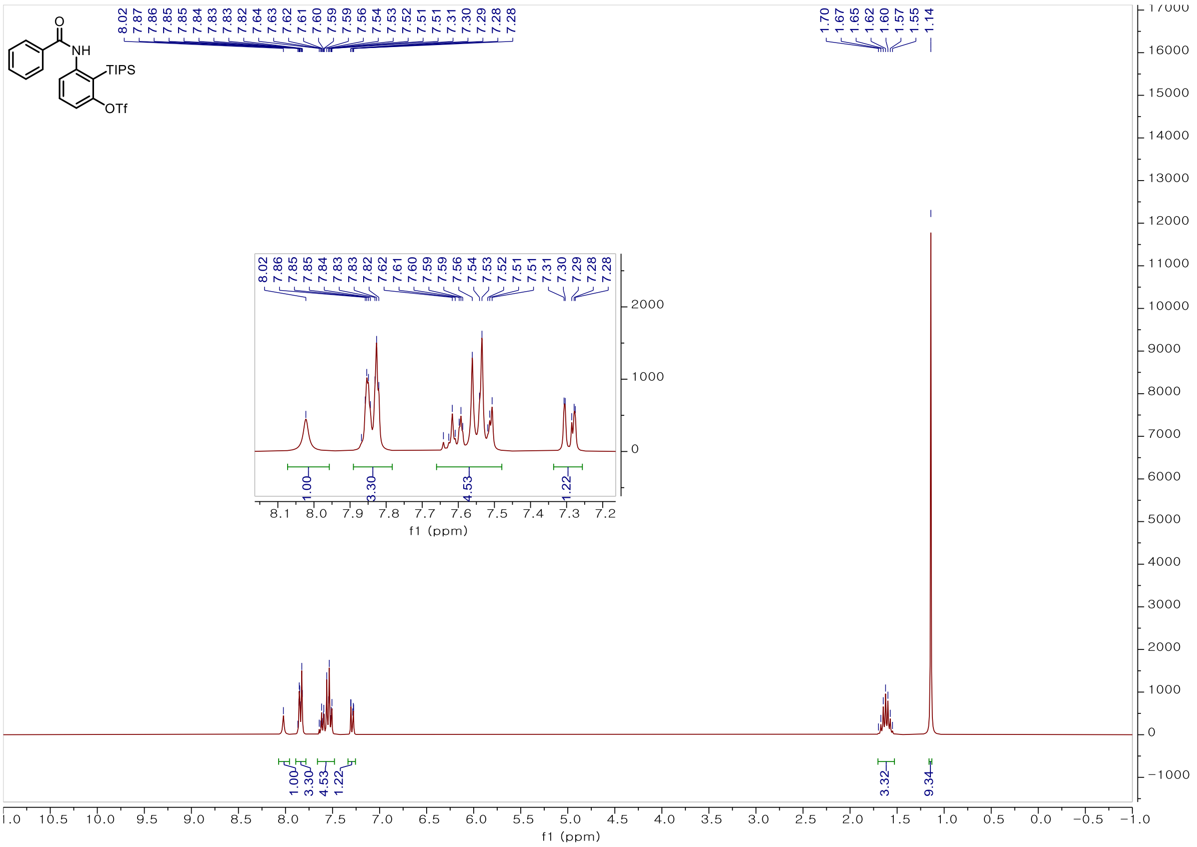

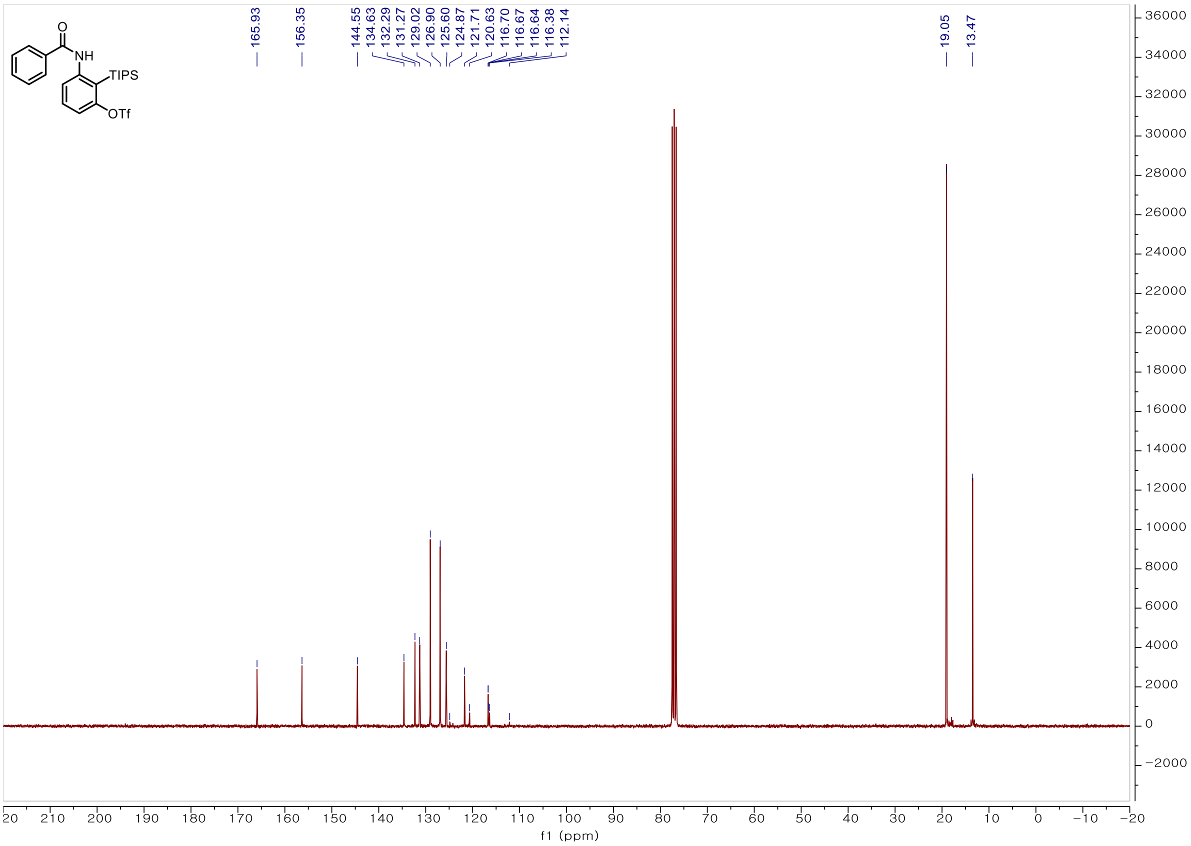

Figure S1, **1c**, ^{1}H NMR-300 MHz in $CDCl_3$ and ^{13}C NMR-75 MHz in $CDCl_3$

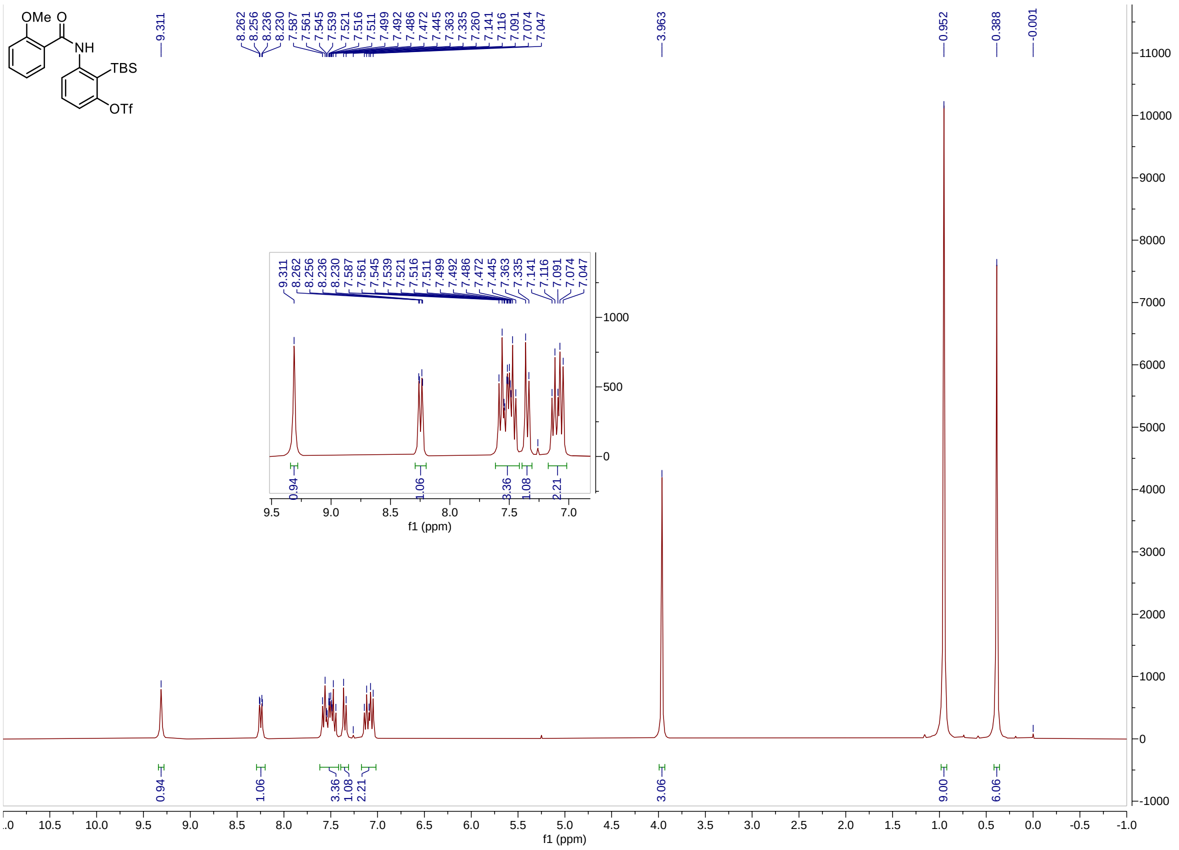

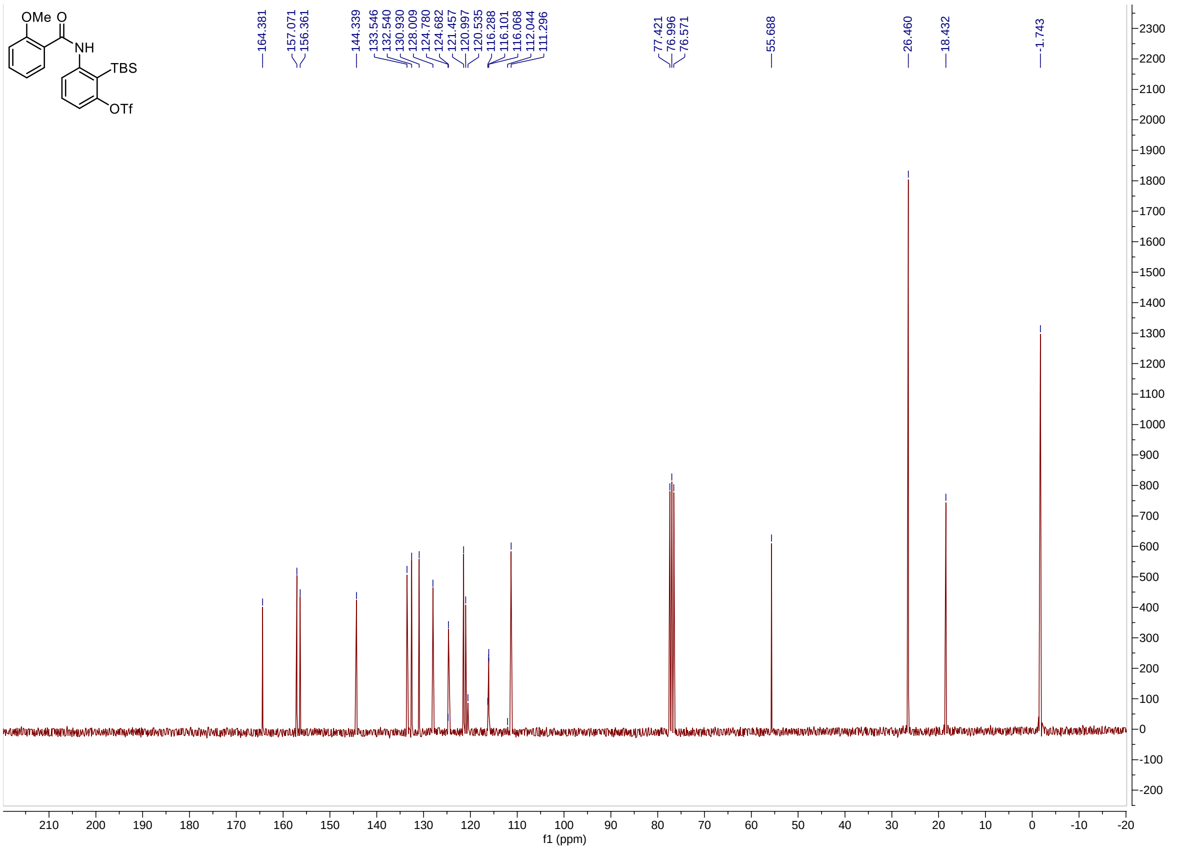

Figure S1, **1d**, ^{1}H NMR-300 MHz in $CDCl_3$ and ^{13}C NMR-75 MHz in $CDCl_3$

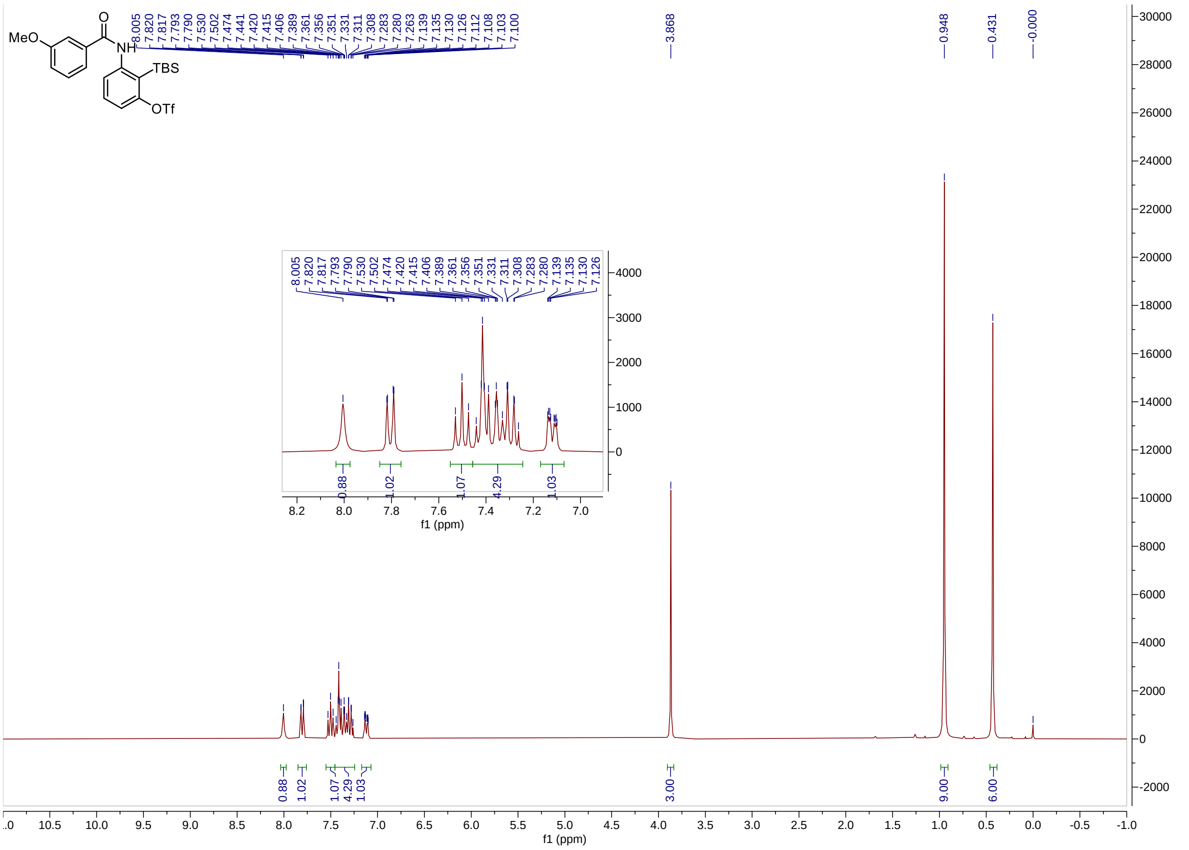

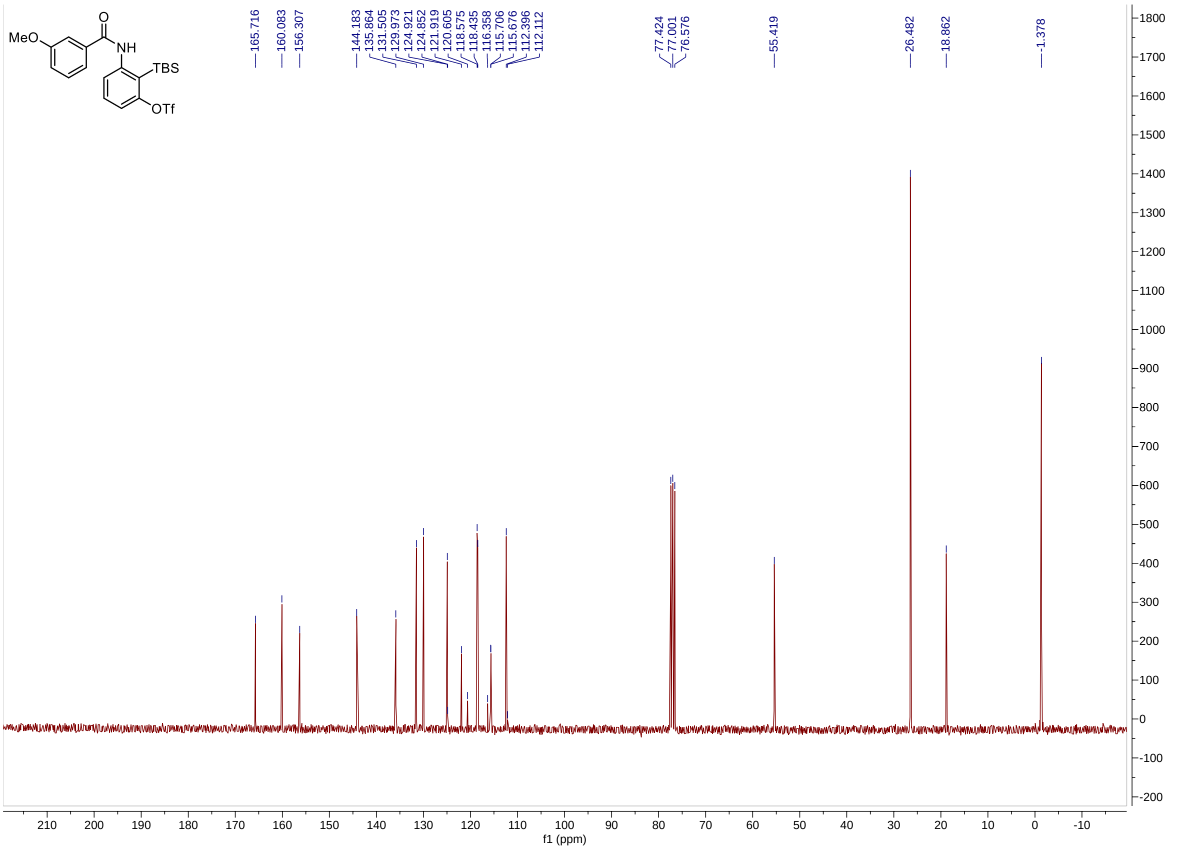

Figure S1, **1e**, ^{1}H NMR-300 MHz in $CDCl_3$ and ^{13}C NMR-75 MHz in $CDCl_3$

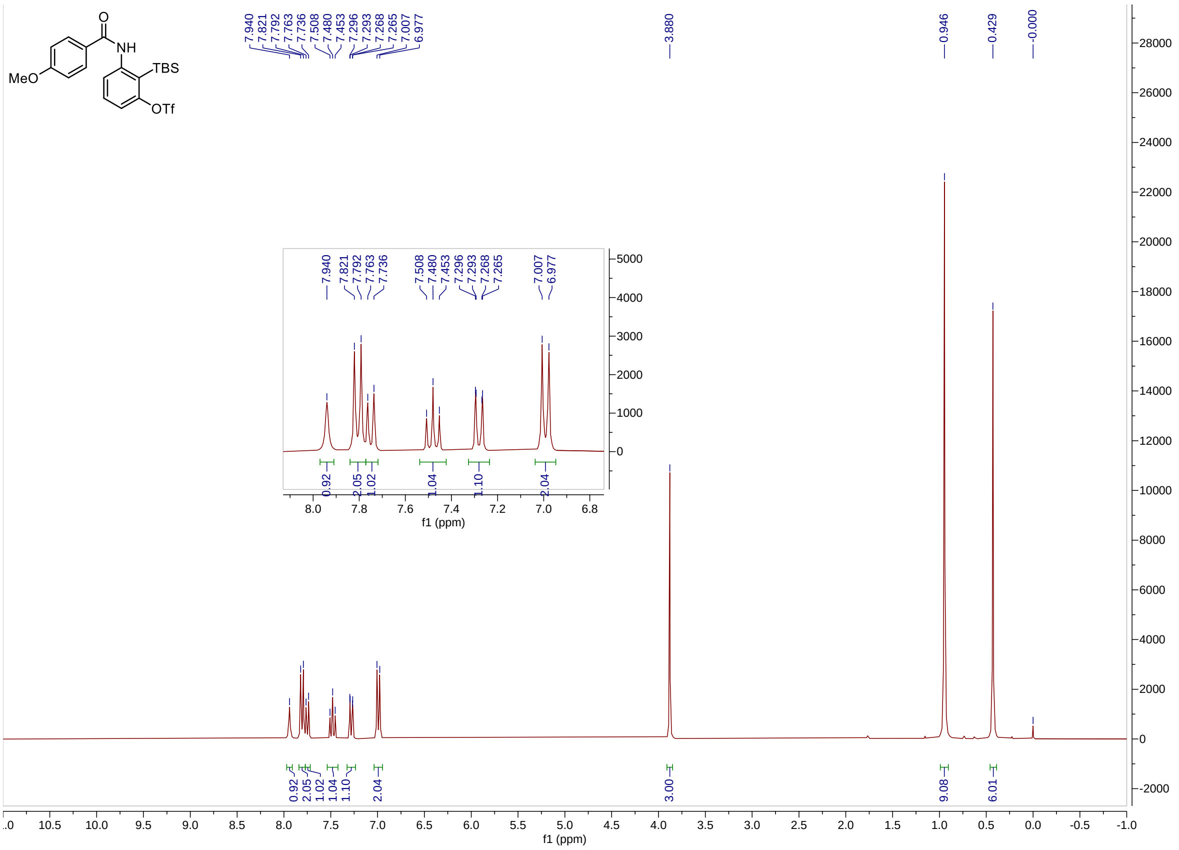

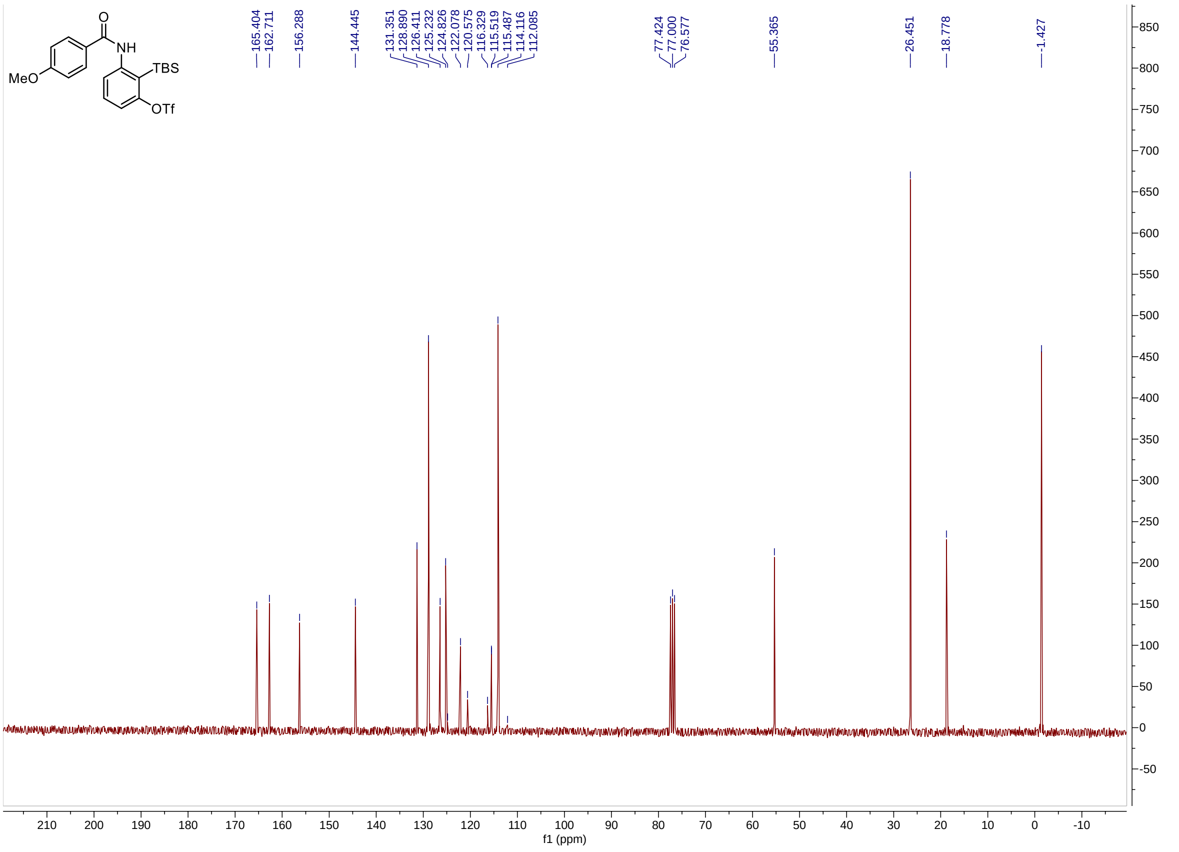

Figure S1, **1f**, ^{1}H NMR-300 MHz in $CDCl_3$ and ^{13}C NMR-75 MHz in $CDCl_3$

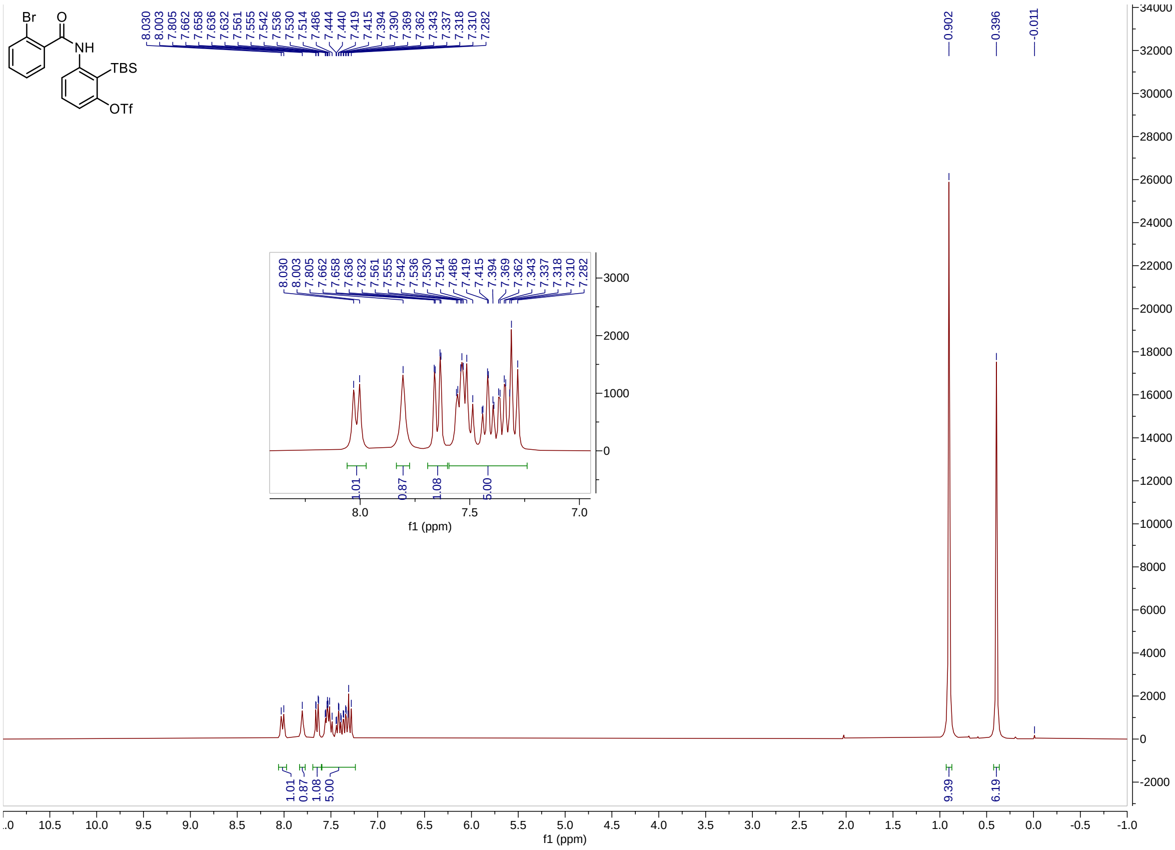

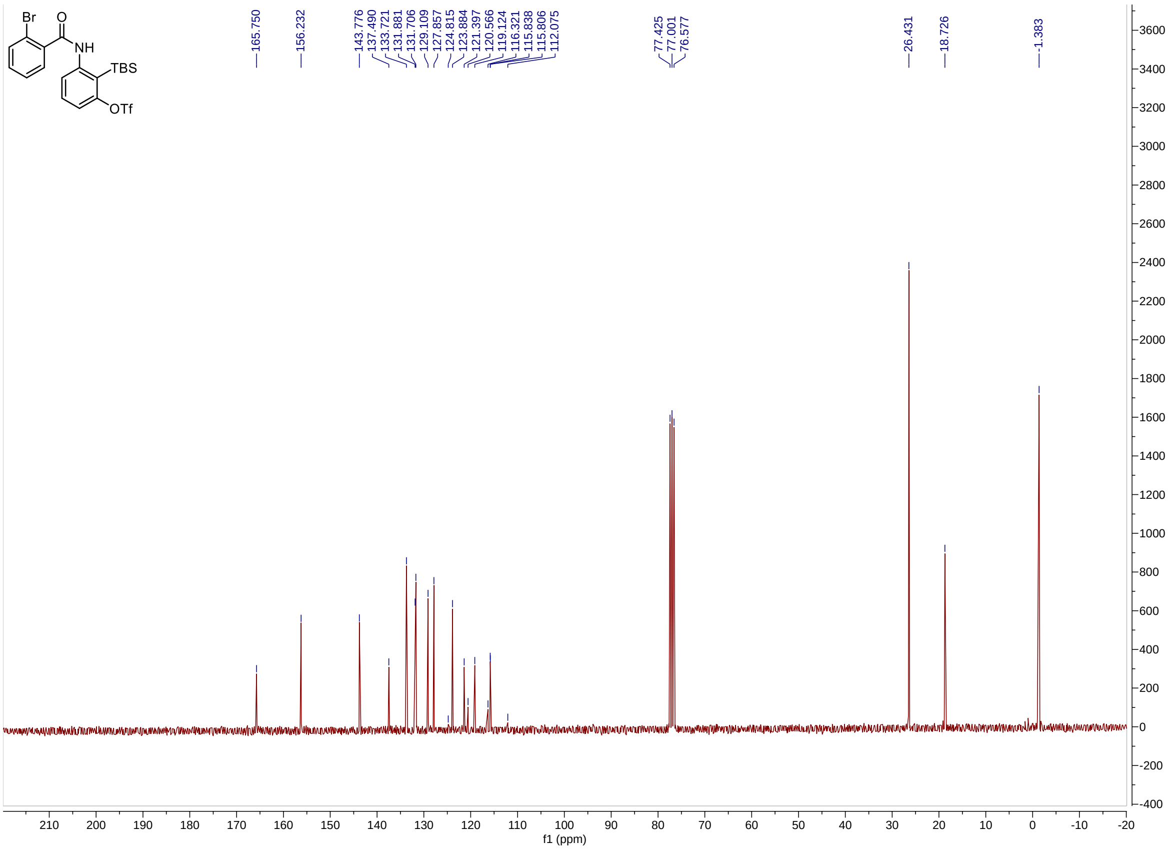

Figure S1, **1g**, ^{1}H NMR-300 MHz in $CDCl_3$ and ^{13}C NMR-75 MHz in $CDCl_3$

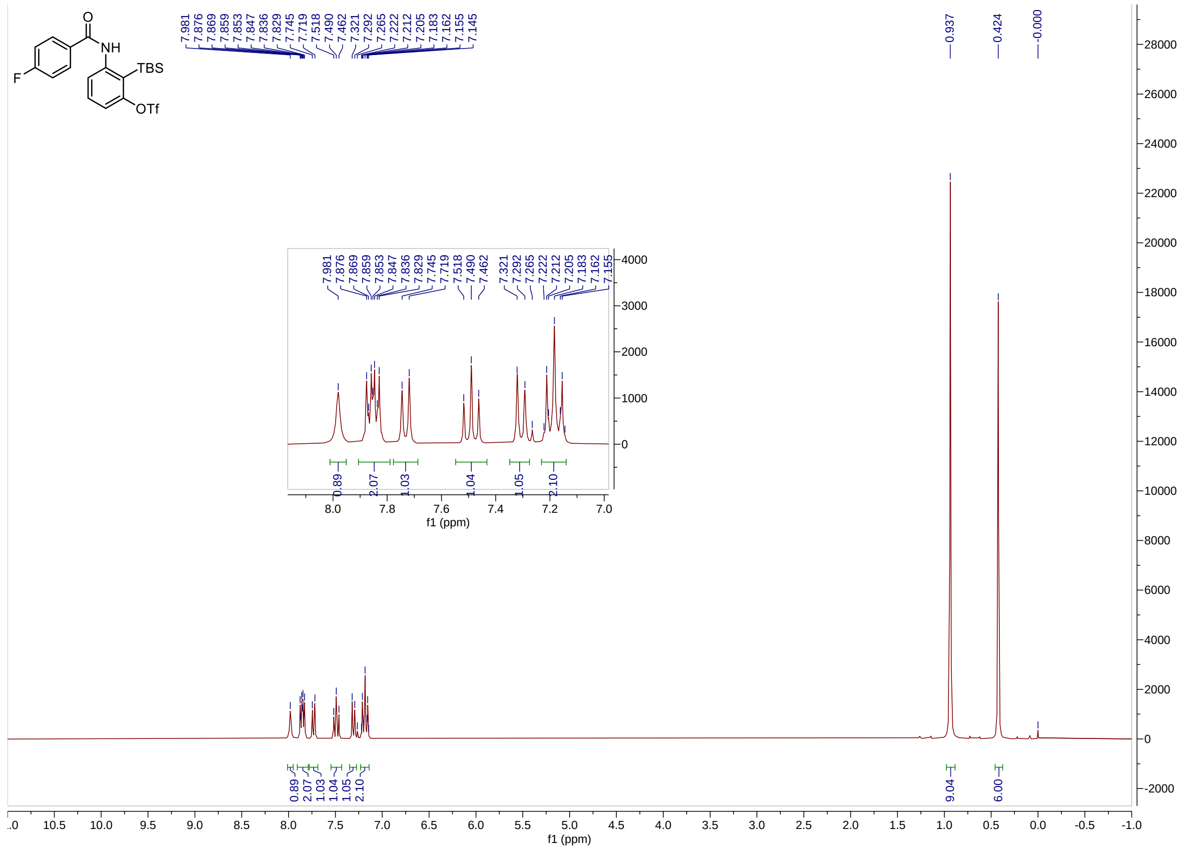

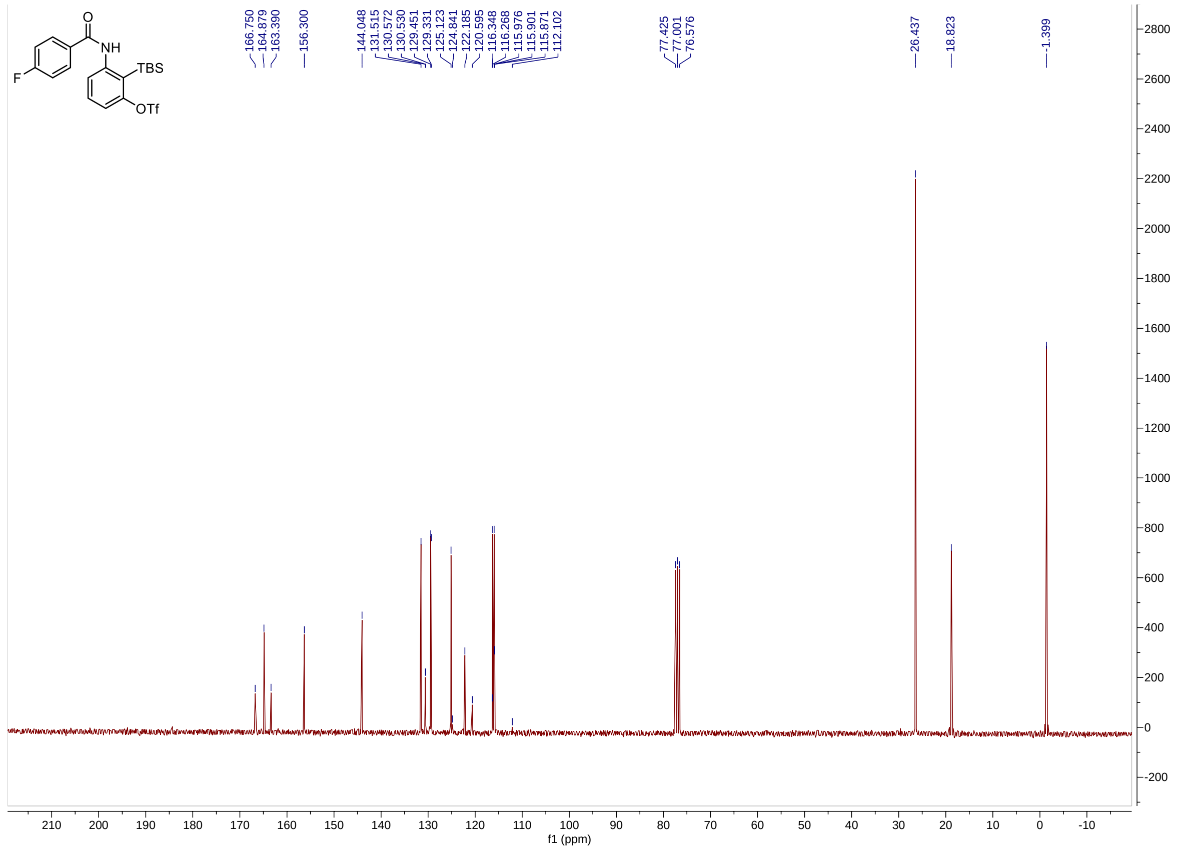

Figure S1, **1h**, ^{1}H NMR-300 MHz in $CDCl_3$ and ^{13}C NMR-75 MHz in $CDCl_3$

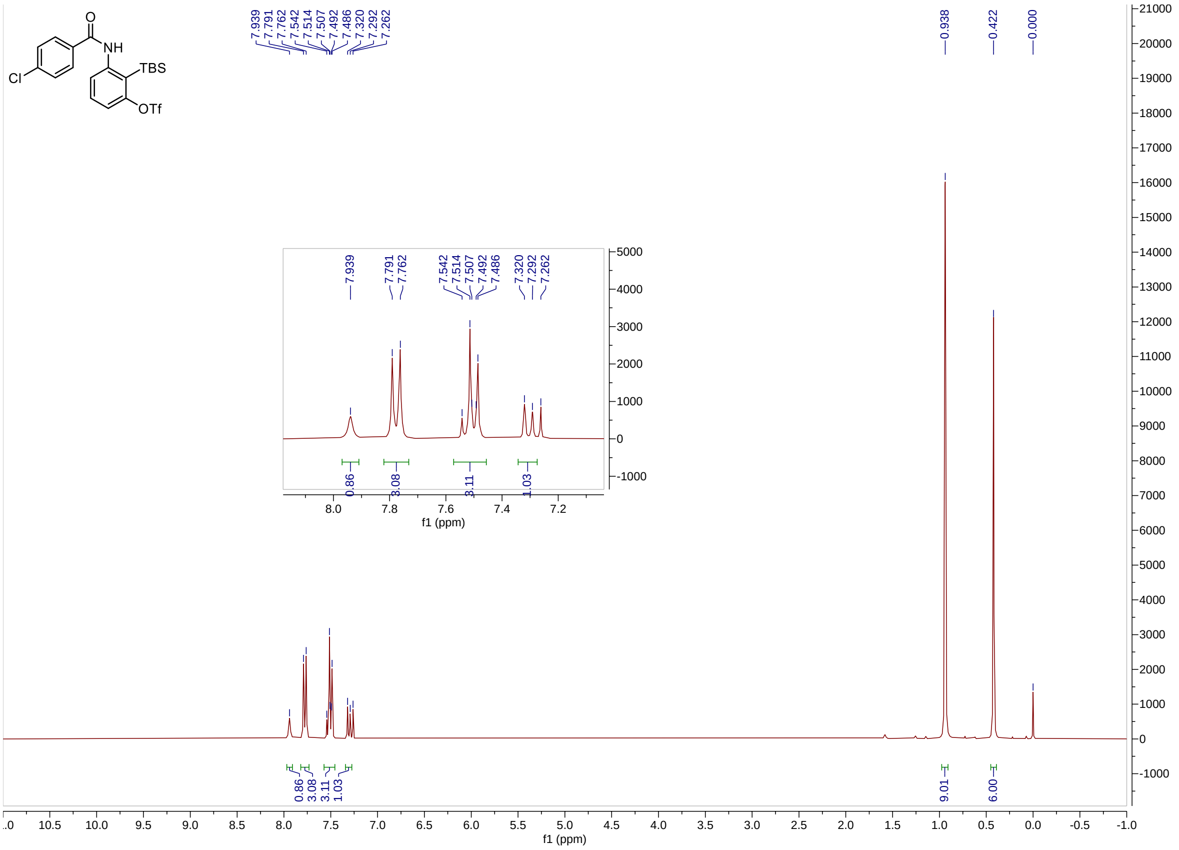

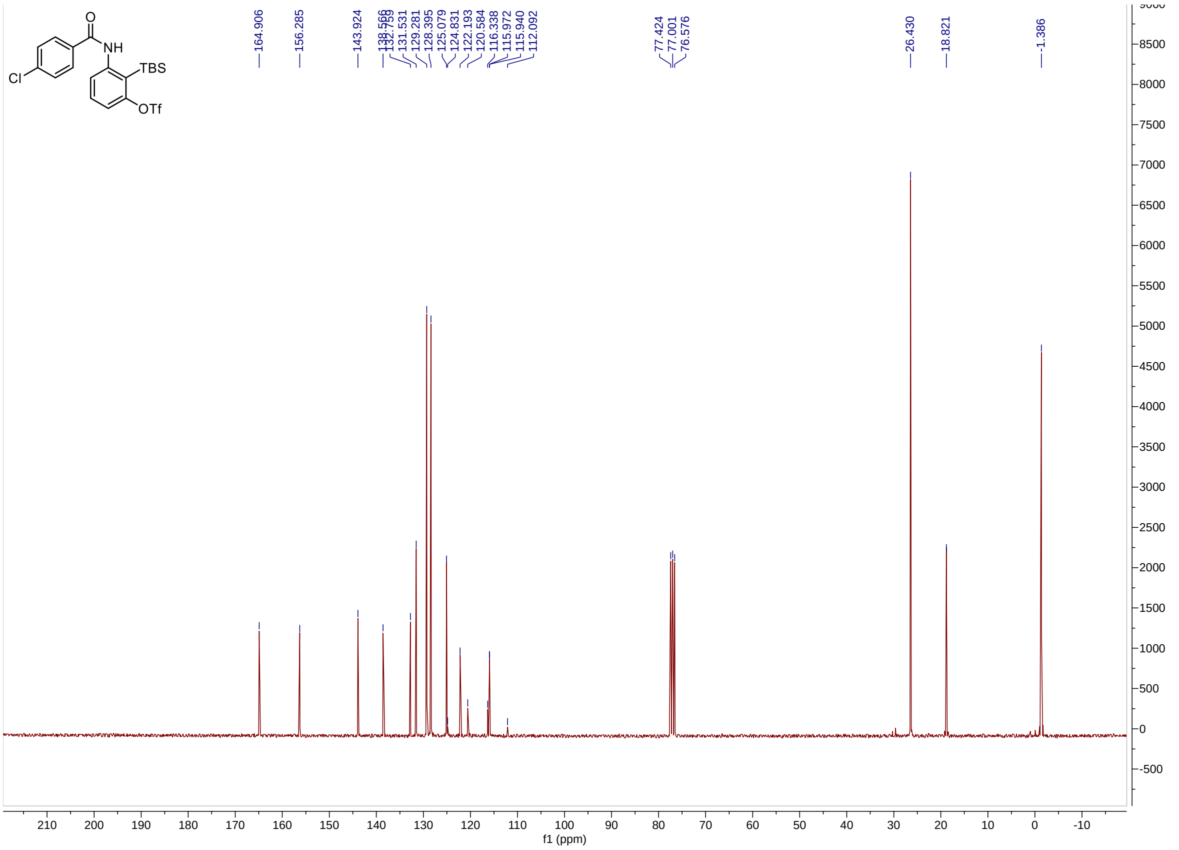

Figure S1, **1i**, ^{1}H NMR-300 MHz in $CDCl_3$ and ^{13}C NMR-75 MHz in $CDCl_3$

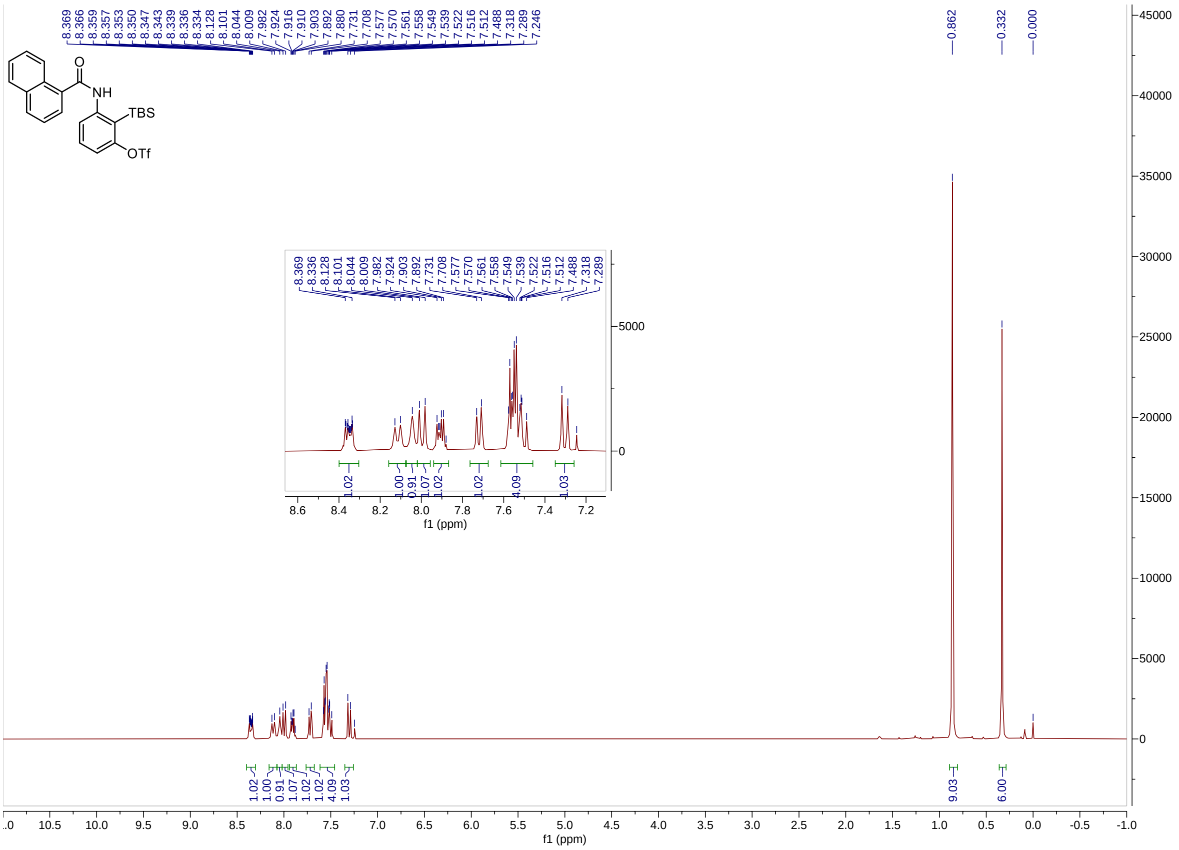

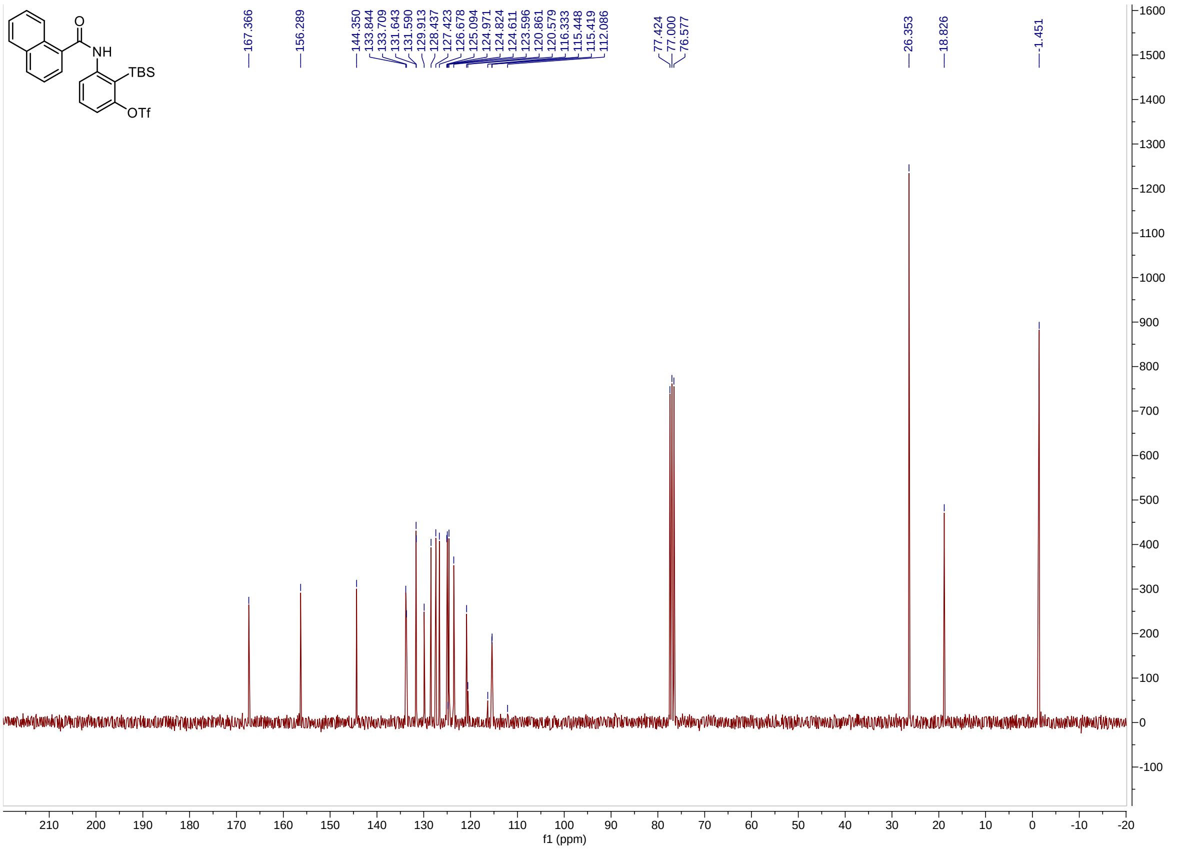

Figure S1, **1j**, ^{1}H NMR-300 MHz in $CDCl_3$ and ^{13}C NMR-75 MHz in $CDCl_3$

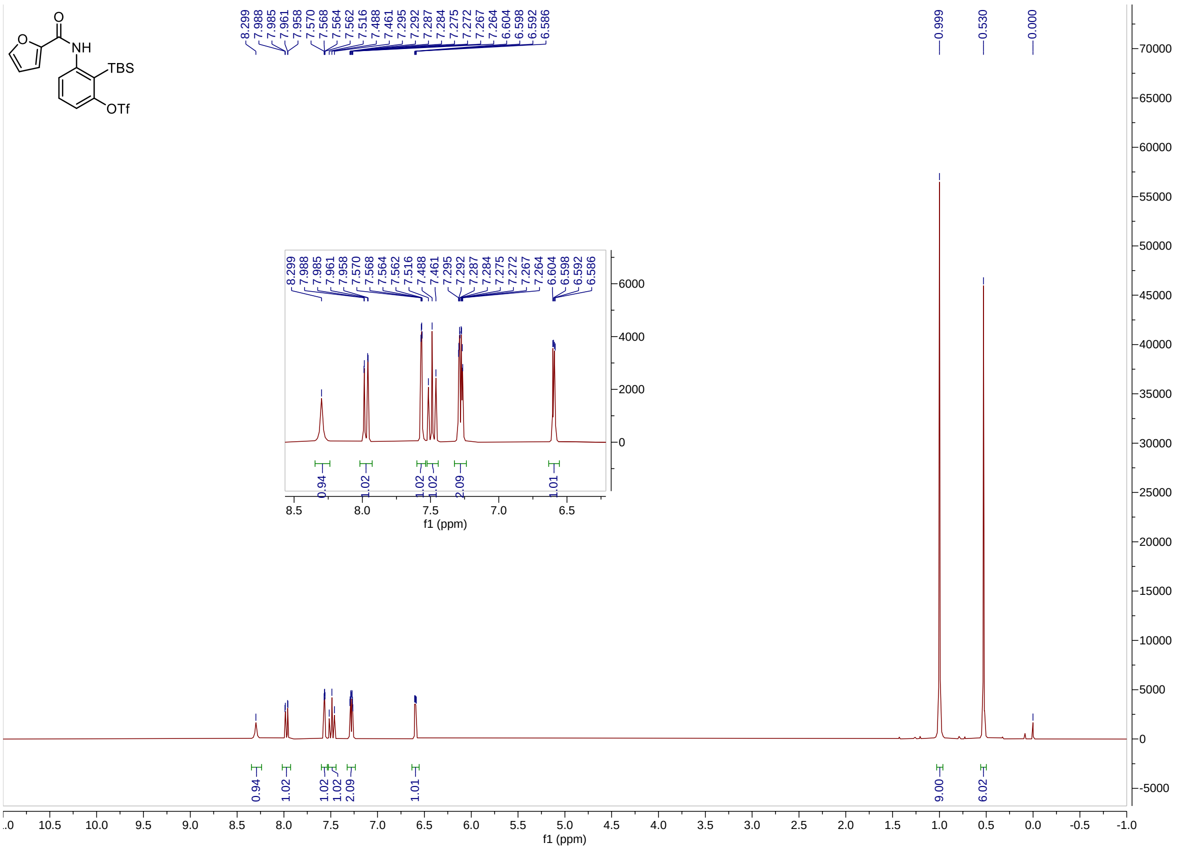

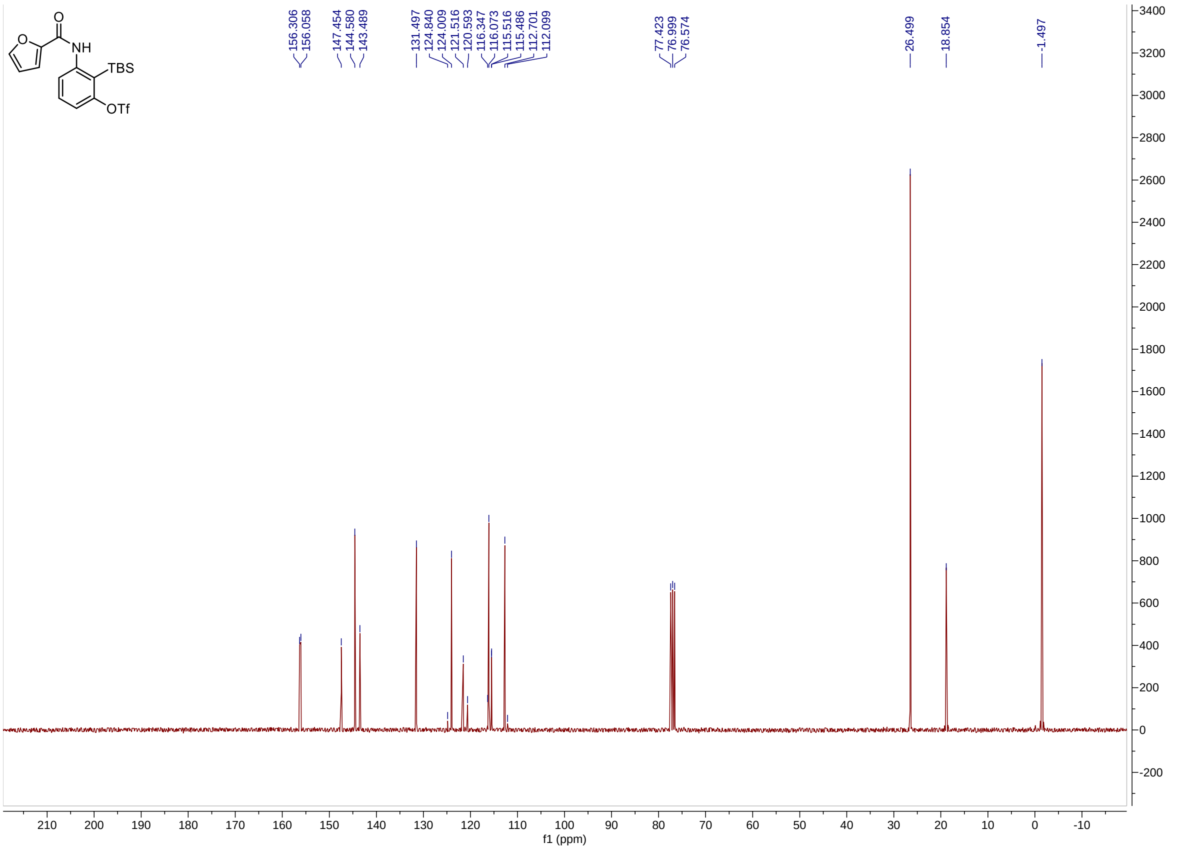

Figure S1, **1k**, ^{1}H NMR-300 MHz in $CDCl_3$ and ^{13}C NMR-75 MHz in $CDCl_3$

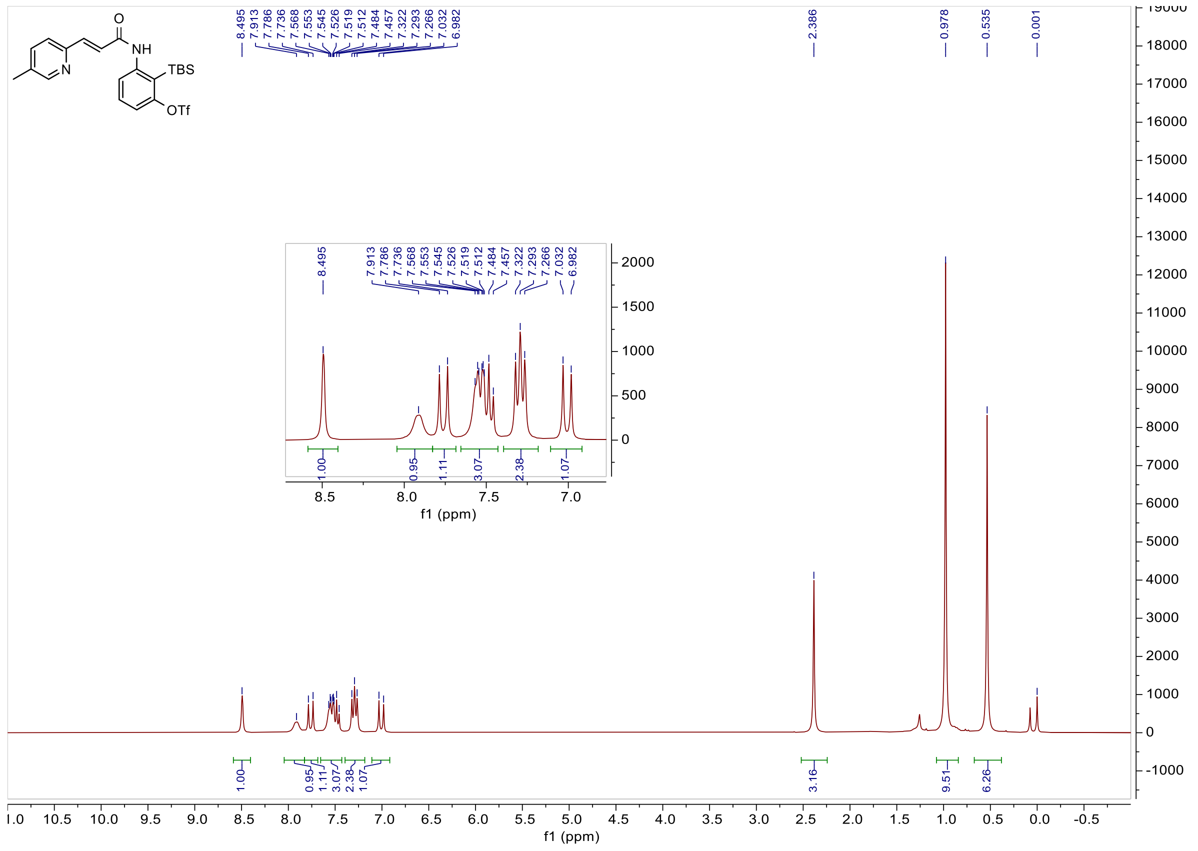

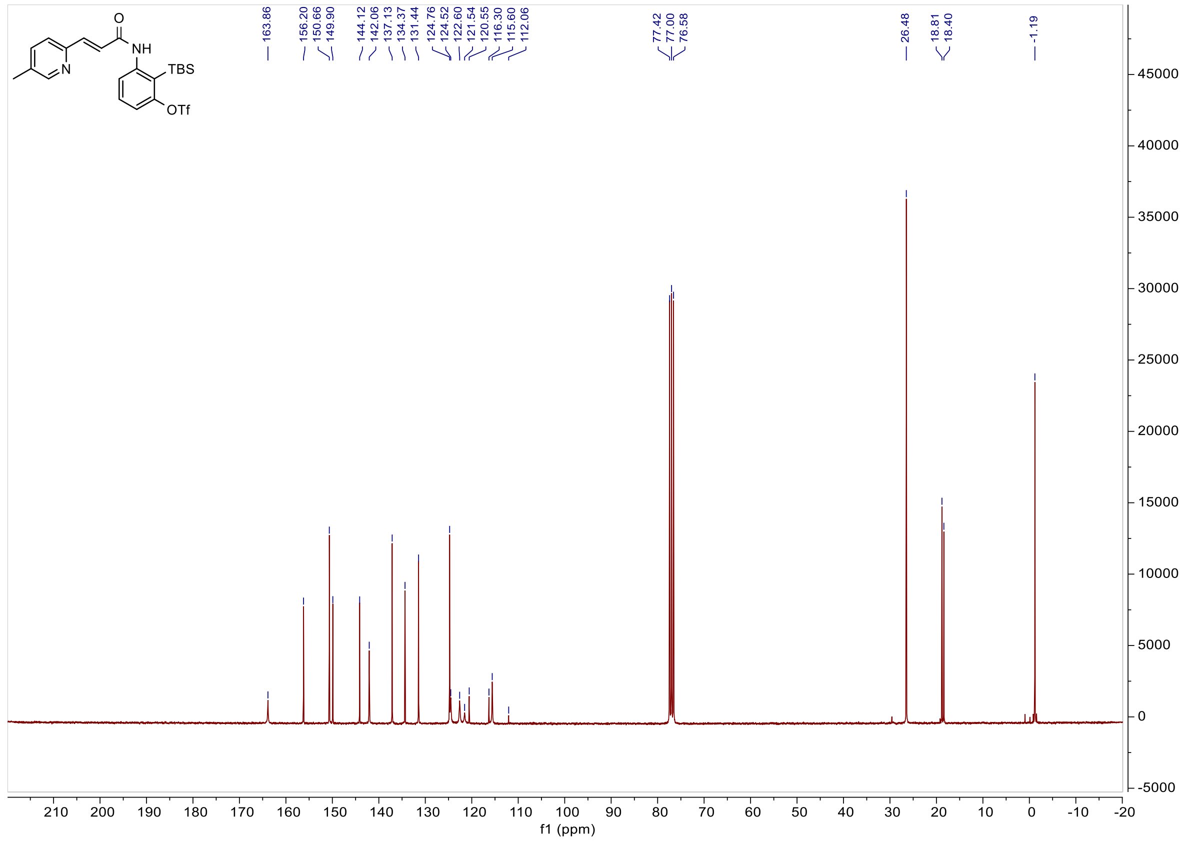

Figure S1, **1l**, ^{1}H NMR-300 MHz in $CDCl_3$ and ^{13}C NMR-75 MHz in $CDCl_3$

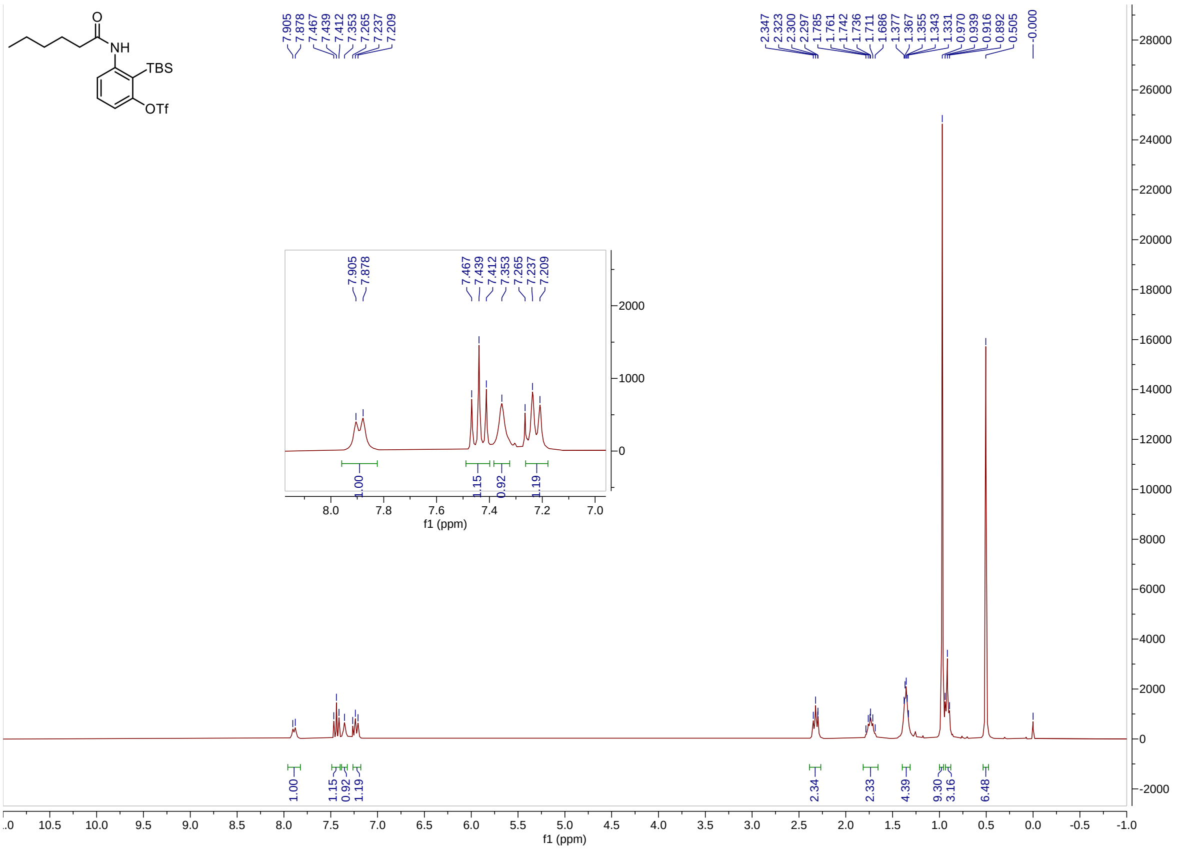

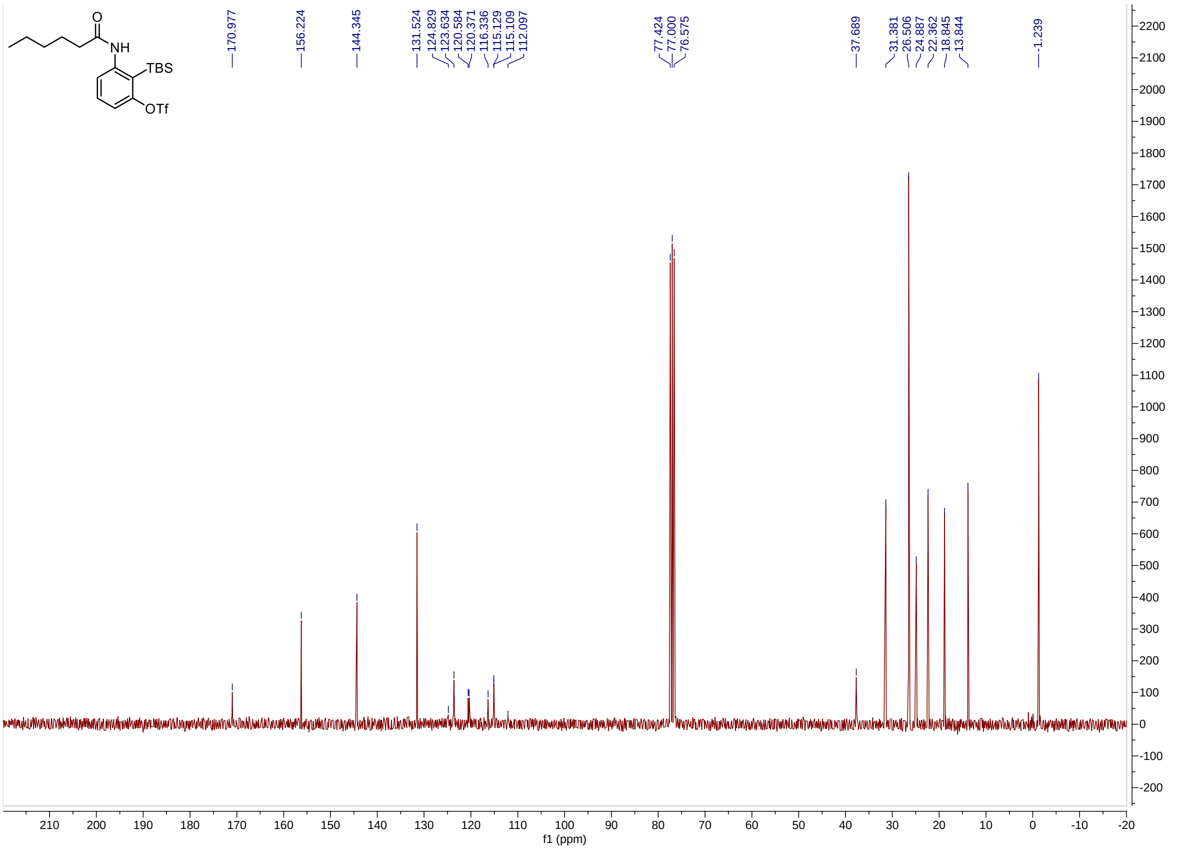

Figure S1, **1m**, ^{1}H NMR-300 MHz in $CDCl_3$ and ^{13}C NMR-75 MHz in $CDCl_3$

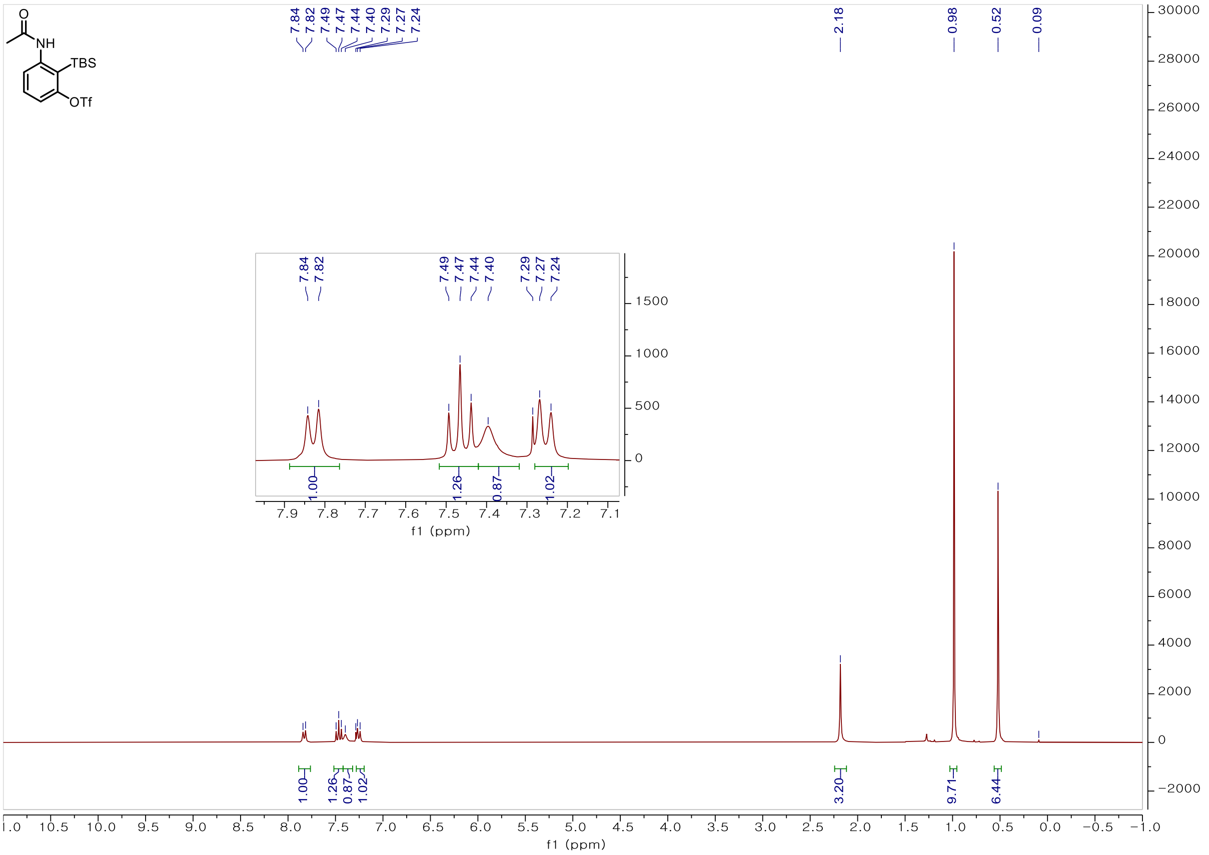

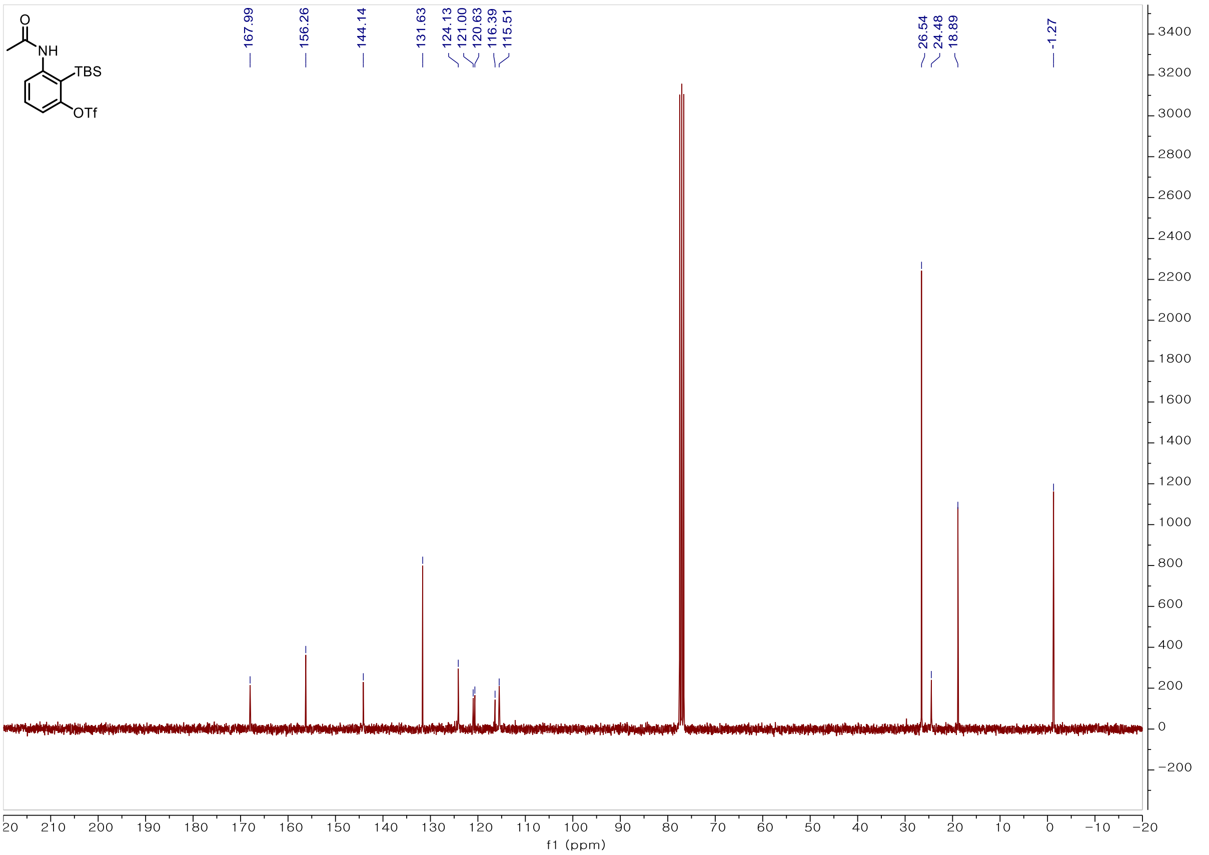

Figure S1, **1n**, ^{1}H NMR-300 MHz in $CDCl_3$ and ^{13}C NMR-75 MHz in $CDCl_3$

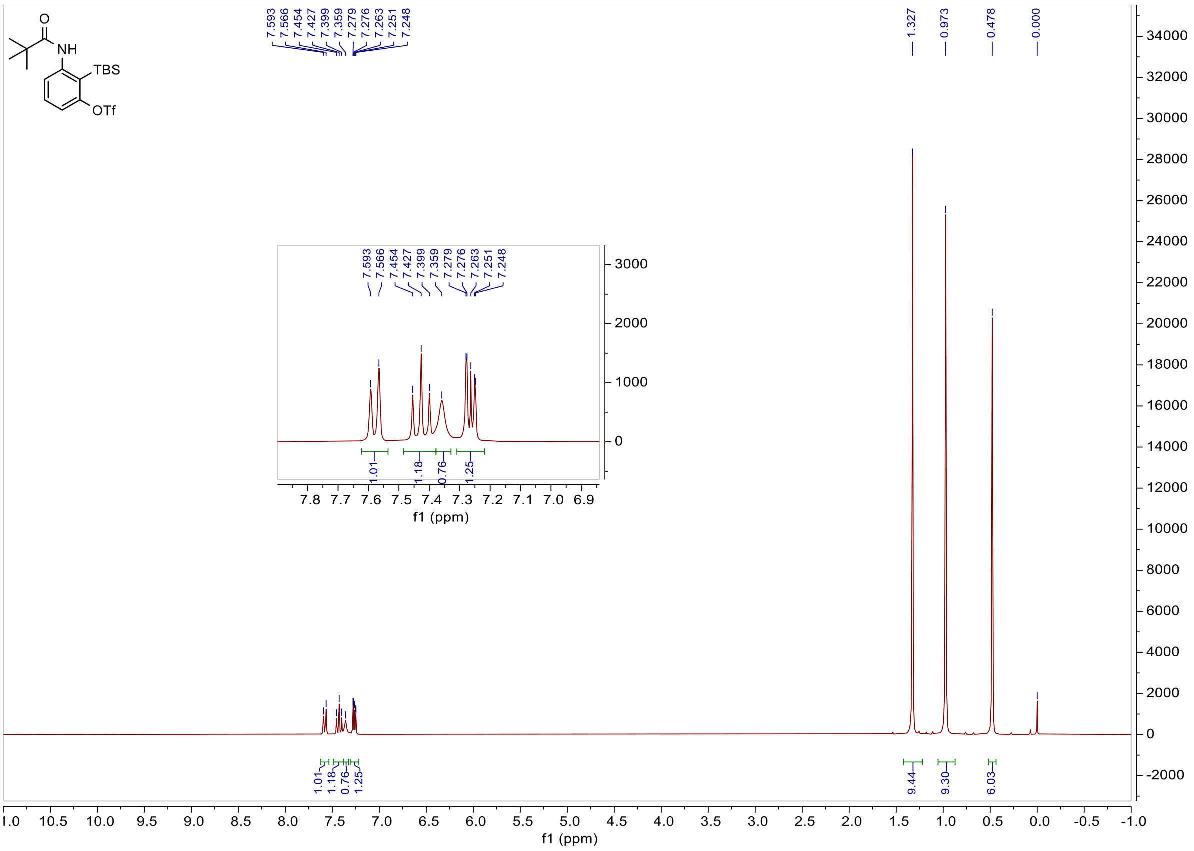

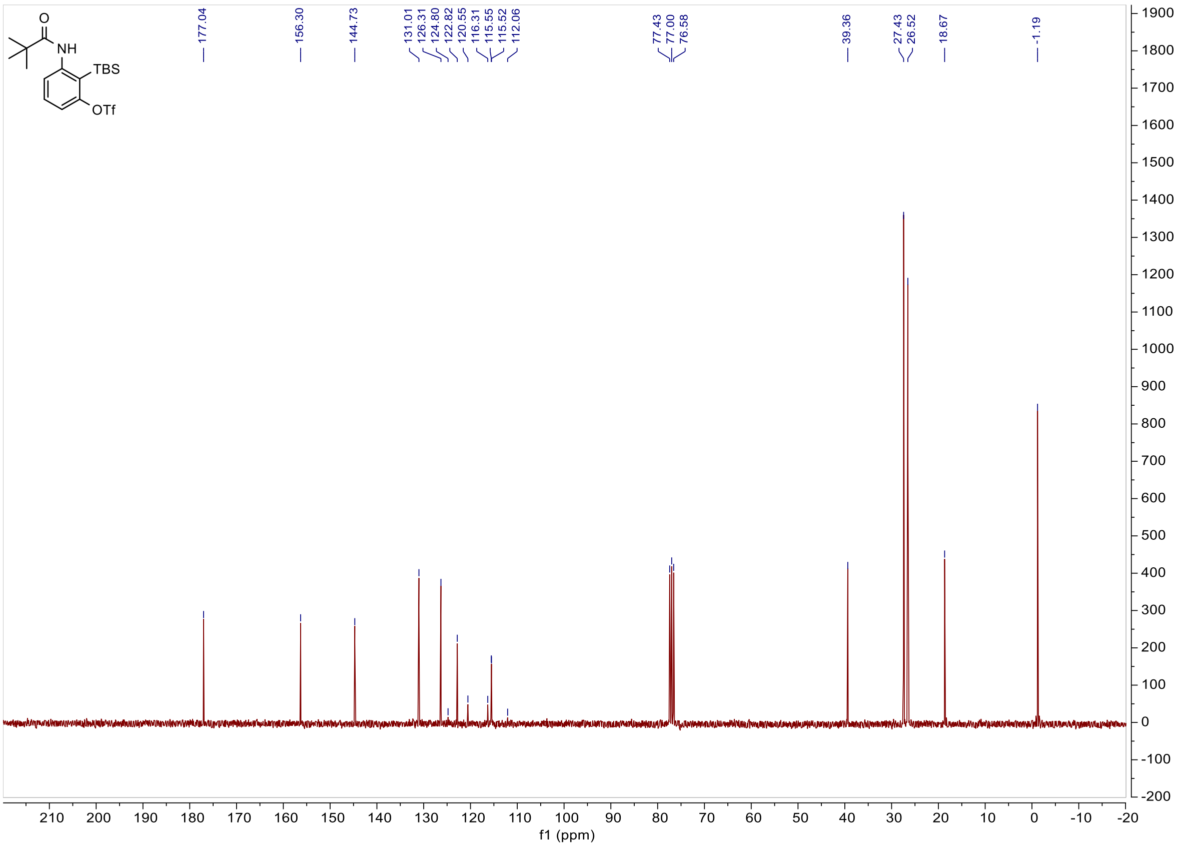

Figure S1, **1o**, ^{1}H NMR-300 MHz in $CDCl_3$ and ^{13}C NMR-75 MHz in $CDCl_3$

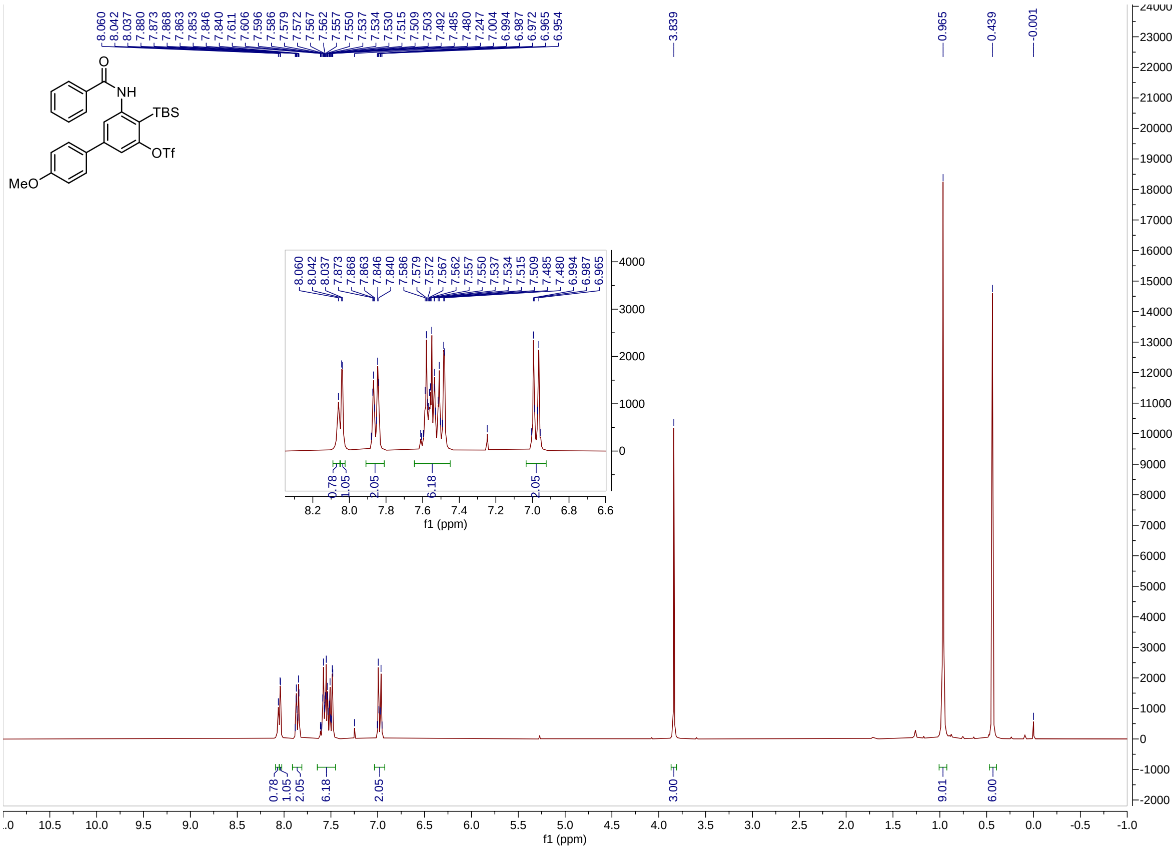

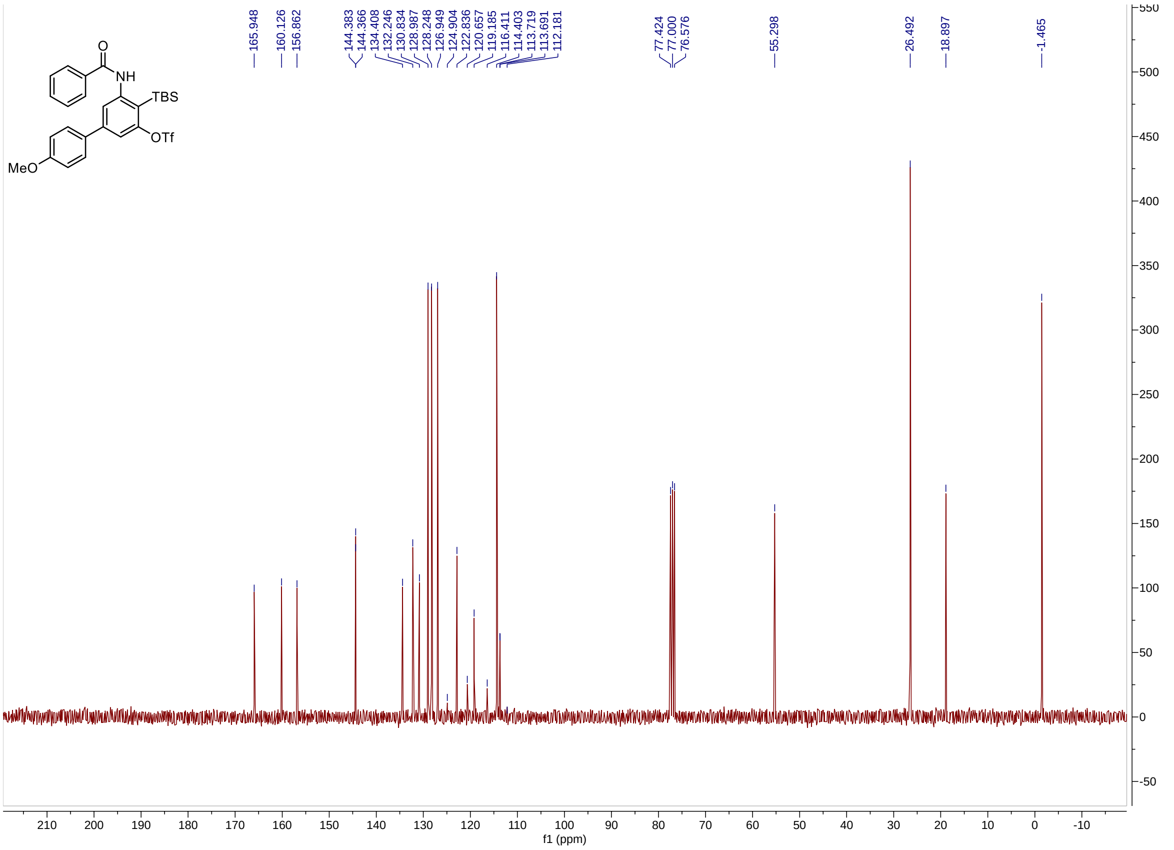

Figure S1, **1p**, ^{1}H NMR-300 MHz in $CDCl_3$ and ^{13}C NMR-75 MHz in $CDCl_3$

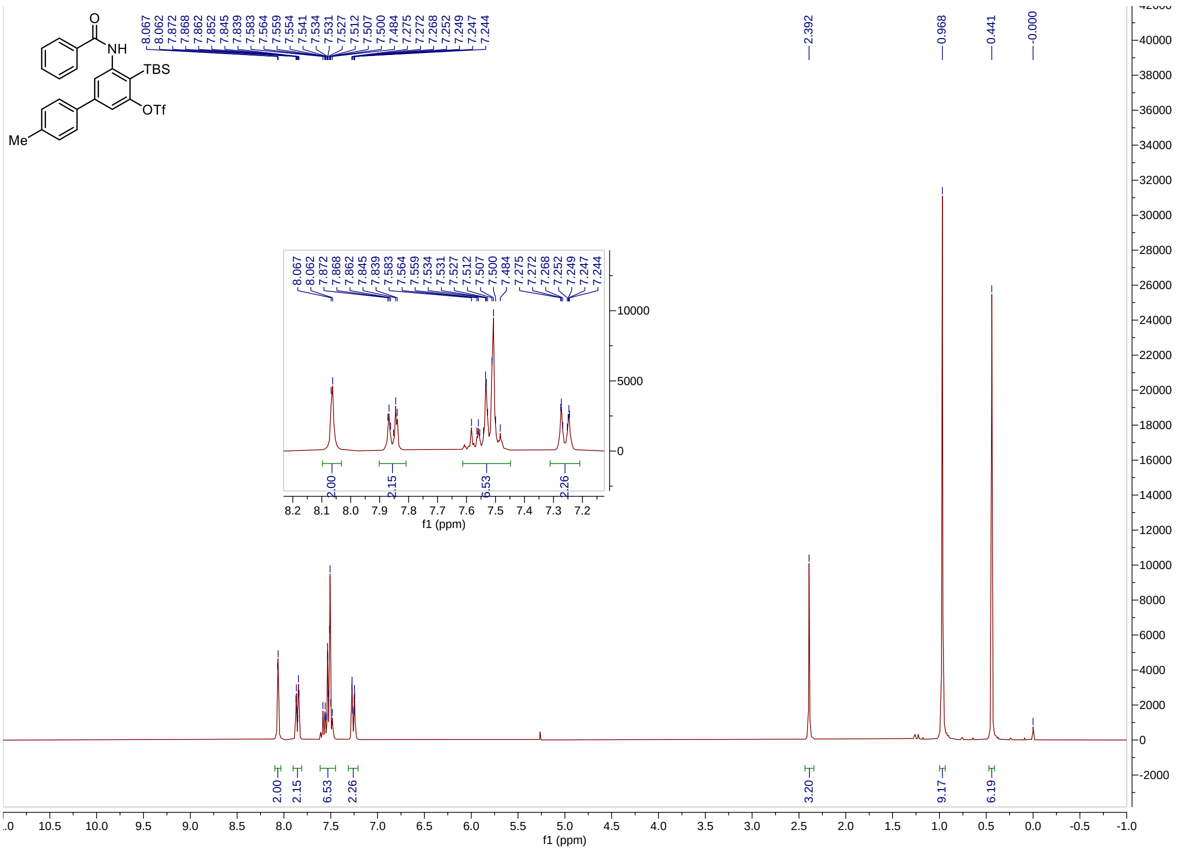

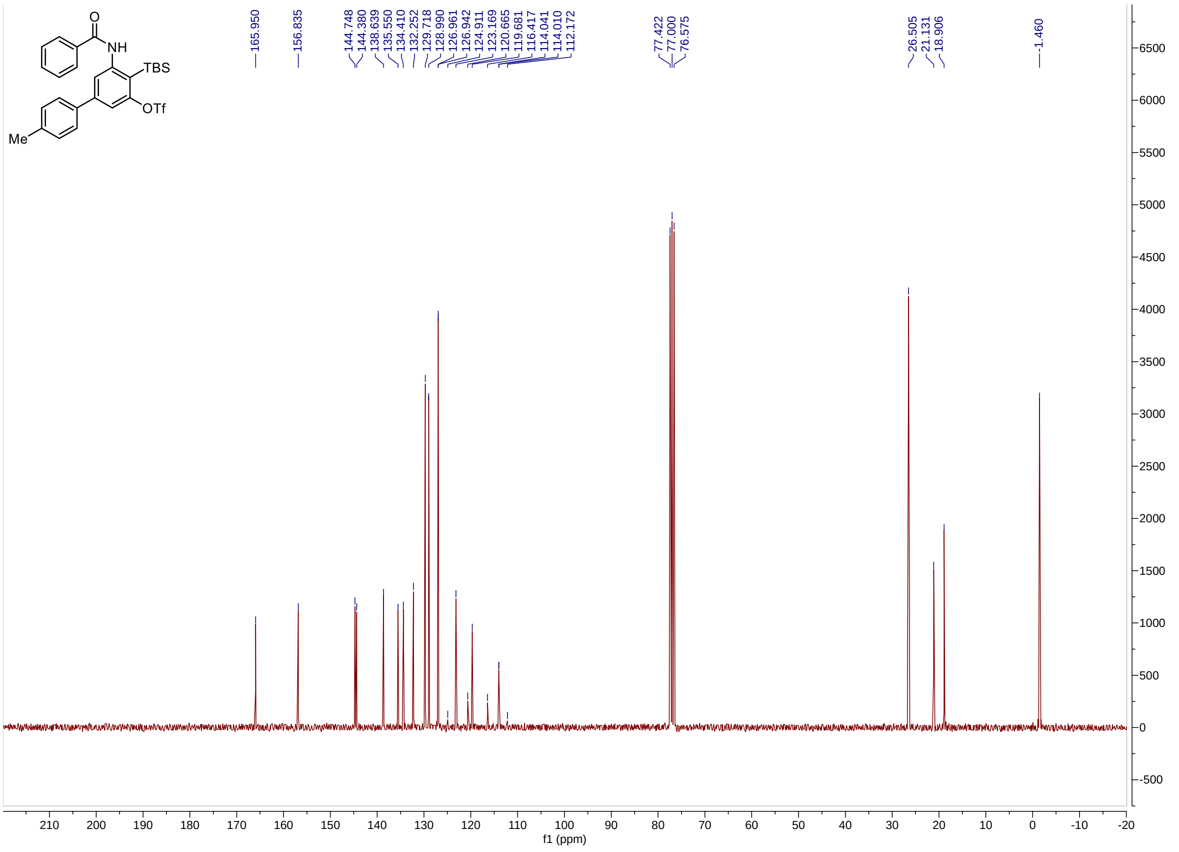

Figure S1, **1q**, ^{1}H NMR-300 MHz in $CDCl_3$ and ^{13}C NMR-75 MHz in $CDCl_3$

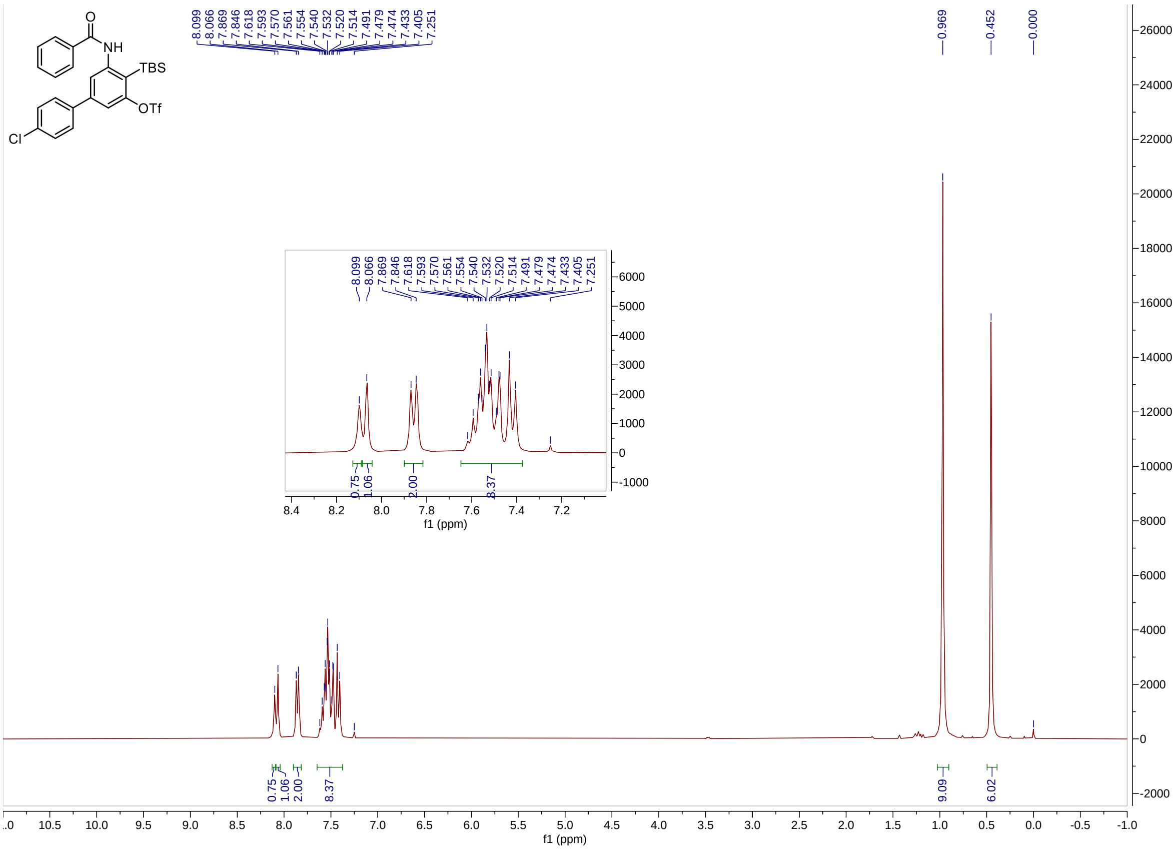

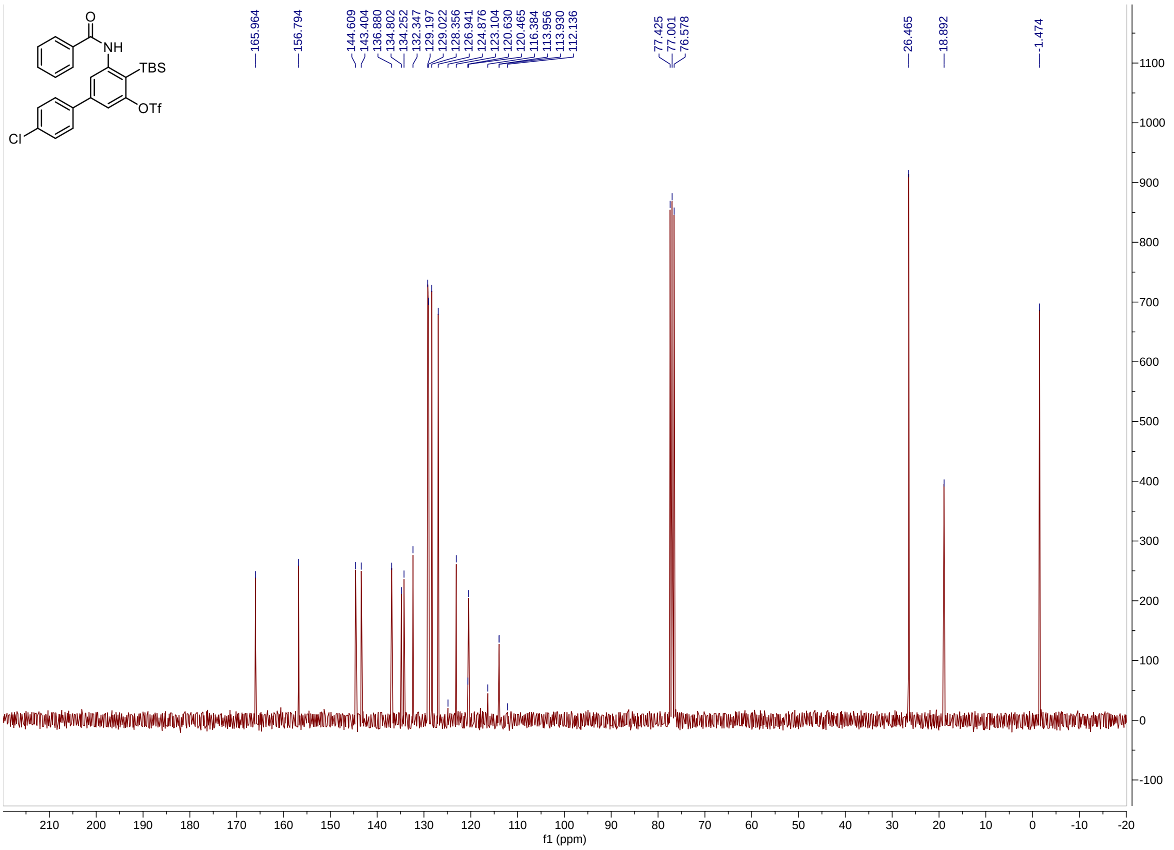

3a, ^{1}H NMR-300 MHz in $CDCl_3$ and ^{13}C NMR-75 MHz in $CDCl_3$

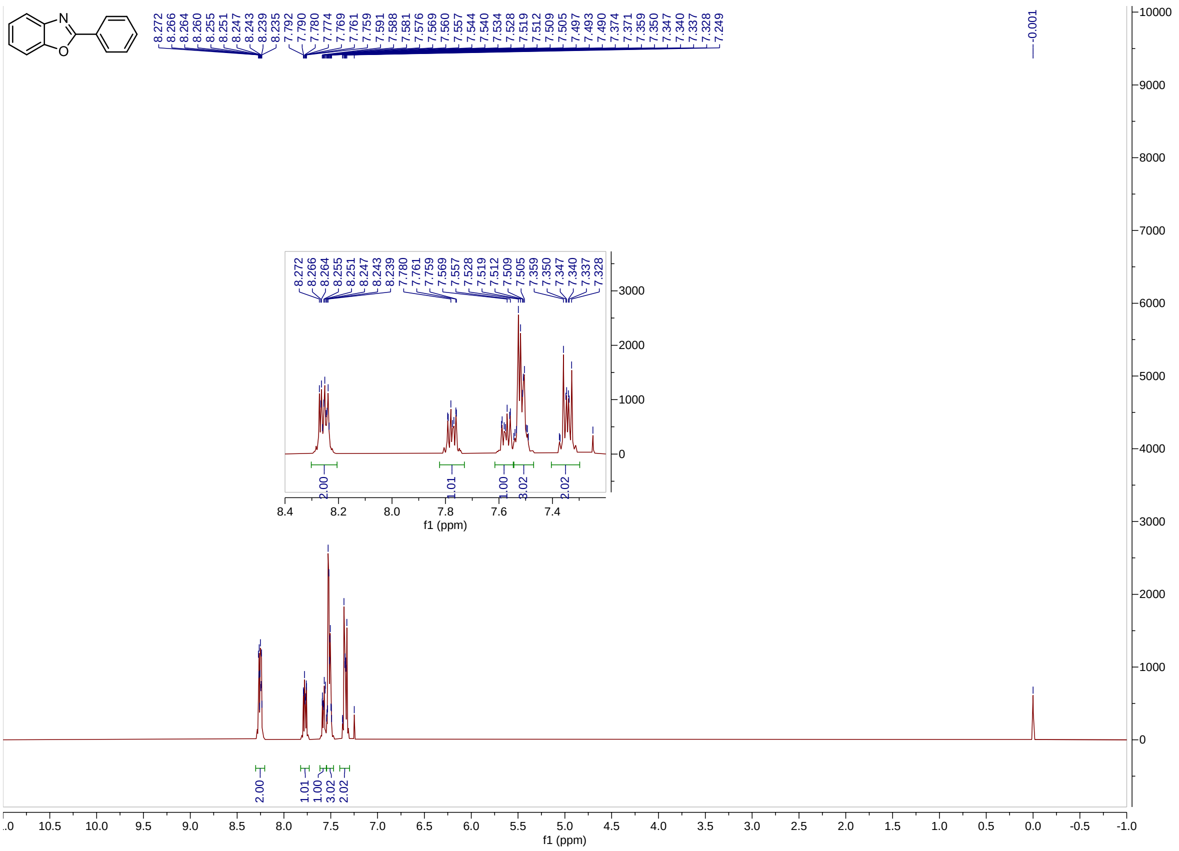

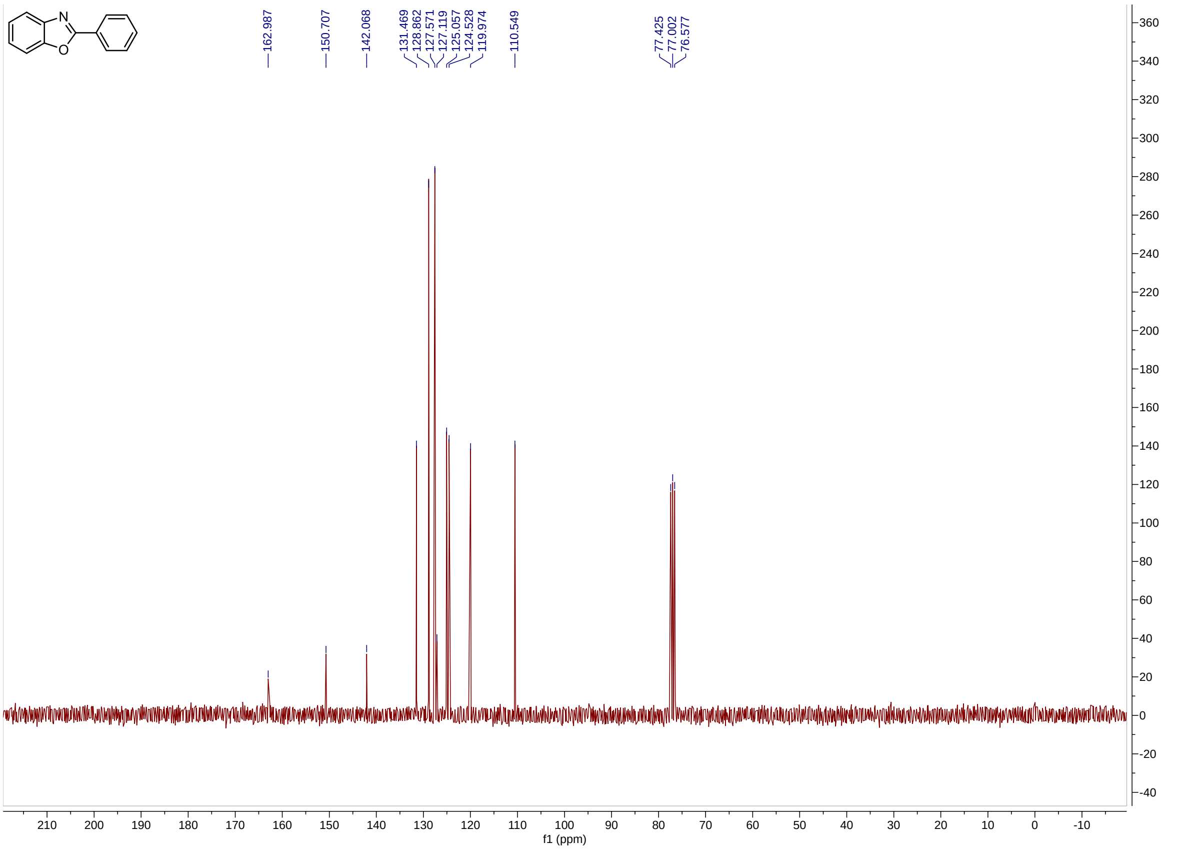

3b, ^{1}H NMR-300 MHz in $CDCl_3$ and ^{13}C NMR-75 MHz in $CDCl_3$

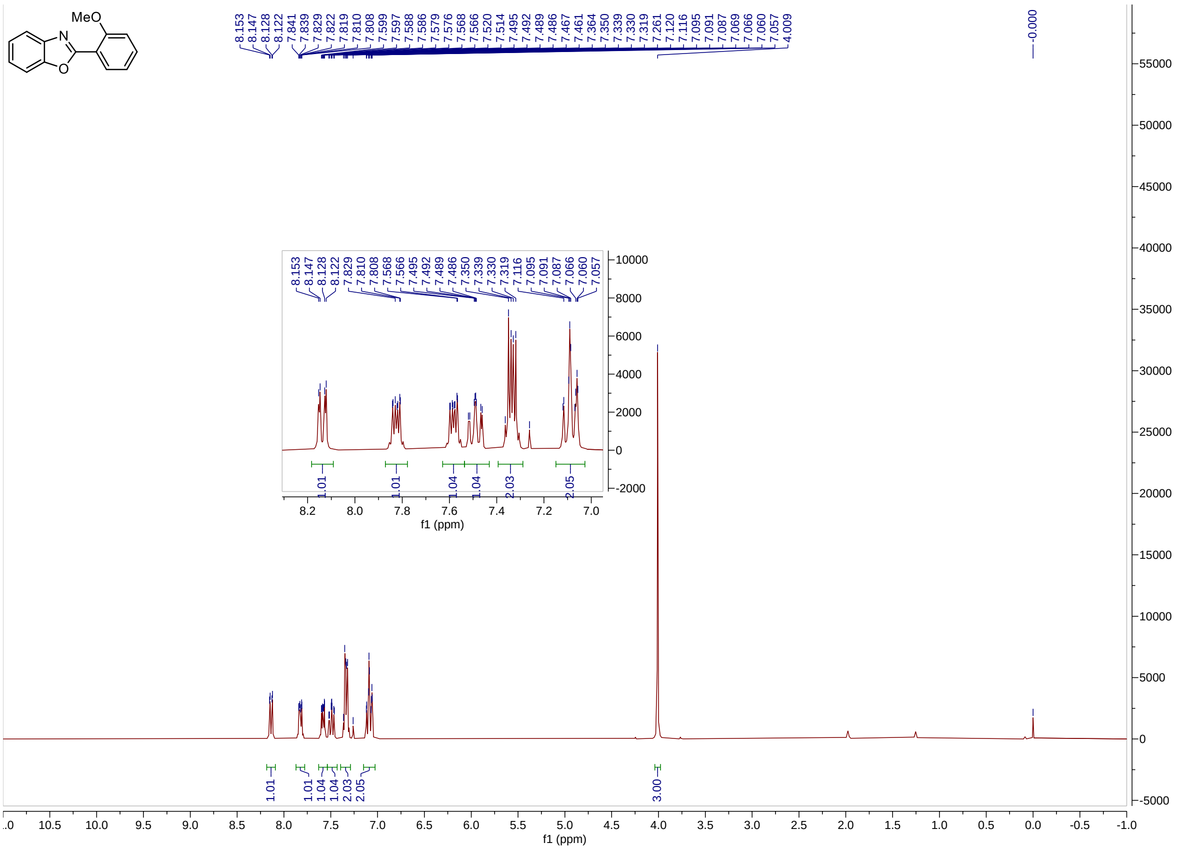

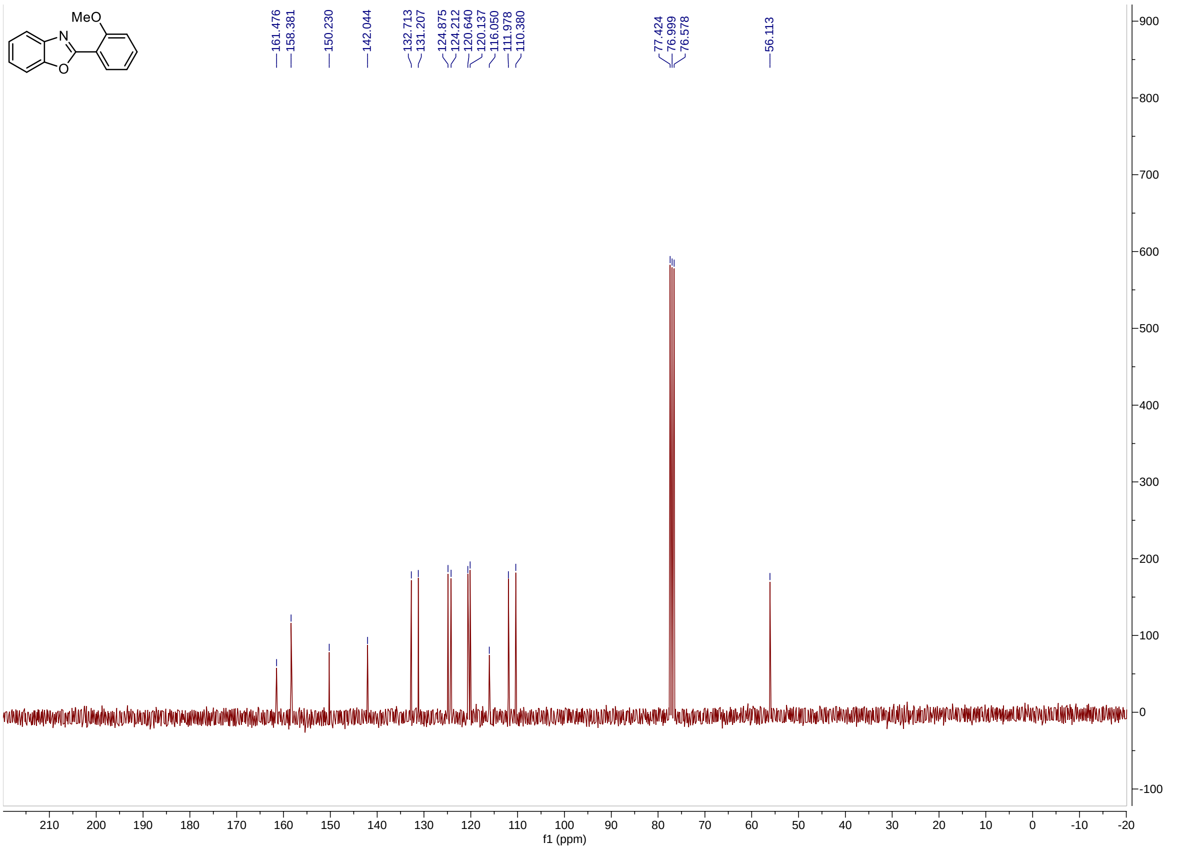

3c, ^{1}H NMR-300 MHz in $CDCl_3$ and ^{13}C NMR-75 MHz in $CDCl_3$

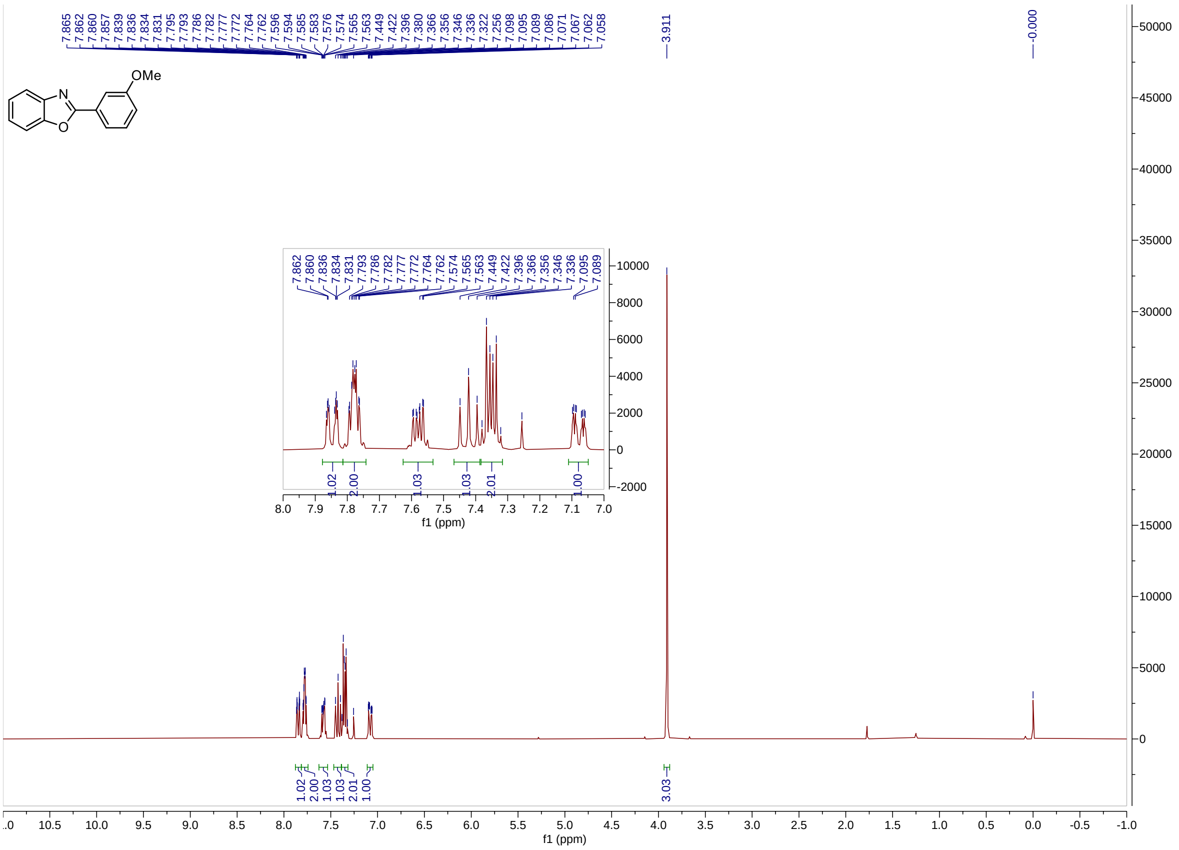

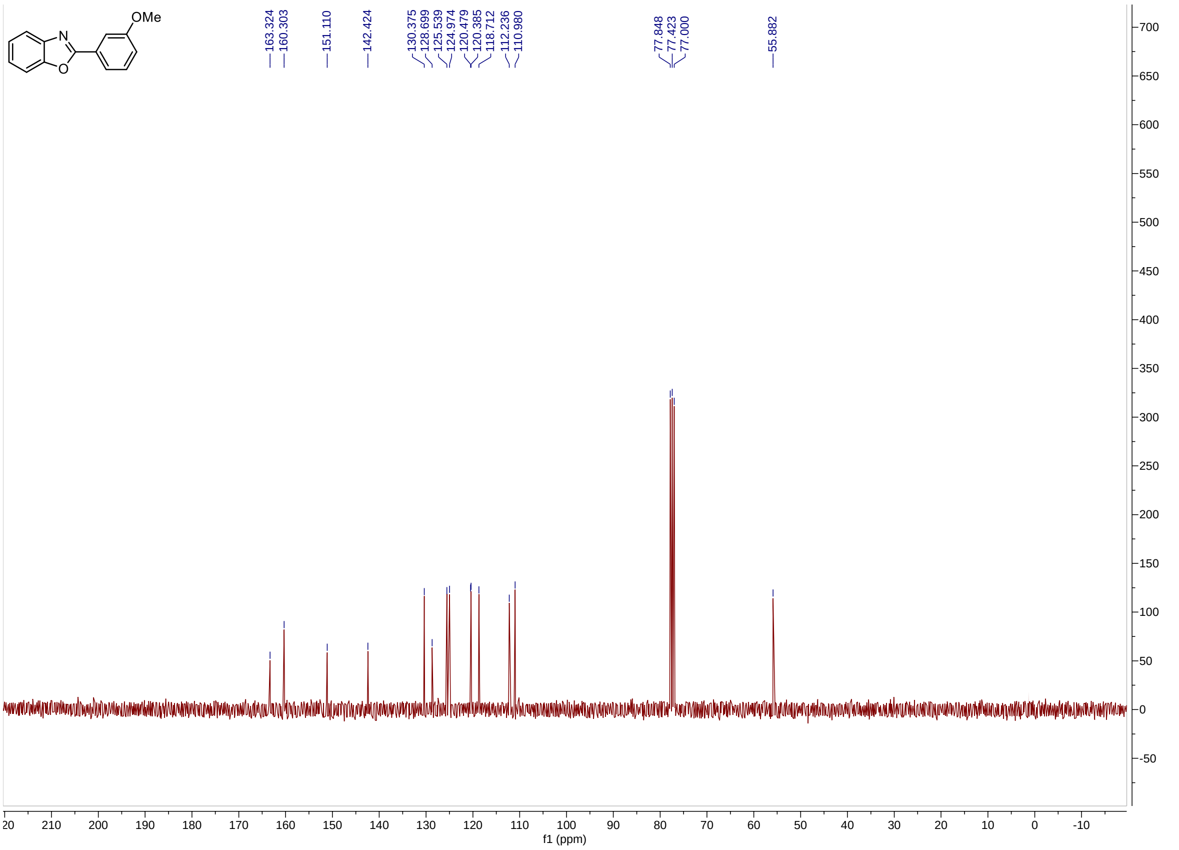

3d, ^{1}H NMR-300 MHz in $CDCl_3$ and ^{13}C NMR-75 MHz in $CDCl_3$

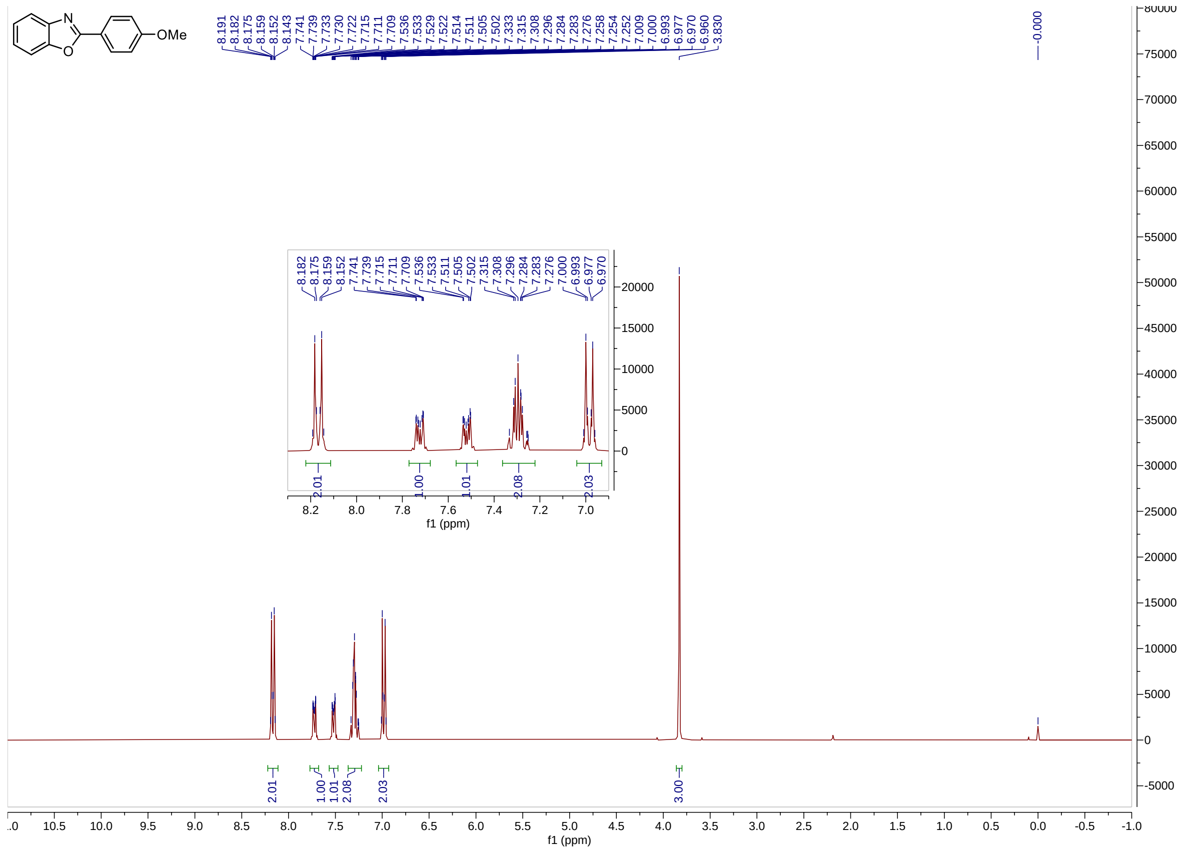

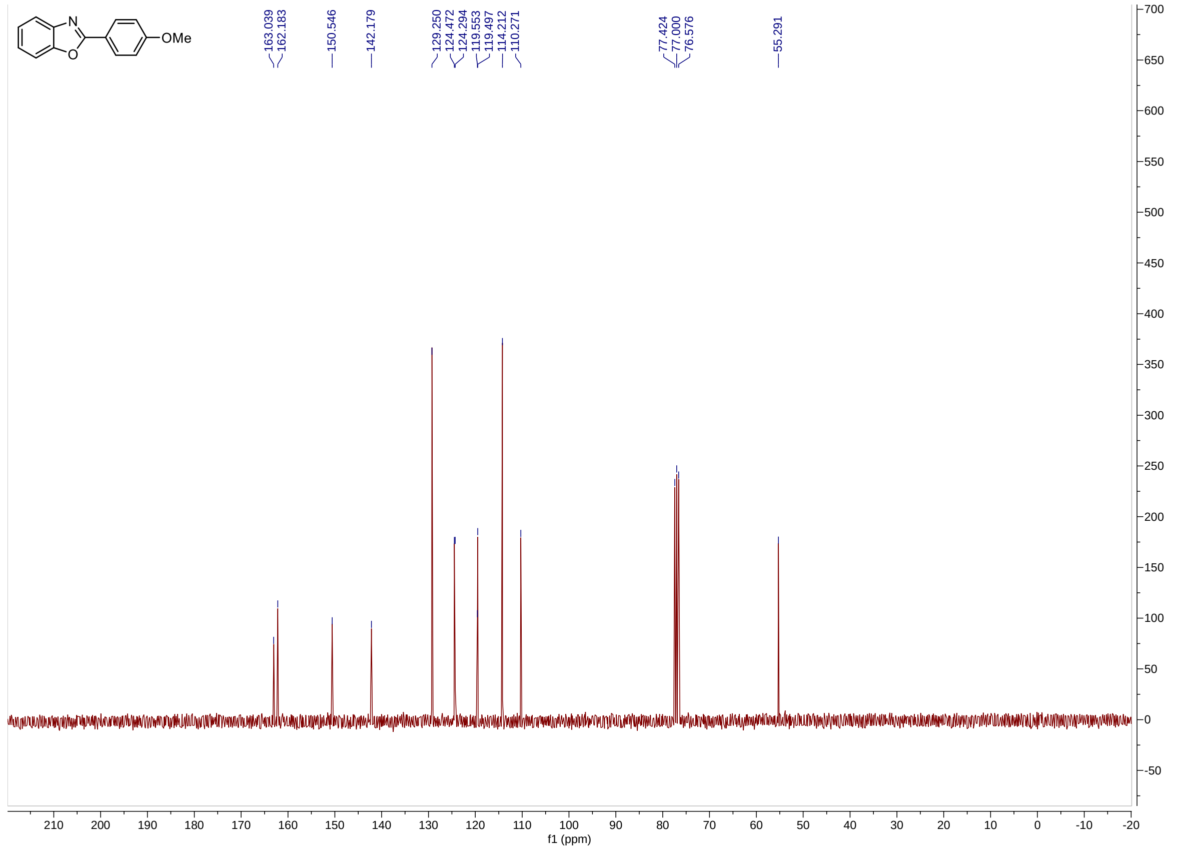

3e, ^{1}H NMR-300 MHz in $CDCl_3$ and ^{13}C NMR-75 MHz in $CDCl_3$

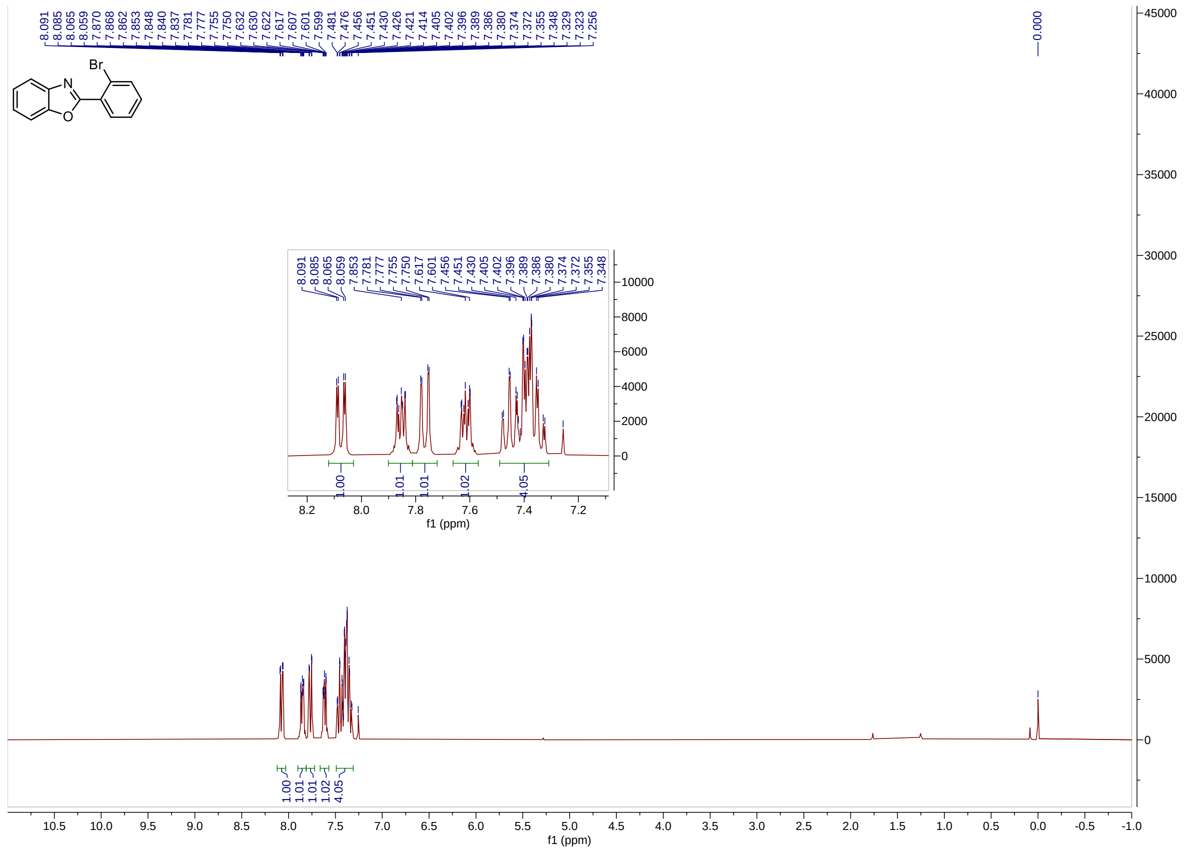

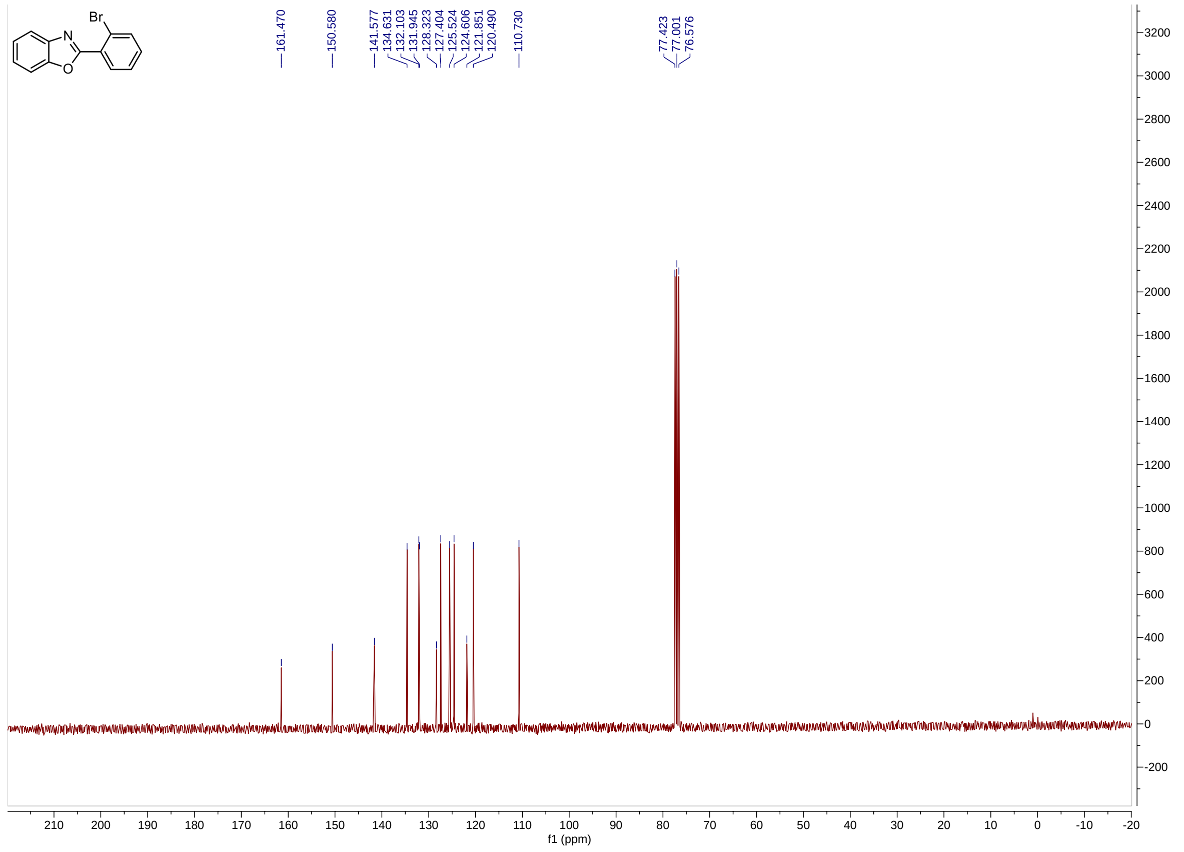

3f, ^{1}H NMR-300 MHz in $CDCl_3$ and ^{13}C NMR-75 MHz in $CDCl_3$

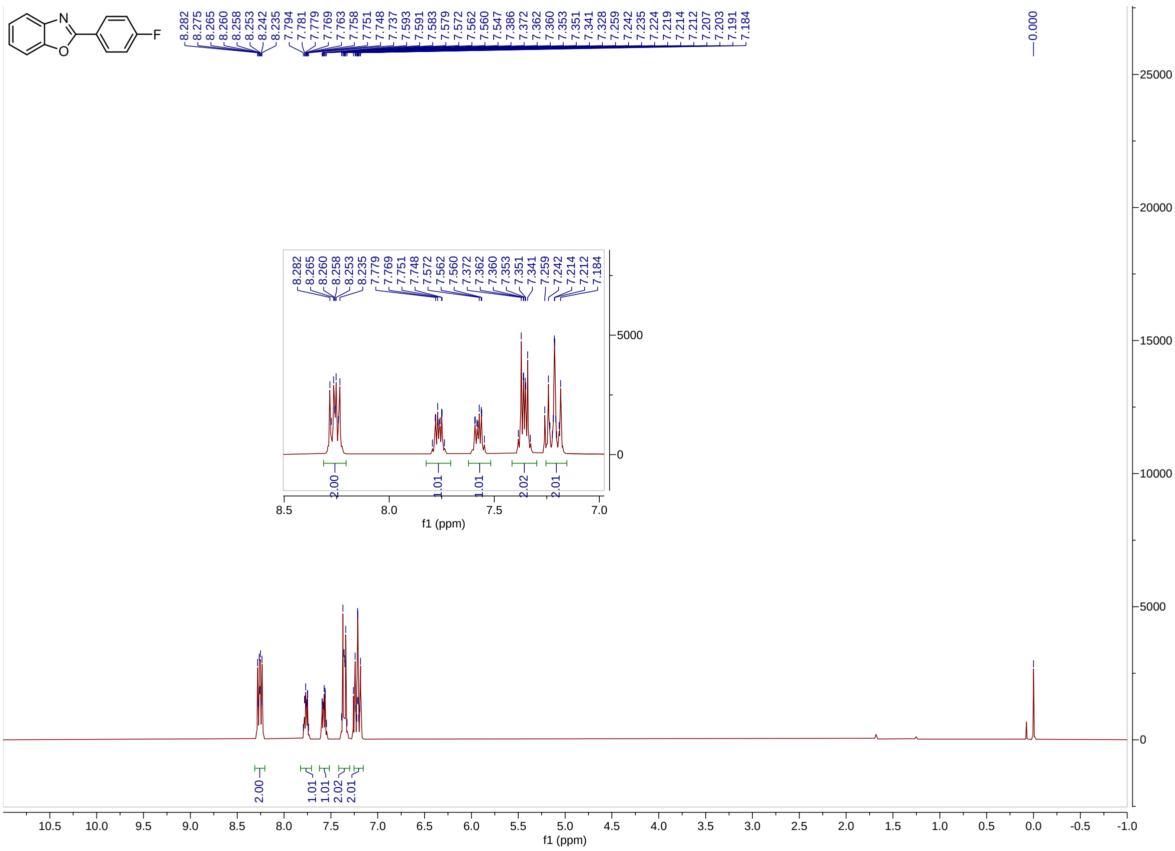

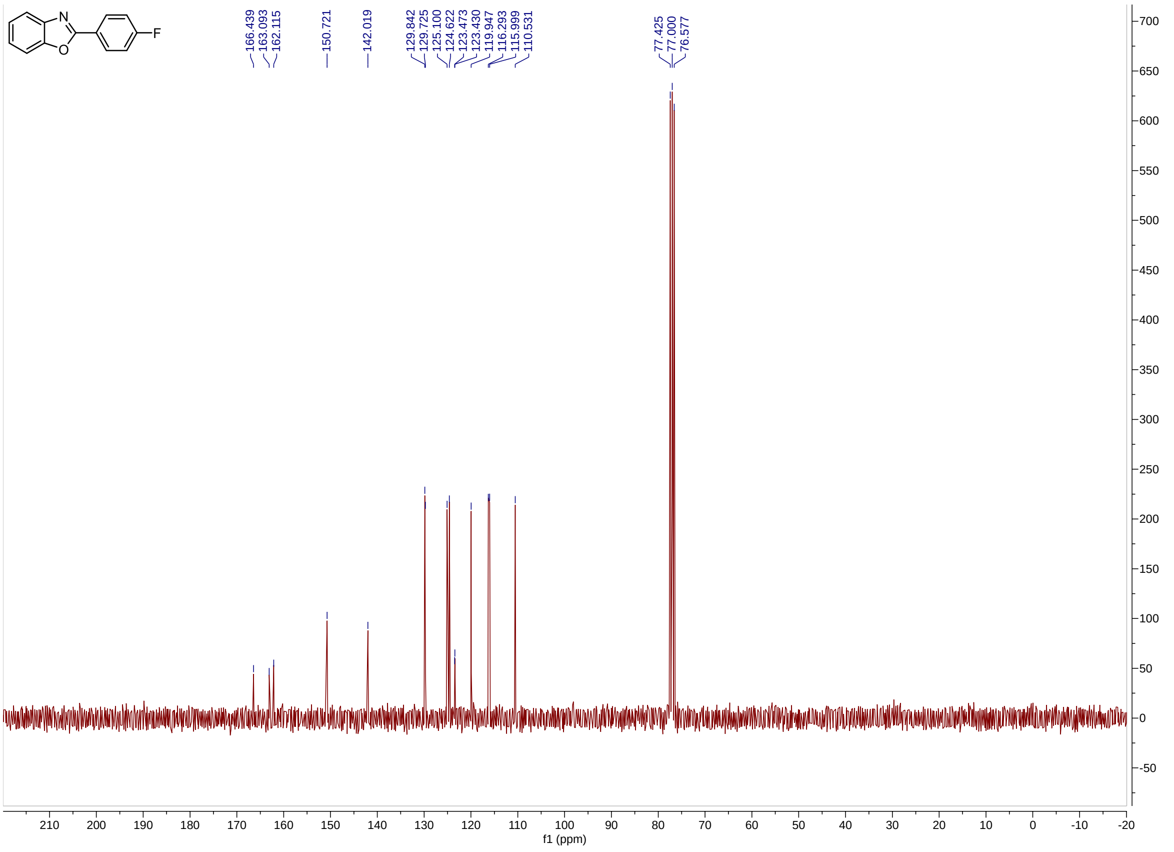

3g, ^{1}H NMR-300 MHz in $CDCl_3$ and ^{13}C NMR-75 MHz in $CDCl_3$

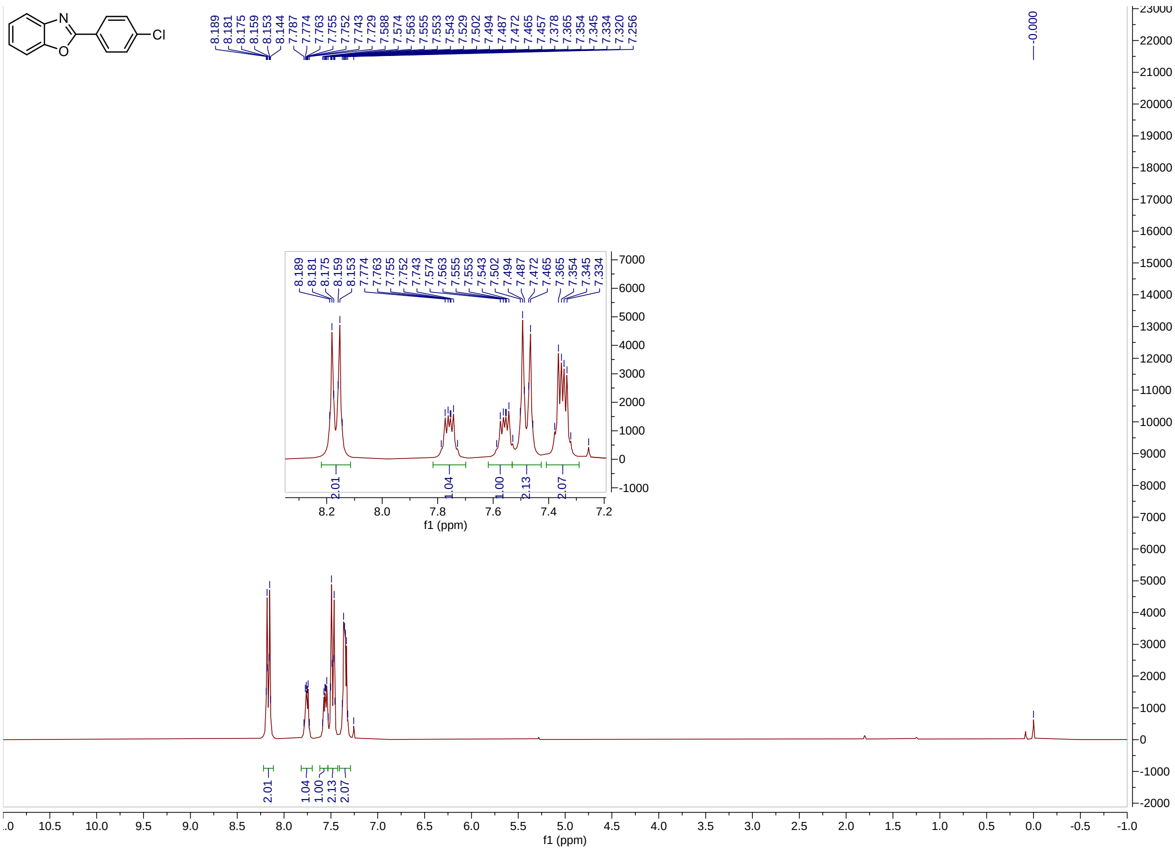

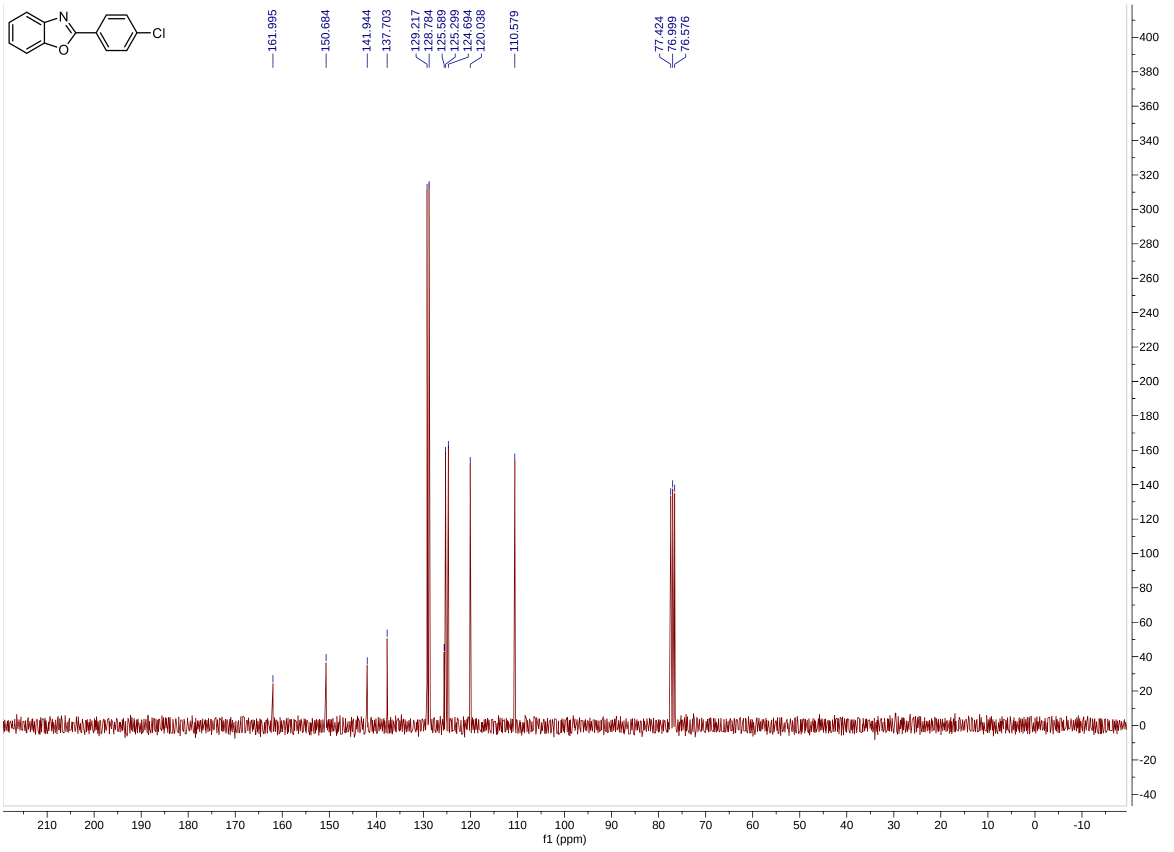

3h, ^{1}H NMR-300 MHz in $CDCl_3$ and ^{13}C NMR-75 MHz in $CDCl_3$

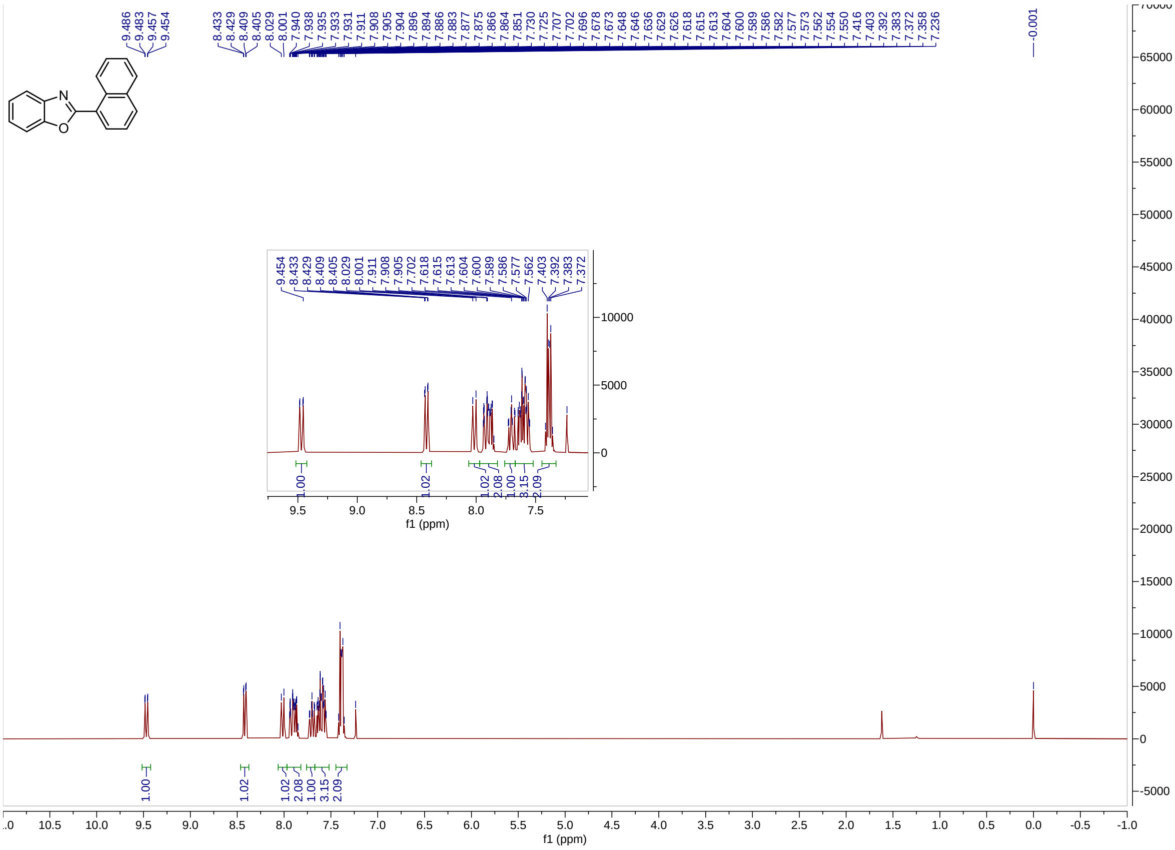

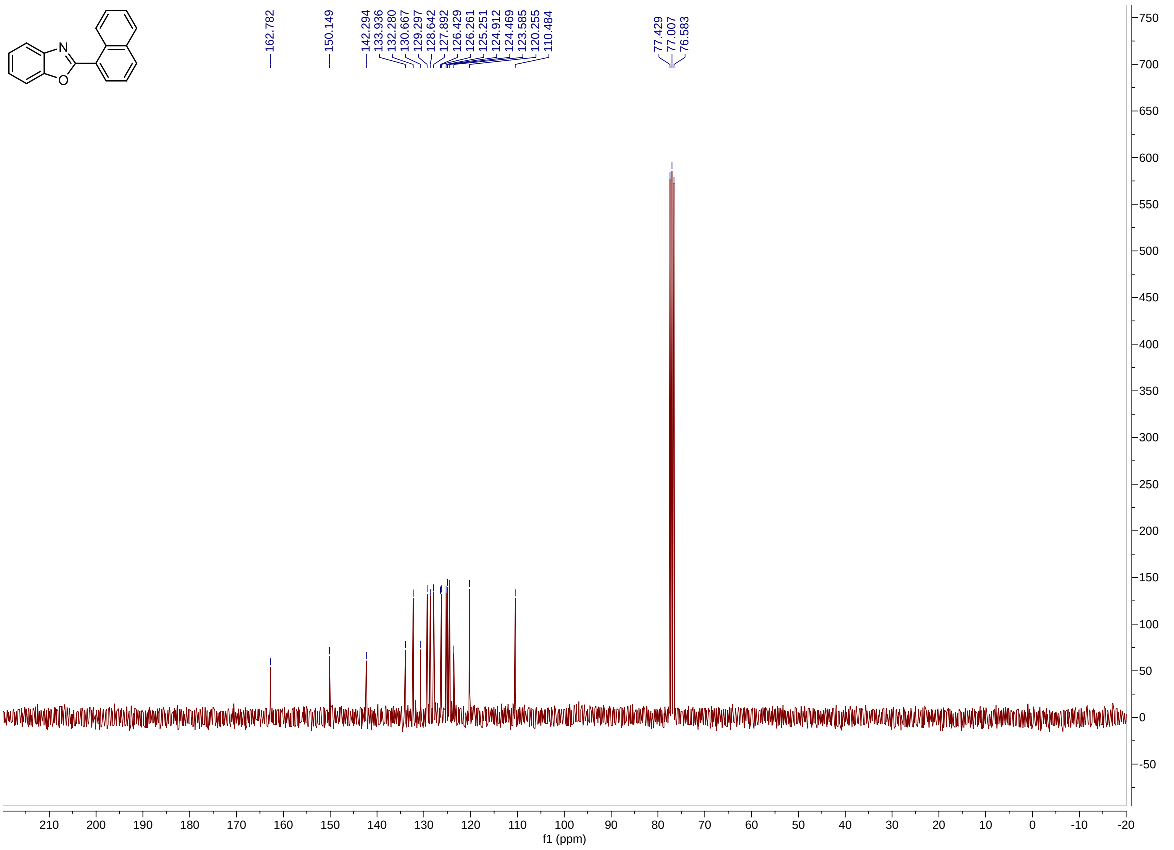

3i, ^{1}H NMR-300 MHz in $CDCl_3$ and ^{13}C NMR-75 MHz in $CDCl_3$

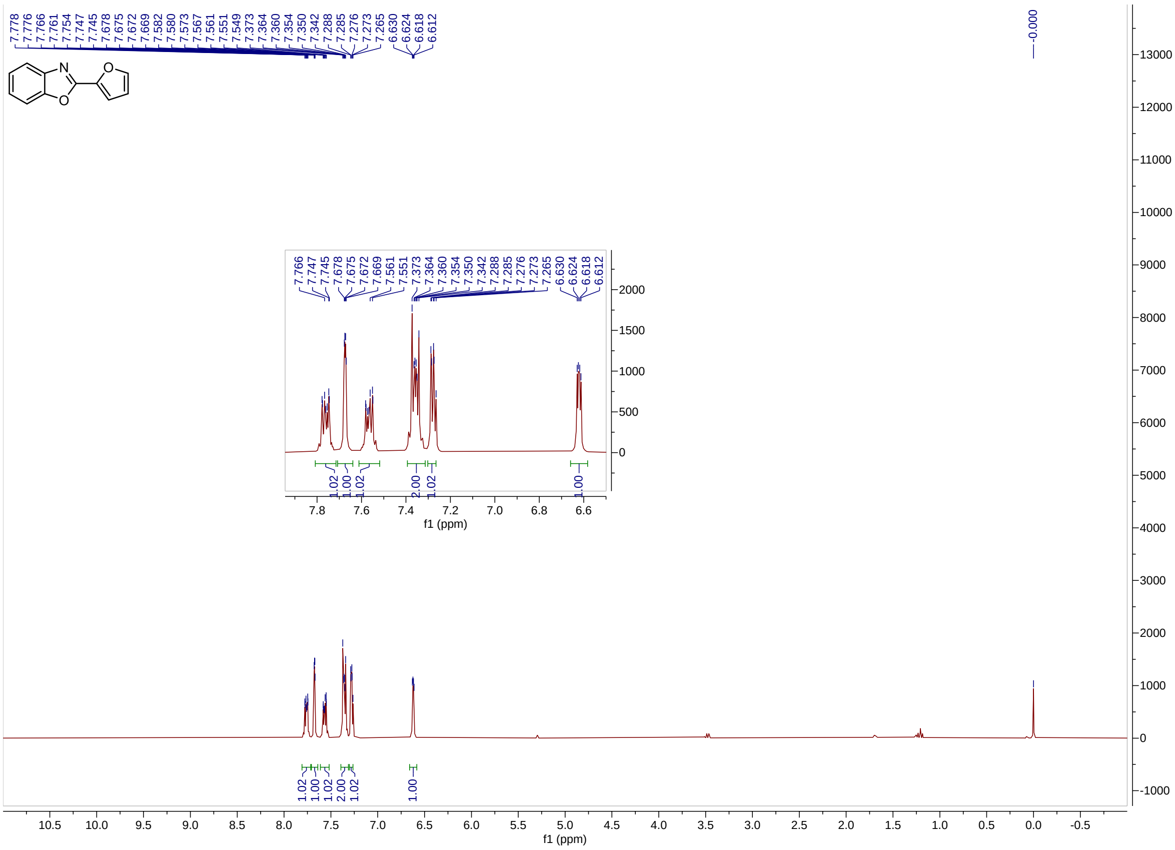

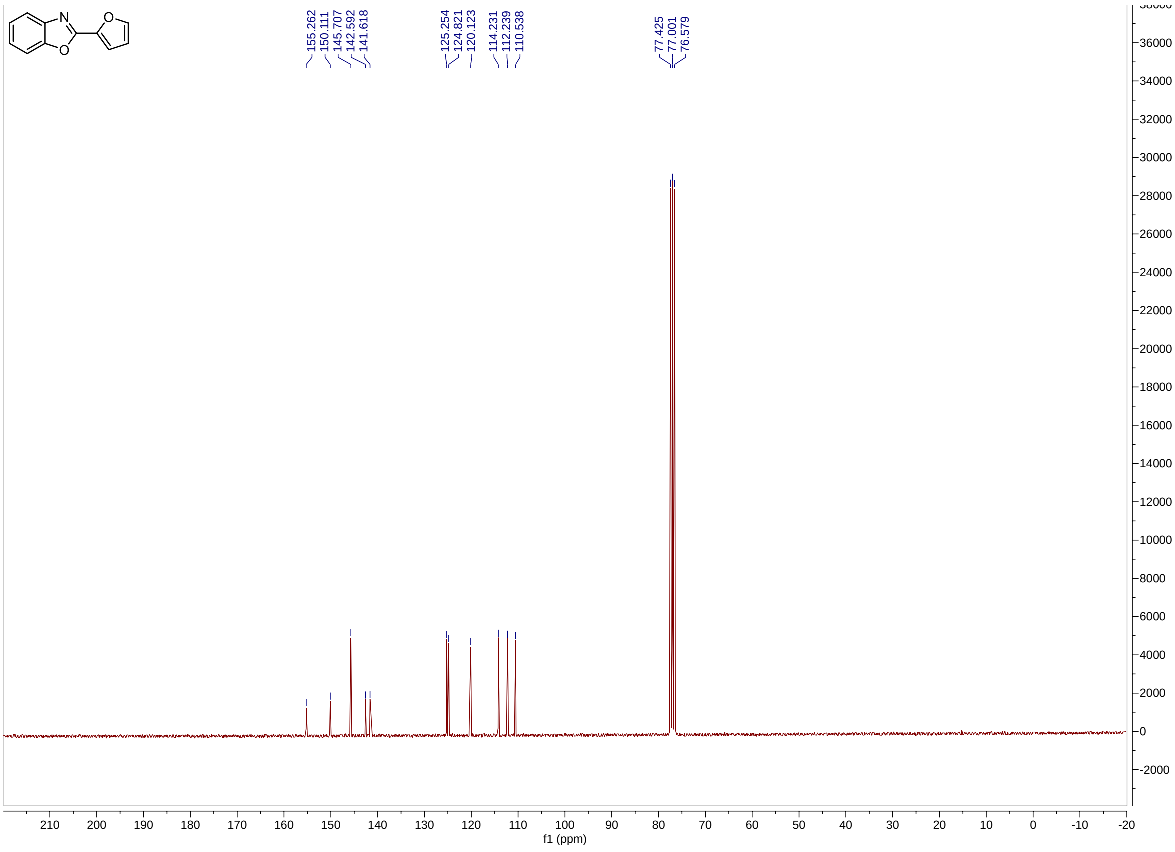

3j, ^{1}H NMR-300 MHz in $CDCl_3$ and ^{13}C NMR-75 MHz in $CDCl_3$

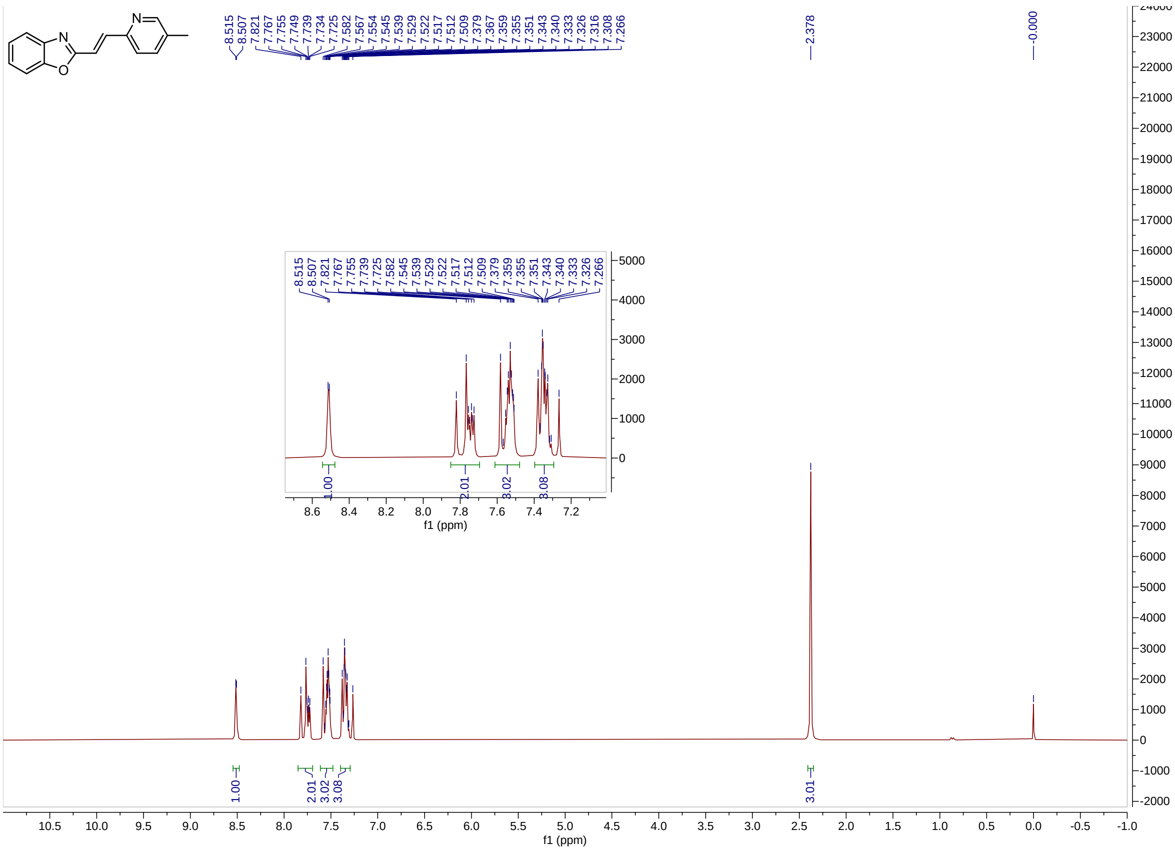

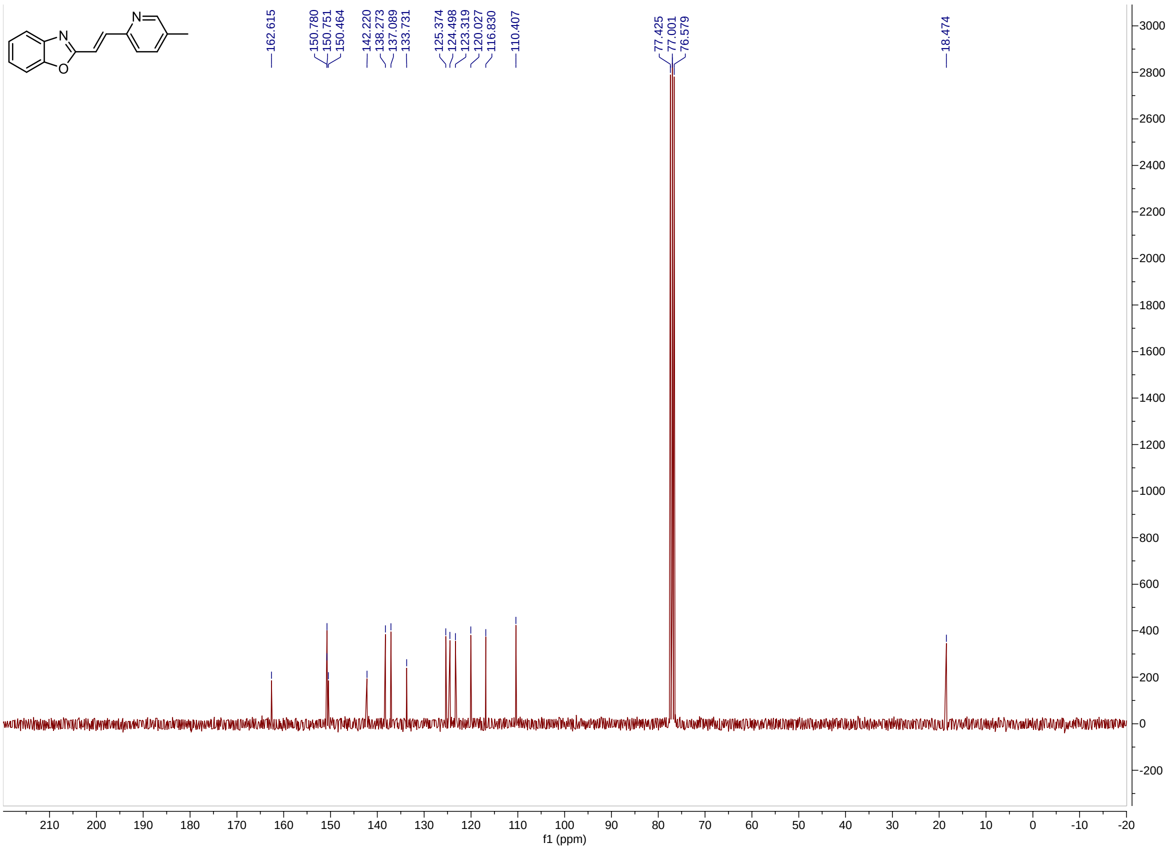

3k, ^{1}H NMR-300 MHz in $CDCl_3$ and ^{13}C NMR-75 MHz in $CDCl_3$

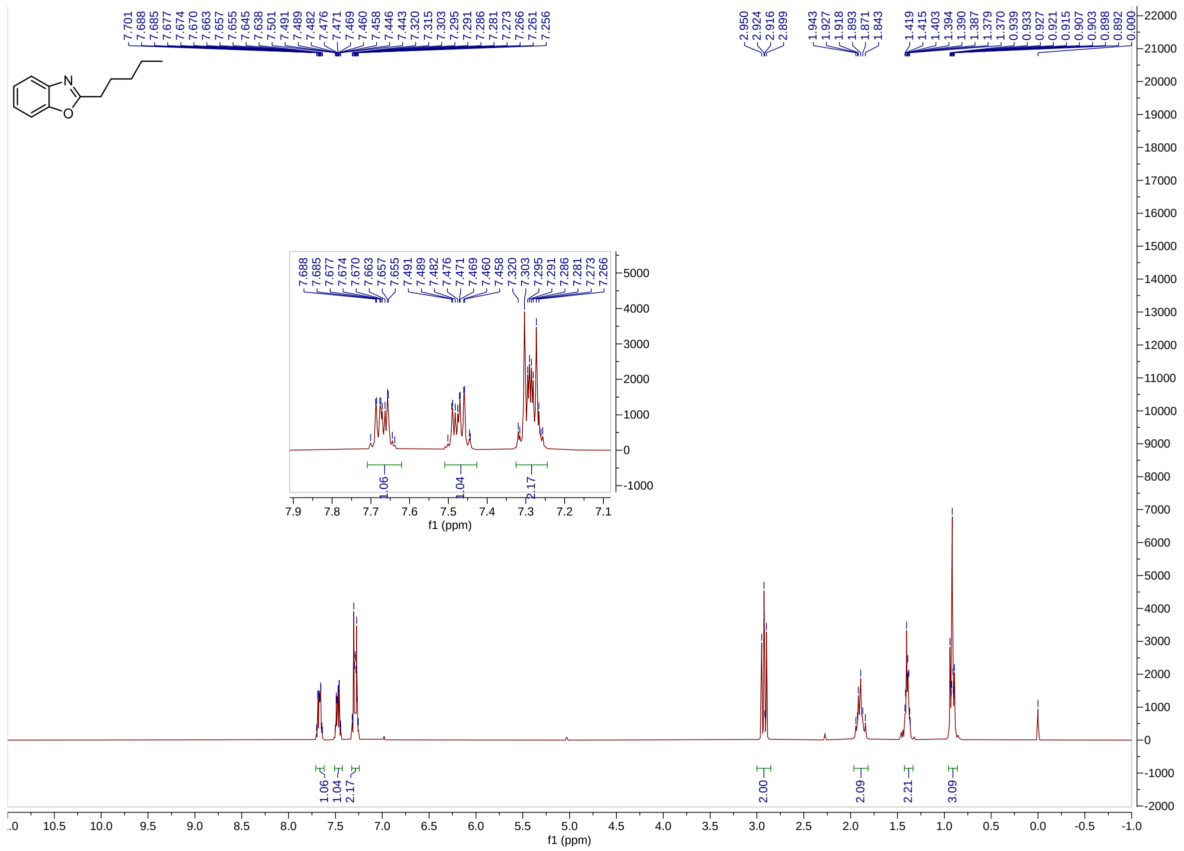

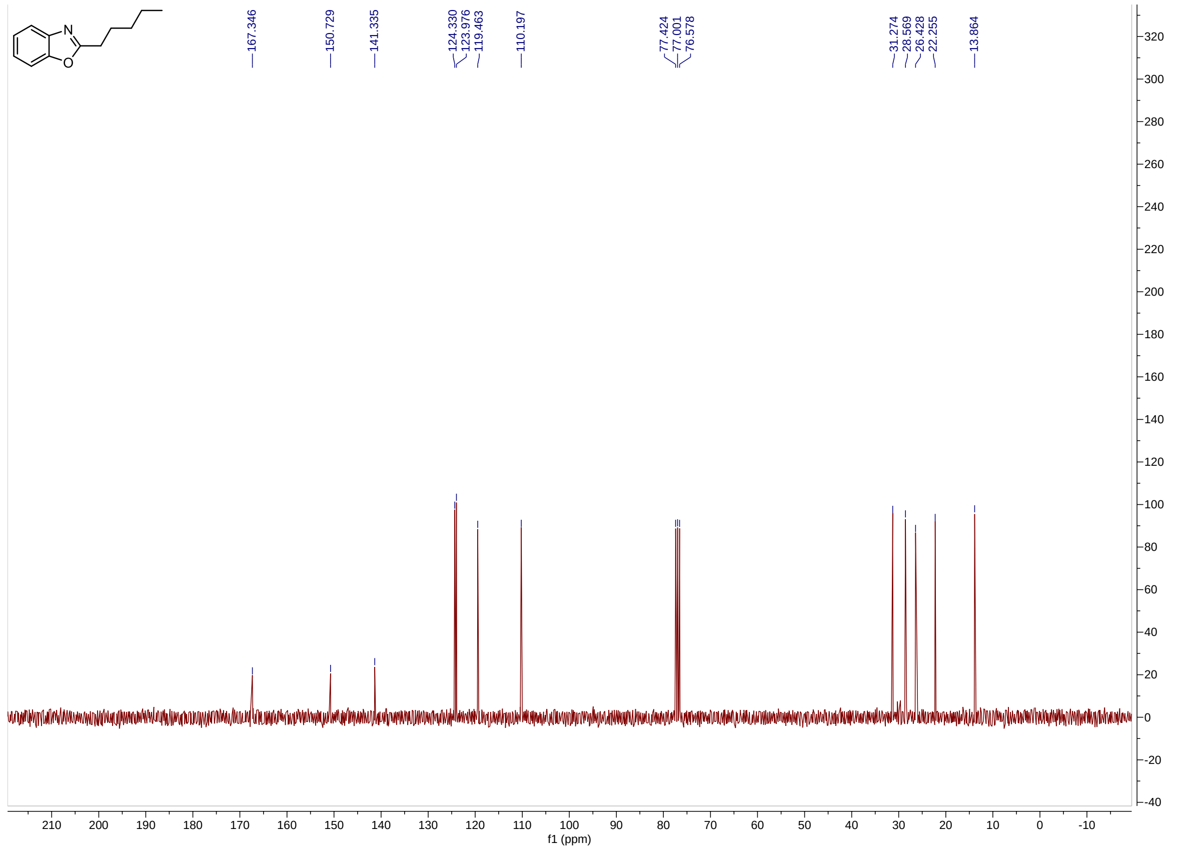

3l, ^{1}H NMR-300 MHz in $CDCl_3$ and ^{13}C NMR-75 MHz in $CDCl_3$

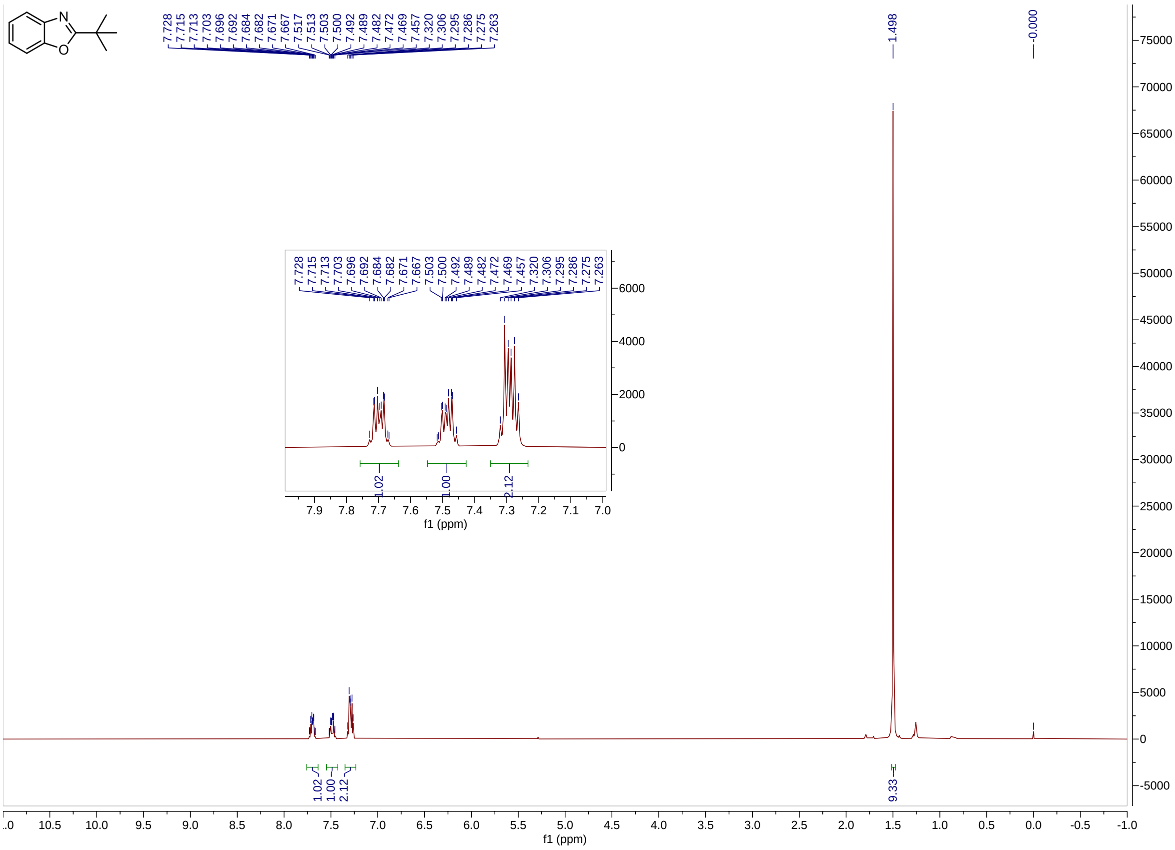

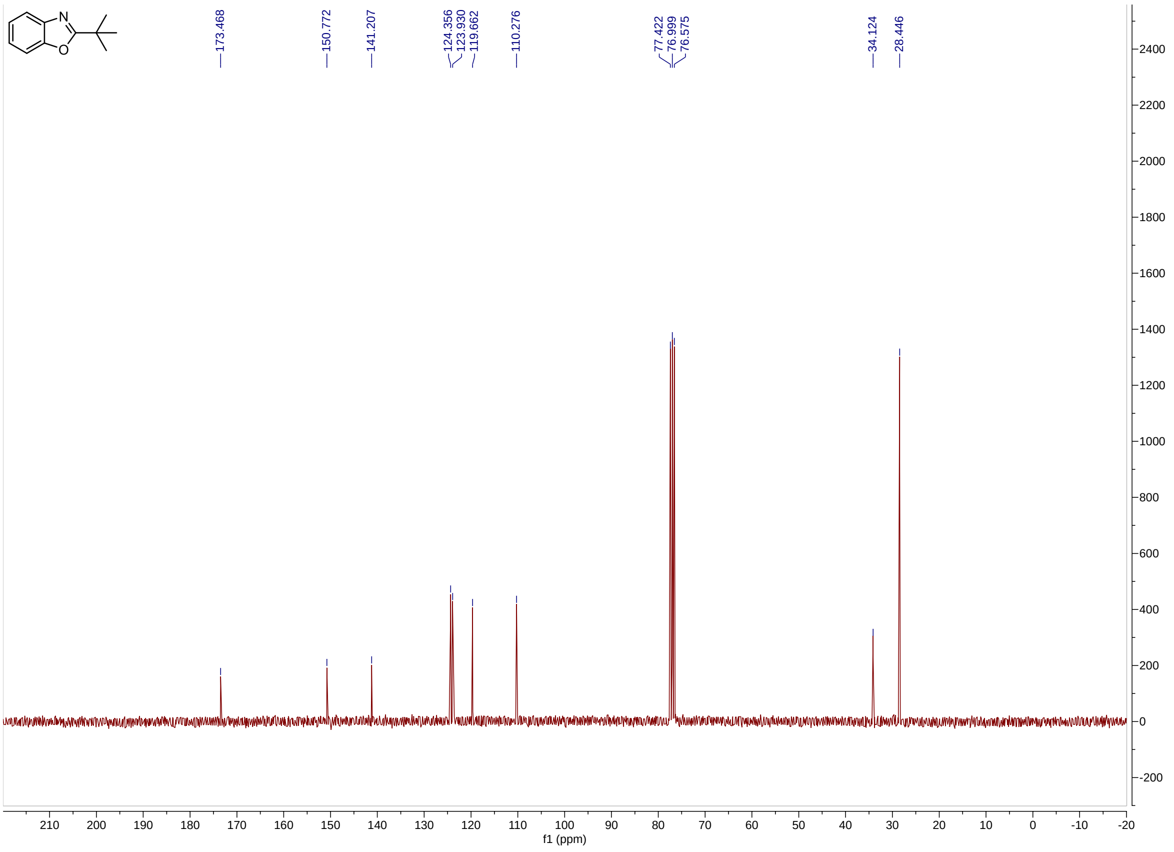

4a, ^{1}H NMR-300 MHz in $CDCl_3$ and ^{13}C NMR-75 MHz in $CDCl_3$

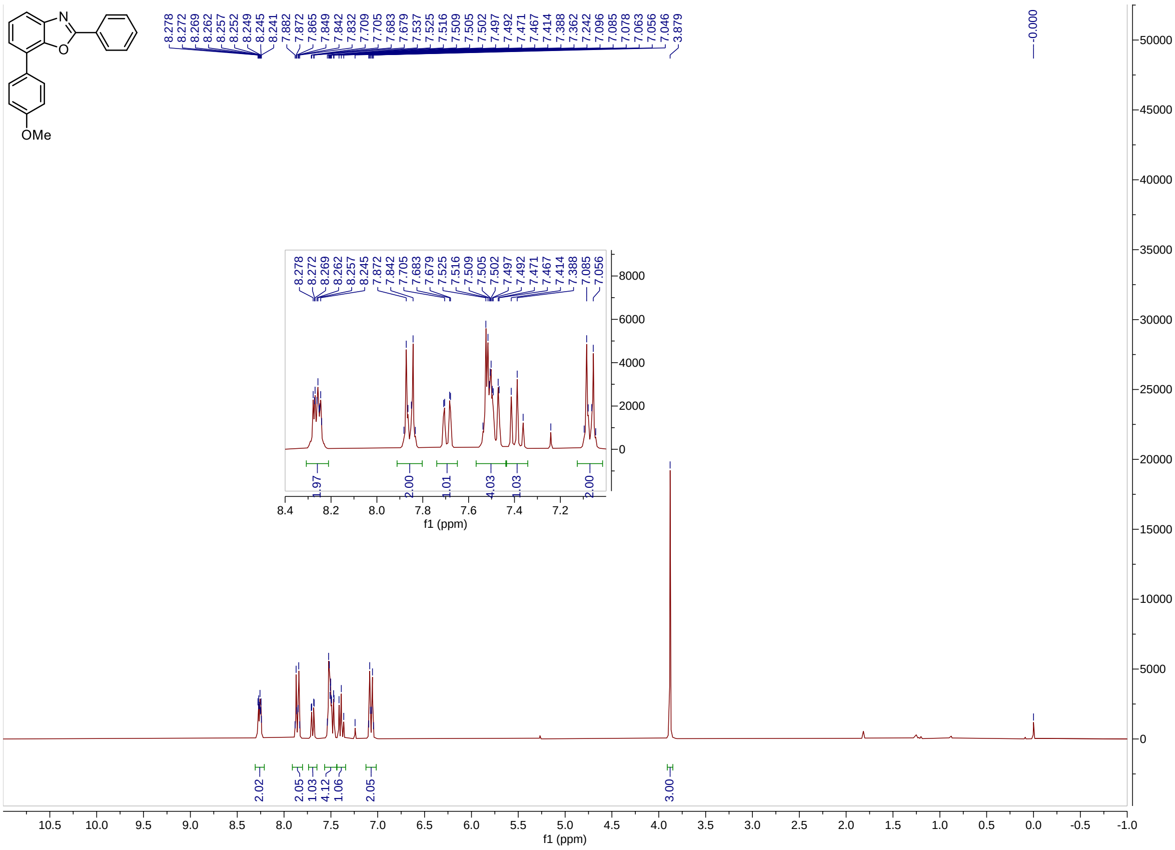

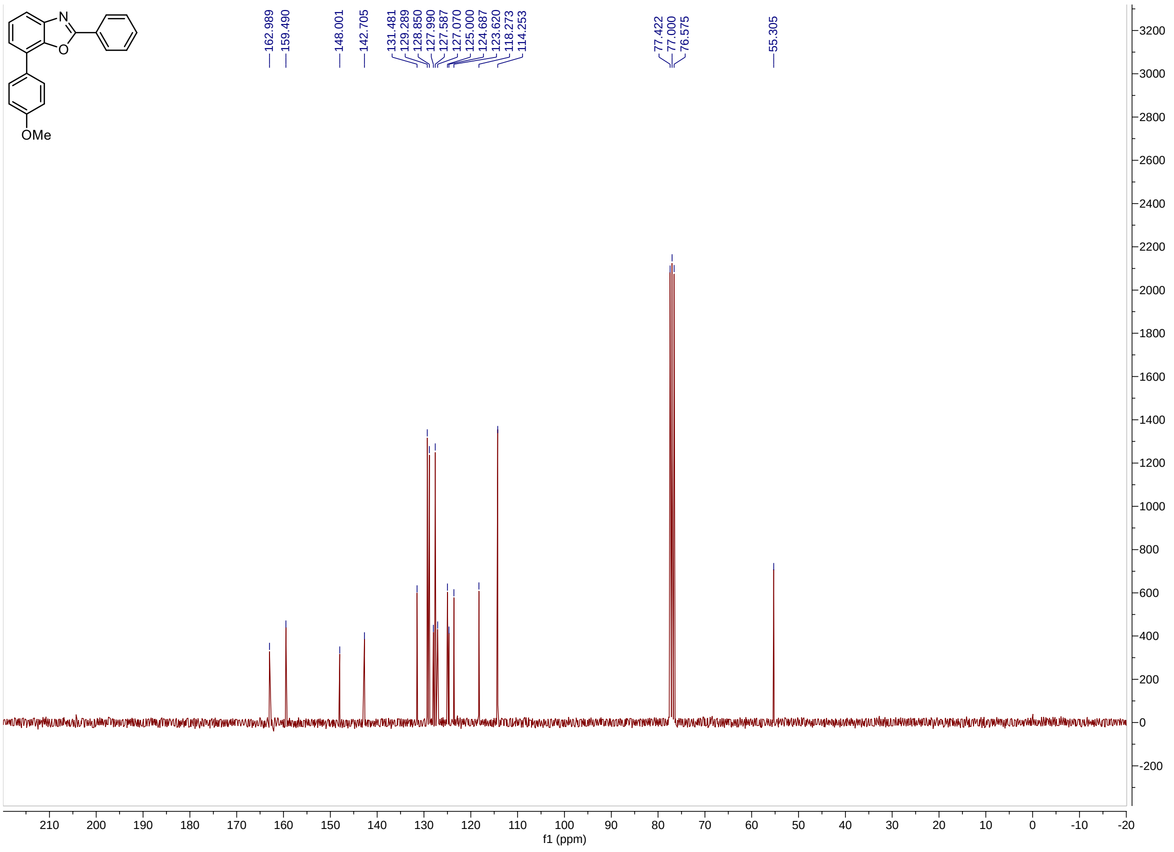

4b, ^{1}H NMR-300 MHz in $CDCl_3$ and ^{13}C NMR-75 MHz in $CDCl_3$

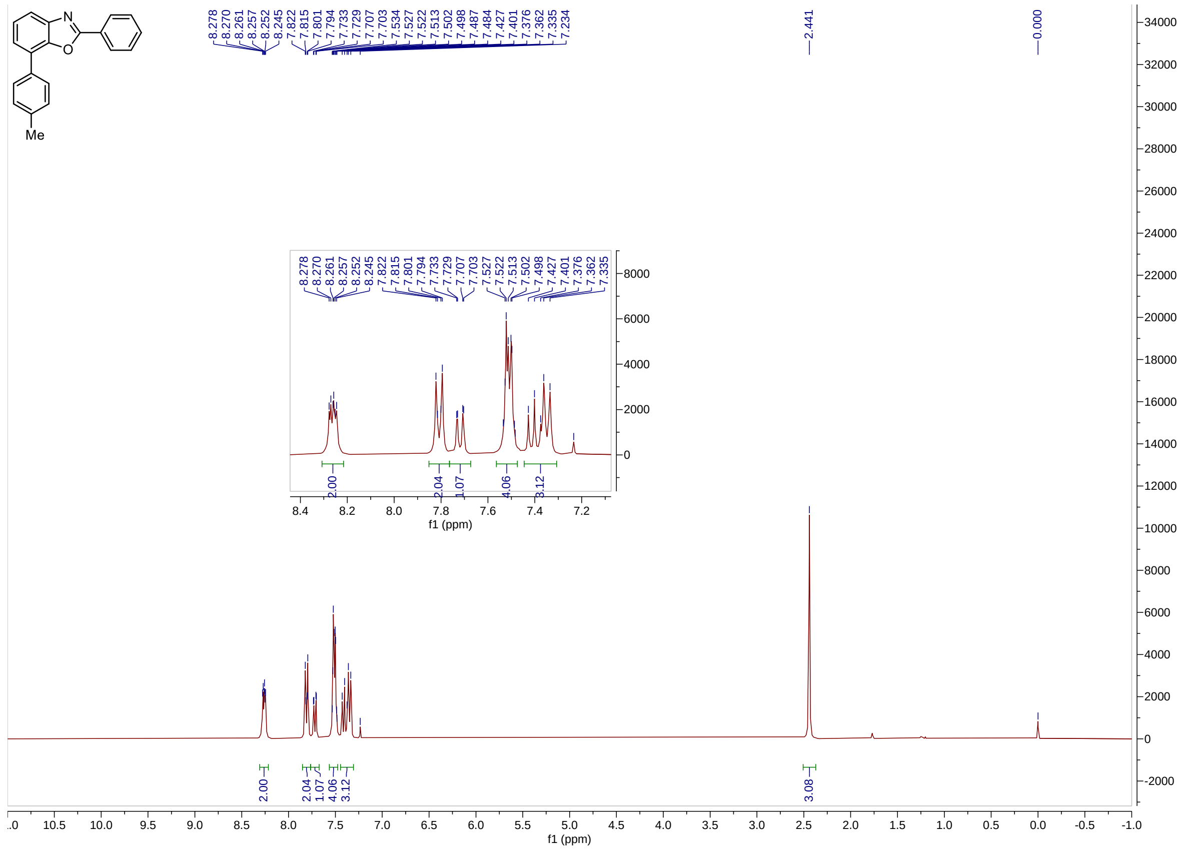

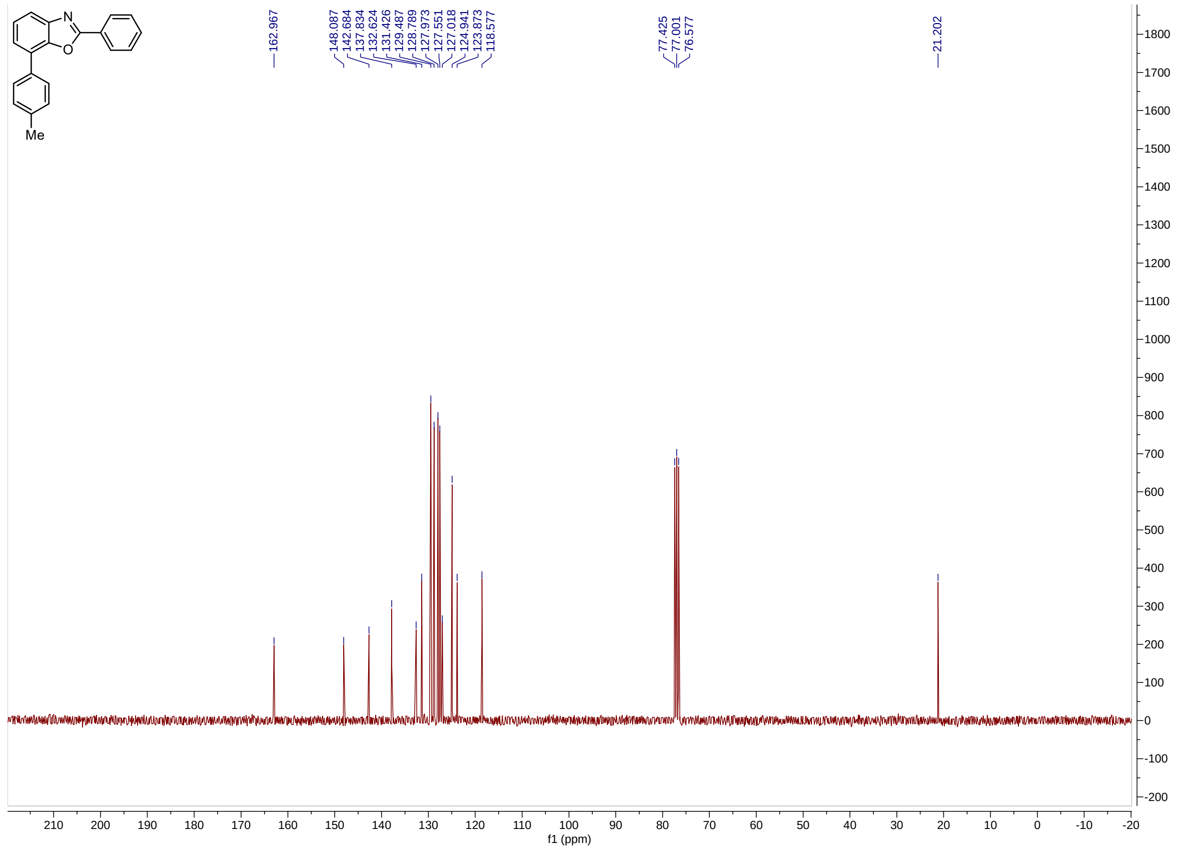

4c, ^{1}H NMR-300 MHz in $CDCl_3$ and ^{13}C NMR-75 MHz in $CDCl_3$

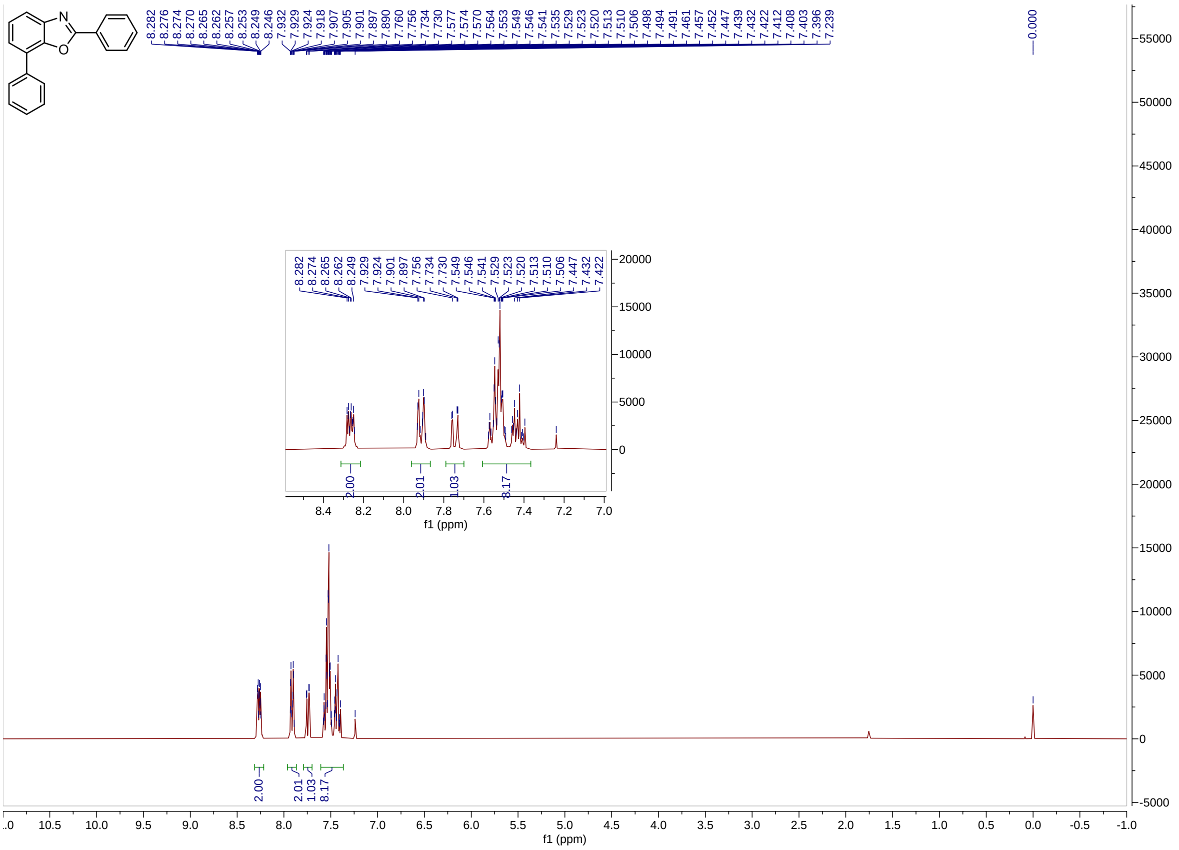

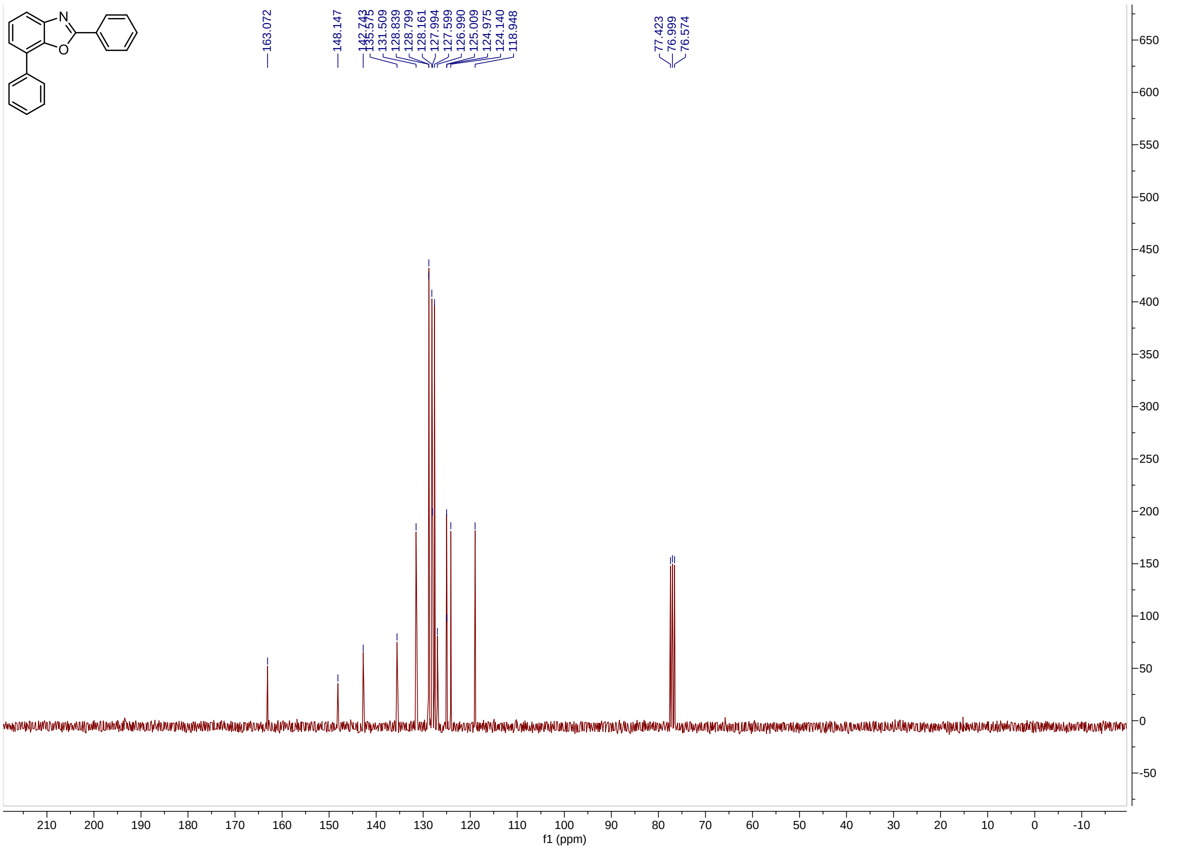

4d, ^{1}H NMR-300 MHz in $CDCl_3$ and ^{13}C NMR-75 MHz in $CDCl_3$

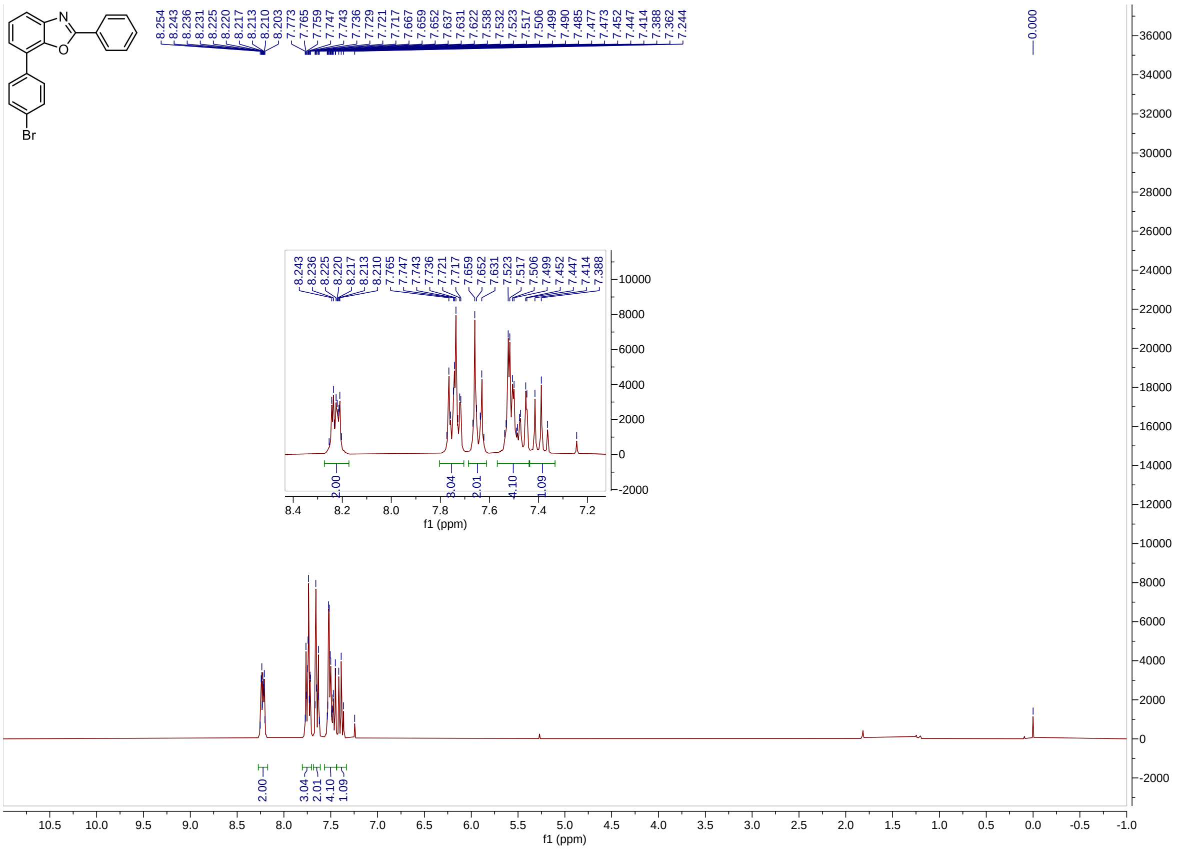

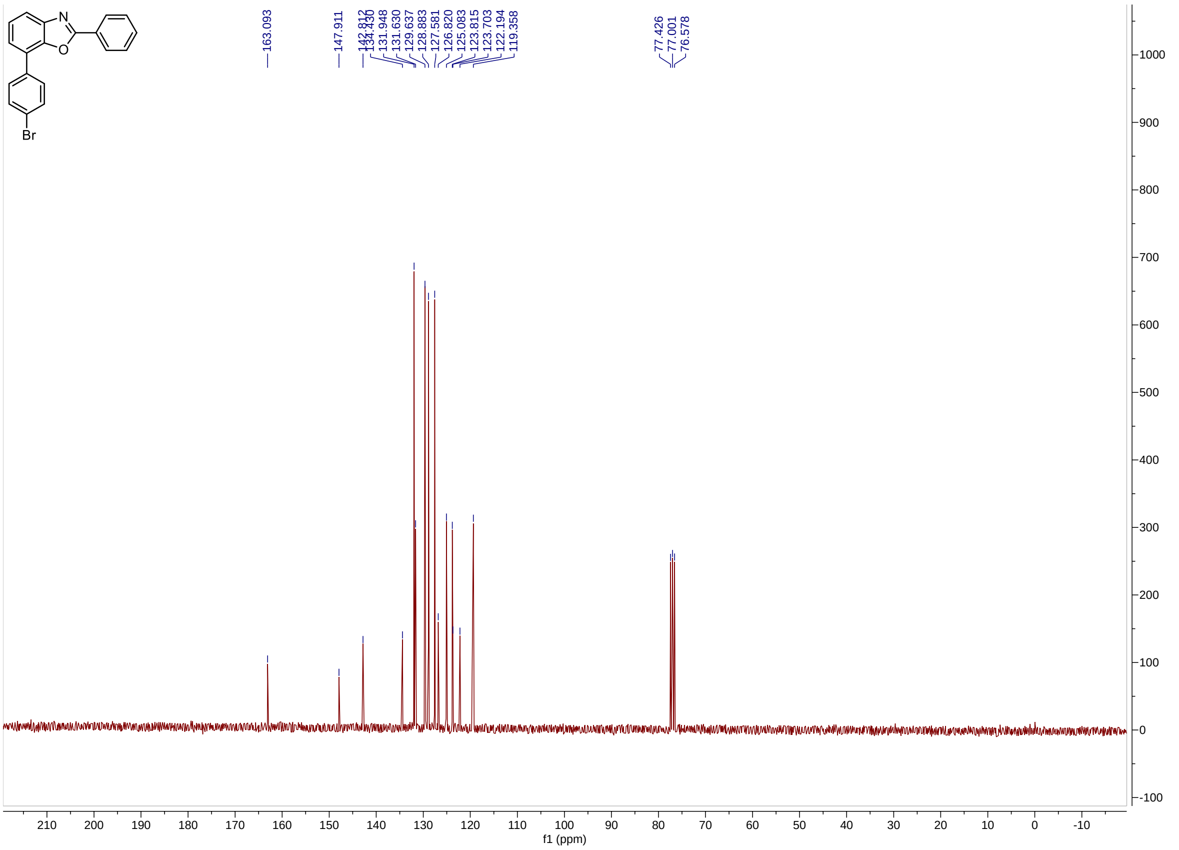

4e, ^{1}H NMR-300 MHz in $CDCl_3$ and ^{13}C NMR-75 MHz in $CDCl_3$

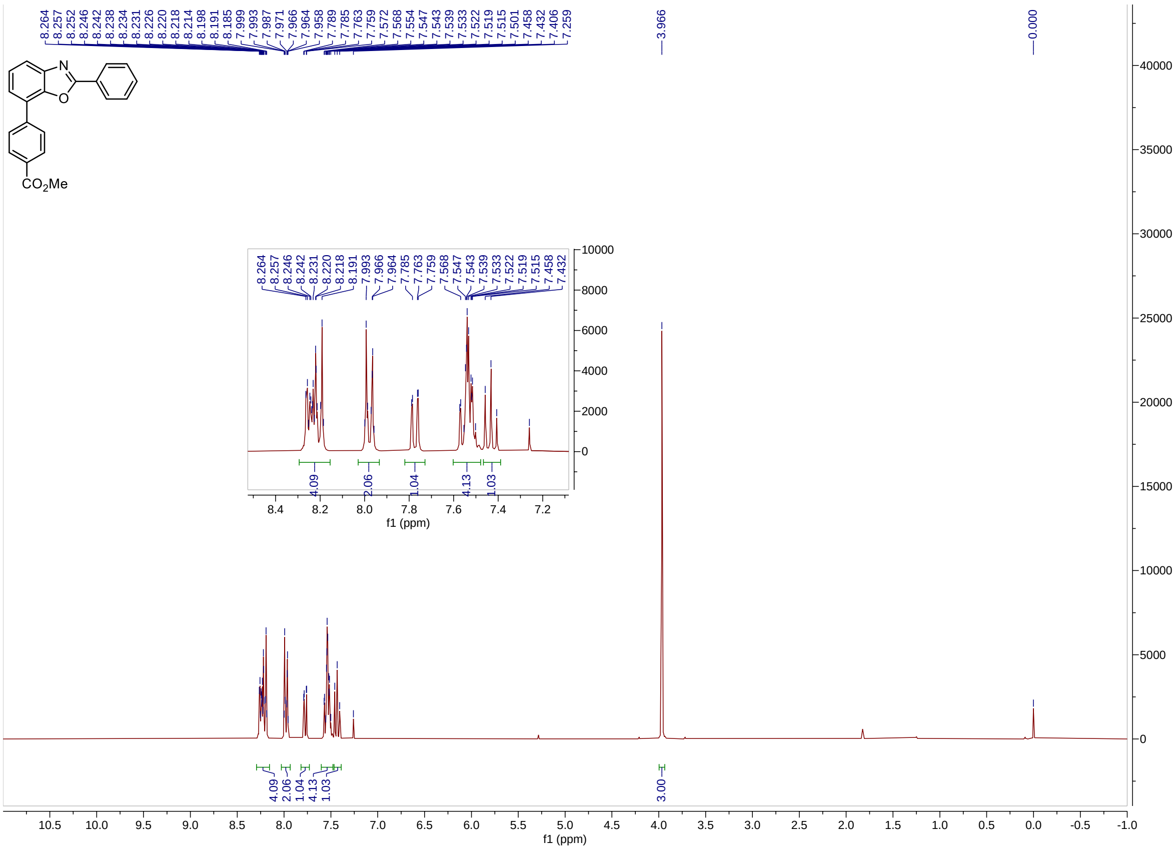

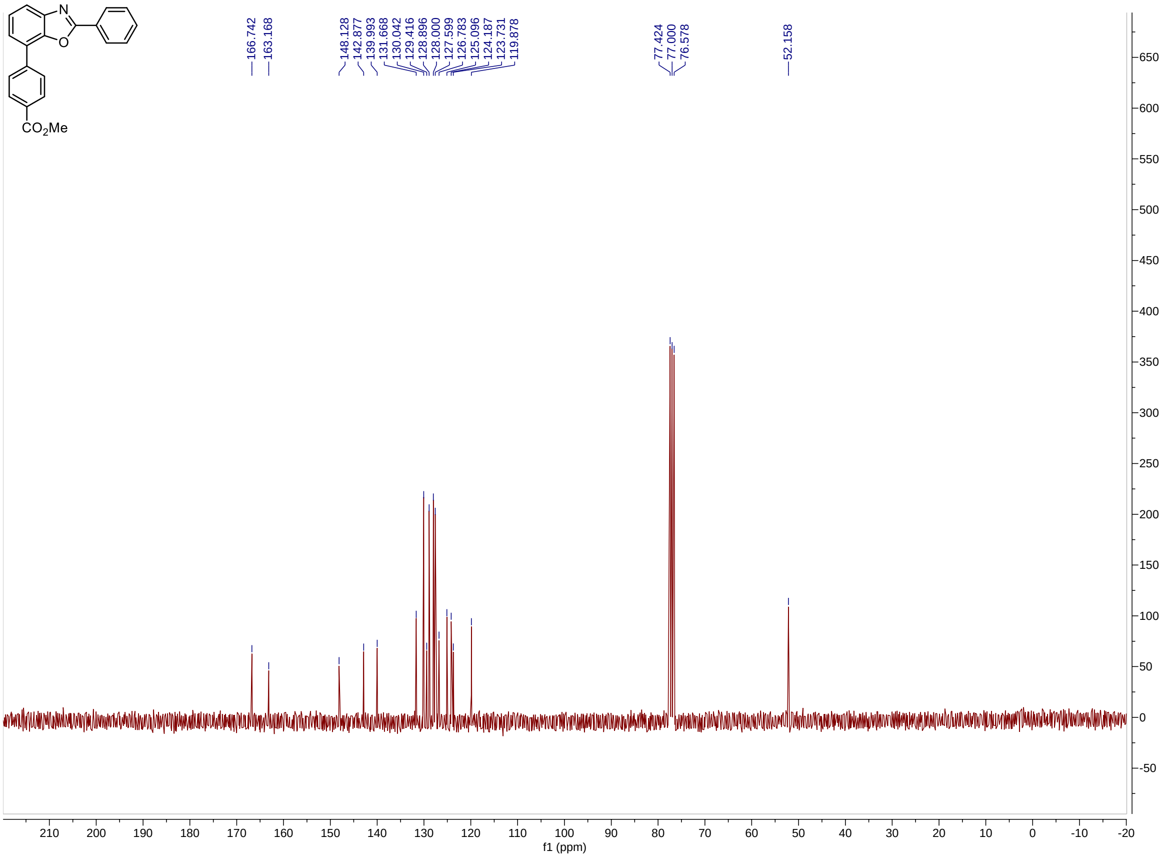

4f, ^{1}H NMR-300 MHz in $CDCl_3$ and ^{13}C NMR-75 MHz in $CDCl_3$

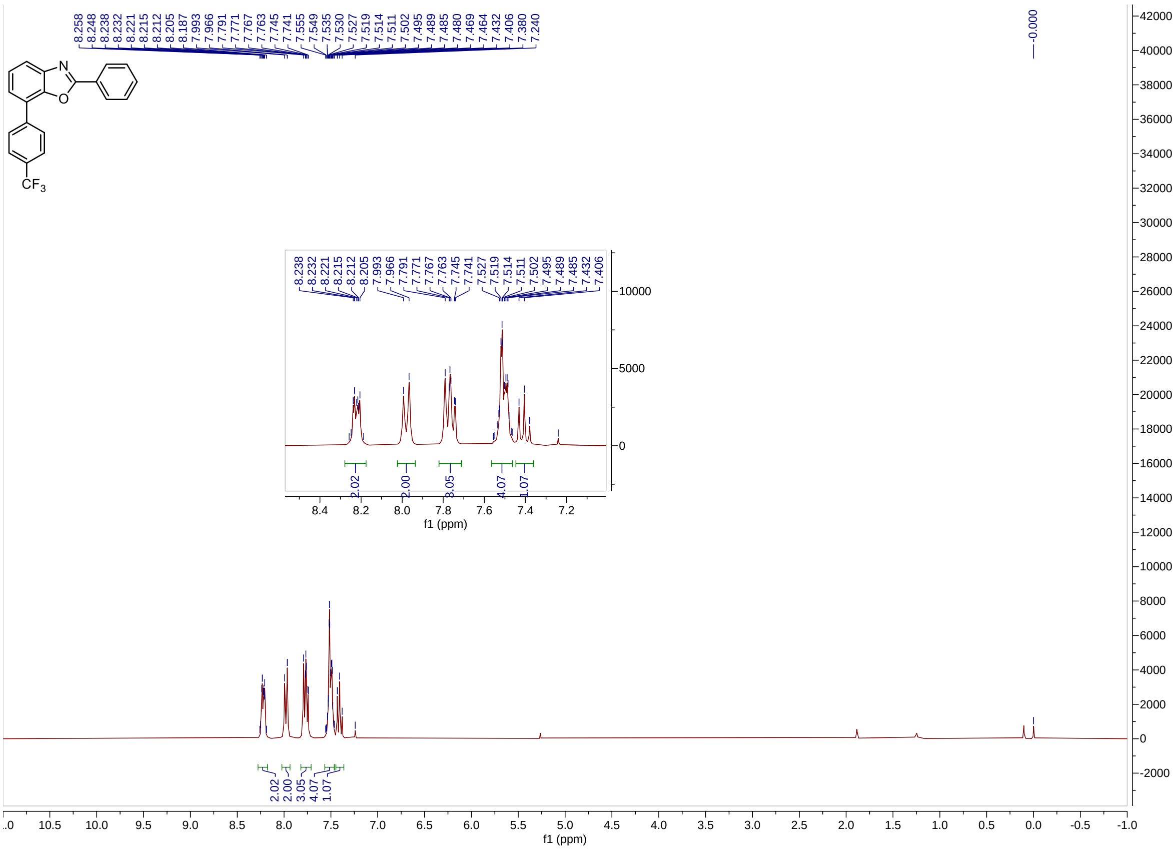

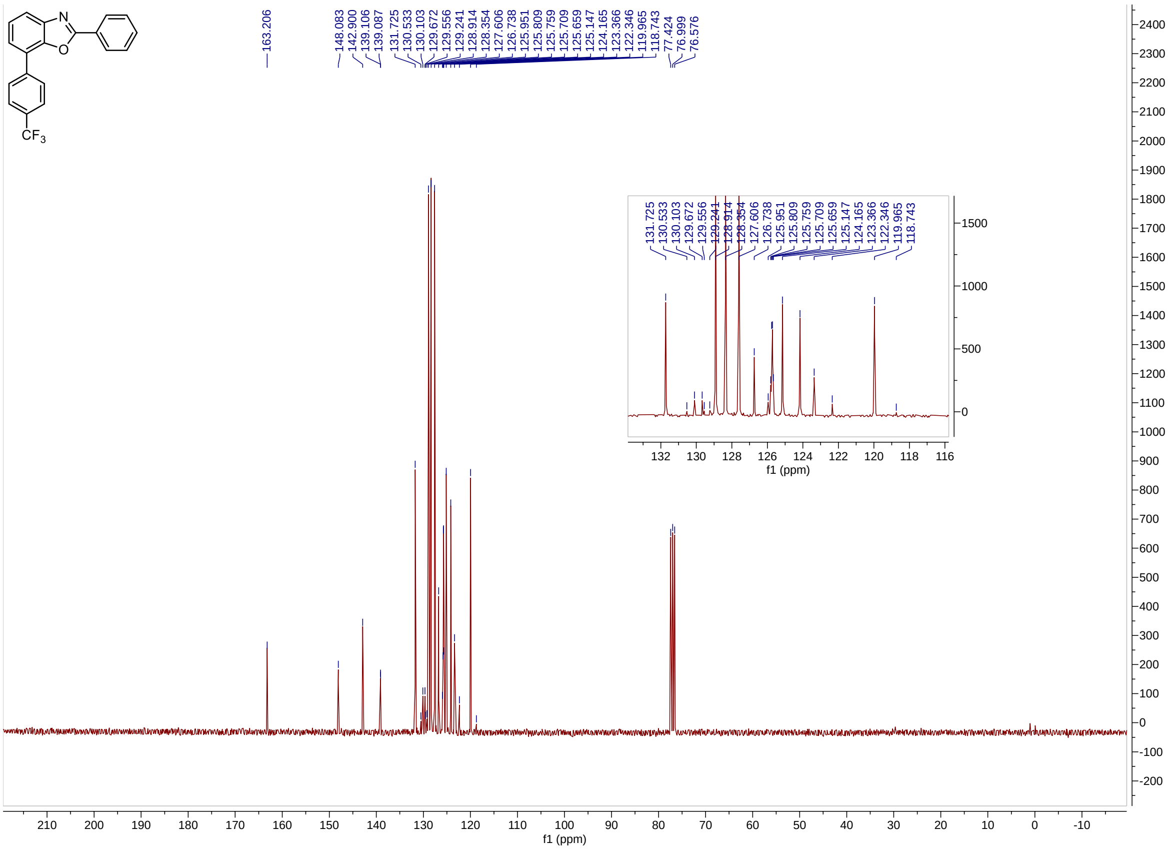

4g, ^{1}H NMR-300 MHz in CD_2Cl_2 and ^{13}C NMR-75 MHz in CD_2Cl_2

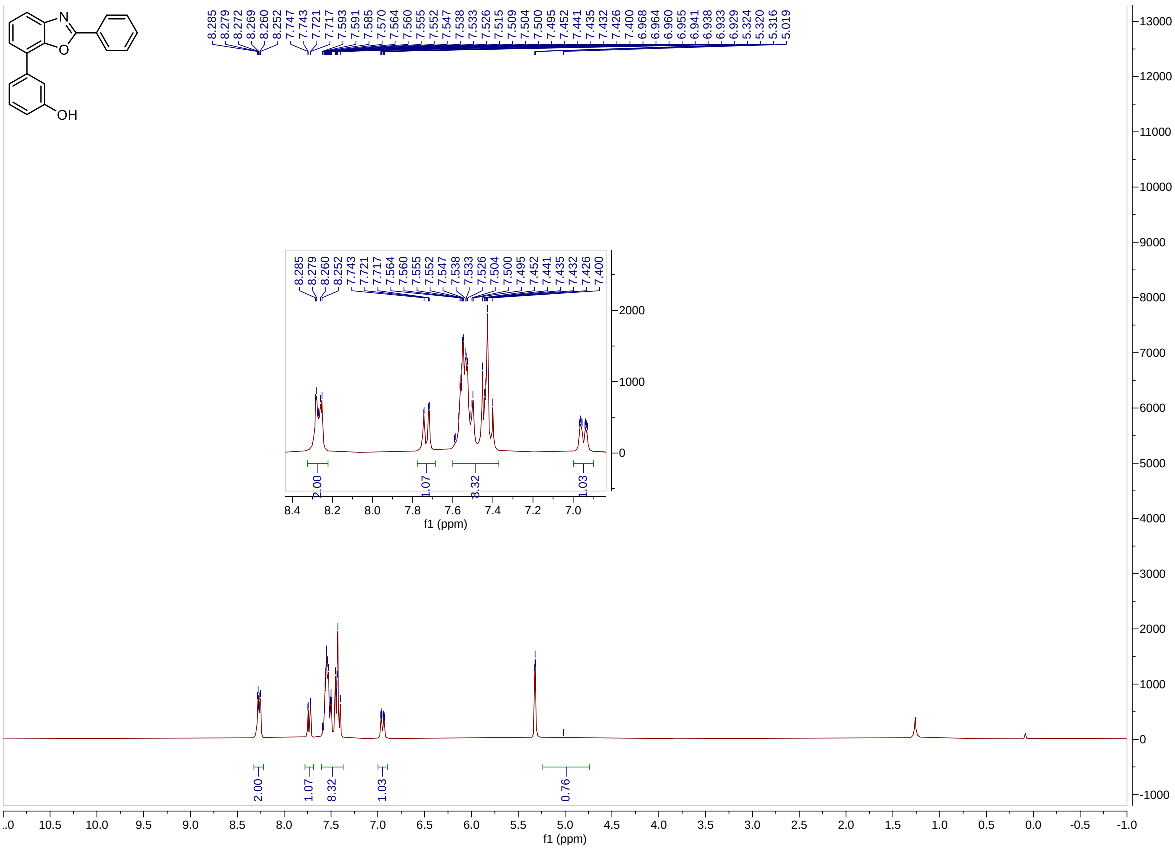

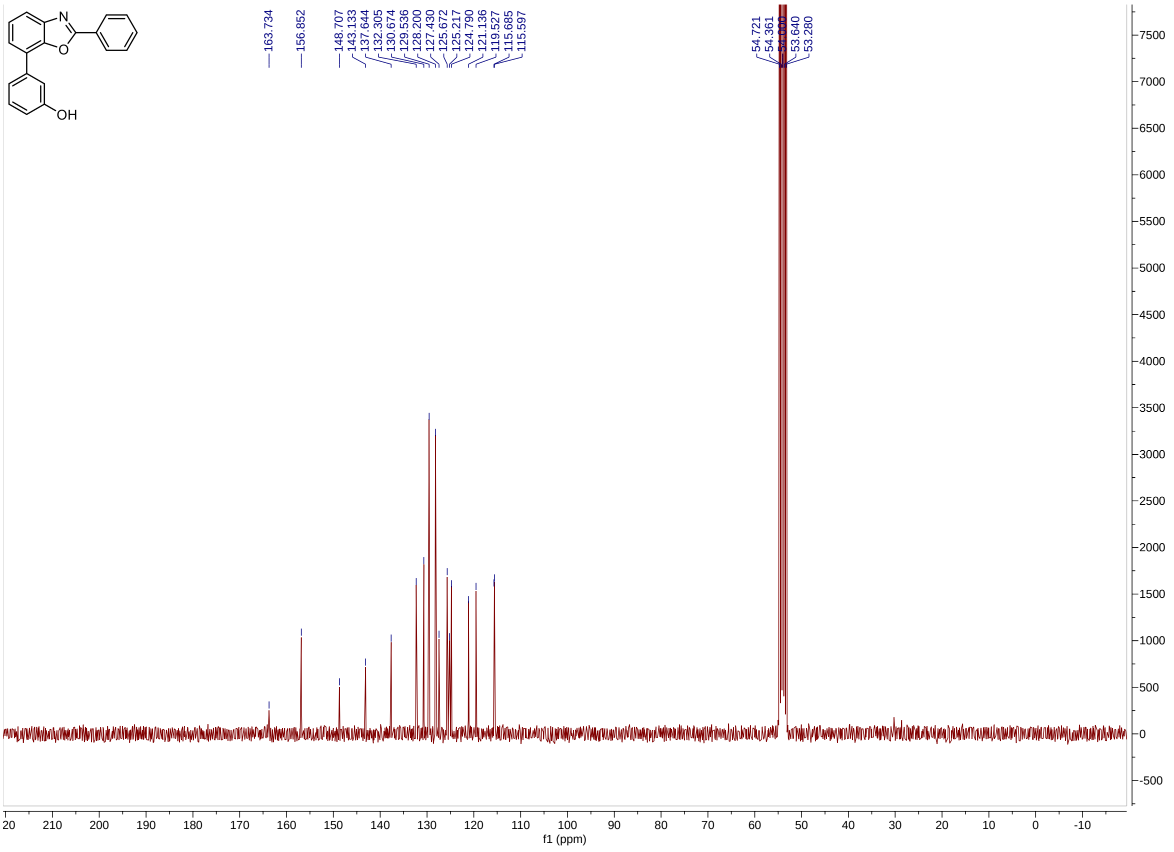

4h, ^{1}H NMR-300 MHz in $CDCl_3$ and ^{13}C NMR-75 MHz in $CDCl_3$

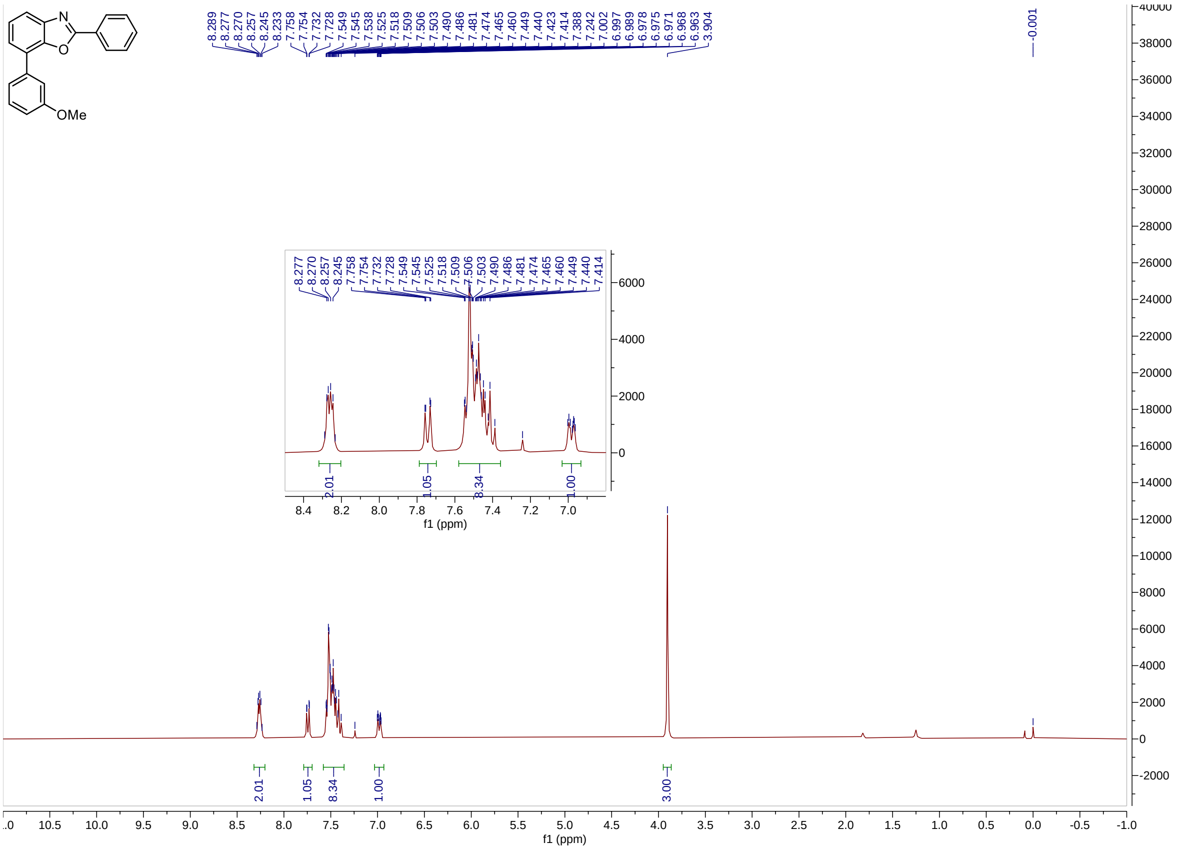

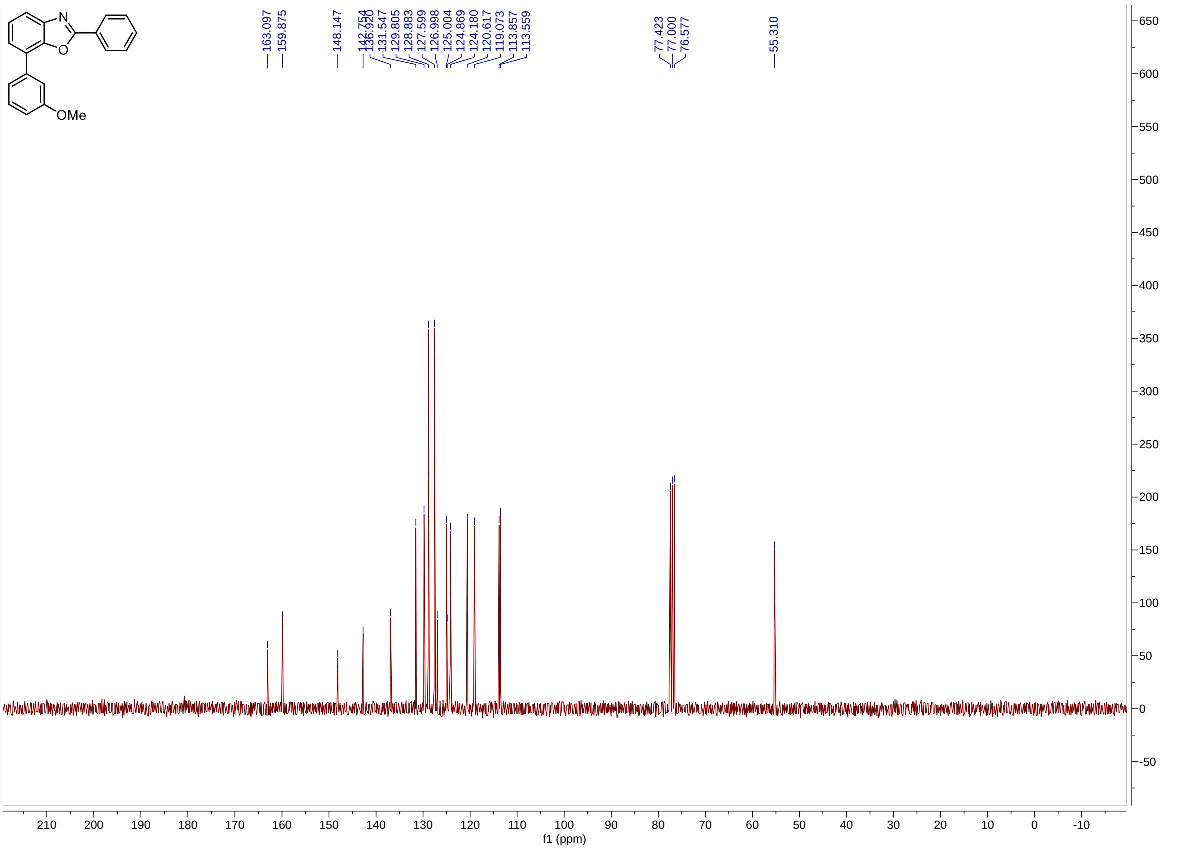

4i, ^{1}H NMR-300 MHz in $CDCl_3$ and ^{13}C NMR-75 MHz in $CDCl_3$

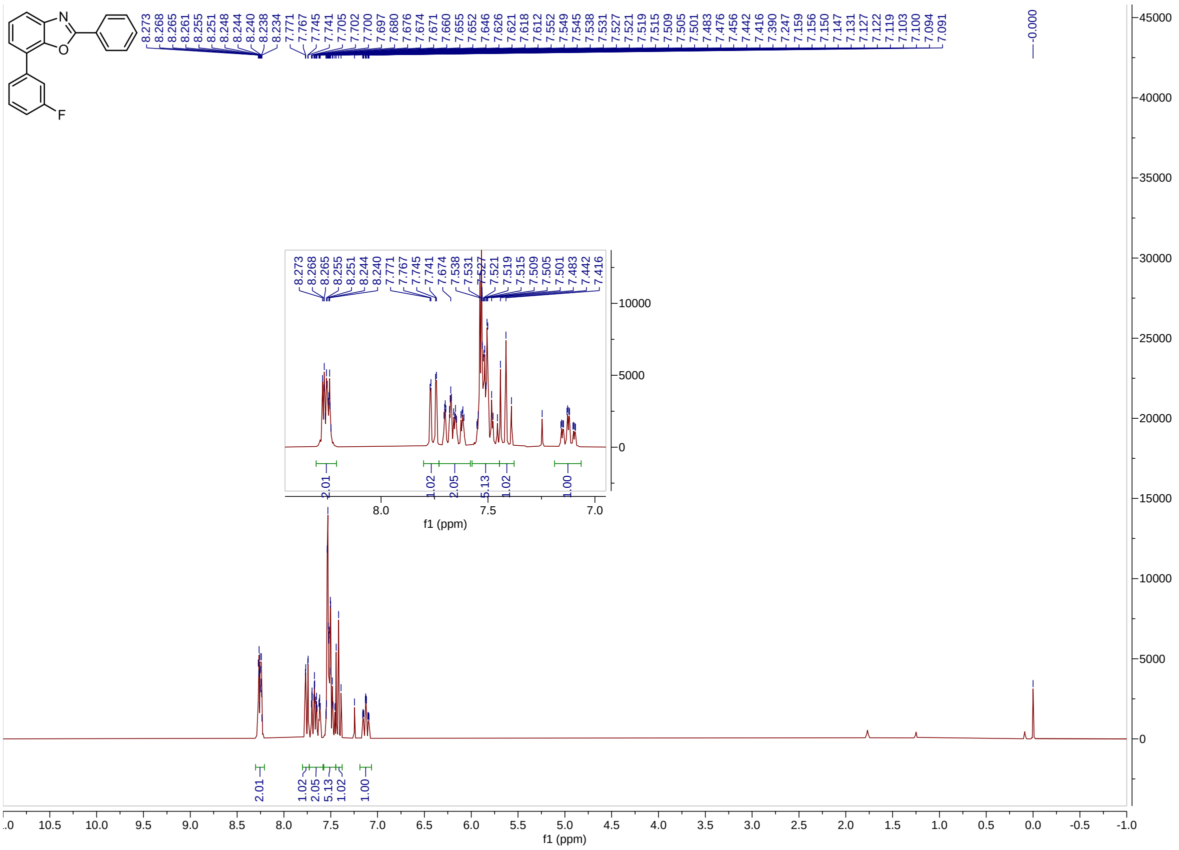

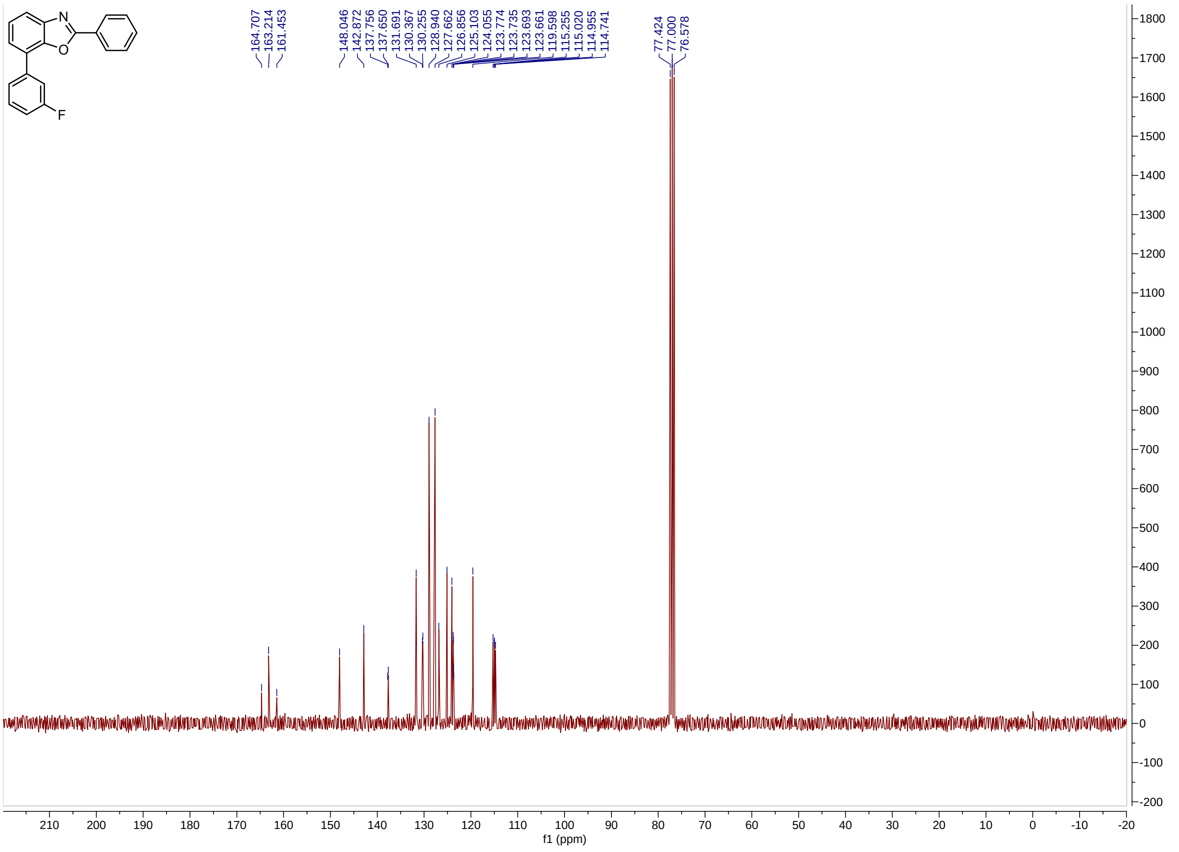

4j, ^{1}H NMR-300 MHz in $CDCl_3$ and ^{13}C NMR-75 MHz in $CDCl_3$

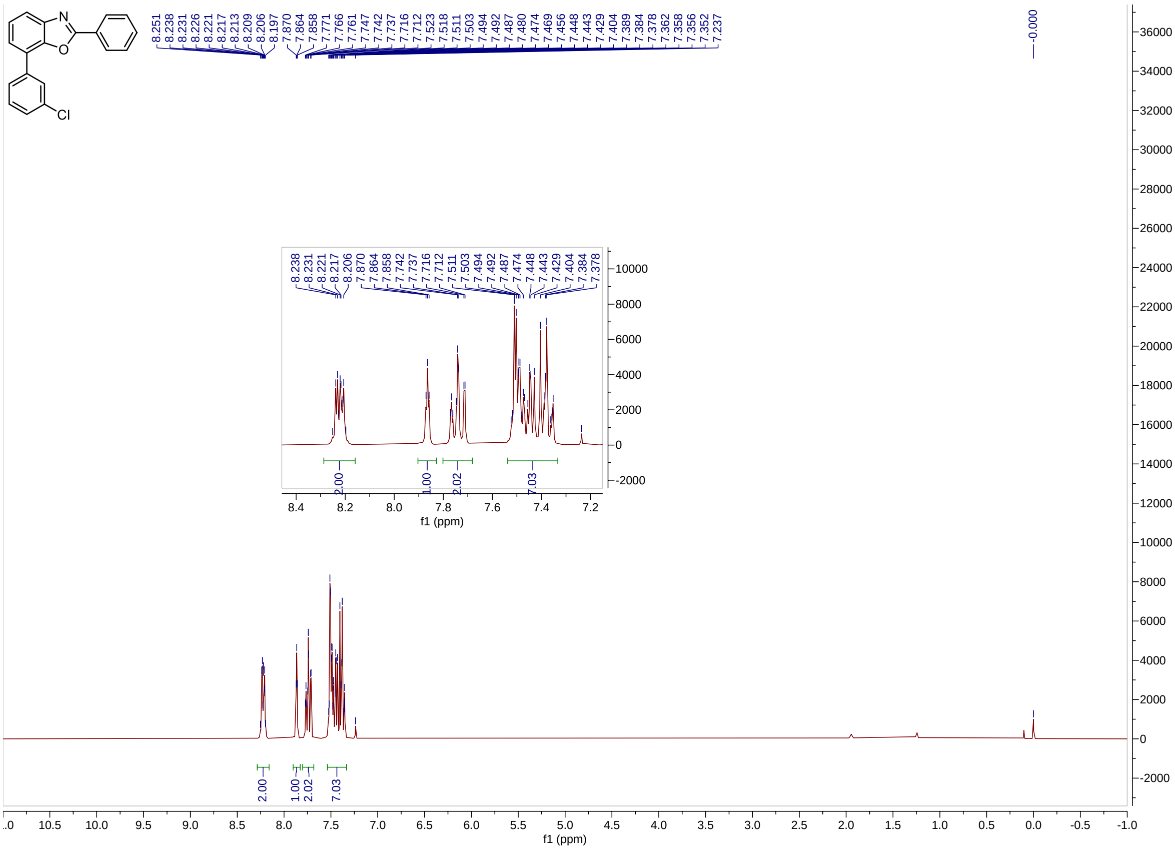

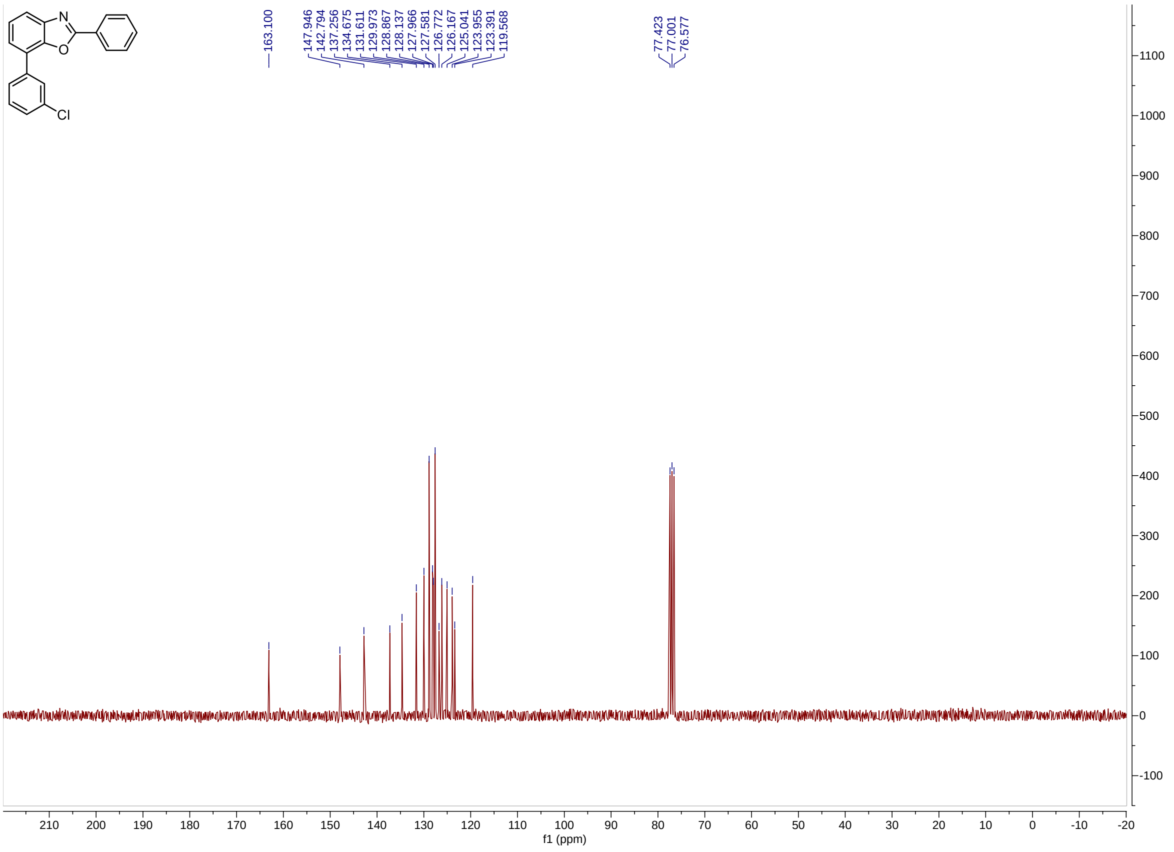

4k, ^{1}H NMR-300 MHz in $CDCl_3$ and ^{13}C NMR-75 MHz in $CDCl_3$

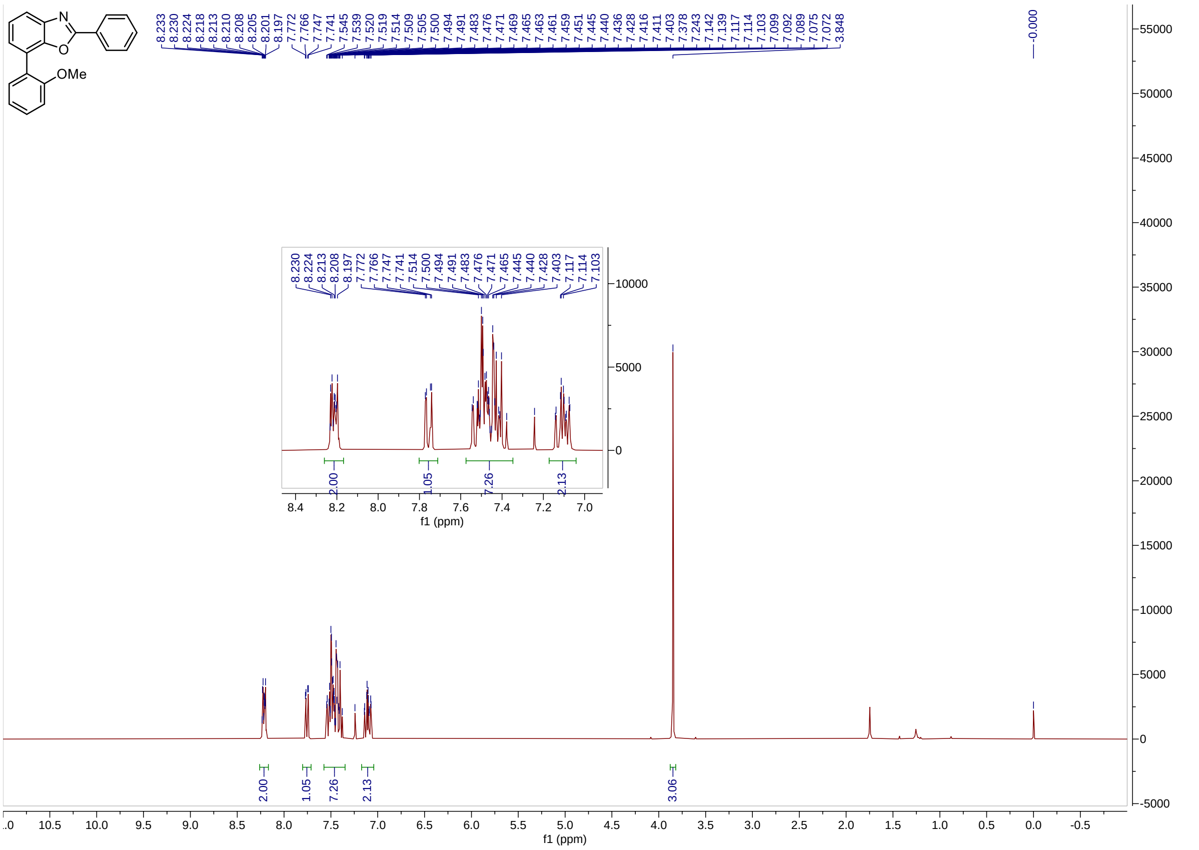

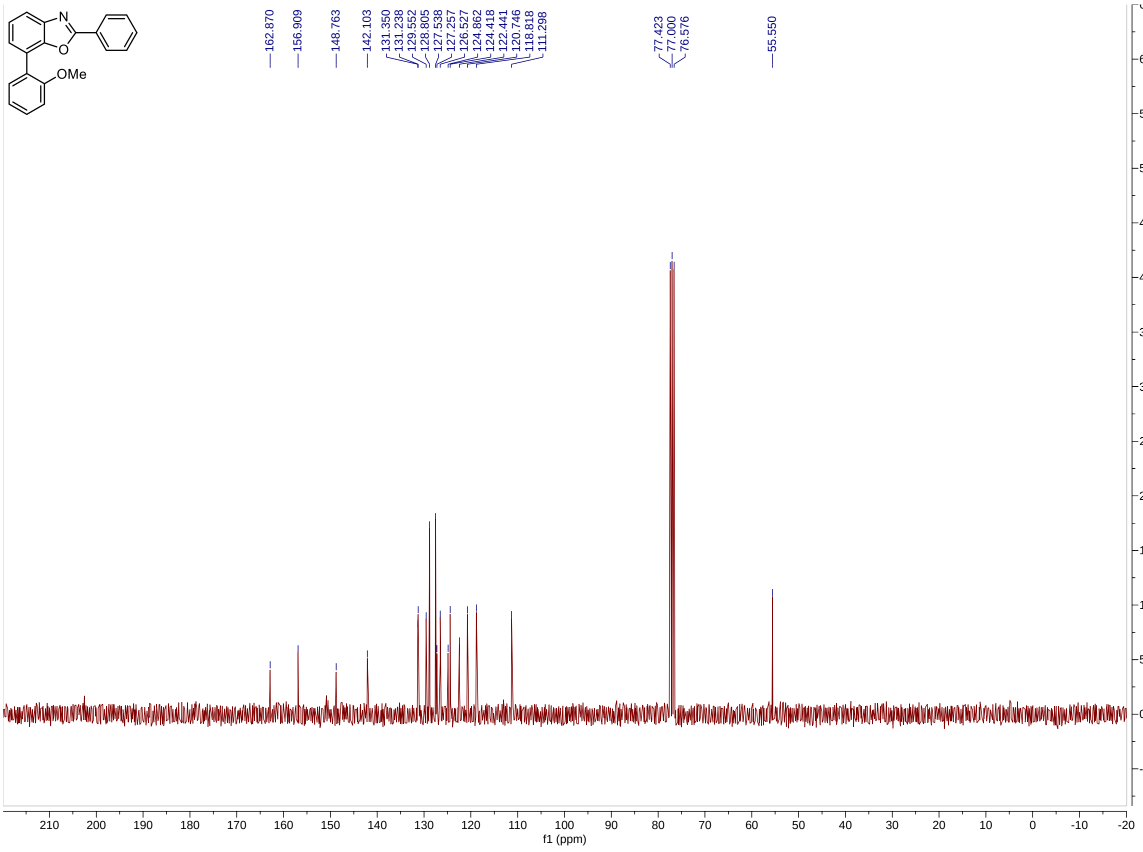

4l, ^{1}H NMR-300 MHz in $CDCl_3$ and ^{13}C NMR-75 MHz in $CDCl_3$

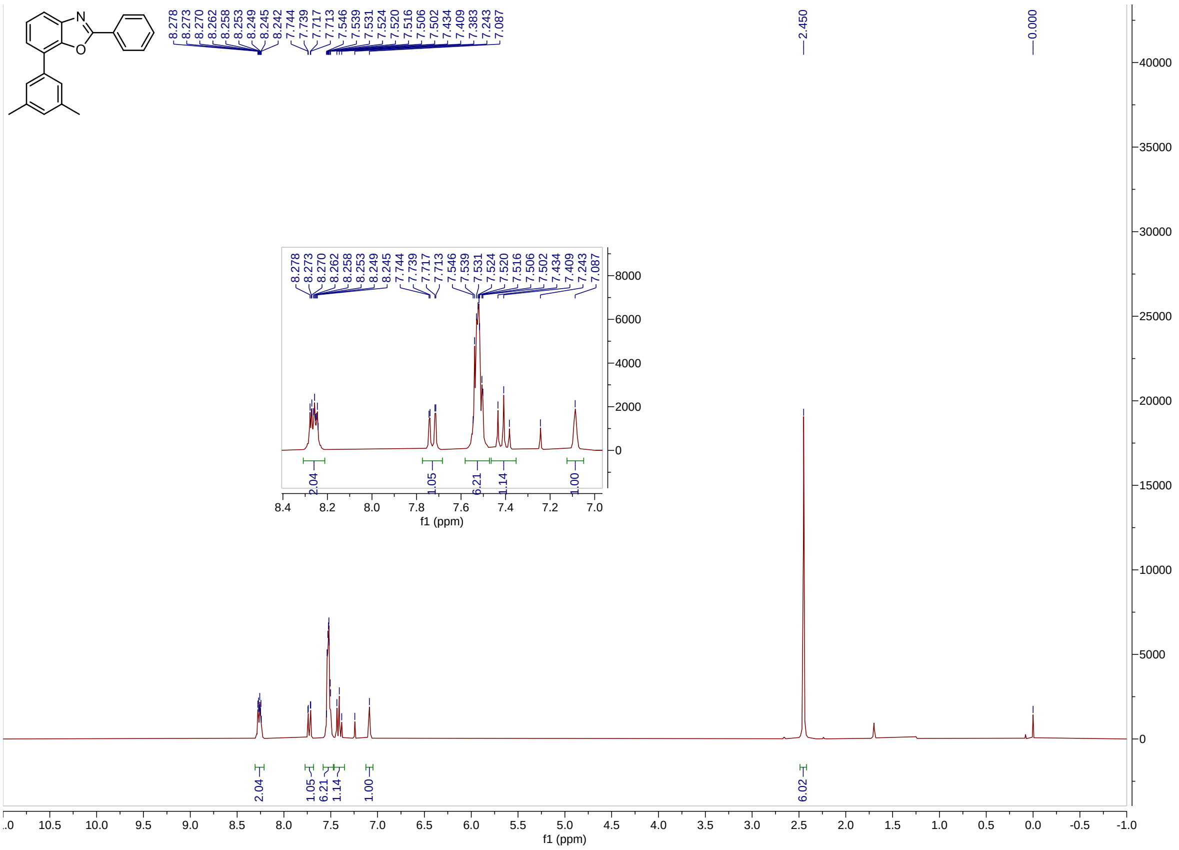

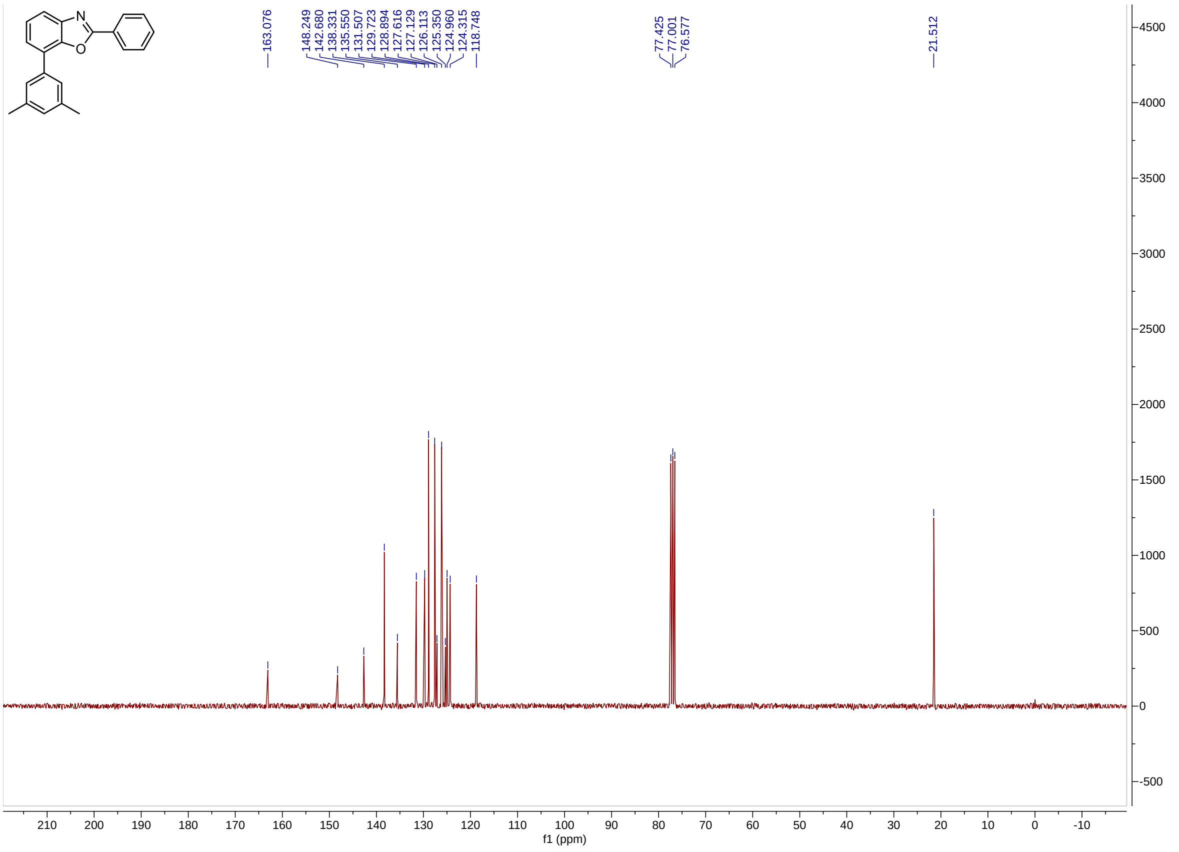

4m, ^{1}H NMR-300 MHz in $CDCl_3$ and ^{13}C NMR-75 MHz in $CDCl_3$

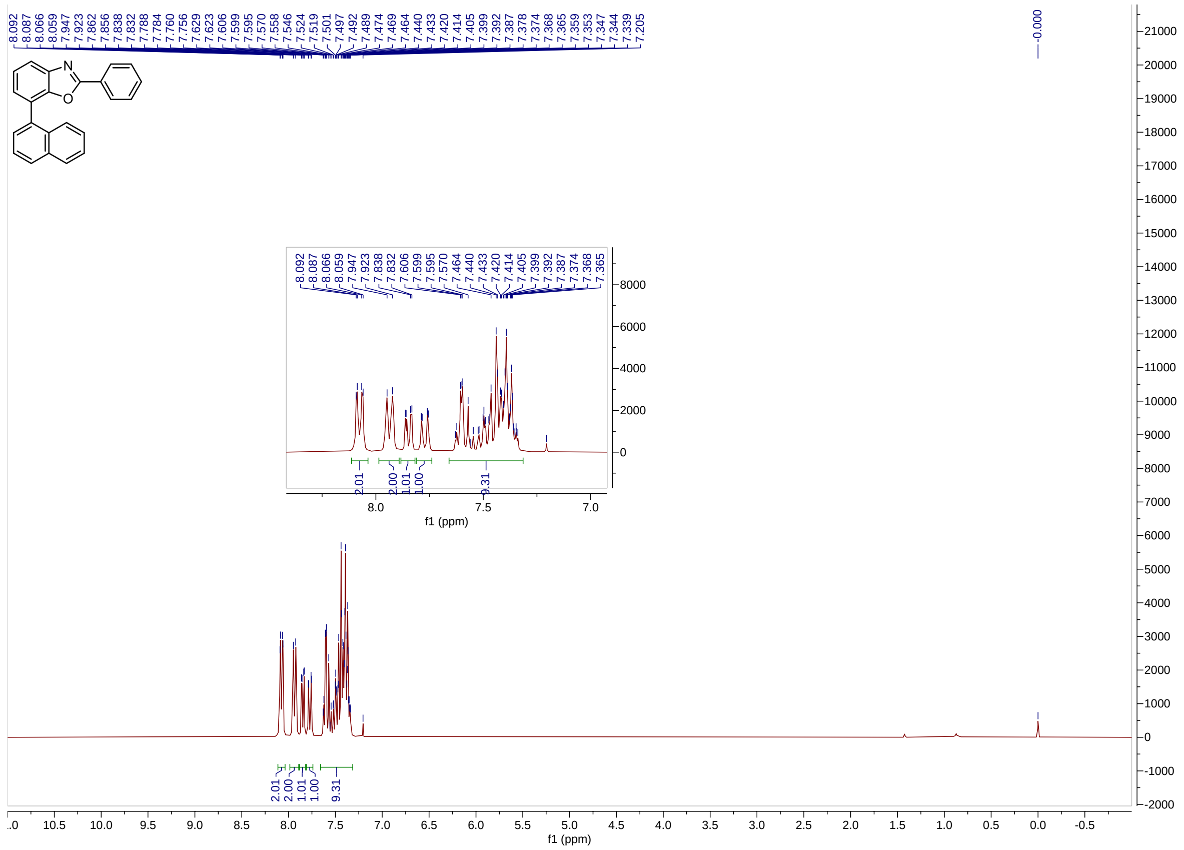

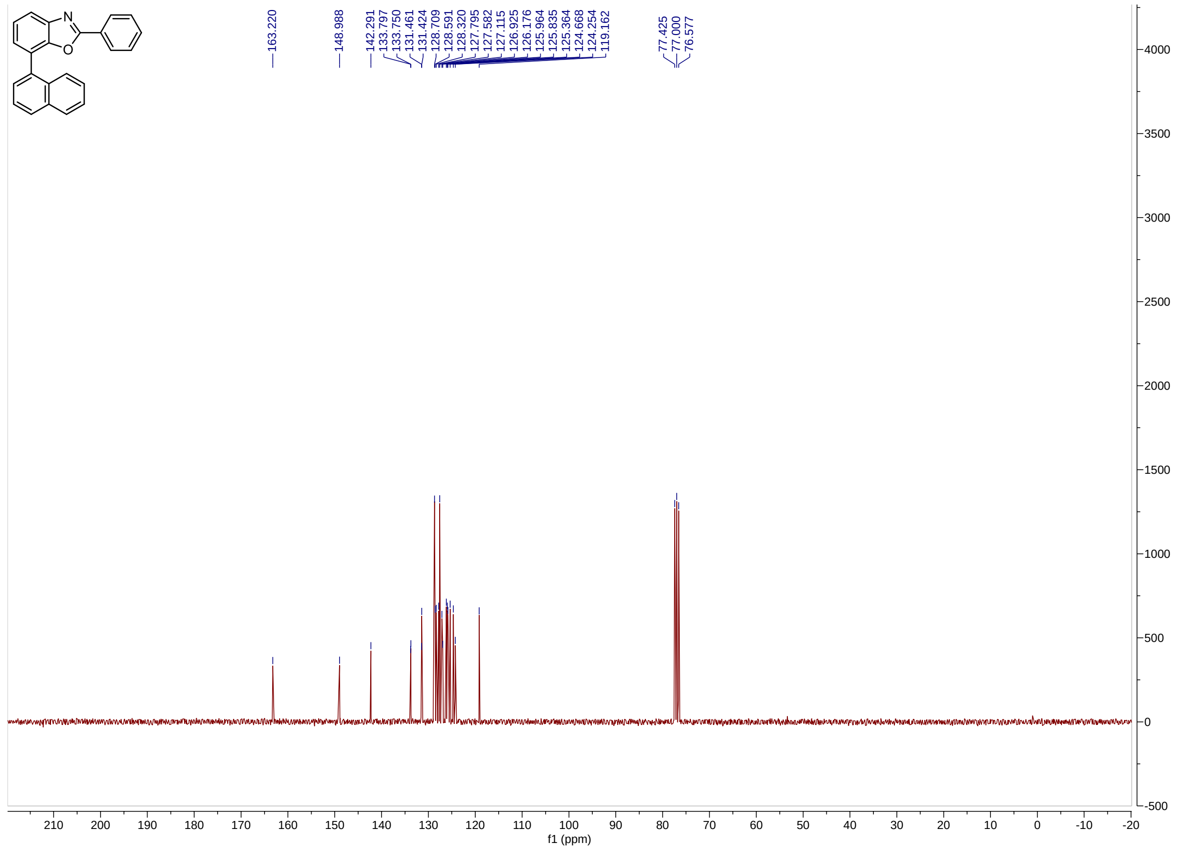

4n, ^{1}H NMR-300 MHz in $CDCl_3$ and ^{13}C NMR-75 MHz in $CDCl_3$

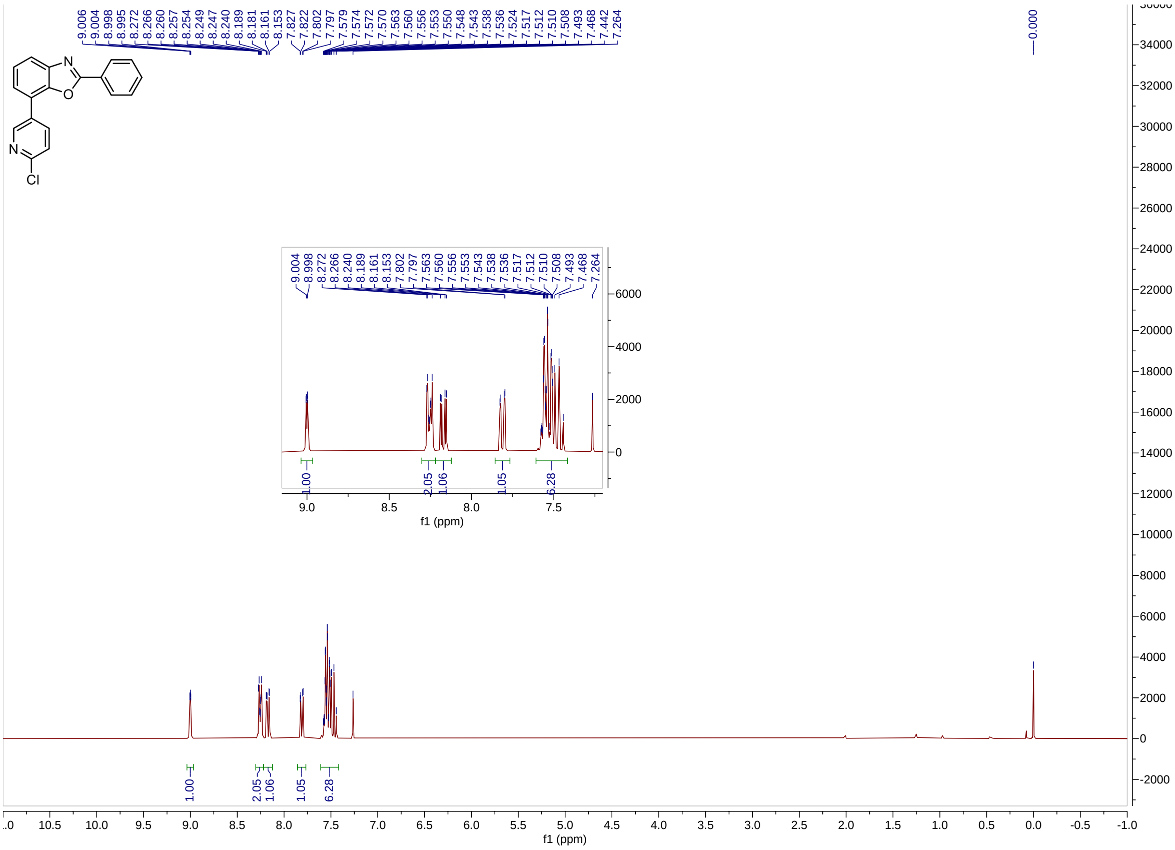

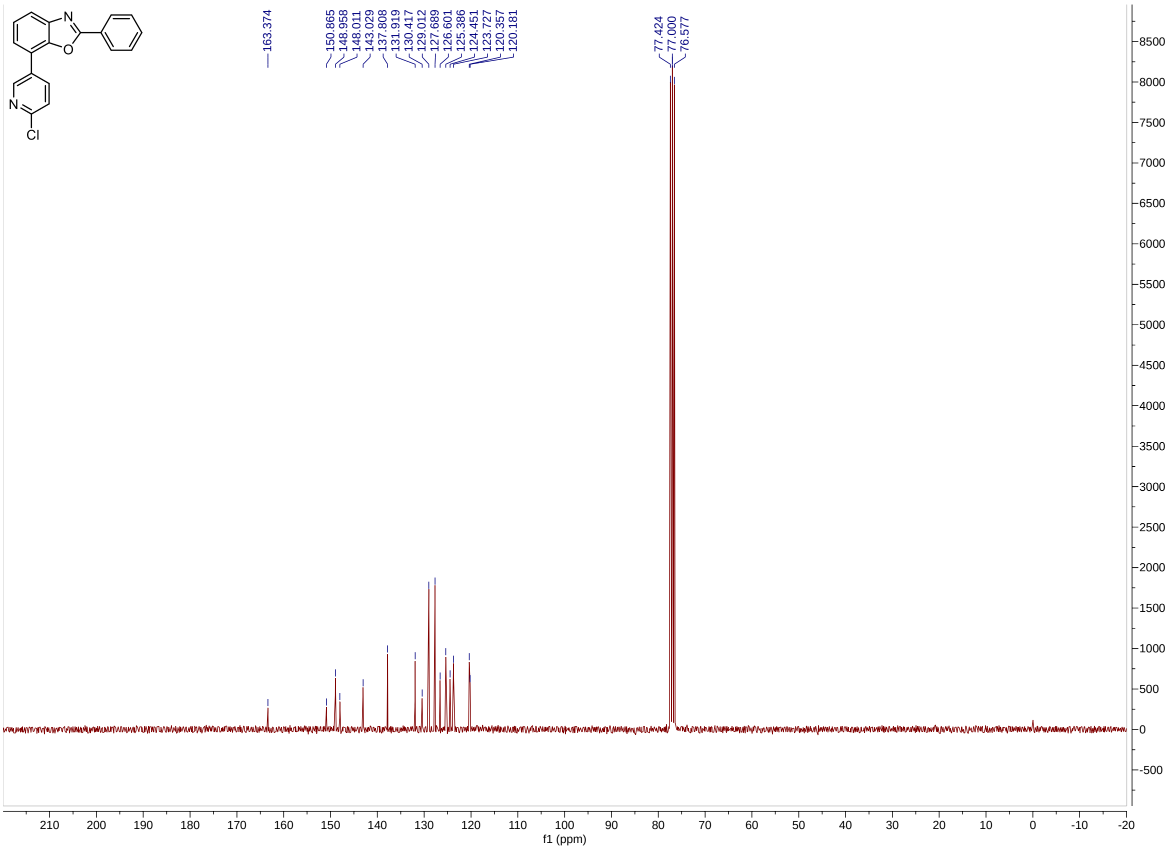

4o, ^{1}H NMR-300 MHz in $CDCl_3$ and ^{13}C NMR-75 MHz in $CDCl_3$

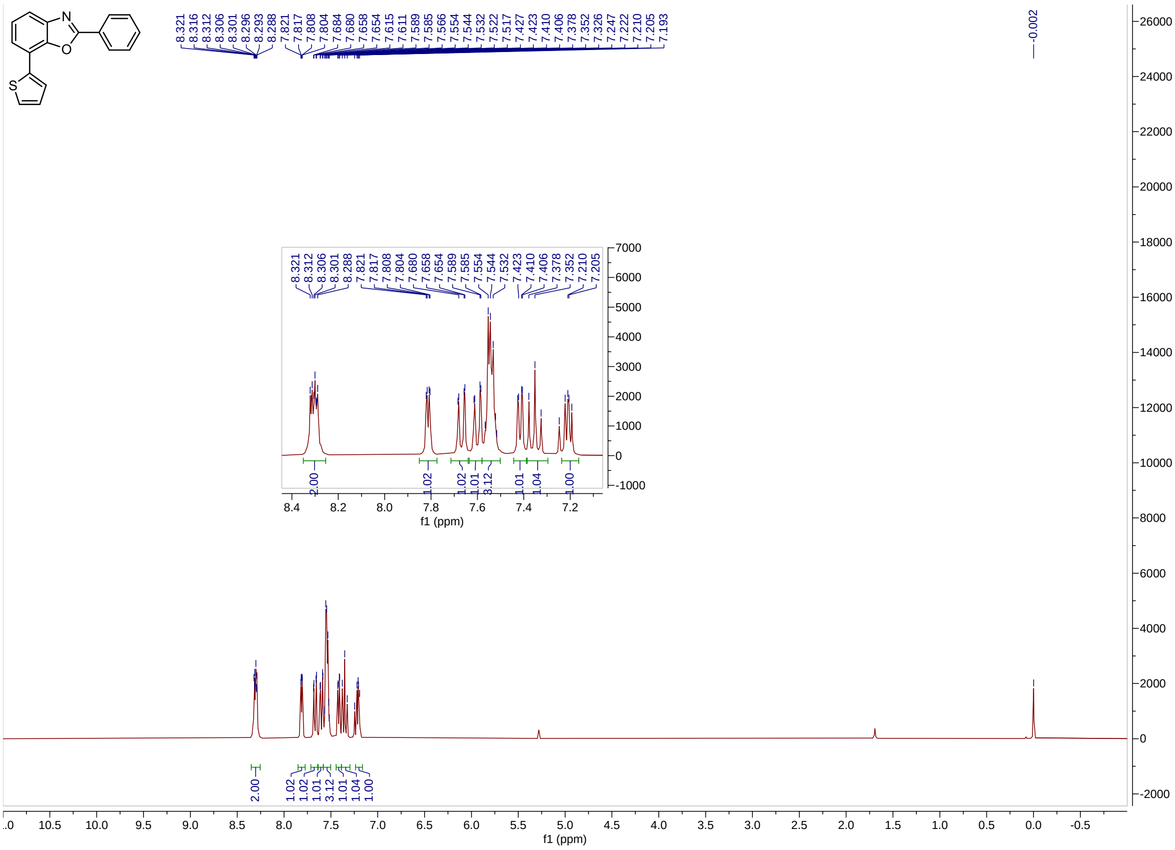

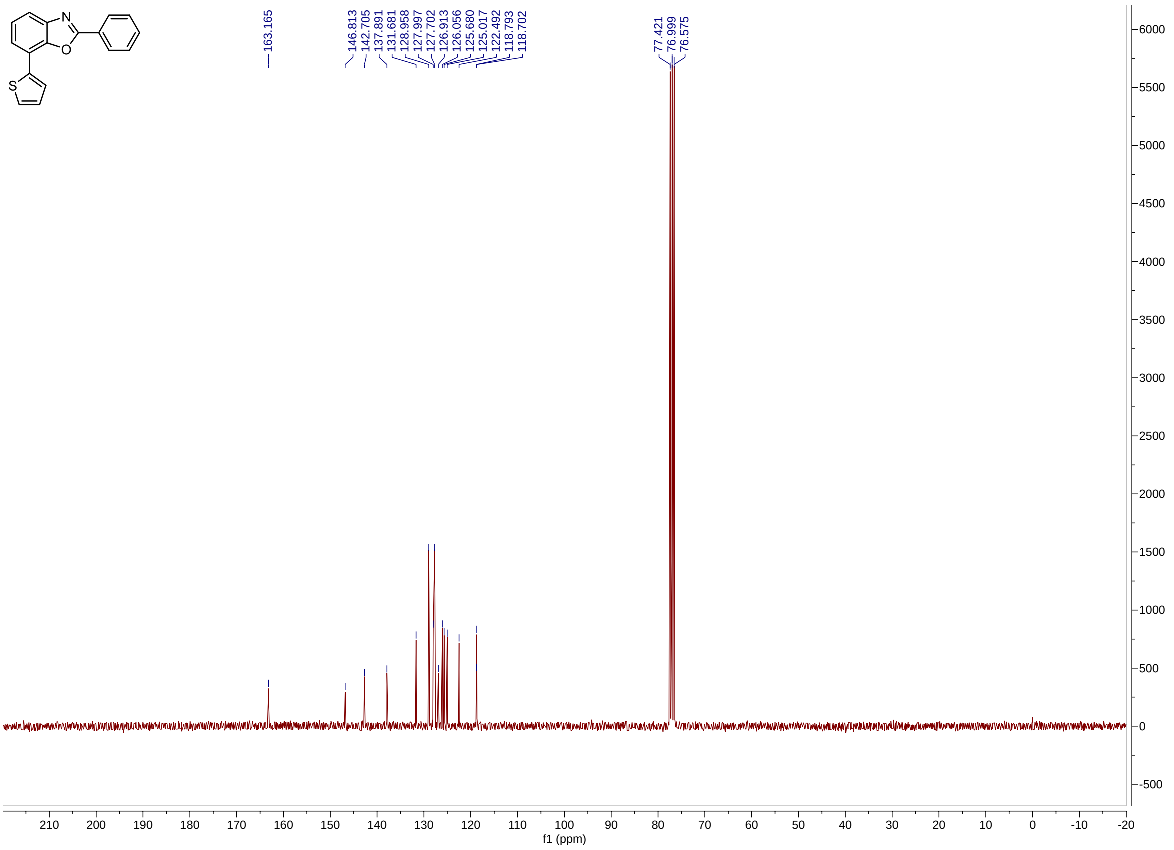

4p, ^{1}H NMR-300 MHz in $CDCl_3$ and ^{13}C NMR-75 MHz in $CDCl_3$

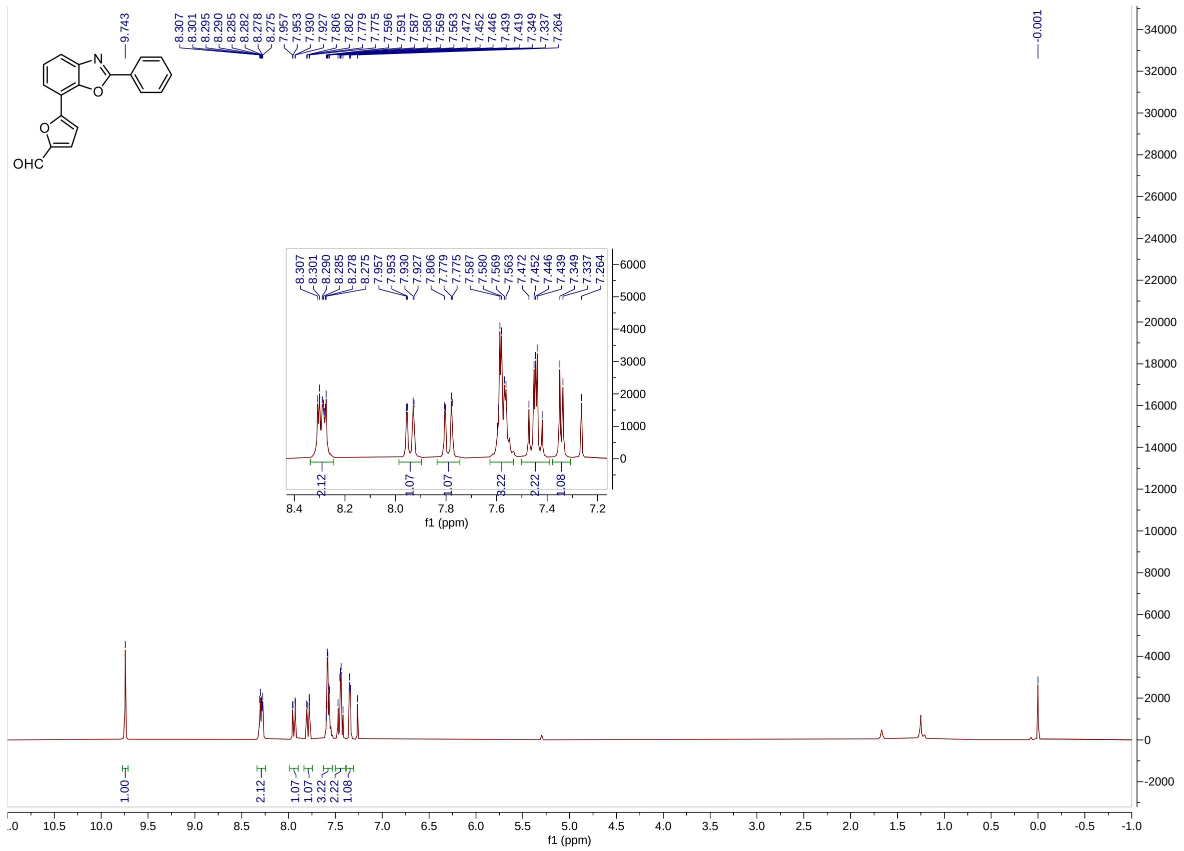

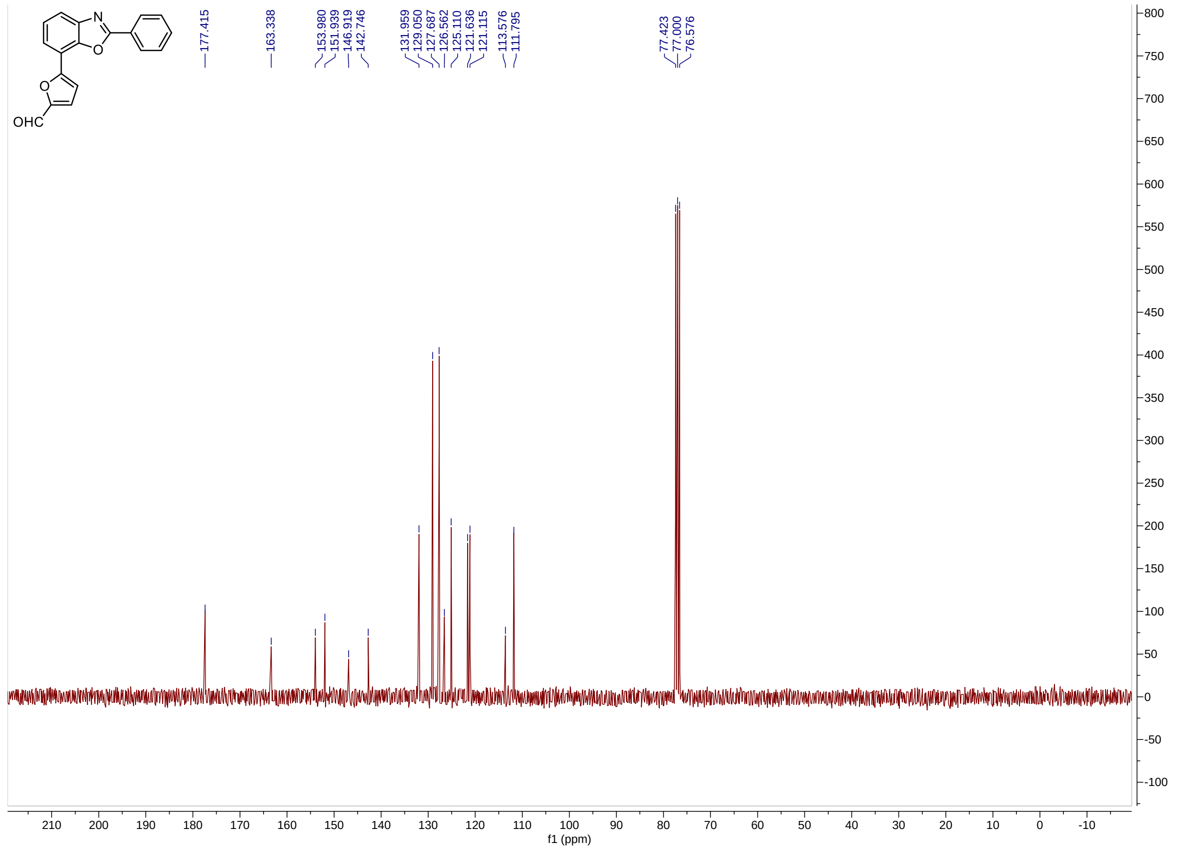

4q, ^{1}H NMR-300 MHz in $CDCl_3$ and ^{13}C NMR-75 MHz in $CDCl_3$

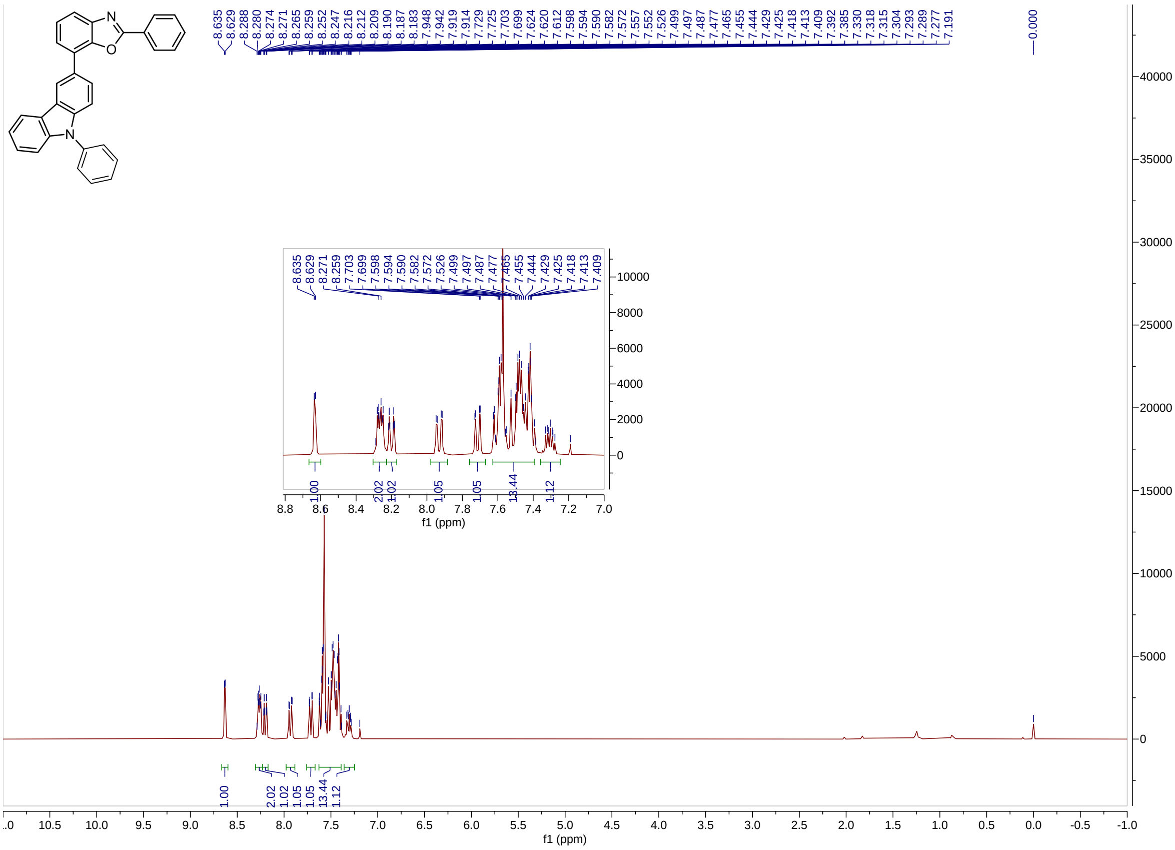

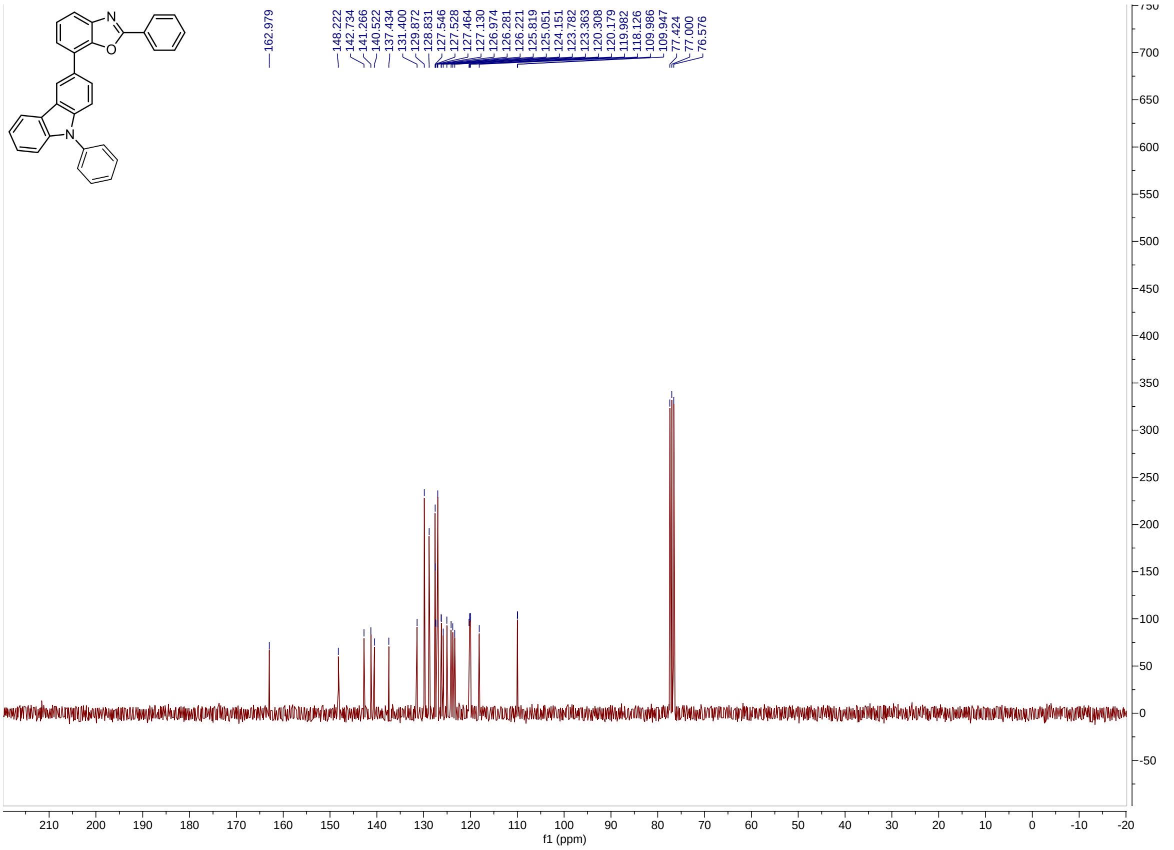

4r, ^{1}H NMR-300 MHz in $CDCl_3$ and ^{13}C NMR-75 MHz in $CDCl_3$

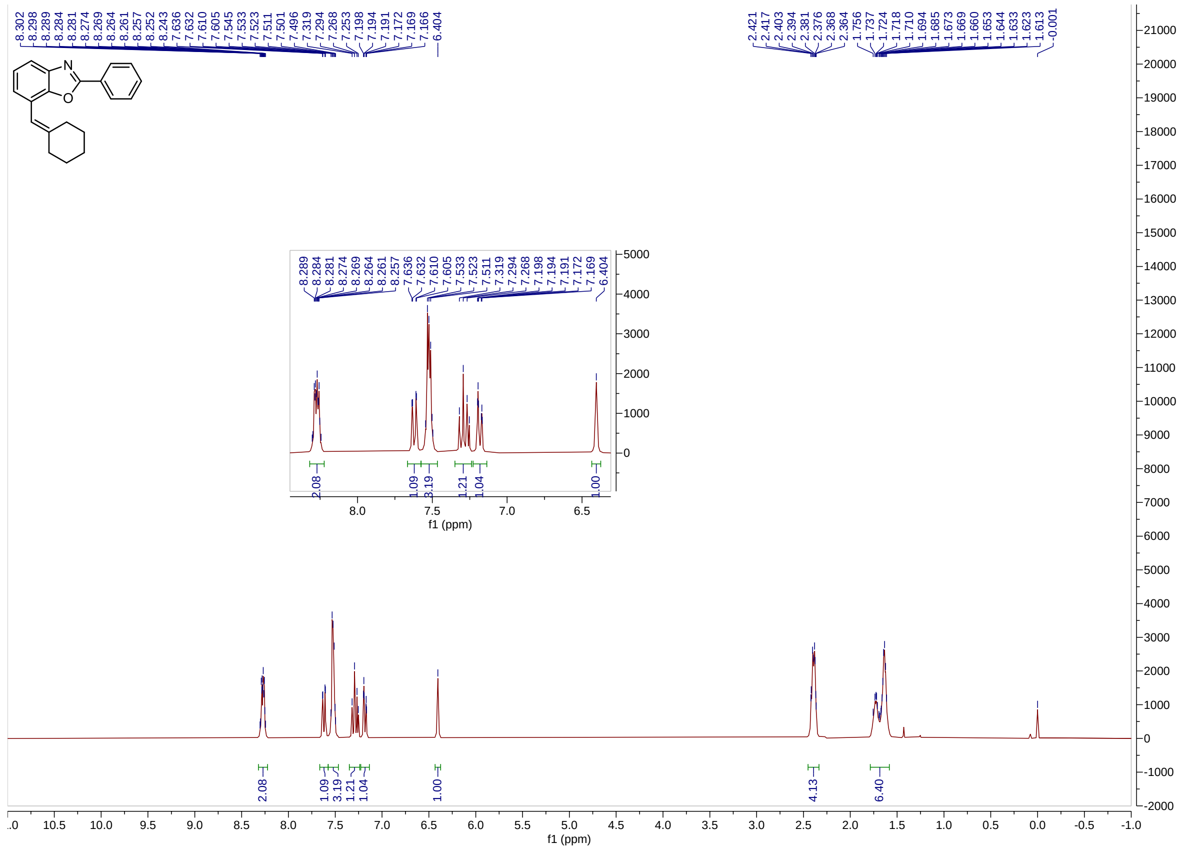

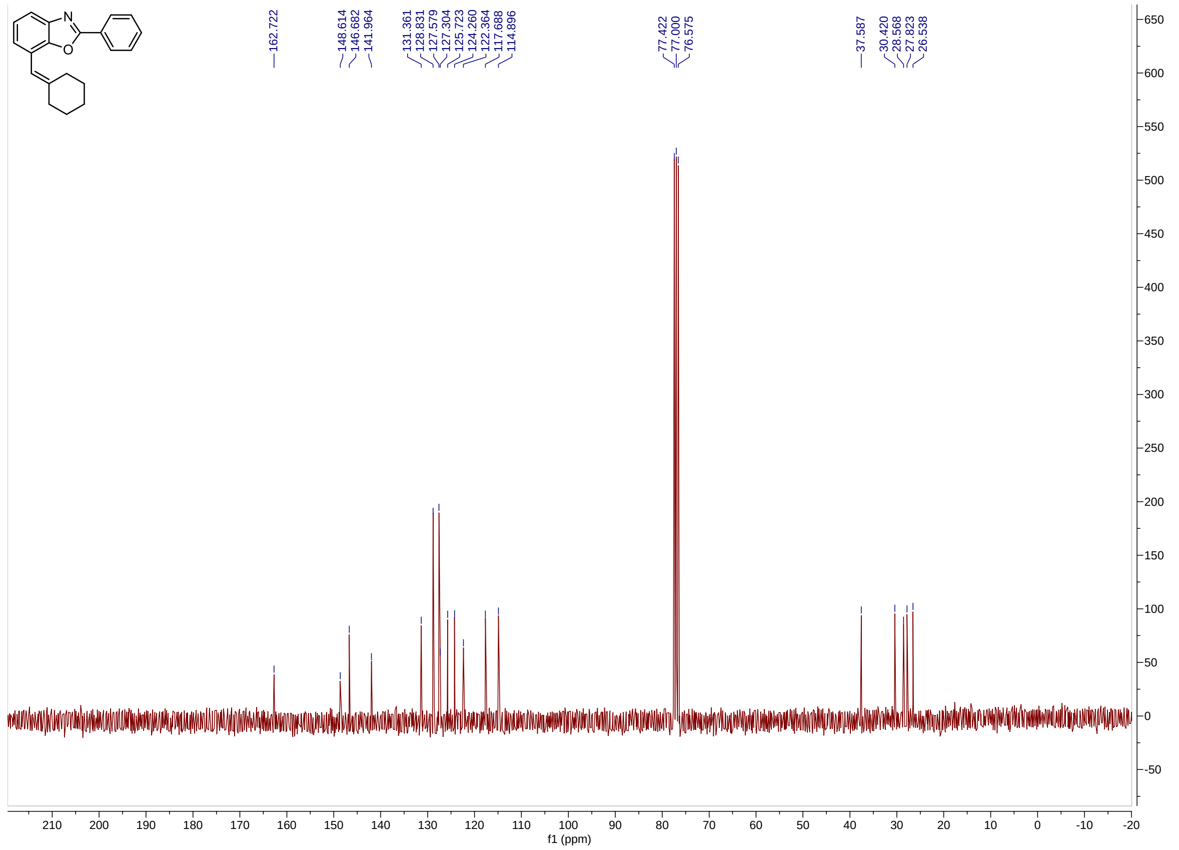

4s, ^{1}H NMR-300 MHz in $CDCl_3$ and ^{13}C NMR-75 MHz in $CDCl_3$

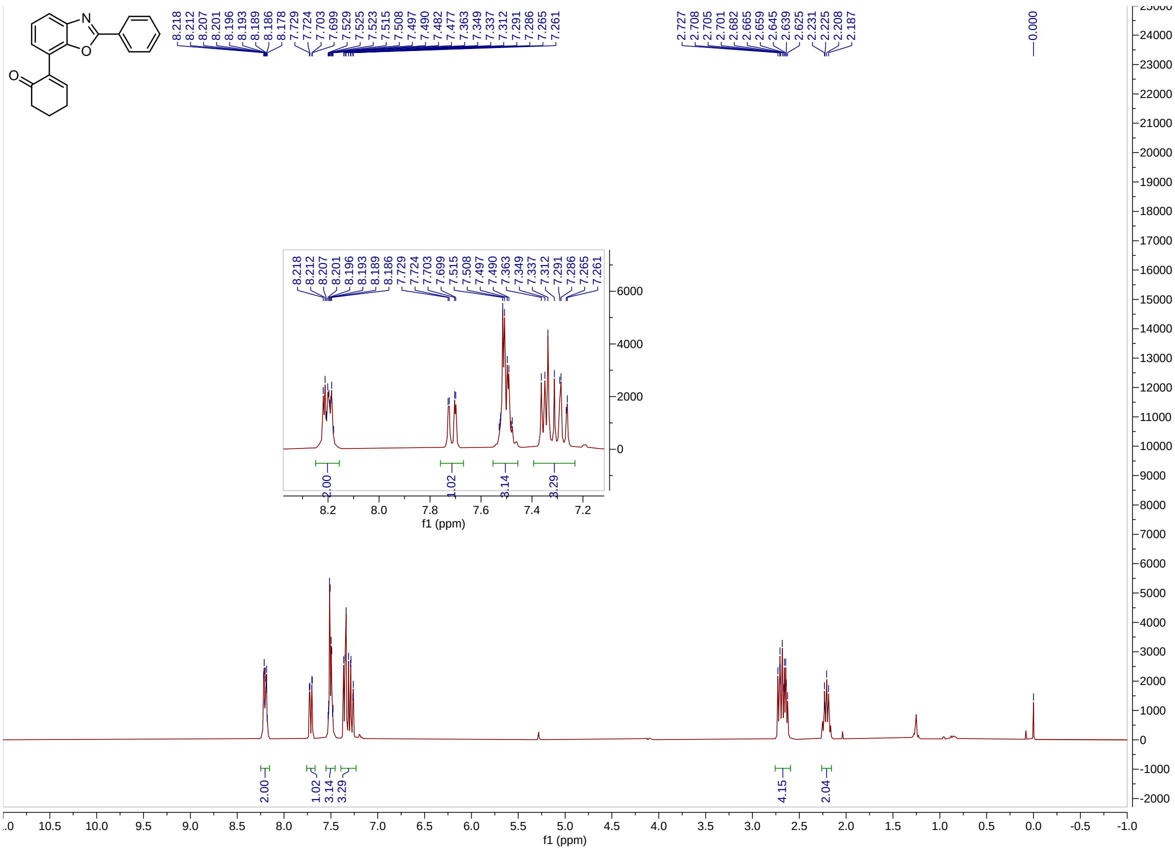

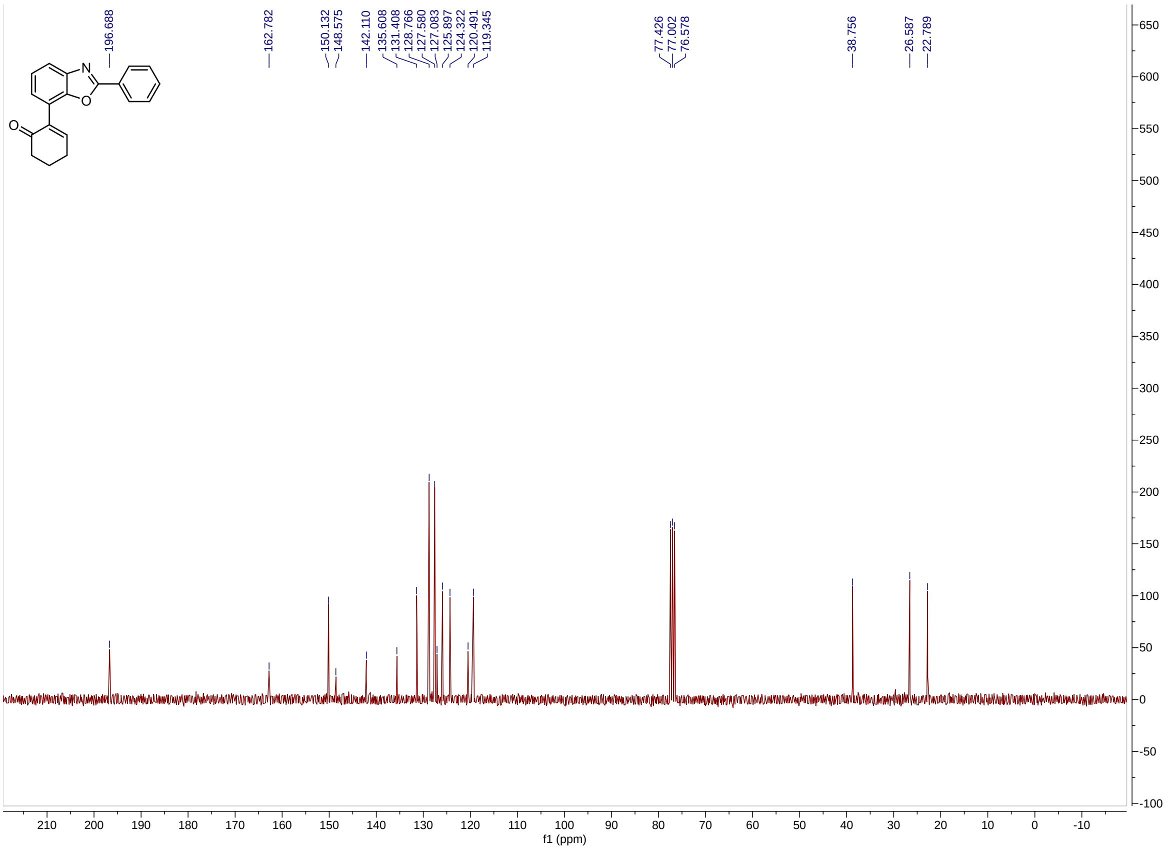

4t, ^{1}H NMR-300 MHz in $CDCl_3$ and ^{13}C NMR-75 MHz in $CDCl_3$

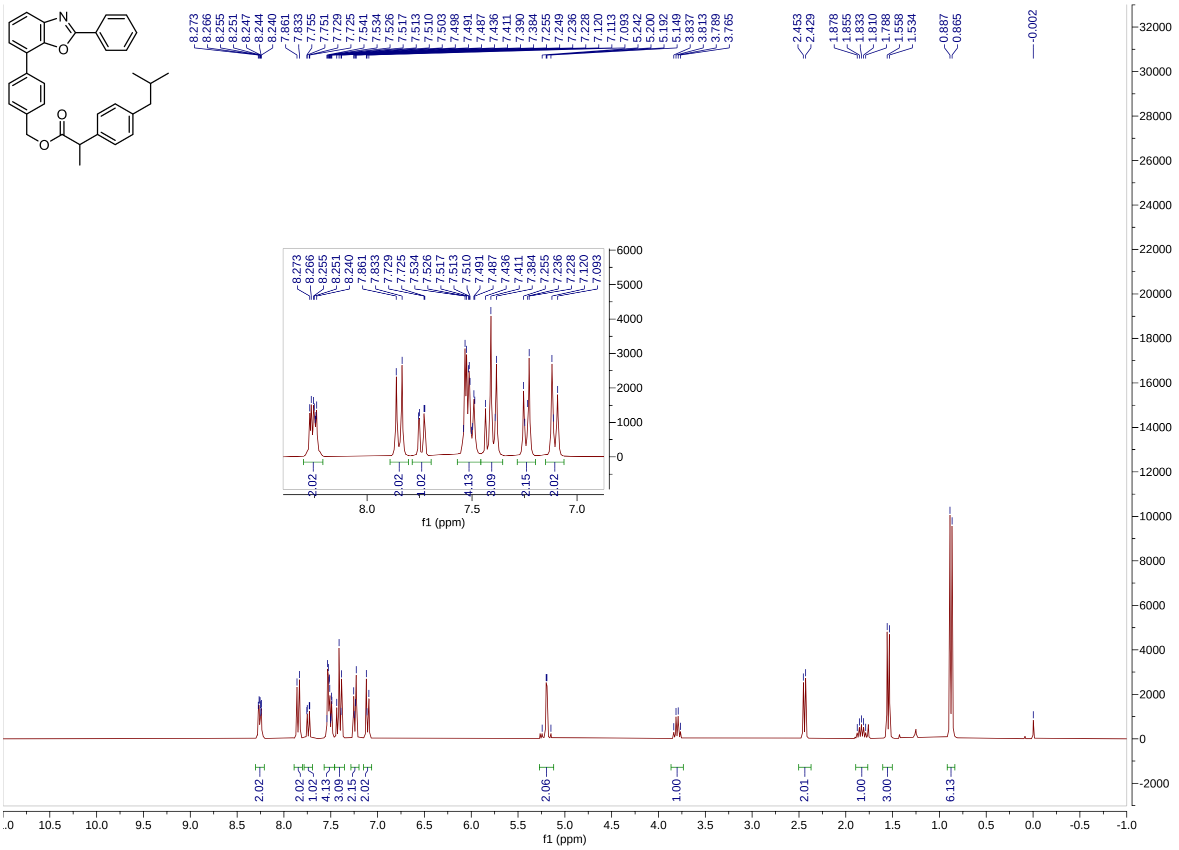

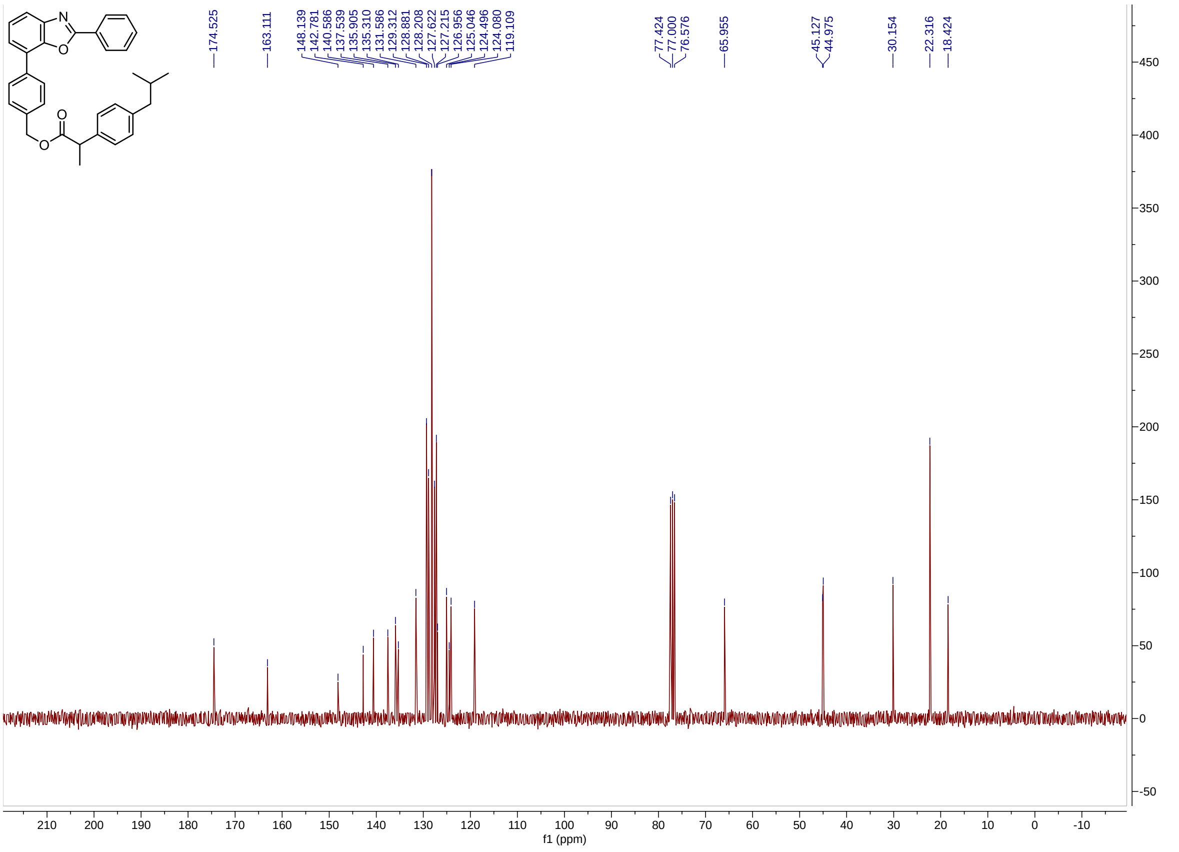

4u, ^{1}H NMR-300 MHz in $CDCl_3$ and ^{13}C NMR-75 MHz in $CDCl_3$

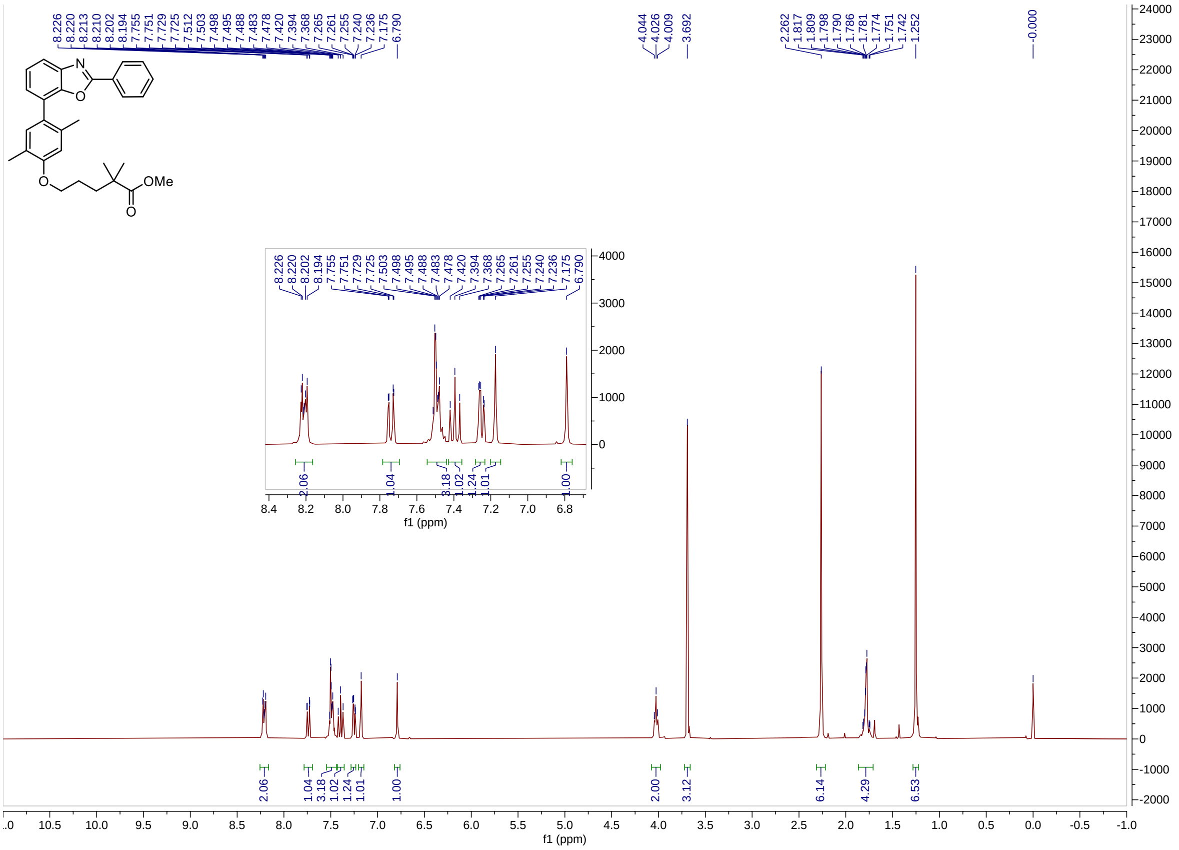

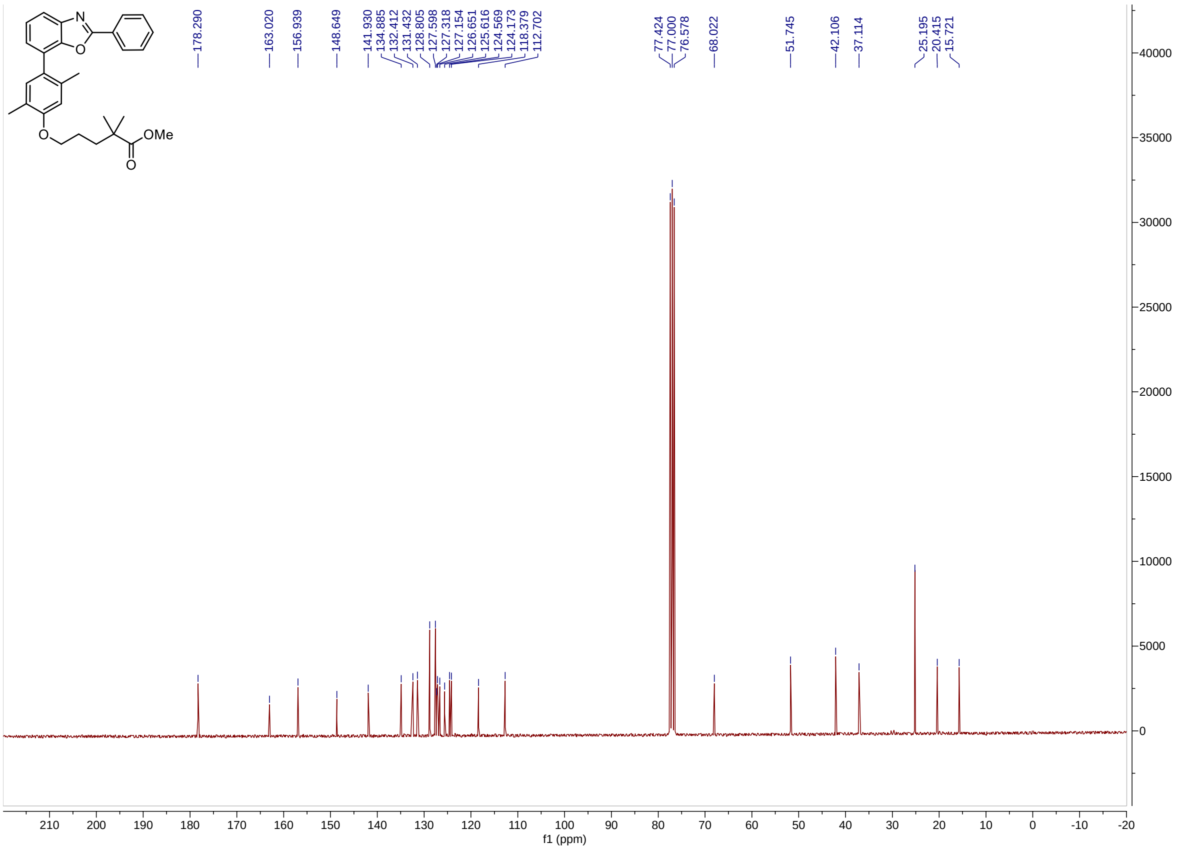

4v, ^{1}H NMR-300 MHz in $CDCl_3$ and ^{13}C NMR-75 MHz in $CDCl_3$

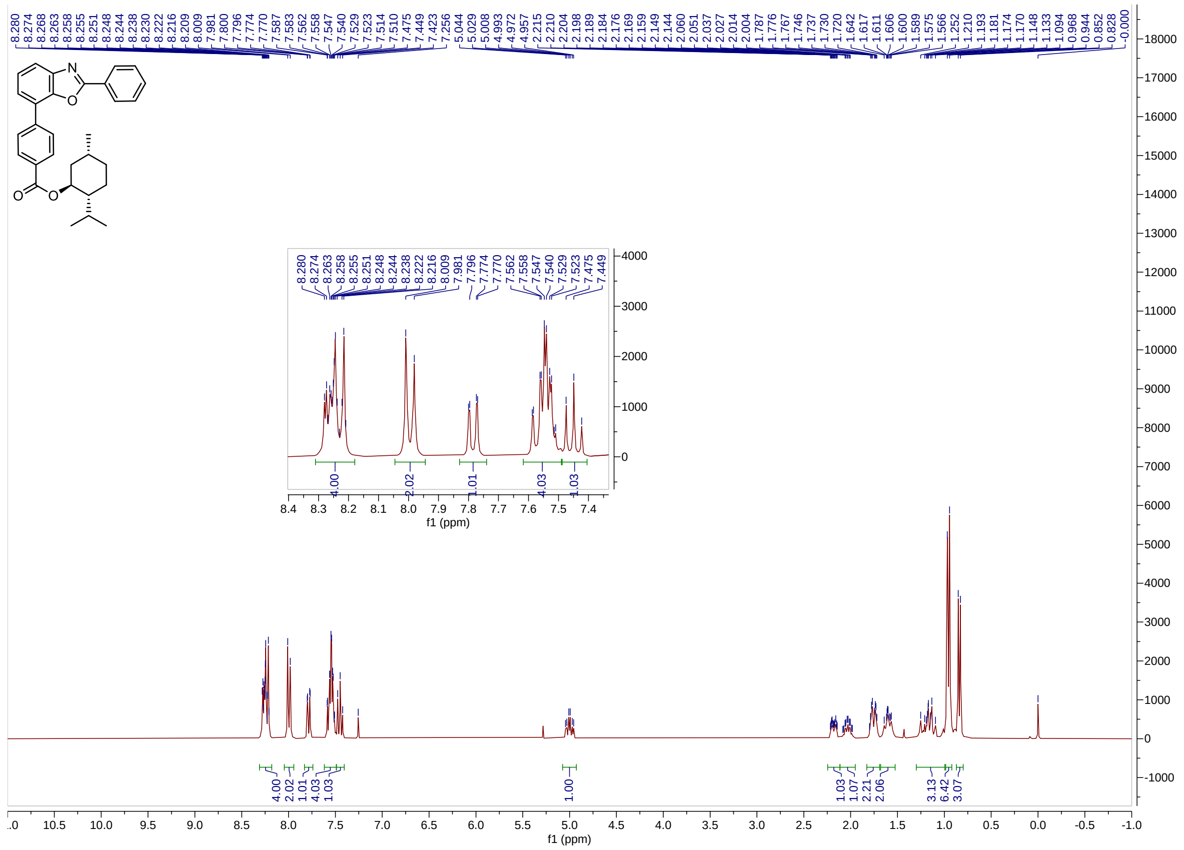

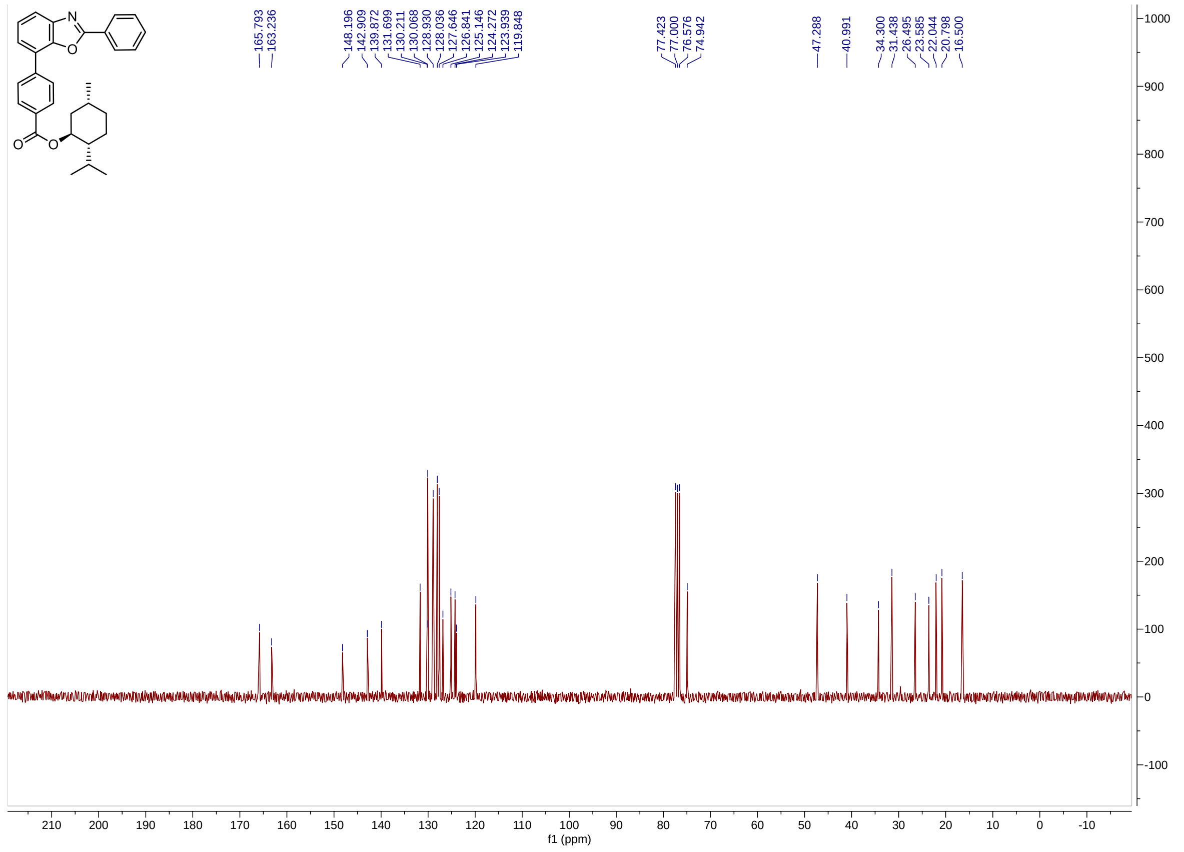

5a, ^{1}H NMR-300 MHz in $CDCl_3$ and ^{13}C NMR-75 MHz in $CDCl_3$

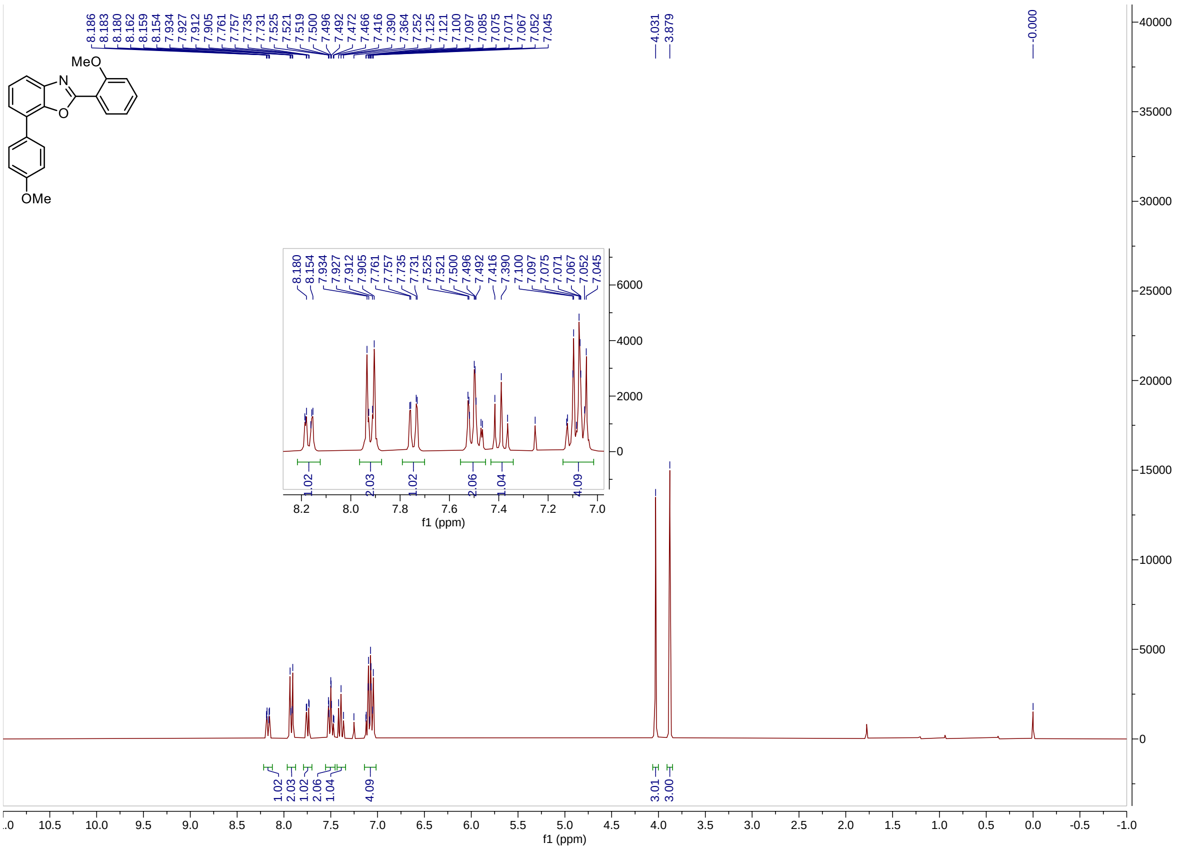

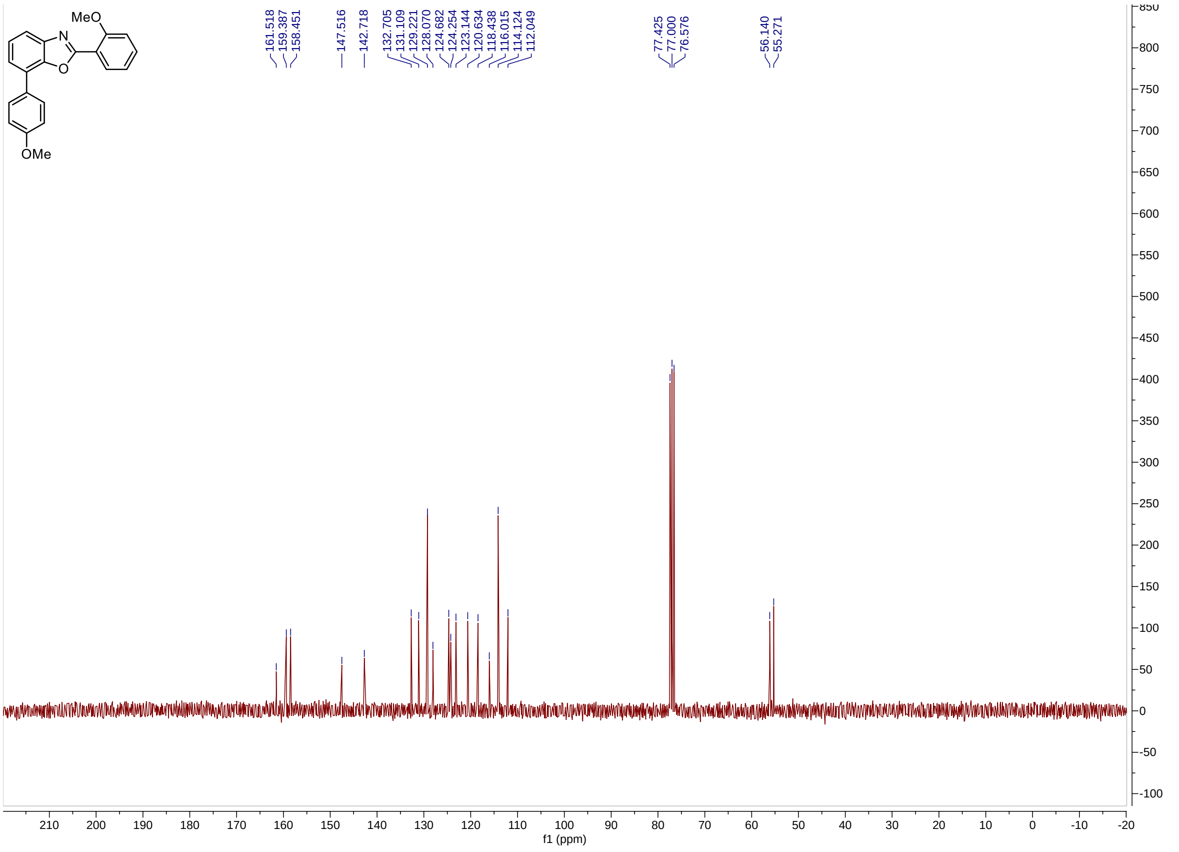

5b, ^{1}H NMR-300 MHz in $CDCl_3$ and ^{13}C NMR-75 MHz in $CDCl_3$

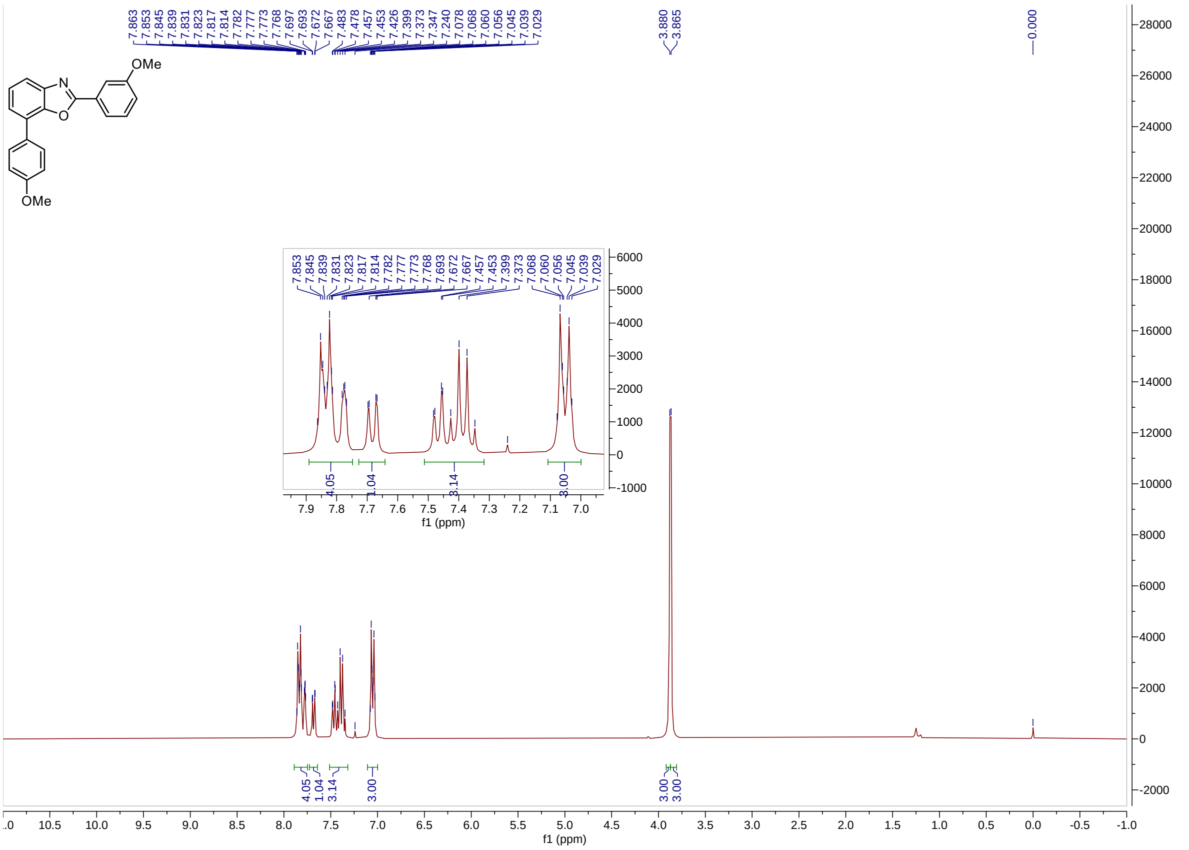

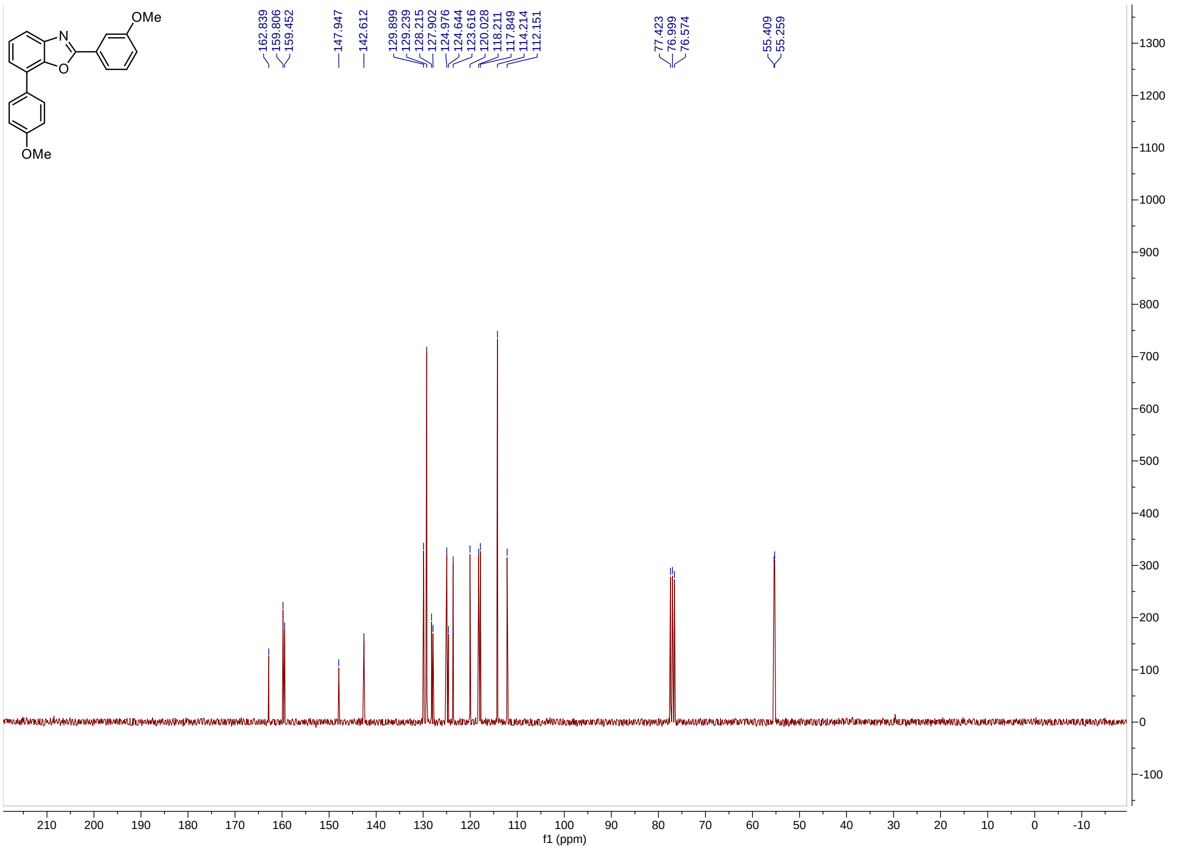

5c, ^{1}H NMR-300 MHz in $CDCl_3$ and ^{13}C NMR-75 MHz in $CDCl_3$

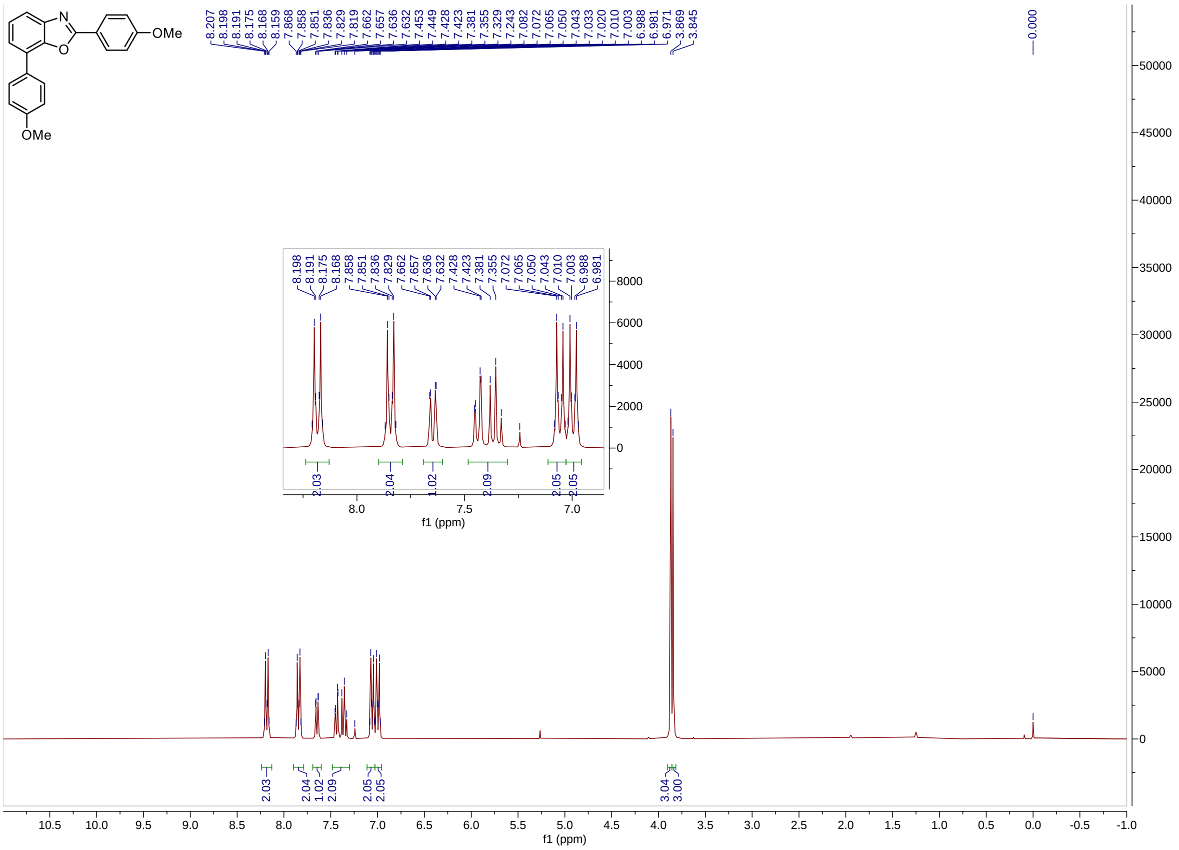

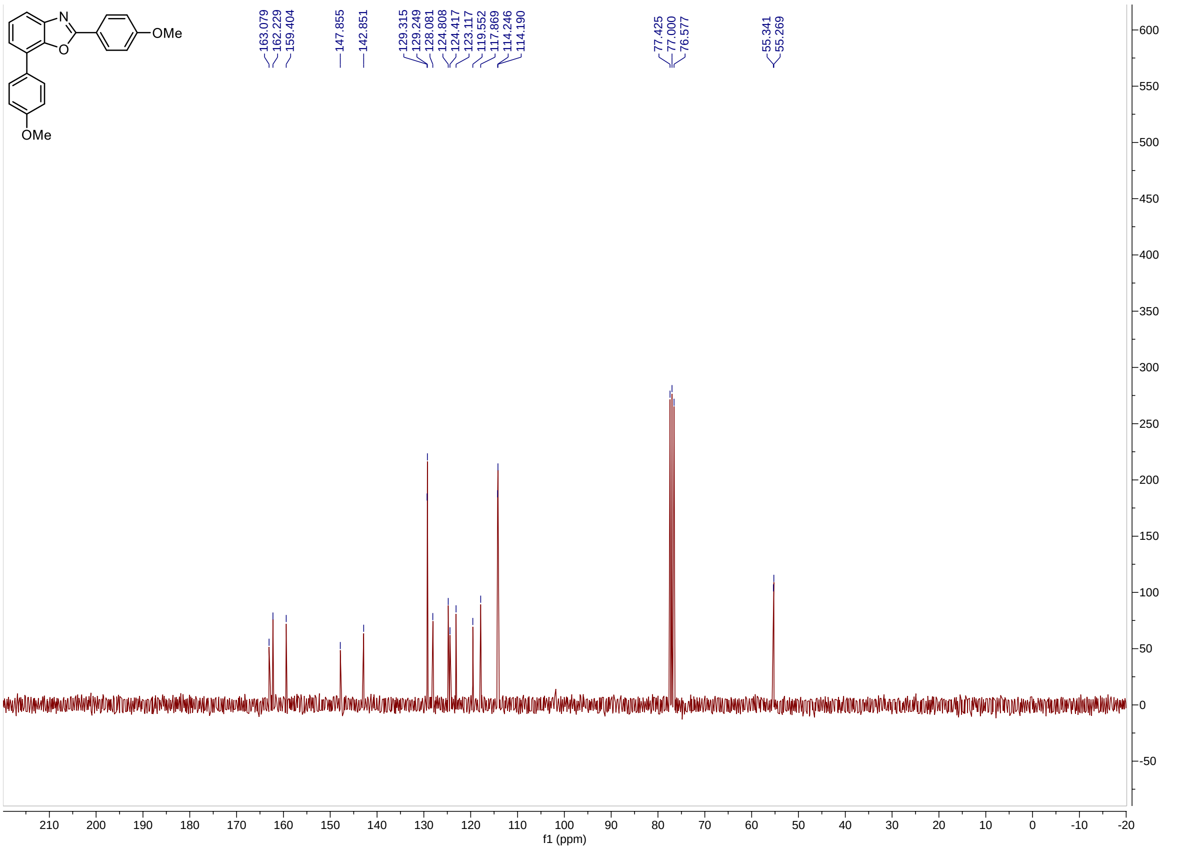

5d, ^{1}H NMR-300 MHz in $CDCl_3$ and ^{13}C NMR-75 MHz in $CDCl_3$

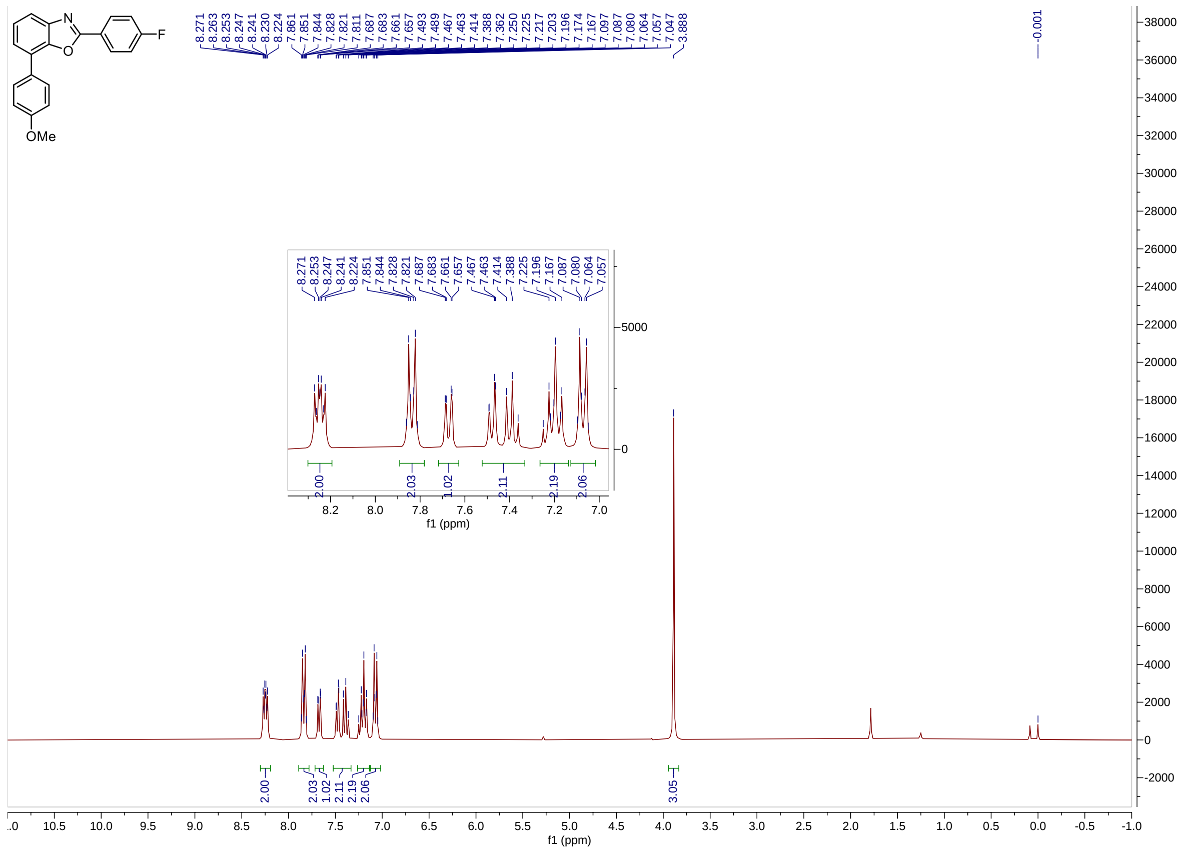

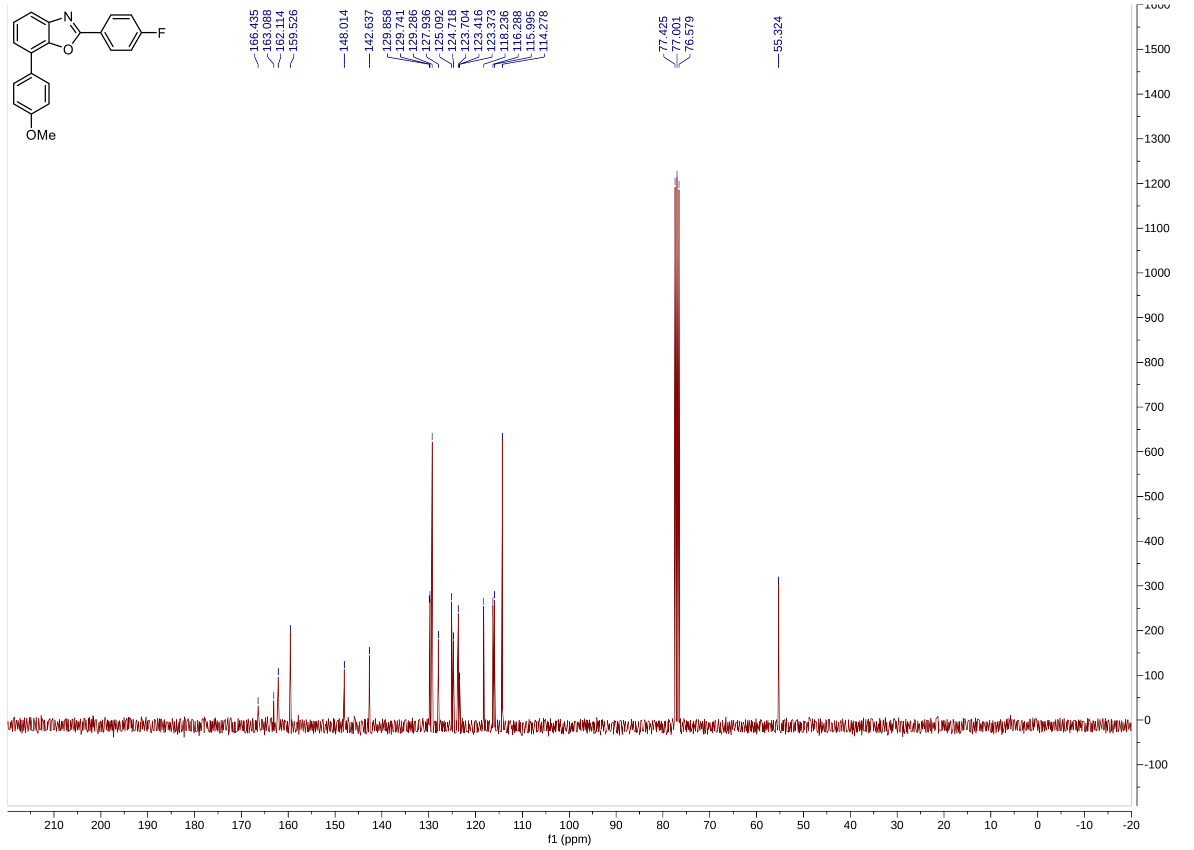

5e, ^{1}H NMR-300 MHz in $CDCl_3$ and ^{13}C NMR-75 MHz in $CDCl_3$

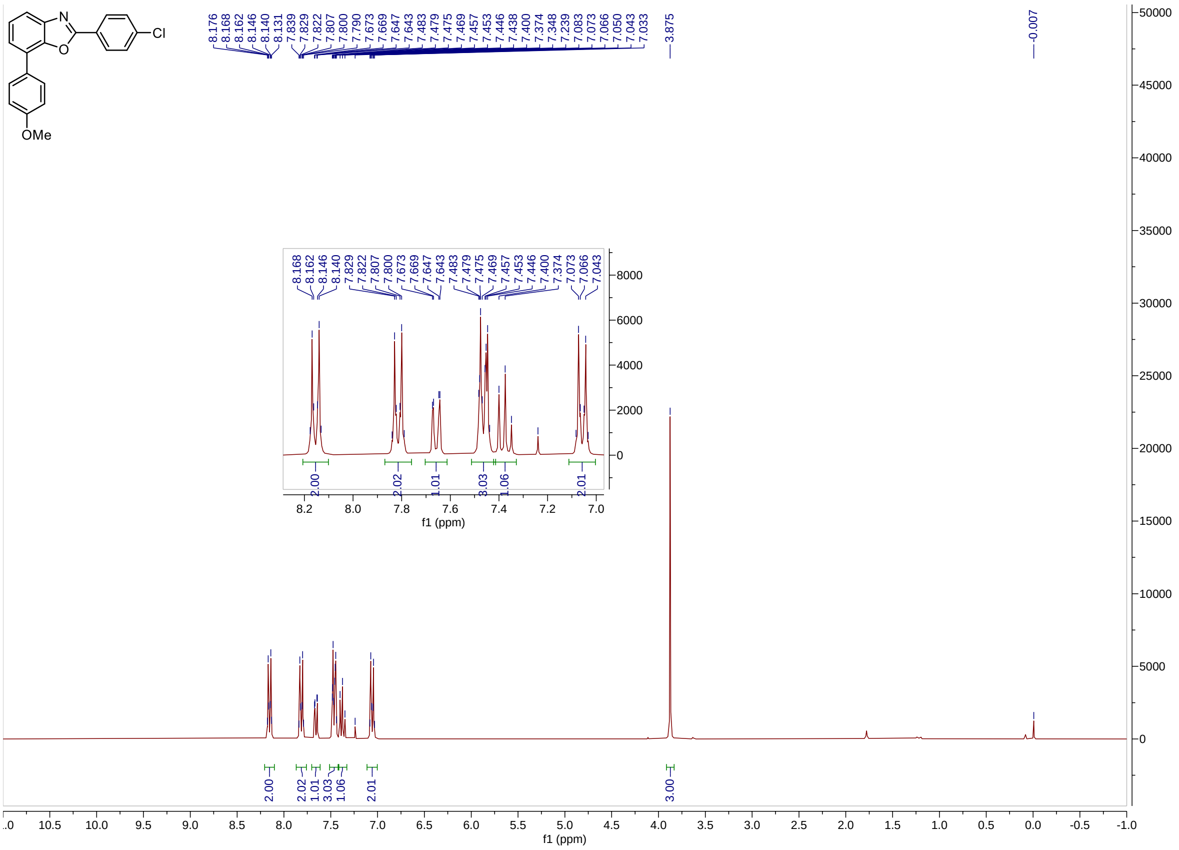

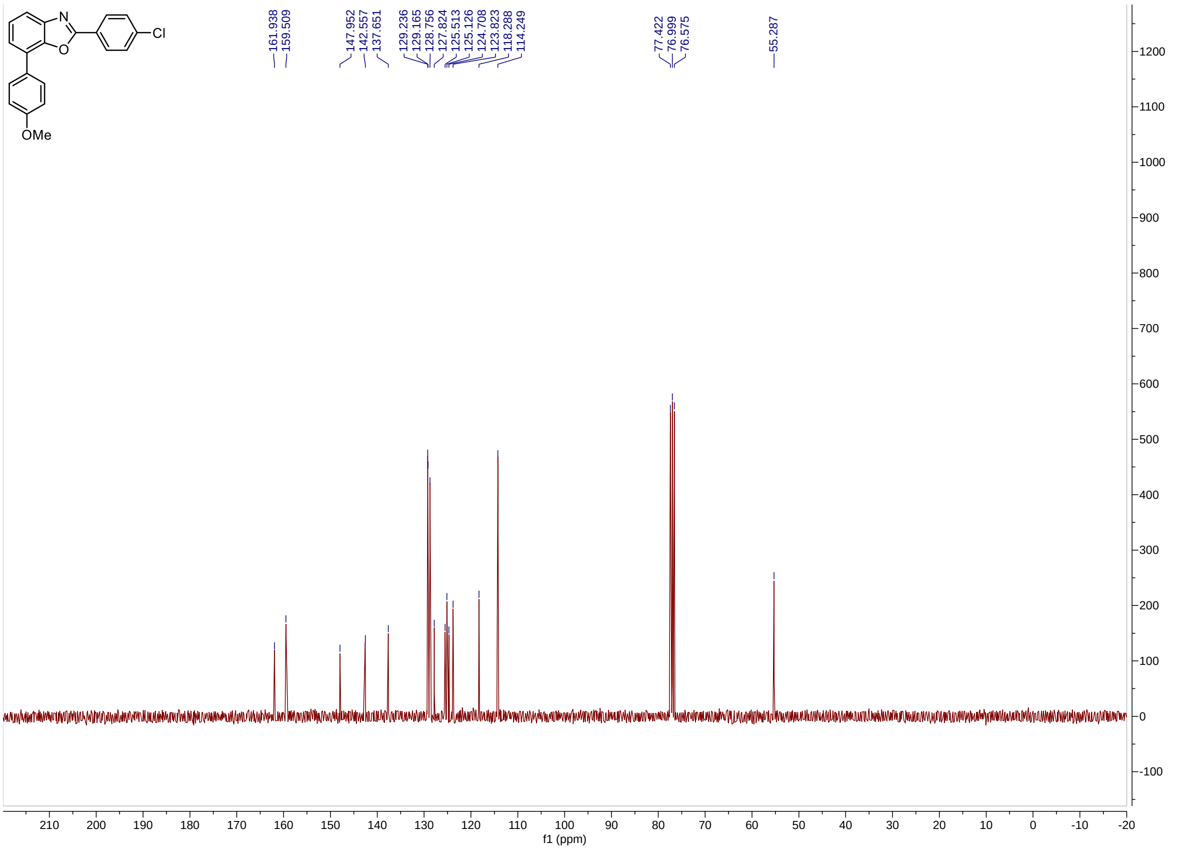

5f, ^{1}H NMR-300 MHz in $CDCl_3$ and ^{13}C NMR-75 MHz in $CDCl_3$

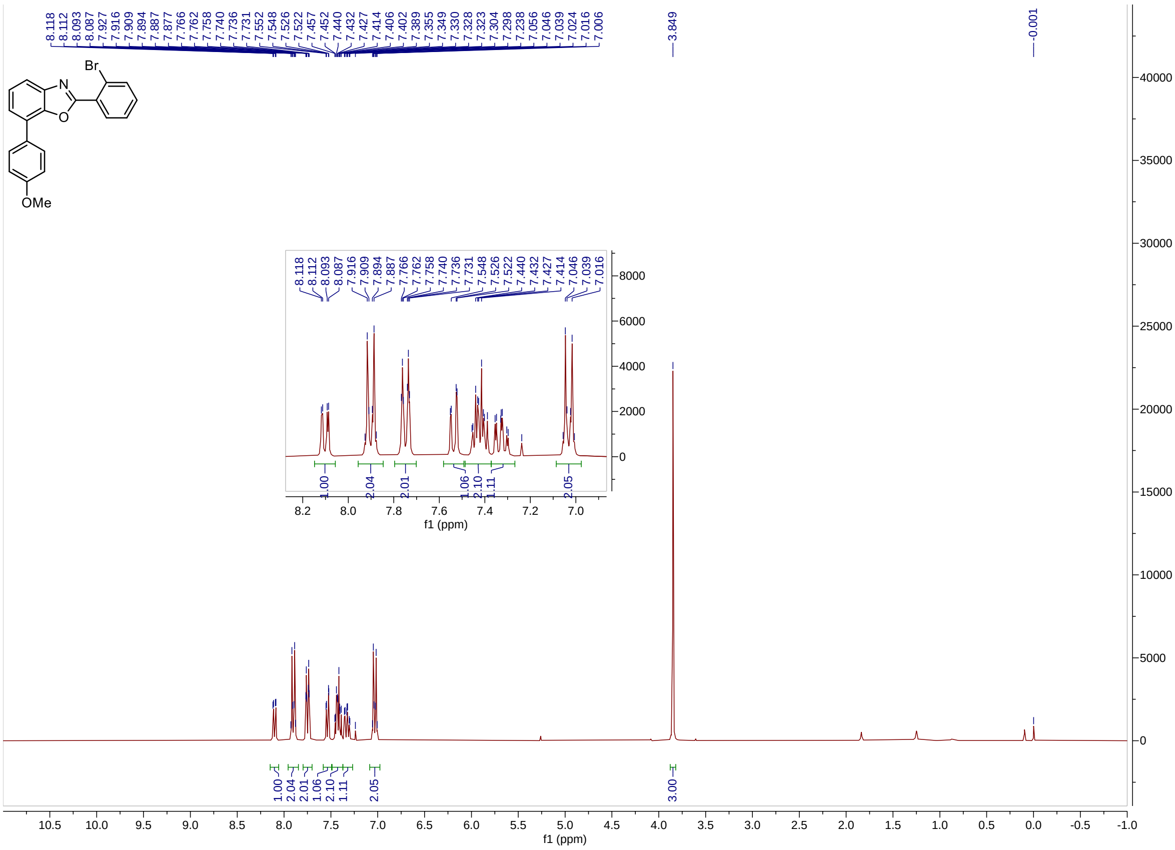

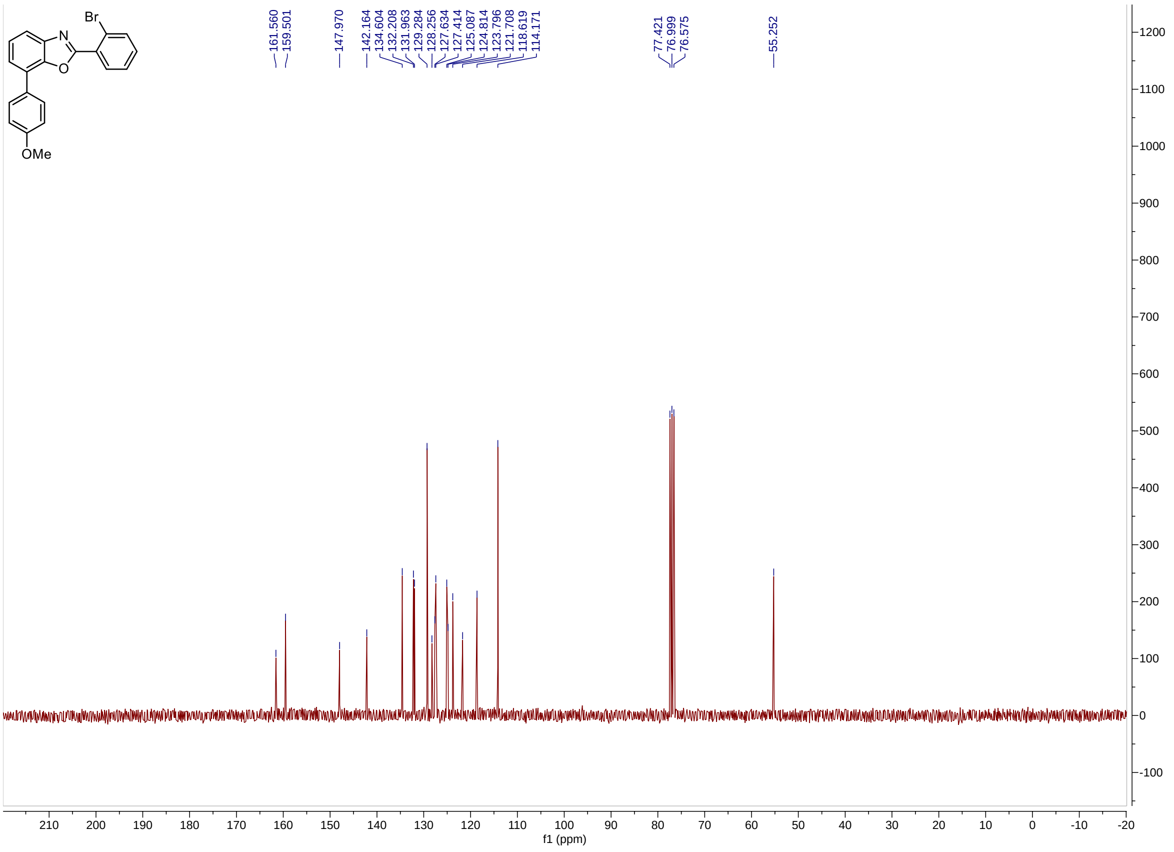

5g, ^{1}H NMR-300 MHz in $CDCl_3$ and ^{13}C NMR-75 MHz in $CDCl_3$

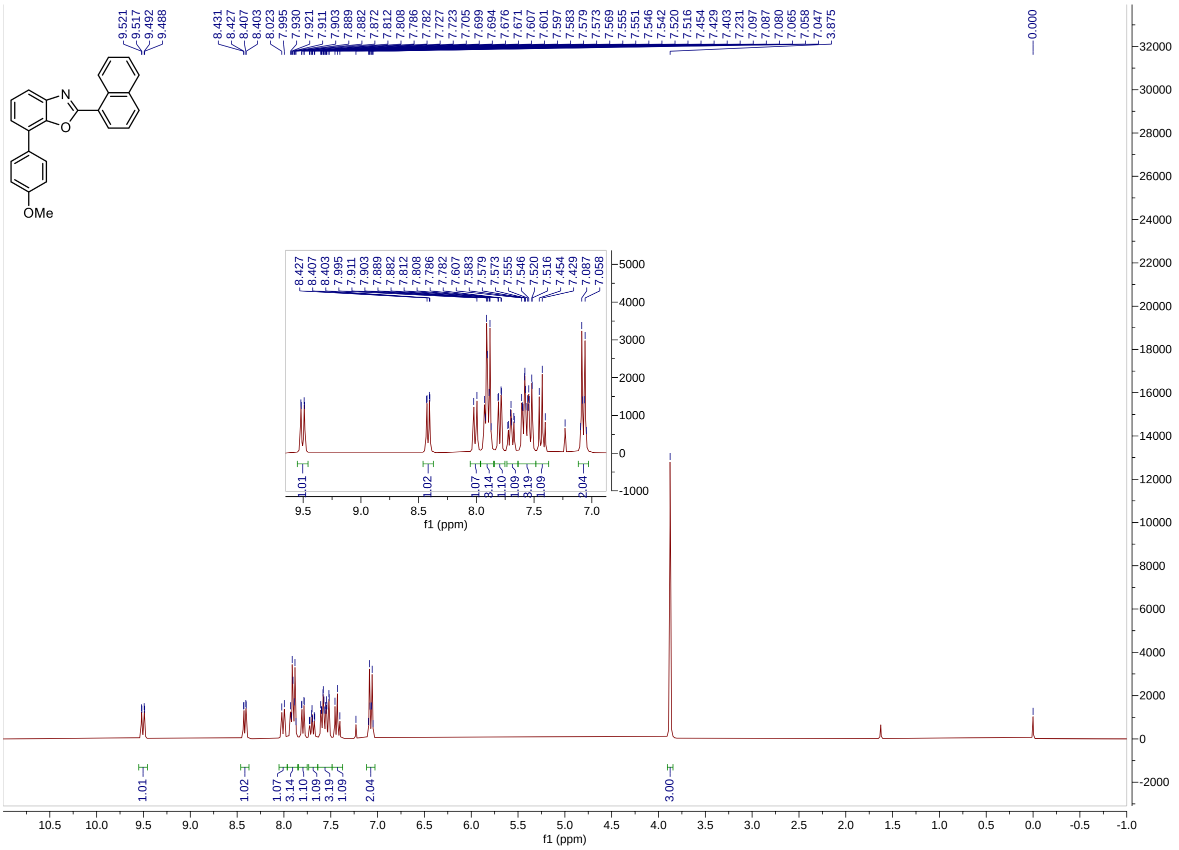

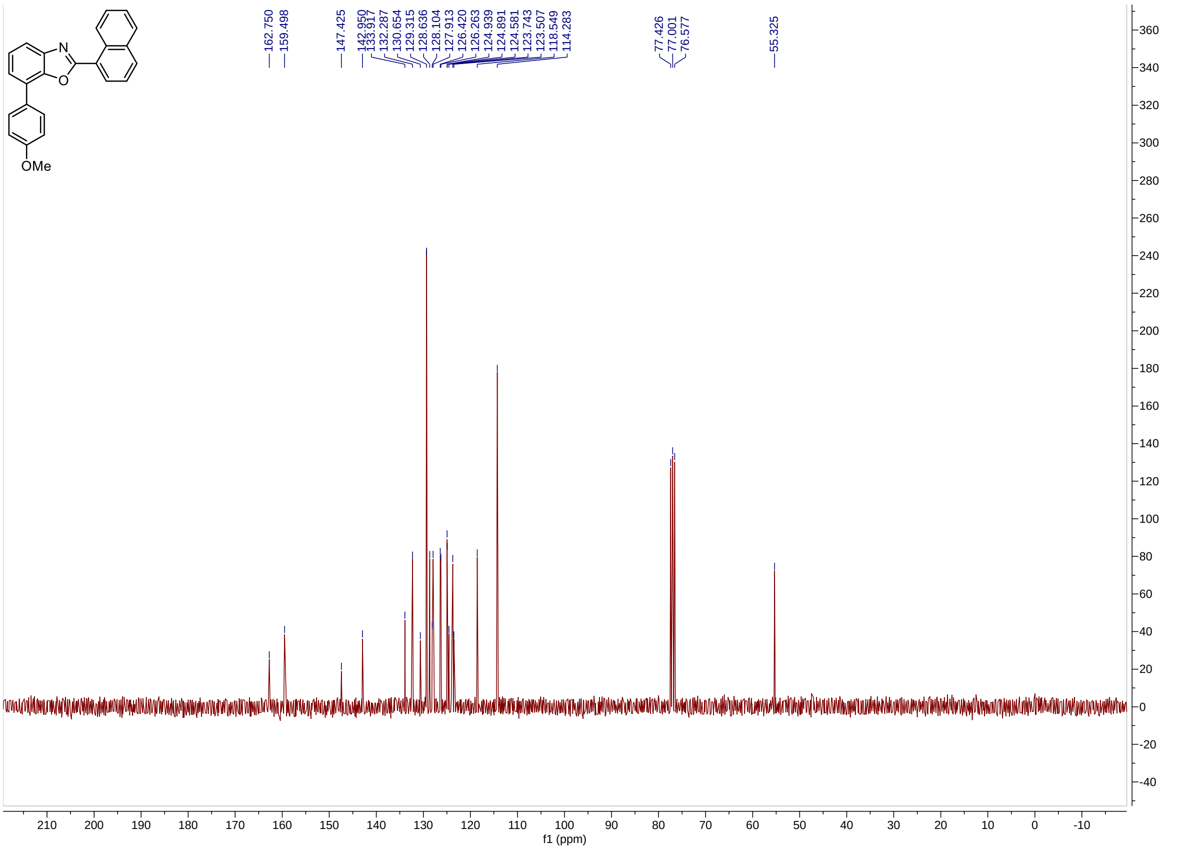

5h, ^{1}H NMR-300 MHz in $CDCl_3$ and ^{13}C NMR-75 MHz in $CDCl_3$

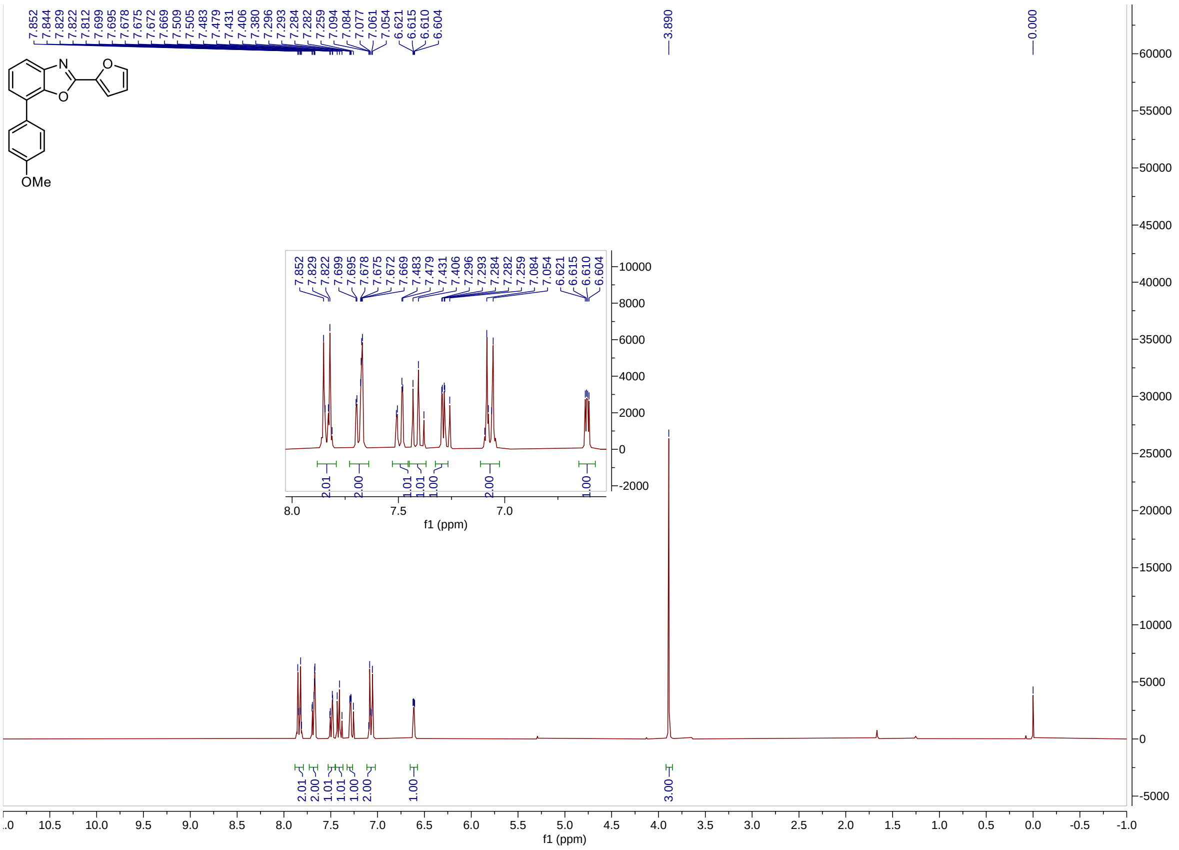

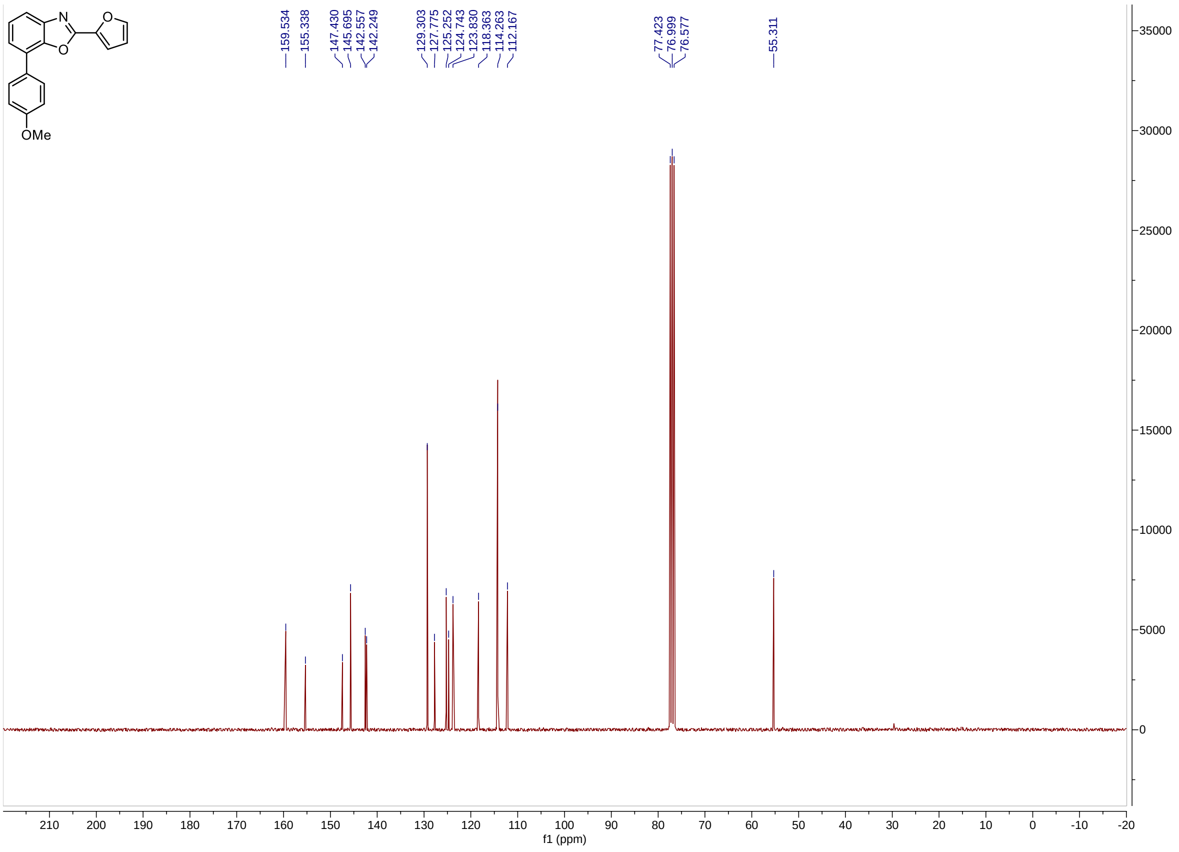

5i, ^{1}H NMR-300 MHz in $CDCl_3$ and ^{13}C NMR-75 MHz in $CDCl_3$

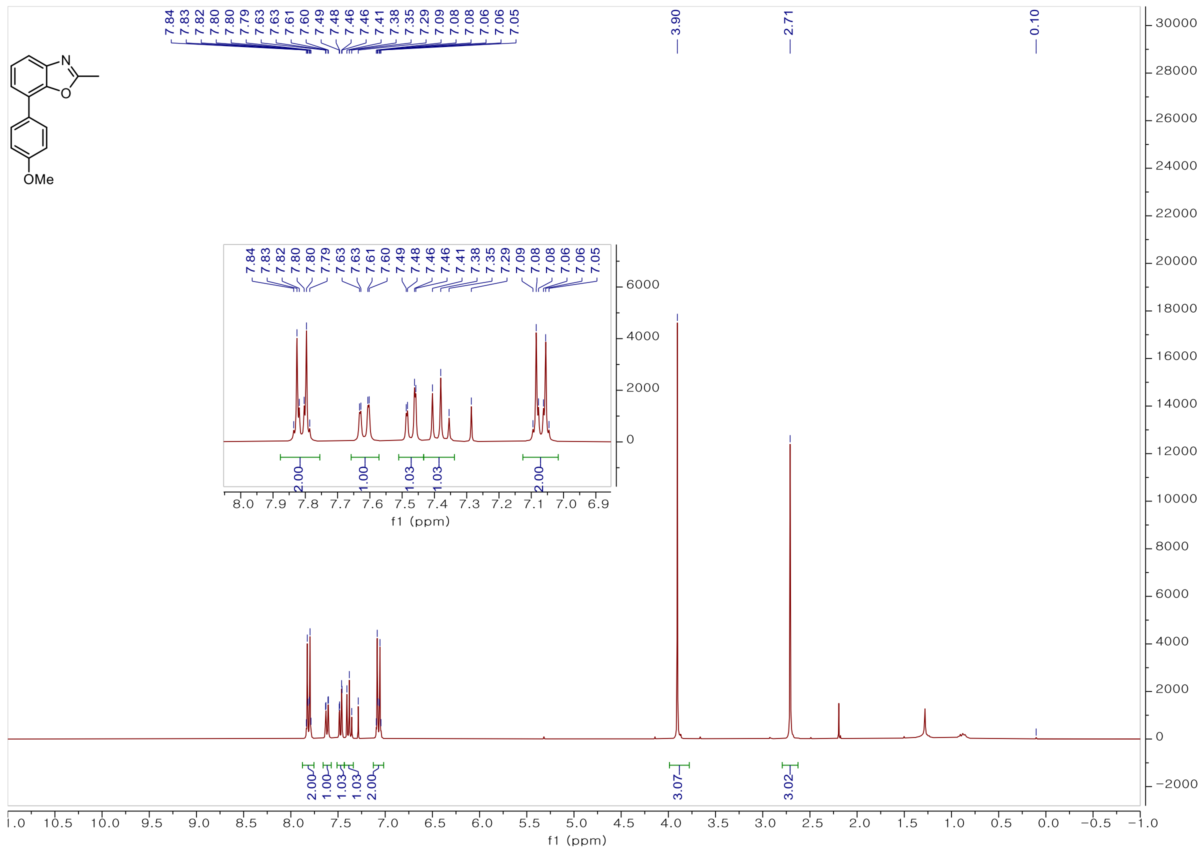

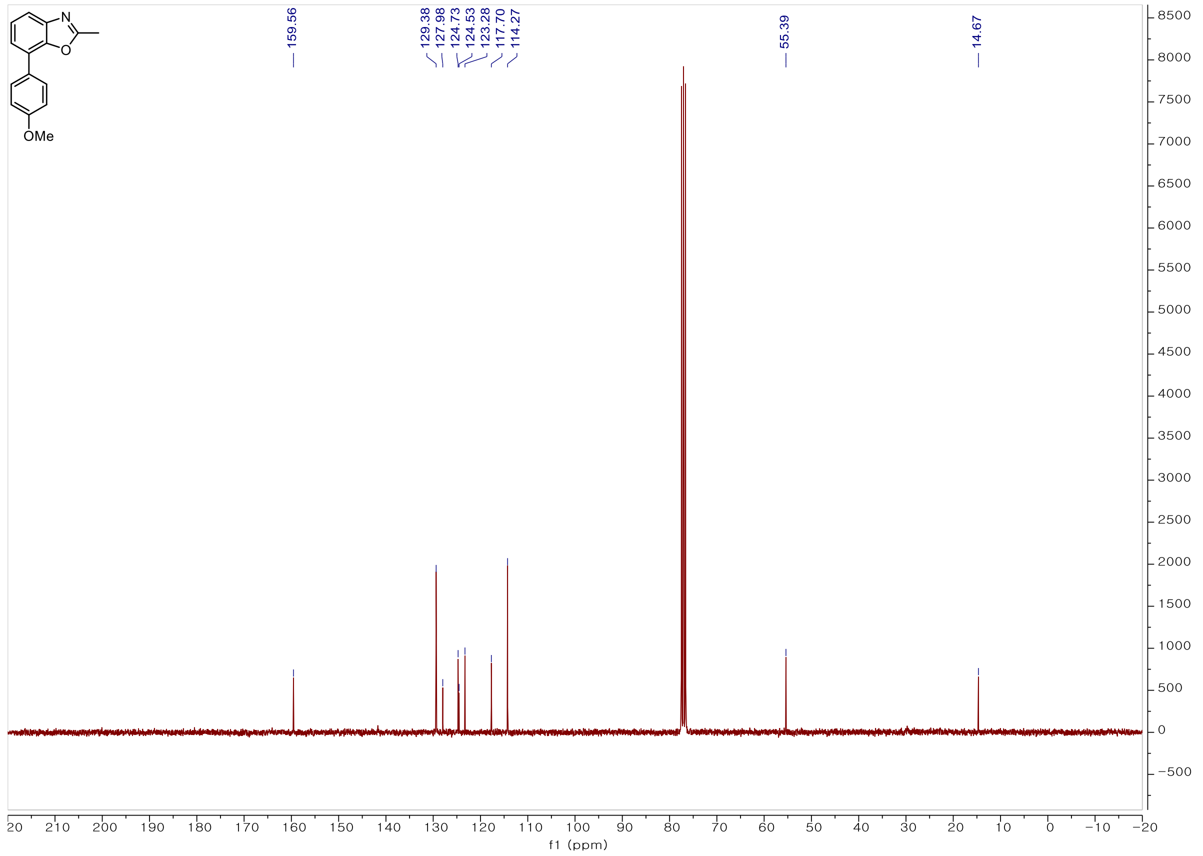

5j, ^{1}H NMR-300 MHz in $CDCl_3$ and ^{13}C NMR-75 MHz in $CDCl_3$

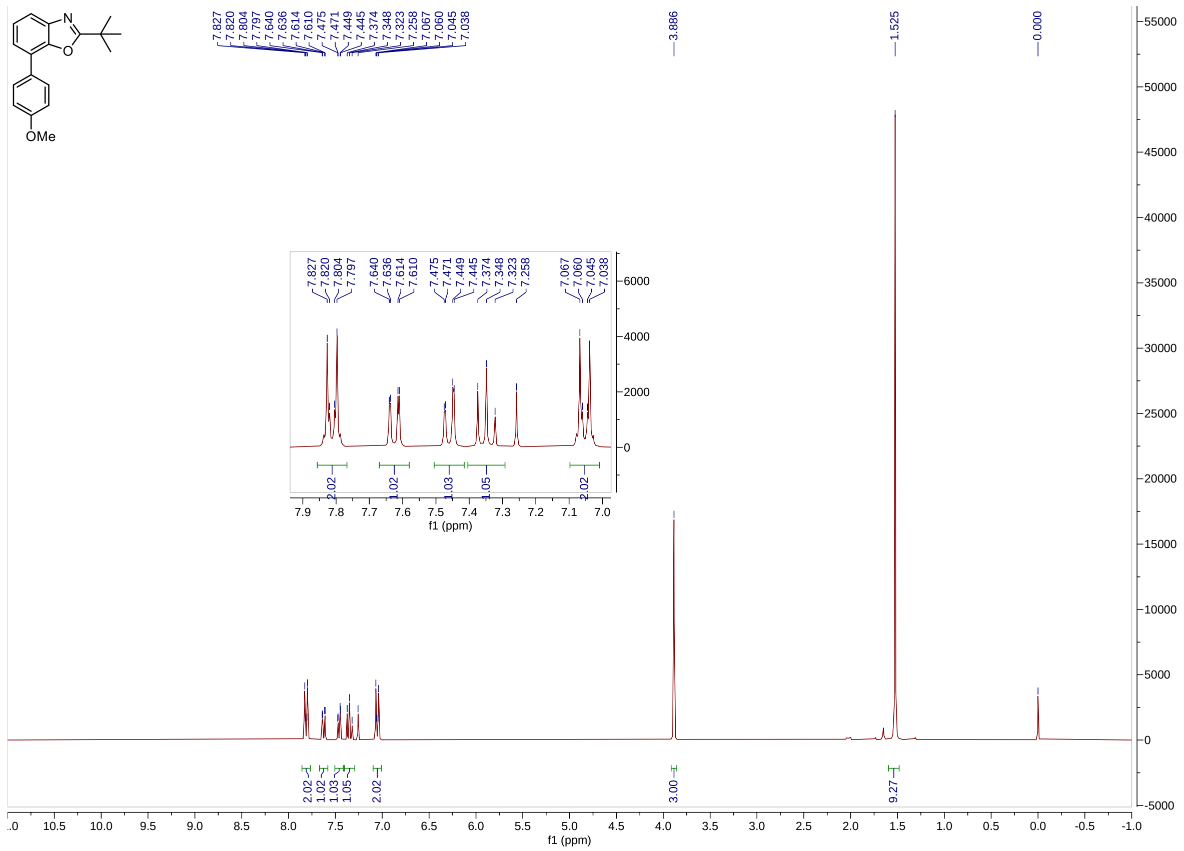

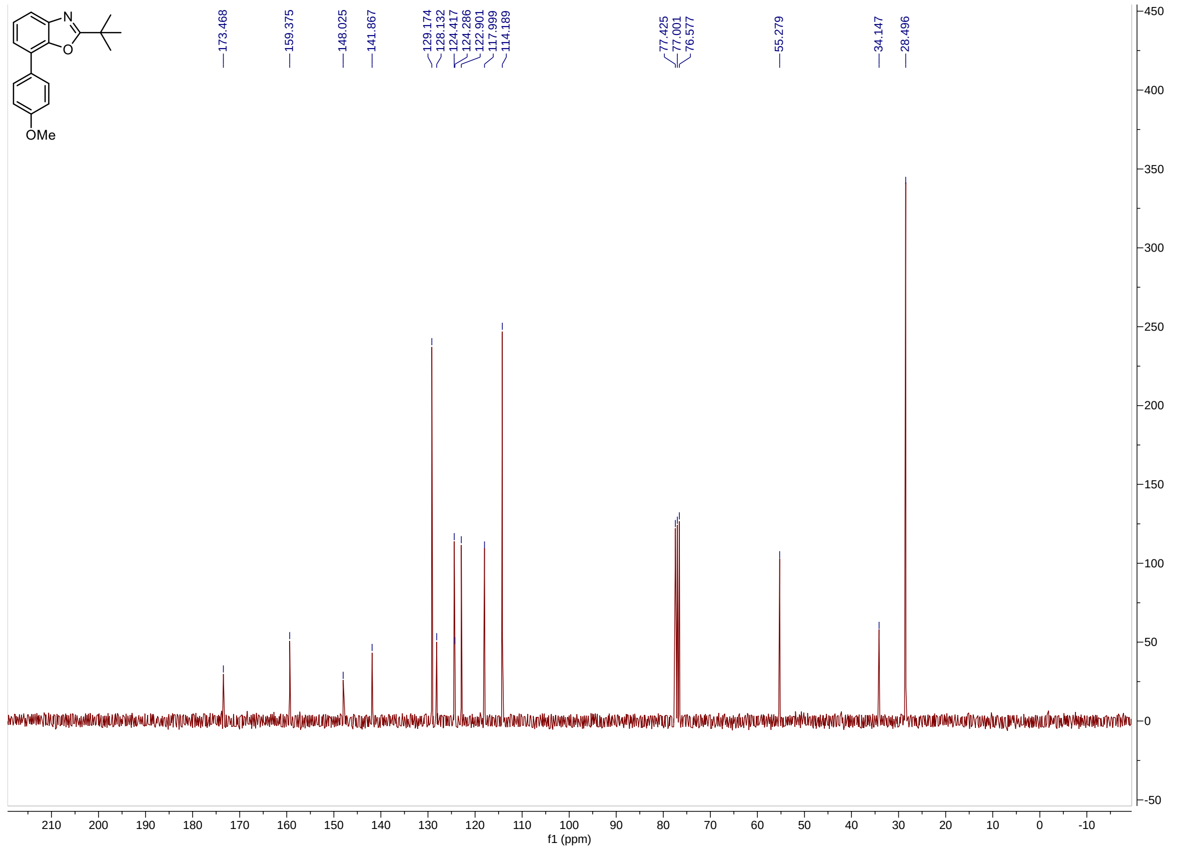

5k, ^{1}H NMR-300 MHz in $CDCl_3$ and ^{13}C NMR-75 MHz in $CDCl_3$

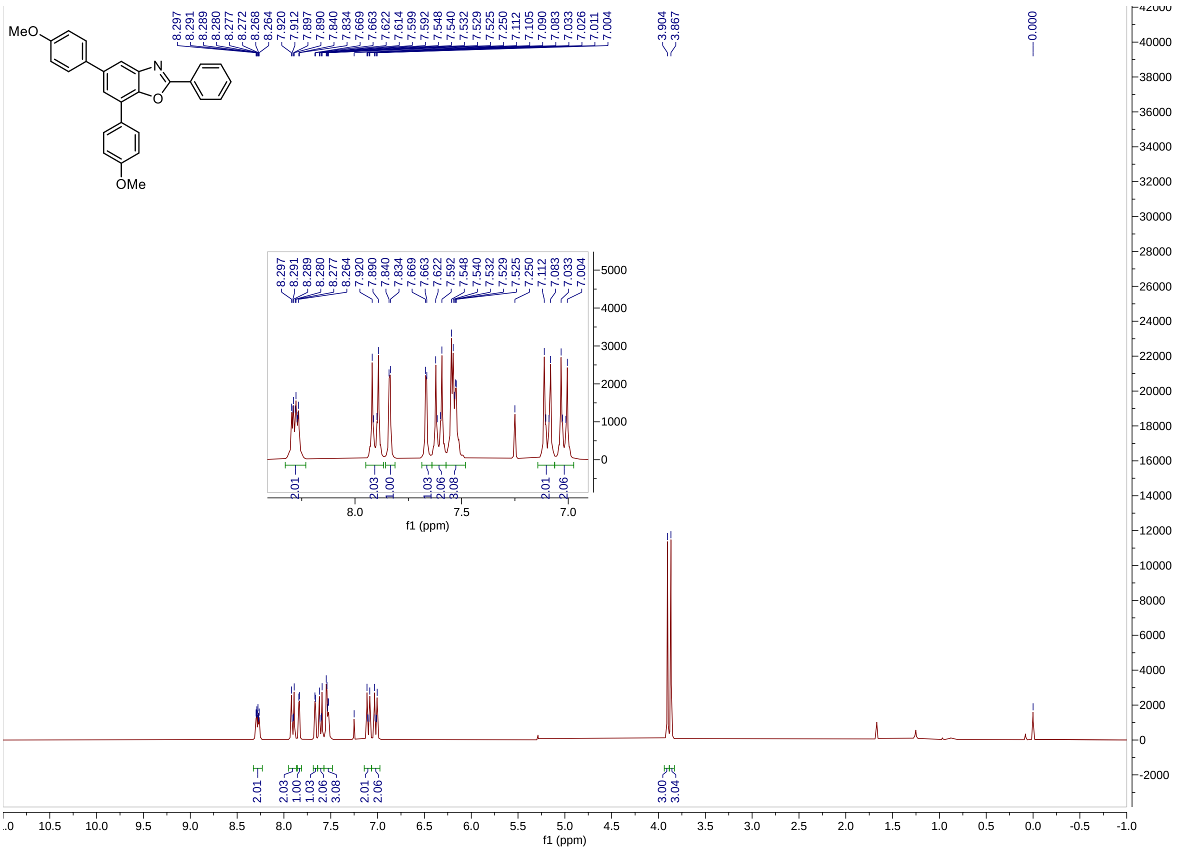

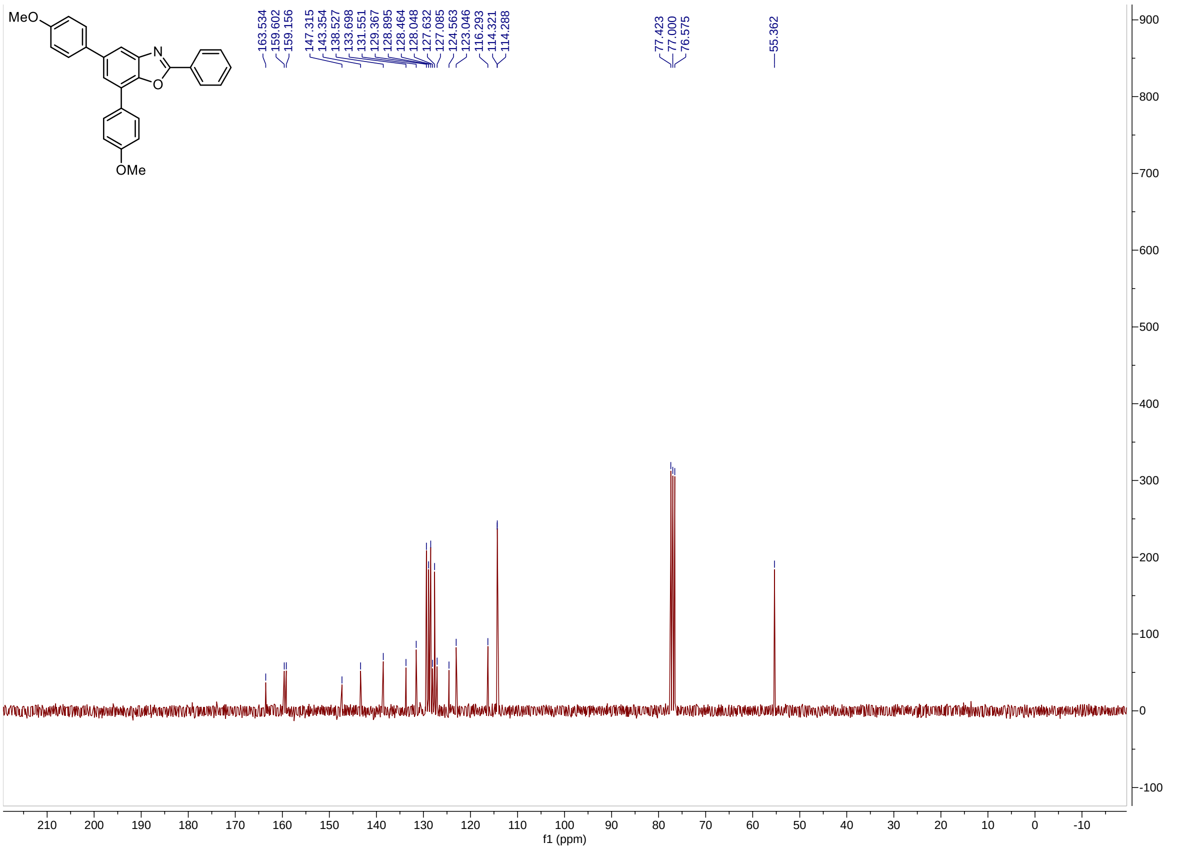

5l, ^{1}H NMR-300 MHz in $CDCl_3$ and ^{13}C NMR-75 MHz in $CDCl_3$

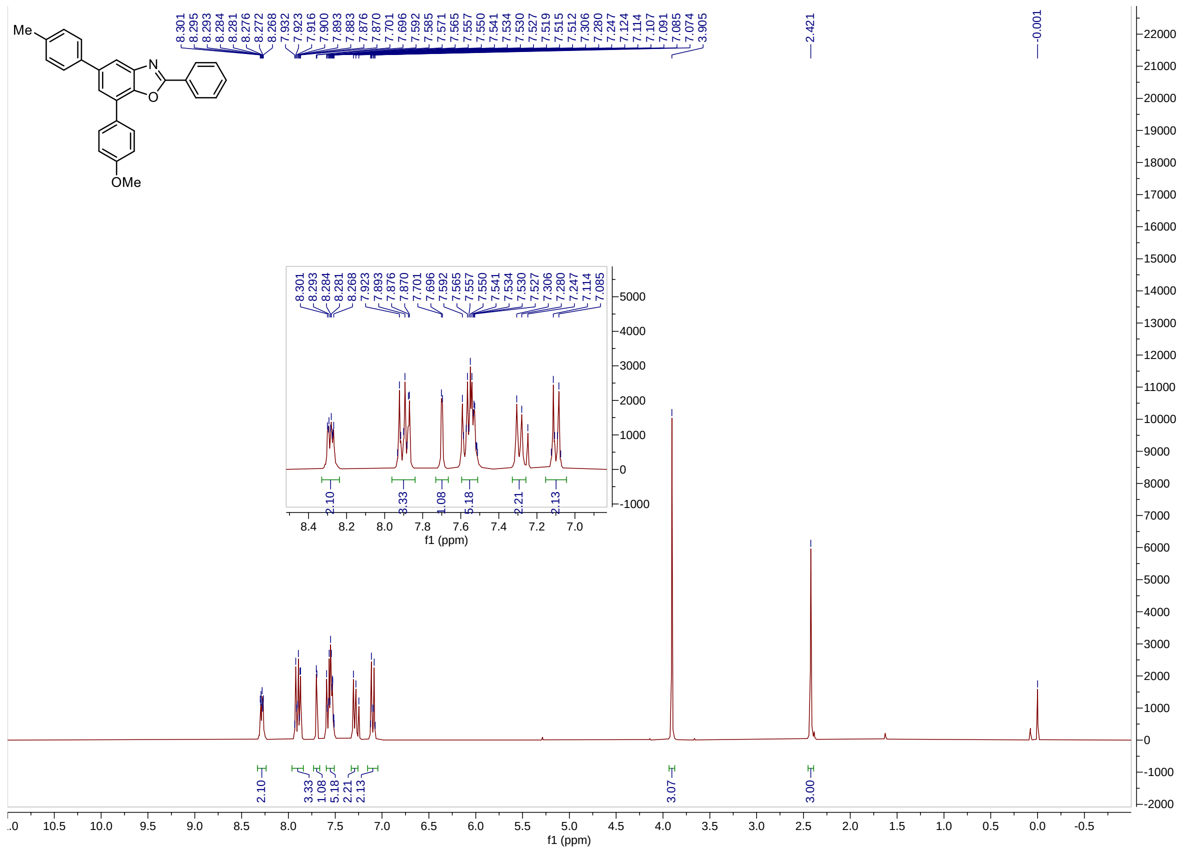

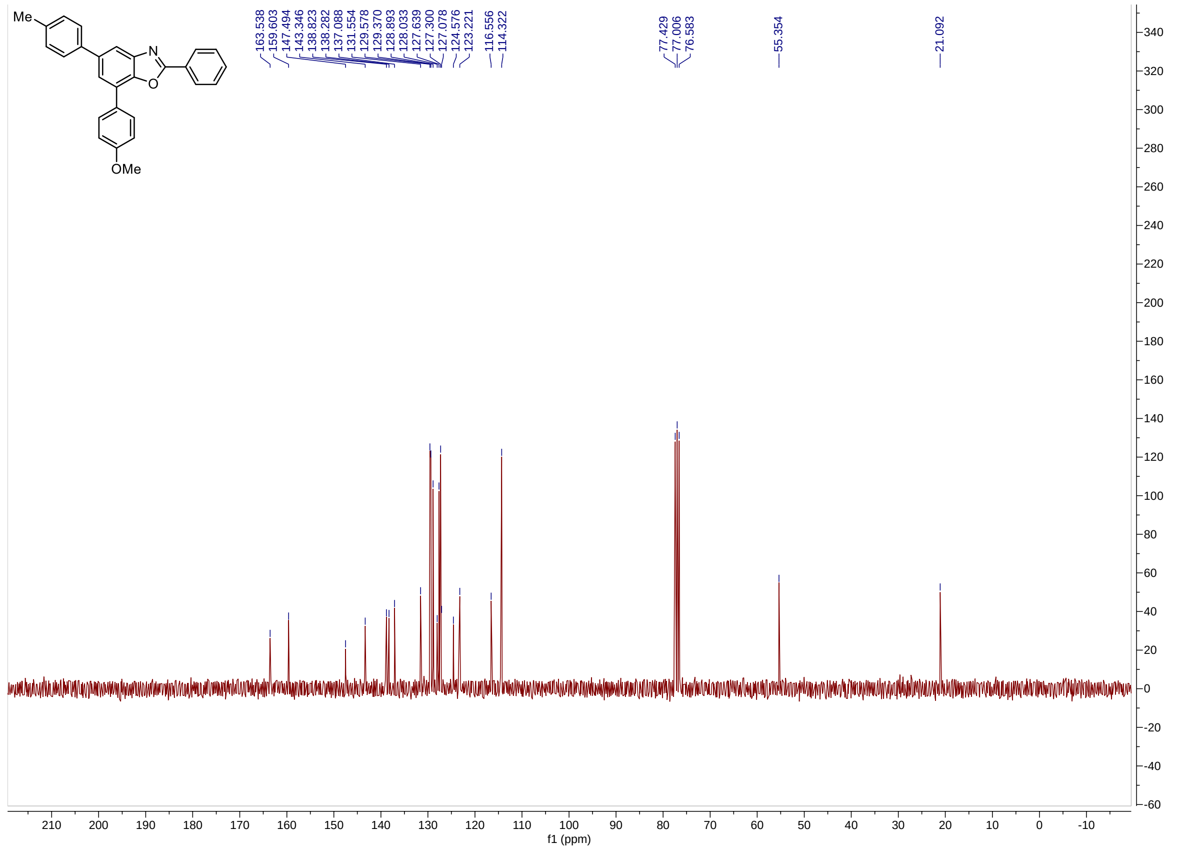

5m, ^{1}H NMR-300 MHz in $CDCl_3$ and ^{13}C NMR-75 MHz in $CDCl_3$

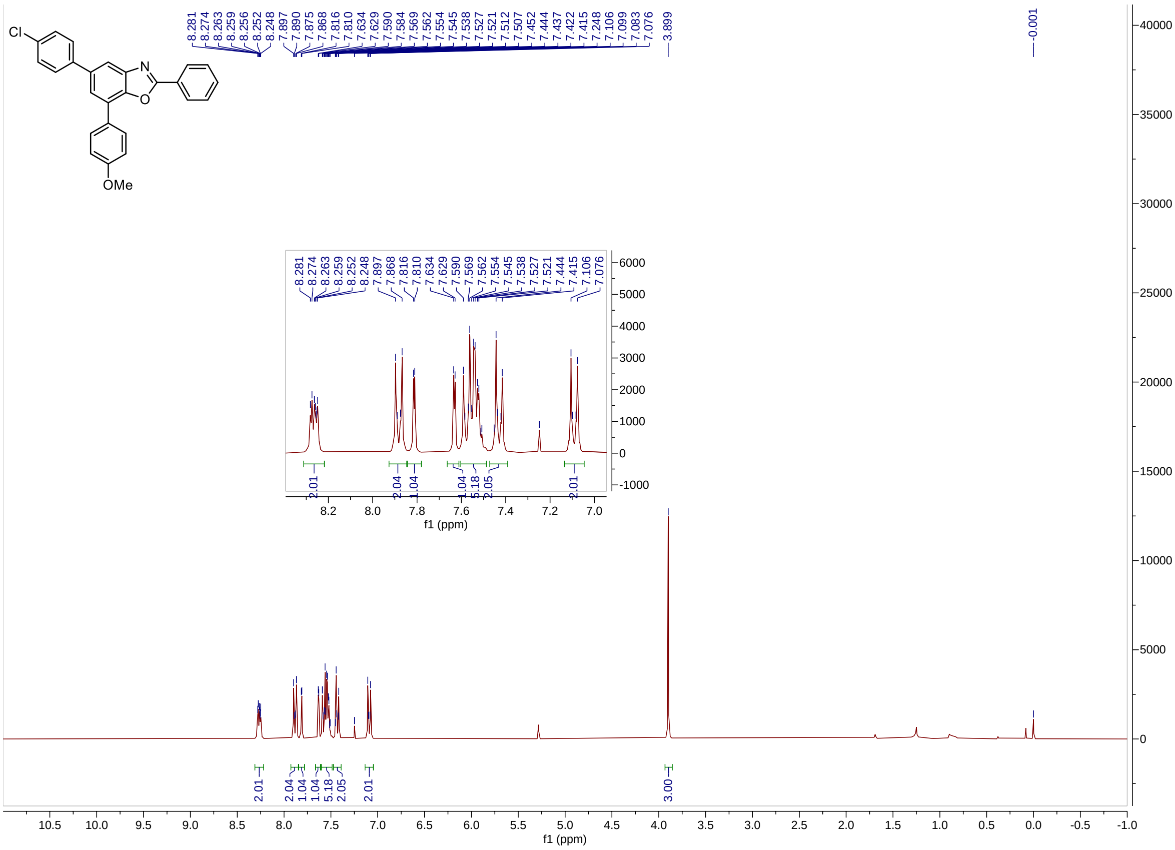

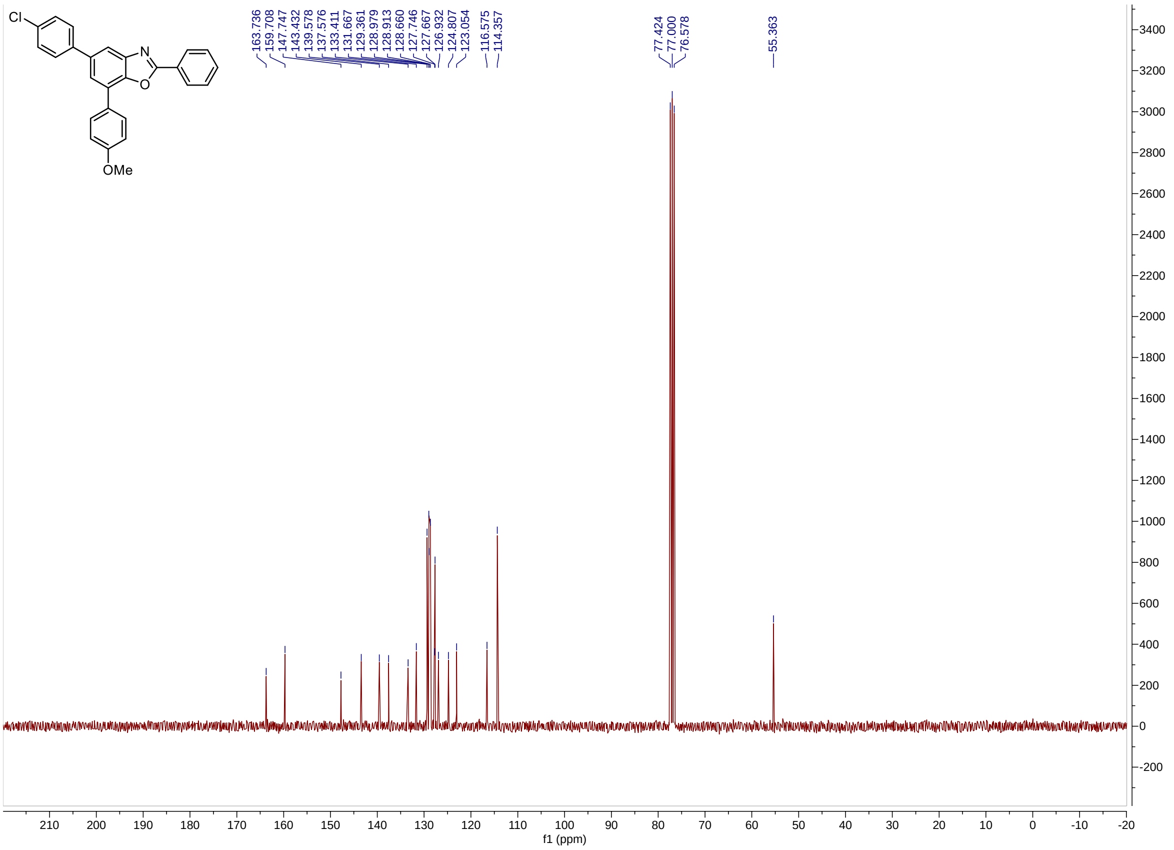

12, ^{1}H NMR-300 MHz in $CDCl_3$ and ^{13}C NMR-75 MHz in $CDCl_3$

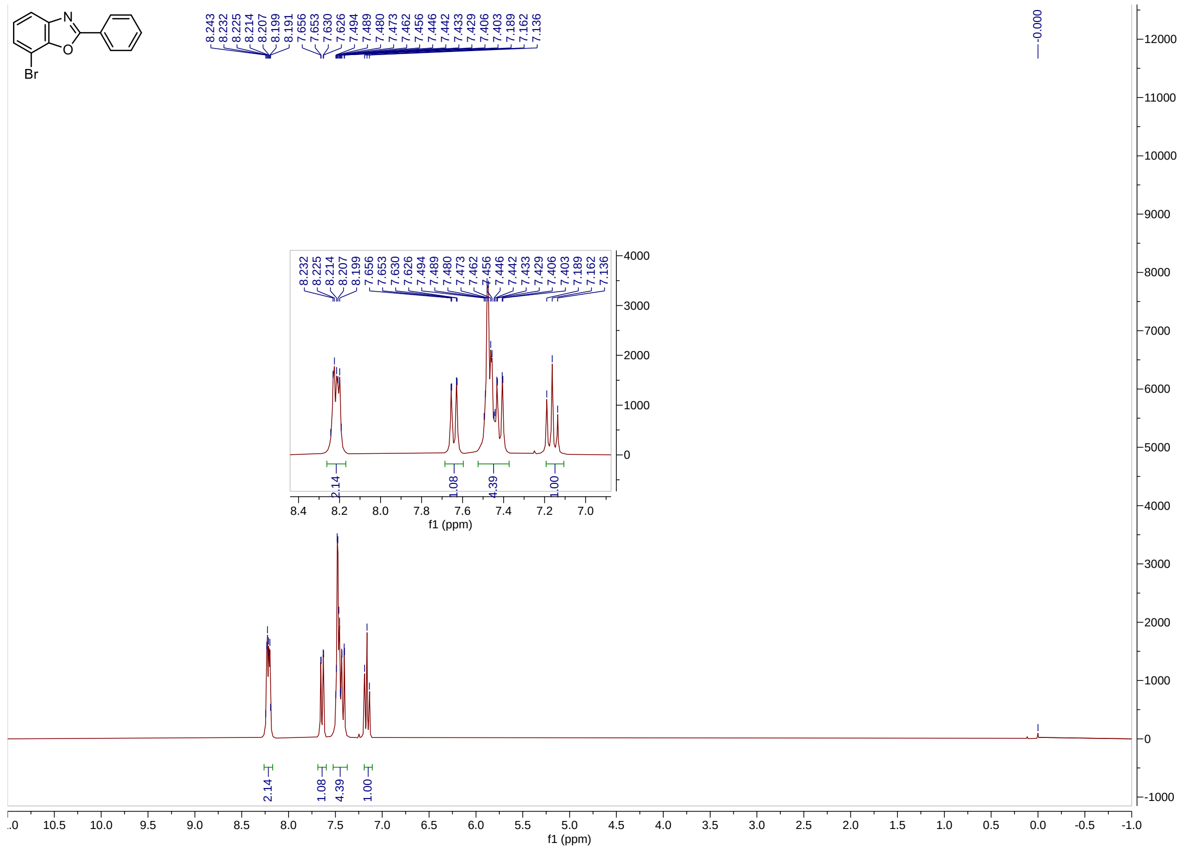

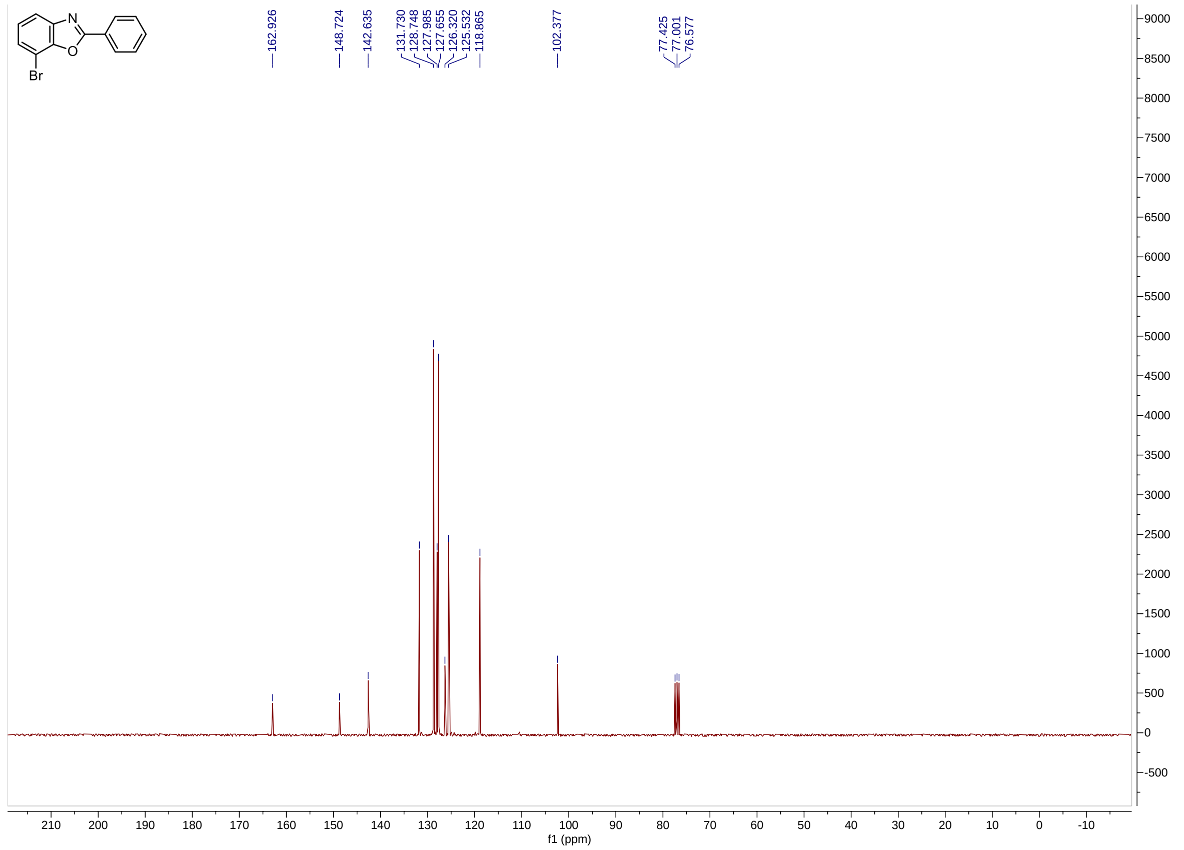

13, ^{1}H NMR-300 MHz in $CDCl_3$ and ^{13}C NMR-75 MHz in $CDCl_3$

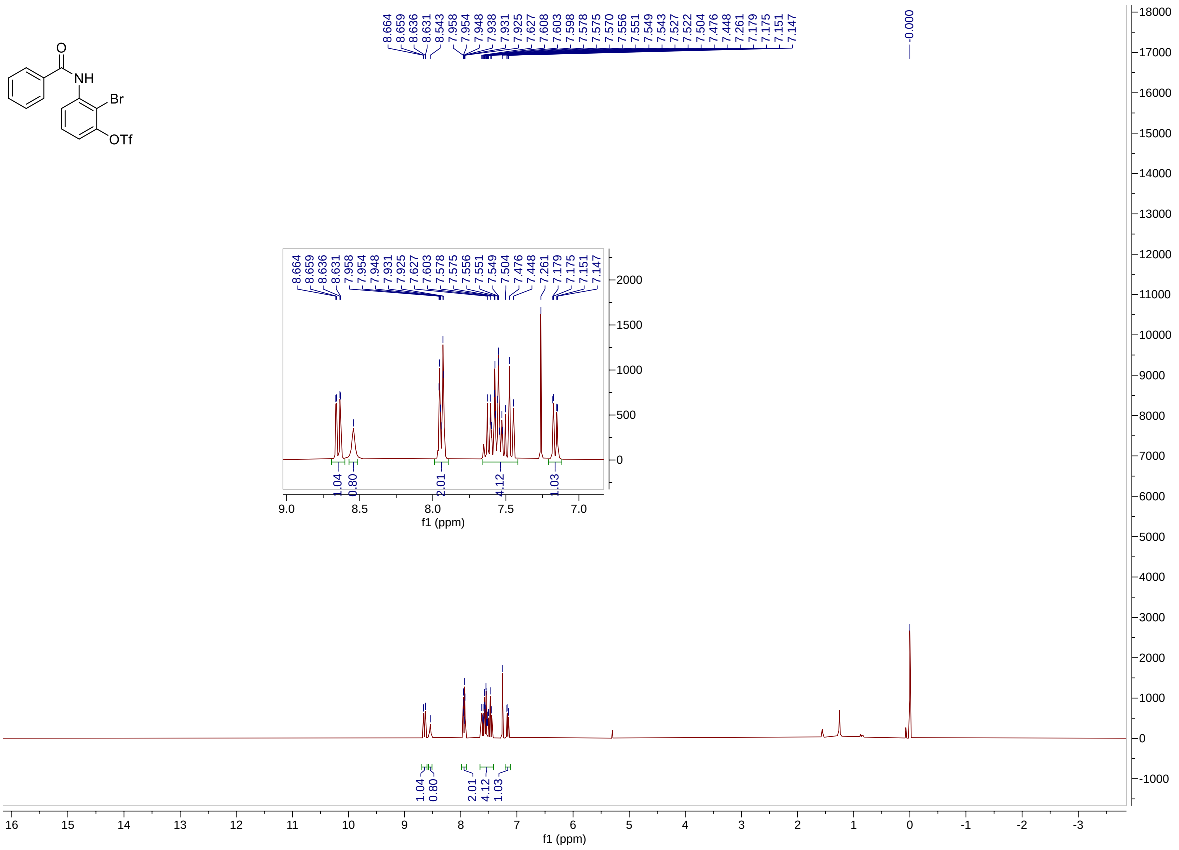

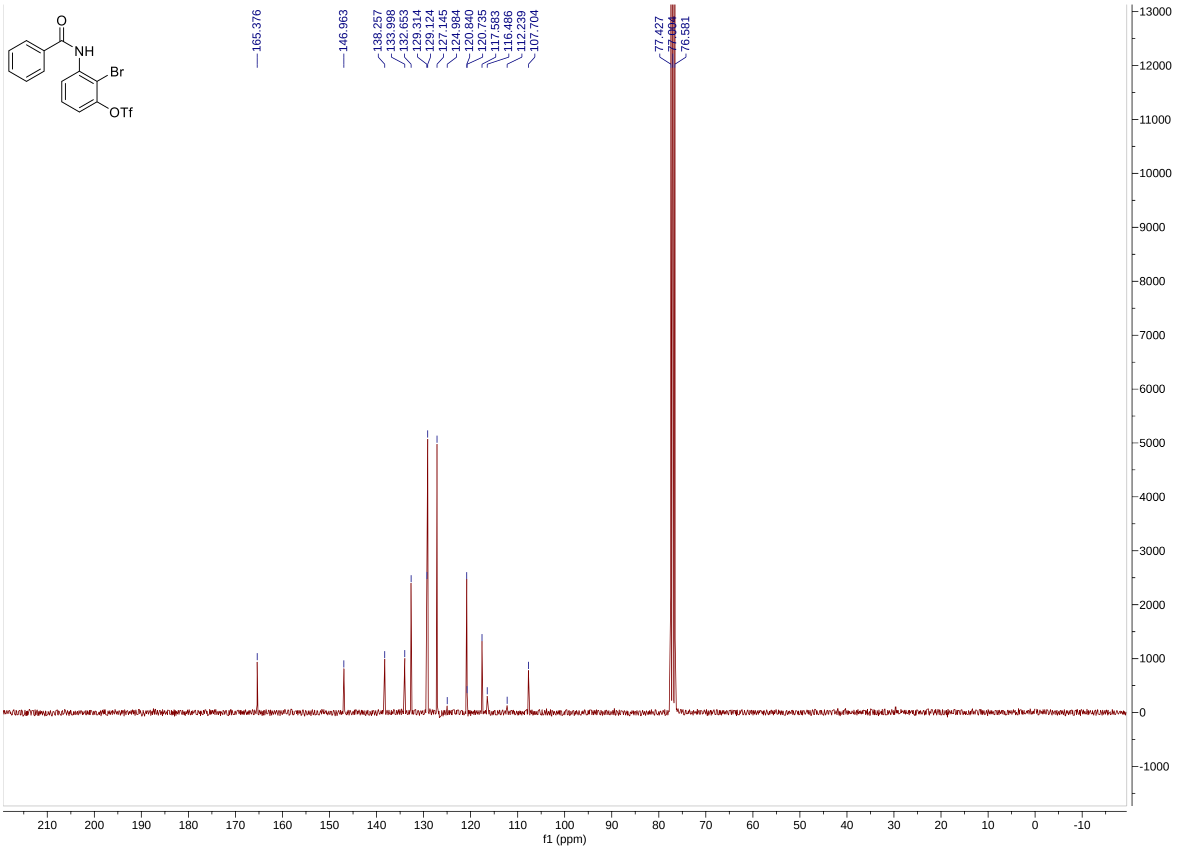

15b, ^{1}H NMR-300 MHz in $CDCl_3$, ^{13}C NMR-75 MHz in $CDCl_3$, and ^{11}B NMR-96 MHz in $CDCl_3$

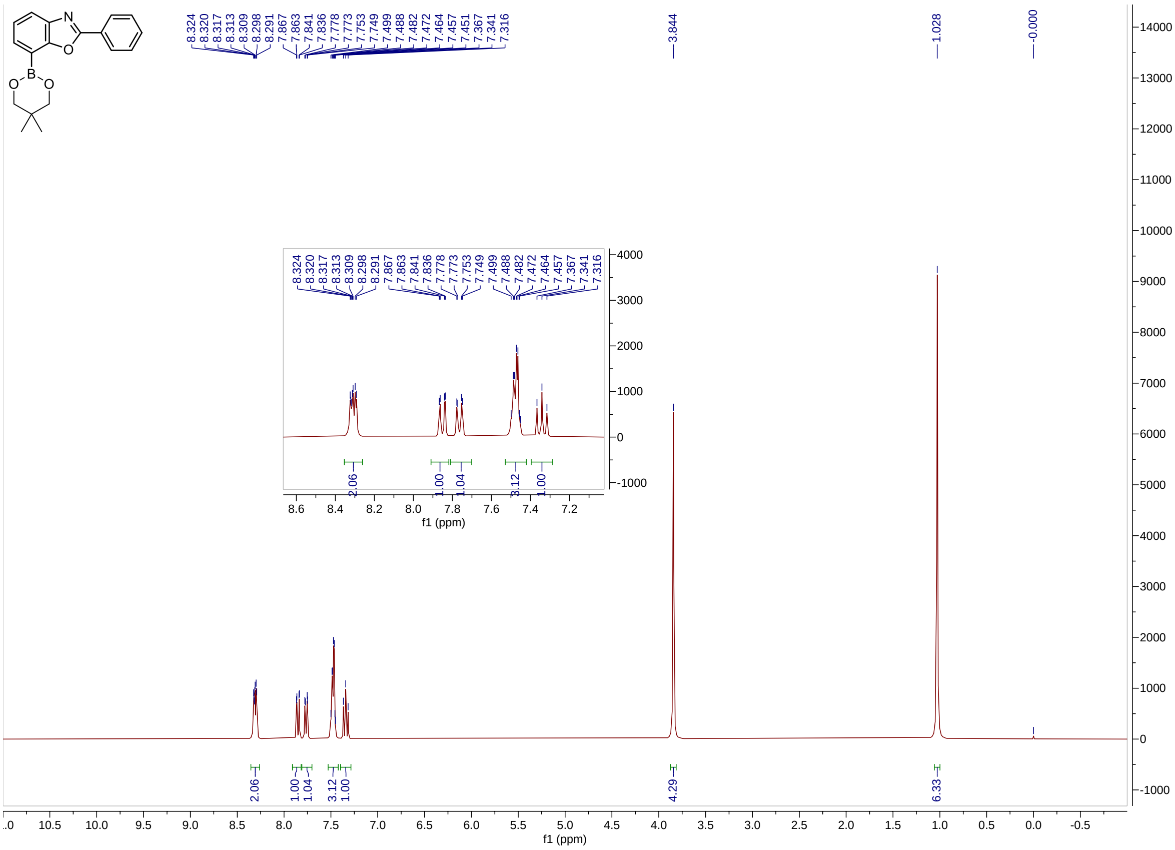

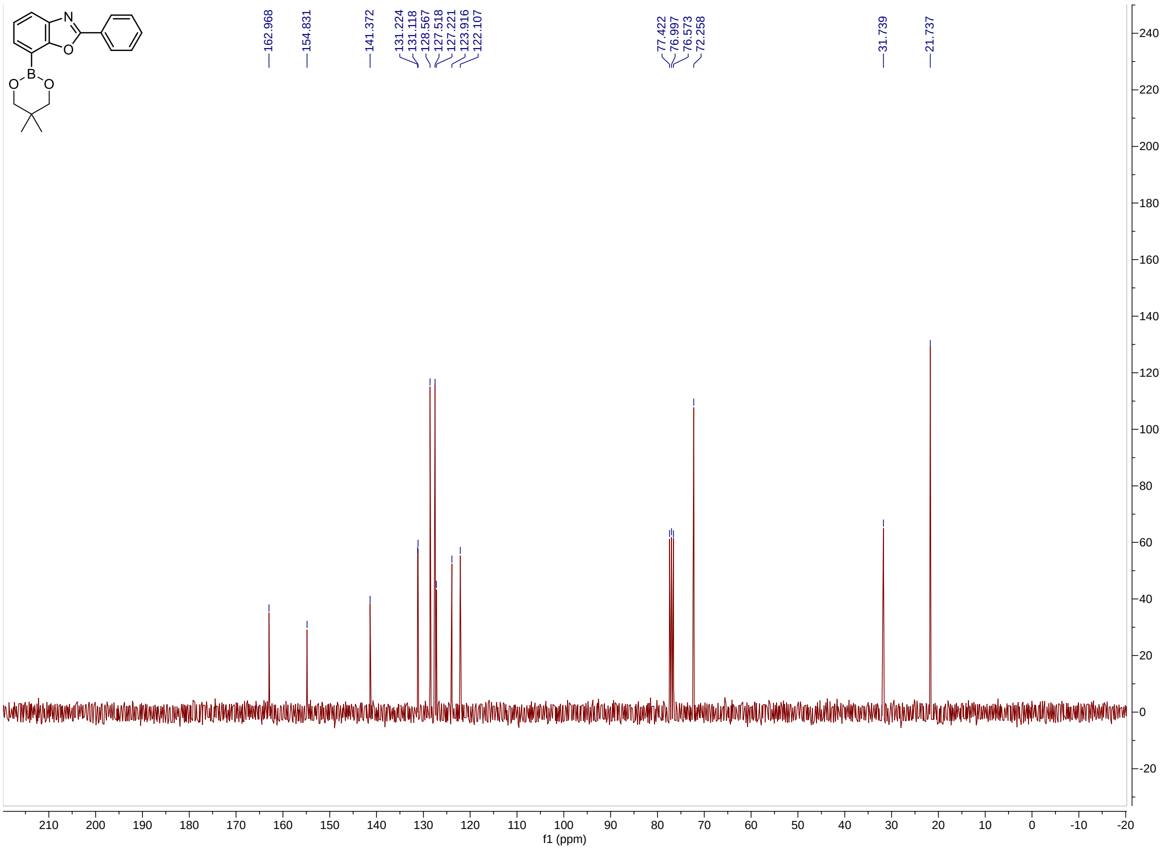

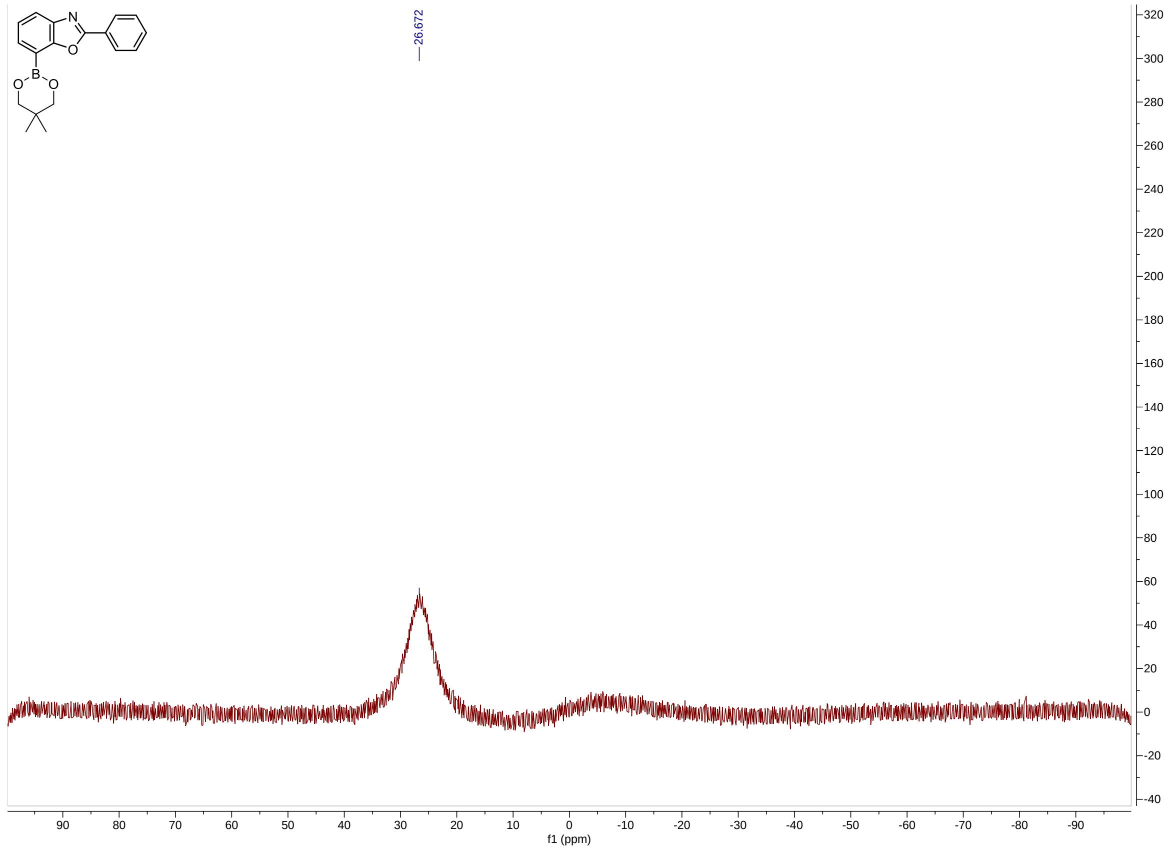

N
O
B
O
O
26.672
320
300
280
260
240
220
200
180
160
140
120
100
80
60
40
20
0
-20
-40
90
80
70
60
50
40
30
20
10
0
-10
-20
-30
-40
-50
-60
-70
-80
-90
f1 (ppm)

15, ^{1}H NMR-300 MHz in pyridine-d_6

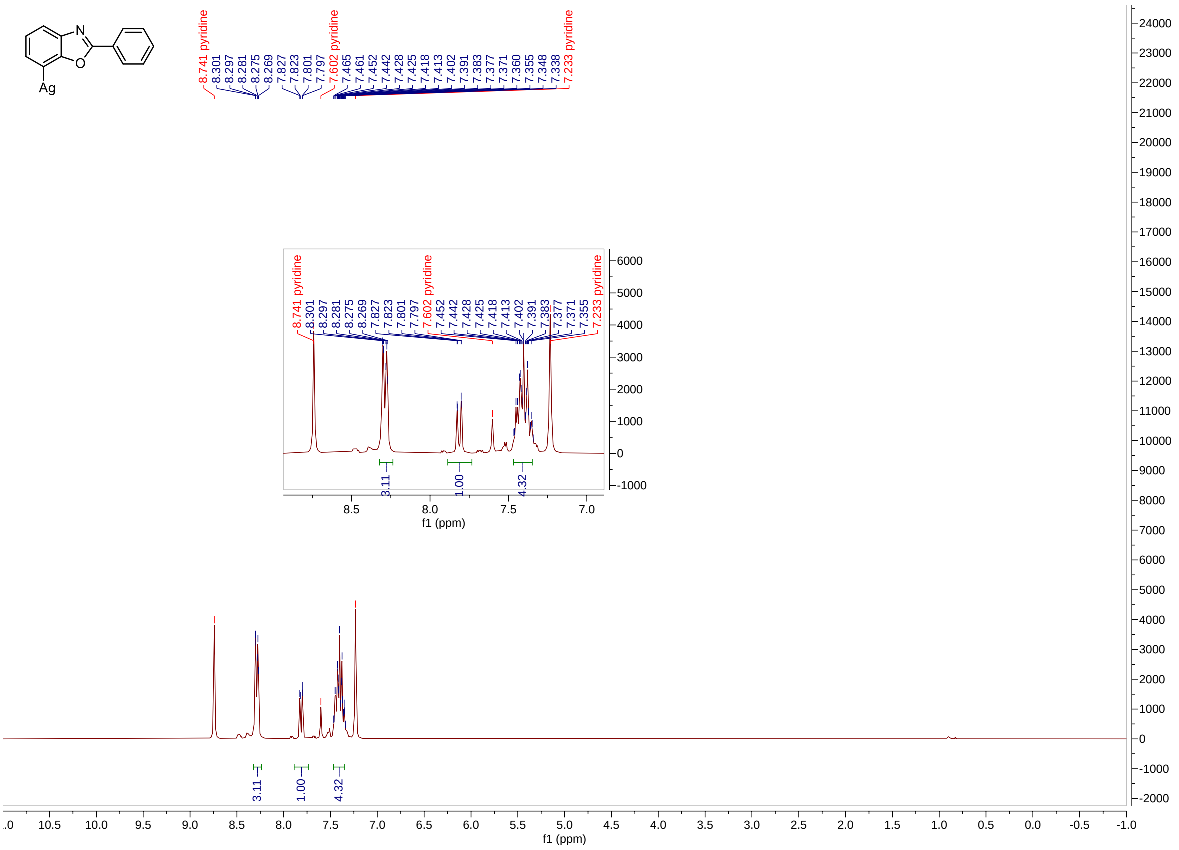

Appendix 2: Cartesian Coordinates for Optimized Structures

A

C	3.21786	0.61164	3.91029
C	2.22232	-0.06106	3.18511
C	1.58887	-1.17153	3.72309
C	1.96005	-1.64457	4.97925
C	3.58200	0.21672	5.22638
C	2.92735	-0.94152	5.68434
N	3.89761	1.67398	3.25514
C	3.29968	2.63531	2.47405
C	4.22555	3.60445	1.69970
C	3.75512	3.58512	0.22925
C	4.00667	5.01424	2.29045
C	5.71922	3.24237	1.75200
O	2.07754	2.73743	2.38462
H	1.96841	0.28401	2.19159
H	0.83180	-1.69850	3.15138
H	1.51119	-2.54317	5.37851
H	2.69301	3.83018	0.15748
H	3.91011	2.59915	-0.22350
H	4.32371	4.31756	-0.35332
H	2.94838	5.28804	2.25731
H	4.57327	5.75358	1.71429
H	4.34228	5.06723	3.33185
H	5.90866	2.23366	1.36887
H	6.13326	3.31594	2.76337
H	6.28320	3.94228	1.12685
Si	4.81895	1.19806	6.35060
C	4.86545	3.02265	5.83668
C	6.58885	0.43508	6.23304
C	4.19834	1.23837	8.13715
O	3.38749	-1.47031	6.93424
S	2.39614	-1.91732	8.15052
O	3.11679	-1.65804	9.38695
O	1.03172	-1.45756	7.93466
C	2.43794	-3.78929	7.96427
F	1.67597	-4.18440	6.94664
F	1.96716	-4.30545	9.09662
F	3.69133	-4.18402	7.77071
C	7.08233	0.44122	4.77141
C	6.62091	-1.01408	6.75894
C	7.54604	1.29480	7.08864
H	3.12017	1.43454	8.17528
H	4.69189	2.06736	8.65759
H	4.39475	0.32510	8.69934
H	5.47559	3.23169	4.95569
H	5.28577	3.59713	6.67060
H	3.85697	3.40861	5.65332
H	4.90173	1.70613	3.35344
H	5.99038	-1.68291	6.16375
H	6.29054	-1.08392	7.80081
H	7.64693	-1.40611	6.71372
H	7.59375	2.33267	6.74024
H	8.56575	0.88770	7.03871
H	7.25396	1.30872	8.14506
H	6.44502	-0.16972	4.12101
H	8.09873	0.02750	4.71006
H	7.12237	1.45352	4.35259

B

C	2.93313	0.72990	3.84509
C	1.92026	-0.00504	3.19858
C	1.40574	-1.15881	3.77387
C	1.88061	-1.61309	4.99913
C	3.41630	0.34164	5.13317
C	2.84568	-0.83968	5.63079
N	3.48836	1.86537	3.21161
C	3.28060	2.33303	1.92767
C	4.12592	3.56481	1.51380
C	3.73542	3.95380	0.07832
C	3.83721	4.75905	2.44963
C	5.62785	3.20292	1.54190
O	2.48899	1.81759	1.14085
H	1.56542	0.33162	2.23756
H	0.63728	-1.72187	3.25322
H	1.50744	-2.53069	5.43106
H	2.67600	4.21641	0.00962
H	3.92320	3.13659	-0.62324
H	4.32557	4.82072	-0.23639
H	2.76860	5.00038	2.46085
H	4.37696	5.64204	2.09059
H	4.15581	4.57719	3.47864
H	5.83972	2.35567	0.88051
H	5.98164	2.94542	2.54399
H	6.21795	4.05764	1.19396
Si	4.73570	1.26022	6.24675
C	5.08608	3.02817	5.66675
C	6.39398	0.22697	6.31409
C	3.96221	1.59067	7.94133
O	3.42717	-1.31323	6.85714
S	2.59937	-1.96767	8.09257
O	3.28634	-1.57609	9.31382
O	1.15936	-1.81697	7.93903
C	3.04908	-3.77162	7.83503
F	2.45472	-4.25249	6.74544
F	2.63558	-4.44576	8.90536
F	4.36857	-3.87884	7.71210
C	6.36503	-1.02783	5.41993
C	6.67430	-0.17728	7.77879
C	7.56555	1.13368	5.87081
H	3.01256	2.13008	7.83484
H	4.69957	2.24605	8.42485
H	3.79325	0.71486	8.56794
H	5.66826	3.15657	4.74976
H	5.70103	3.38246	6.51479
H	4.17495	3.63495	5.60680
H	4.16655	2.37136	3.76437
H	5.88706	-0.81017	8.20213
H	6.77601	0.73102	8.38257
H	7.61653	-0.74359	7.83894
H	7.46410	1.47851	4.83444
H	8.51100	0.57295	5.93206
H	7.61920	1.99663	6.54488
H	5.62038	-1.76004	5.74740
H	7.34397	-1.52842	5.44975
H	6.15863	-0.78725	4.36995
F	6.70906	3.00683	8.18541

B2

C	-0.81756	1.42303	-1.19475
C	-2.19003	1.34097	-1.56344
C	-3.00442	0.29253	-1.17491
C	-2.47602	-0.74665	-0.40047
C	-0.32397	0.51061	-0.19226
C	-1.17734	-0.58260	0.05551
N	-0.10808	2.38078	-1.89938
C	1.10861	2.06698	-2.38748
C	1.62329	3.05941	-3.48139
C	1.60480	4.50914	-2.95447
C	3.05732	2.68779	-3.89638
C	0.70798	2.96112	-4.71911
O	1.80657	1.05757	-2.11727
H	-2.56161	2.10317	-2.24173
H	-4.03360	0.23493	-1.51846
H	-3.06327	-1.62667	-0.17788
H	2.26992	4.63504	-2.09282
H	0.59704	4.80112	-2.64636
H	1.93593	5.20927	-3.73308
H	3.74550	2.71971	-3.04734
H	3.42270	3.38235	-4.66270
H	3.09937	1.67542	-4.31109
H	-0.31871	3.24671	-4.47377
H	0.68558	1.93824	-5.11542
H	1.06698	3.61899	-5.52039
Si	1.16998	0.74800	1.01142
C	0.44243	0.40014	2.73849
C	1.92342	2.55106	1.19112
C	2.56927	-0.48164	0.68165
O	-0.54315	-1.67167	0.77450
S	-1.23351	-2.74137	1.74626
O	-0.20854	-3.15505	2.69456
O	-2.56422	-2.34727	2.19313
C	-1.42469	-4.18257	0.56377
F	-2.32462	-3.89703	-0.37520
F	-1.83198	-5.23841	1.26782
F	-0.24940	-4.44541	-0.00096
C	0.82915	3.63656	1.08447
C	3.06743	2.84393	0.20279
C	2.54539	2.67412	2.60507
H	2.23172	-1.51845	0.78102
H	3.39592	-0.33137	1.38670
H	2.94709	-0.33855	-0.33357
H	-0.41023	1.05503	2.95276
H	1.19166	0.56847	3.51620
H	0.11913	-0.63420	2.84884
H	2.78367	2.67945	-0.83329
H	3.93398	2.20130	0.40277
H	3.40823	3.88394	0.31973
H	1.79150	2.69287	3.39670
H	3.10289	3.61933	2.66797
H	3.25462	1.86891	2.83306
H	0.39296	3.68264	0.08451
H	1.25169	4.62620	1.31671
H	0.01502	3.46326	1.79912

C

C	-0.89104	0.75416	-1.61688
C	-1.99493	0.13122	-2.22802
C	-2.59513	-0.96730	-1.62155
C	-2.07006	-1.47900	-0.43811
C	-0.39143	0.37363	-0.33838
C	-1.00299	-0.78911	0.13143
N	-0.17022	1.74456	-2.34547
C	-0.57570	2.49980	-3.41261
C	0.48557	3.42607	-4.06323
C	0.29854	3.33363	-5.59094
C	0.17772	4.86605	-3.59421
C	1.94101	3.06524	-3.71807
O	-1.72873	2.48679	-3.85101
H	-2.35733	0.50091	-3.17675
H	-3.44191	-1.45592	-2.09444
H	-2.48171	-2.37643	0.00294
H	-0.73138	3.56283	-5.87215
H	0.53570	2.32871	-5.95859
H	0.96592	4.04264	-6.09216
H	-0.85012	5.14518	-3.84553
H	0.85534	5.57427	-4.08368
H	0.30526	4.96904	-2.51122
H	2.17324	2.02575	-3.97369
H	2.17451	3.22495	-2.66039
H	2.61927	3.70691	-4.29036
Si	0.99249	1.47757	0.97325
C	0.67832	3.19193	0.16261
C	2.56353	0.36556	0.44326
C	-0.23342	1.28985	2.44824
O	-0.33144	-1.39912	1.27974
S	-1.04824	-2.07950	2.54653
O	-0.18861	-1.87024	3.70433
O	-2.48097	-1.81173	2.60251
C	-0.81759	-3.89098	2.12392
F	-1.54666	-4.23505	1.06228
F	-1.20903	-4.60764	3.17820
F	0.46860	-4.12690	1.87427
C	2.35913	-0.64770	-0.69610
C	3.05425	-0.41686	1.68192
C	3.69535	1.32080	-0.00305
H	-1.23471	0.92602	2.20349
H	-0.34711	2.29077	2.88356
H	0.17605	0.65550	3.24001
H	1.47002	3.48927	-0.53553
H	0.69618	3.92721	0.97483
H	-0.27859	3.28911	-0.35501
H	0.76510	1.90696	-2.00436
H	2.30380	-1.13586	2.03034
H	3.28067	0.25847	2.51053
H	3.96774	-0.98713	1.45025
H	3.42990	1.86857	-0.91657
H	4.61497	0.75746	-0.22554
H	3.92602	2.05575	0.77050
H	1.64351	-1.43209	-0.43588
H	3.31330	-1.14522	-0.93112
H	2.00935	-0.17516	-1.62092
F	2.09557	2.25859	2.13327

D

C	-1.20977	0.33508	-2.05288
C	-2.46578	0.05649	-2.62257
C	-3.21316	-1.01572	-2.12150
C	-2.71707	-1.78960	-1.07202
C	-0.62691	-0.41387	-1.00084
C	-1.45471	-1.43214	-0.59286
N	-0.40240	1.41955	-2.51442
C	-0.61870	2.34308	-3.48974
C	0.49895	3.39419	-3.72181
C	0.93669	3.26670	-5.19630
C	-0.13481	4.78252	-3.49109
C	1.72769	3.23740	-2.81056
O	-1.64282	2.38161	-4.18279
H	-2.84497	0.66607	-3.43238
H	-4.18700	-1.24169	-2.54800
H	-3.28015	-2.62037	-0.65661
H	0.07676	3.36375	-5.86410
H	1.40872	2.29586	-5.38655
H	1.66180	4.04918	-5.44553
H	-1.01619	4.91487	-4.12402
H	0.58605	5.57264	-3.72830
H	-0.44243	4.90860	-2.44659
H	2.22567	2.27159	-2.95274
H	1.47160	3.34505	-1.75041
H	2.45955	4.01703	-3.04705
O	-1.00057	-2.34574	0.47534
S	-0.01116	-1.78961	1.60787
O	1.40378	-1.93439	1.26841
O	-0.50162	-0.57551	2.25912
C	-0.37822	-3.18774	2.78976
F	-1.66404	-3.17870	3.14403
F	0.37733	-3.01189	3.87927
F	-0.07918	-4.36397	2.23555
H	0.46576	1.46837	-1.99761

E

C	-0.79979	0.46401	-1.92364
C	-2.12120	0.04649	-2.23500
C	-2.72521	-1.04775	-1.59398
C	-2.08224	-1.82031	-0.59664
C	-0.20529	-0.32897	-0.93325
C	-0.82634	-1.28629	-0.41989
N	-0.11984	1.53998	-2.50903
C	-0.56285	2.40464	-3.48183
C	0.42276	3.50492	-3.93940
C	0.67462	3.27866	-5.44616
C	-0.28696	4.85962	-3.73050
C	1.76585	3.51010	-3.19039
O	-1.68369	2.32105	-3.98166
H	-2.66792	0.59579	-2.98989
H	-3.74036	-1.31852	-1.87628
H	-2.54067	-2.66453	-0.09818
H	-0.26721	3.27176	-6.00108
H	1.18492	2.32542	-5.62423
H	1.30611	4.08029	-5.84336
H	-1.24828	4.87899	-4.25009
H	0.33615	5.67116	-4.12057
H	-0.46785	5.05233	-2.66703
H	2.32442	2.57852	-3.33675
H	1.63806	3.68019	-2.11514
H	2.39062	4.32281	-3.57419
H	0.81569	1.67755	-2.15219

F

C	-0.74969	0.52778	-1.94690
C	-2.09187	0.06928	-2.24426
C	-2.71379	-1.02087	-1.61609
C	-2.09976	-1.80551	-0.61211
C	-0.27466	-0.34372	-0.93888
C	-0.81453	-1.31796	-0.37001
N	-0.02970	1.55968	-2.47239
C	-0.53614	2.35891	-3.43440
C	0.41942	3.48839	-3.92832
C	0.65645	3.26944	-5.43770
C	-0.30112	4.83718	-3.72265
C	1.77142	3.51892	-3.20096
O	-1.66563	2.30375	-3.97762
H	-2.61706	0.63132	-3.00760
H	-3.72865	-1.27464	-1.92209
H	-2.58866	-2.64637	-0.13240
H	-0.29635	3.22625	-5.97283
H	1.18970	2.32856	-5.62188
H	1.25777	4.08231	-5.86283
H	-1.27779	4.82826	-4.21422
H	0.28938	5.66440	-4.13499
H	-0.46213	5.04134	-2.65681
H	2.32103	2.58202	-3.33183
H	1.64677	3.67752	-2.12529
H	2.39340	4.33413	-3.59313

G

C	0.18118	-1.26771	0.37260
C	0.25405	-2.54448	0.94303
C	-0.93813	-3.04040	1.47757
C	-2.13495	-2.28515	1.43831
C	-1.05237	-0.59451	0.37873
C	-2.29069	-0.98596	0.87814
N	1.16467	-0.48136	-0.24385
C	0.53453	0.60738	-0.58567
C	1.09968	1.81775	-1.29295
C	0.35052	2.01289	-2.63113
C	0.89394	3.06404	-0.40200
C	2.59898	1.61209	-1.56109
O	-0.78301	0.64468	-0.26489
H	1.18239	-3.10924	0.96463
H	-0.94127	-4.03002	1.93459
H	-3.00692	-2.77453	1.88840
H	-0.72153	2.15805	-2.46887
H	0.48198	1.14512	-3.28663
H	0.73979	2.89436	-3.15198
H	-0.16647	3.23351	-0.19385
H	1.28943	3.95252	-0.90594

H	1.41622	2.95537	0.55478
H	2.77562	0.73617	-2.19254
H	3.15576	1.47245	-0.62949
H	3.00465	2.49065	-2.07362

D_Ag

C	2.78359	0.43806	3.81848
C	1.84109	-0.34454	3.12592
C	1.47033	-1.59407	3.62459
C	2.02194	-2.07959	4.80769
C	3.36935	-0.01685	5.02497
C	2.94819	-1.26601	5.45766
C	2.78714	2.40712	2.24218
C	3.44900	3.78902	2.01161
C	4.05737	3.76532	0.59313
C	2.31912	4.84005	2.07261
C	4.54385	4.15655	3.02776
O	1.93286	1.99640	1.45378
H	1.41430	0.03380	2.20798
H	0.74526	-2.19312	3.08151
H	1.75165	-3.04958	5.20967
H	3.30074	3.49663	-0.14838
H	4.87599	3.03957	0.52518
H	4.45869	4.75253	0.34023
H	1.52743	4.60020	1.35773
H	2.71566	5.83244	1.83240
H	1.87447	4.88622	3.07341
H	5.37228	3.43867	3.01810
H	4.15274	4.22982	4.04927
H	4.96076	5.13633	2.77275
O	3.57274	-1.82428	6.63130
S	2.92847	-1.55855	8.09844
O	4.00431	-1.16542	8.99950
O	1.68246	-0.80763	8.01733
C	2.50291	-3.32550	8.53291
F	1.57856	-3.78768	7.69240
F	2.02487	-3.34155	9.77613
F	3.59673	-4.07797	8.45958
N	3.19660	1.70517	3.34456
H	3.89886	2.13968	3.92962
Ag	4.81999	1.06346	6.17002

D2_Ag

C	3.18313	0.54147	4.00851
C	2.09013	0.02321	3.28388
C	1.46984	-1.15921	3.67490
C	1.92914	-1.84161	4.80139
C	3.68940	-0.12177	5.14808
C	3.01382	-1.29156	5.47734
C	4.48942	2.74295	3.96459
C	4.85576	3.89695	2.99494
C	4.32493	5.20271	3.62491
C	6.39812	3.94435	2.92290
C	4.28683	3.75226	1.57269
O	4.89205	2.79914	5.13523
H	1.72956	0.55363	2.40554
H	0.62982	-1.54618	3.10645
H	1.46867	-2.76414	5.13732
H	4.71766	5.33610	4.63605
H	3.23031	5.19914	3.67951
H	4.63061	6.06099	3.01723
H	6.83153	4.05946	3.91983
H	6.71734	4.79114	2.30606
H	6.80192	3.02883	2.47533
H	3.19054	3.73658	1.56460
H	4.65725	2.85290	1.06713
H	4.59956	4.61148	0.97078
O	3.52692	-2.08677	6.56969
S	3.07713	-1.76250	8.09209
O	4.27027	-1.74697	8.92916
O	2.08161	-0.70004	8.15601
C	2.20354	-3.37223	8.45781
F	1.14703	-3.50372	7.65601
F	1.80009	-3.34904	9.72753
F	3.04081	-4.38858	8.26929
N	3.74001	1.72913	3.44692
H	3.43960	1.88004	2.49359
Ag	5.31456	0.63584	6.33484

E_Ag

C	2.70954	0.84336	3.60012
C	1.73082	0.14553	2.85158
C	1.03131	-0.94734	3.38054
C	1.23515	-1.44701	4.69249
C	2.88598	0.32579	4.88637
C	2.20096	-0.67097	5.28247
C	4.35947	2.68679	3.66804
C	4.96006	3.85498	2.86185
C	4.60637	5.15171	3.62528
C	6.49182	3.65881	2.84598
C	4.43948	3.95025	1.41830
O	4.75199	2.48390	4.82716
H	1.51896	0.46887	1.83584
H	0.28975	-1.44362	2.75902
H	0.69747	-2.28990	5.10570
H	4.98034	5.11783	4.65194
H	3.52272	5.30998	3.65678
H	5.06054	6.00971	3.11929
H	6.89003	3.59341	3.86158
H	6.96450	4.50778	2.34191
H	6.76867	2.74612	2.30675
H	3.36010	4.13670	1.37776
H	4.66719	3.04931	0.83716
H	4.92900	4.79120	0.91794
N	3.39962	1.92643	3.05968
H	3.15420	2.16470	2.10647
O	5.55988	1.08265	8.22318
Ag	4.18409	0.79617	6.49222
C	6.05505	-1.28720	9.21831
O	3.81600	0.03582	9.67355
O	5.95026	0.92182	10.67516
S	5.24649	0.35821	9.51386
F	5.53264	-1.87108	8.12463
F	5.85940	-2.10024	10.26536

F	7.37353	-1.14274	9.03036

F_Ag

C	-0.75934	0.40557	-1.94095
C	-2.12409	0.09001	-2.24764
C	-2.82721	-0.97929	-1.68199
C	-2.26686	-1.87575	-0.73784
C	-0.28892	-0.54017	-0.99036
C	-0.95541	-1.50519	-0.49779
N	-0.14577	1.46883	-2.52759
C	1.12468	1.79235	-2.29670
C	1.67173	3.03230	-3.04682
C	2.22501	4.02451	-2.00090
C	2.82308	2.56118	-3.96307
C	0.59905	3.73115	-3.89442
O	1.97778	1.21356	-1.54078
H	-2.62436	0.73326	-2.96665
H	-3.86110	-1.13611	-1.98413
H	-2.81220	-2.70507	-0.30518
H	2.99599	3.55444	-1.38458
H	1.42955	4.38278	-1.33644
H	2.66405	4.89710	-2.49801
H	3.60405	2.05993	-3.38434
H	3.27268	3.41594	-4.48139
H	2.46020	1.86034	-4.72437
H	-0.23308	4.08678	-3.27915
H	0.18659	3.06337	-4.65612
H	1.03868	4.59753	-4.40324
Ag	1.66948	-0.51618	-0.12886

G_Ag

C	3.16612	1.06756	2.98301
C	2.05725	0.25511	3.23807
C	2.31844	-1.02738	3.72230
C	3.63523	-1.48551	3.94434
C	4.45727	0.57144	3.21855
C	4.77466	-0.69331	3.69720
N	3.25947	2.37891	2.50363
C	4.53518	2.62546	2.46558
C	5.23433	3.89058	2.03383
C	6.19191	3.56501	0.86434
C	4.19047	4.92480	1.58194
C	6.04436	4.44885	3.22716
O	5.33360	1.59013	2.87959
H	1.04527	0.60949	3.06655
H	1.48735	-1.69518	3.93561
H	3.75739	-2.49732	4.32280
H	5.64404	3.16198	0.00585
H	6.95132	2.83514	1.16008
H	6.70426	4.47806	0.54350
H	3.60164	4.55495	0.73709
H	4.69904	5.84257	1.26964
H	3.49921	5.17512	2.39226
H	6.79760	3.73290	3.56940
H	5.38897	4.68762	4.07157
H	6.55936	5.36682	2.92510

Ag	6.77611	-1.31109	4.00731

TS1

C	2.93672	0.77685	3.93530
C	1.88423	0.07378	3.31713
C	1.35370	-1.06551	3.90754
C	1.86524	-1.53760	5.11059
C	3.44113	0.39344	5.21678
C	2.86917	-0.78709	5.71144
N	3.53271	1.87097	3.26158
C	3.28041	2.36320	1.99769
C	4.22055	3.50070	1.52099
C	3.89851	3.80509	0.04868
C	3.96482	4.77058	2.36336
C	5.69874	3.06890	1.63043
O	2.37396	1.94957	1.27595
H	1.51265	0.42204	2.36627
H	0.55291	-1.60634	3.41250
H	1.49055	-2.45151	5.54895
H	2.85822	4.11610	-0.07662
H	4.06430	2.92987	-0.58644
H	4.54591	4.61382	-0.30601
H	2.91471	5.07696	2.30220
H	4.57821	5.59418	1.98164
H	4.21739	4.62923	3.41709
H	5.88533	2.15150	1.06148
H	6.01483	2.89638	2.66320
H	6.34187	3.85538	1.22102
Si	4.75726	1.35301	6.37901
C	4.88659	3.13545	5.73648
C	6.41112	0.31186	6.22987
C	3.79131	1.45972	8.01194
O	3.49644	-1.30565	6.90358
S	2.71336	-1.95870	8.16370
O	3.48579	-1.64570	9.35656
O	1.27467	-1.73948	8.10045
C	3.05819	-3.77539	7.84260
F	2.37620	-4.21128	6.78517
F	2.68073	-4.45268	8.92527
F	4.36115	-3.94267	7.63542
C	6.32740	-0.82379	5.19128
C	6.77094	-0.27986	7.60849
C	7.55209	1.26959	5.82018
H	2.70636	1.40839	7.85887
H	4.06208	2.41191	8.46940
H	4.07206	0.67467	8.71595
H	5.51001	3.29151	4.85080
H	5.39419	3.61103	6.58036
H	3.90777	3.60012	5.57345
H	4.29231	2.31046	3.76066
H	6.02490	-1.00113	7.96040
H	6.84199	0.54729	8.32016
H	7.73671	-0.80668	7.56006
H	7.40026	1.69699	4.82079
H	8.51173	0.73050	5.79670
H	7.59565	2.07328	6.56046
H	5.59770	-1.59197	5.46537
H	7.30459	-1.32254	5.10784

H	6.06739	-0.45909	4.19029
F	6.32393	2.58684	8.08900

TS1_1,3 Brook

C	0.57993	0.06332	-1.33403
C	0.12249	-0.55893	-2.50555
C	-0.23060	-1.91644	-2.45269
C	-0.10541	-2.63728	-1.26734
C	0.79483	-0.62914	-0.12066
C	0.41337	-1.94526	-0.16348
N	1.02338	1.39062	-1.16898
H	0.04991	-0.02536	-3.44729
H	-0.59676	-2.41075	-3.34866
H	-0.36900	-3.68795	-1.20648
Si	1.74359	1.45004	0.56541
C	2.05050	3.36507	0.60575
C	3.52584	0.66866	0.82988
C	0.58318	1.37758	2.09016
O	0.65350	-2.78737	1.02475
S	-0.42306	-2.83319	2.21265
O	0.27373	-2.68063	3.48562
O	-1.62431	-2.06093	1.91466
C	-0.89603	-4.63709	2.09890
F	-1.46711	-4.88444	0.91845
F	-1.76411	-4.91137	3.07455
F	0.18634	-5.40100	2.23821
C	4.07996	0.14082	-0.50965
C	3.48884	-0.48785	1.84801
C	4.51919	1.72282	1.36813
H	0.76611	2.20277	2.79133
H	0.67746	0.43198	2.63192
H	-0.46264	1.45498	1.76574
H	2.72636	3.74690	-0.16330
H	2.44837	3.66051	1.58429
H	1.09630	3.89801	0.48693
H	2.80000	-1.27930	1.54377
H	3.18054	-0.14321	2.84282
H	4.48904	-0.93346	1.95862
H	4.66126	2.55700	0.67400
H	5.50545	1.26257	1.52979
H	4.19982	2.14203	2.32940
H	3.45644	-0.65894	-0.92136
H	5.09238	-0.26833	-0.37322
H	4.14864	0.93178	-1.26564
H	0.60794	3.21012	-4.68168
H	-0.29670	1.68887	-4.59672
H	-1.16493	3.22015	-4.75565
C	-0.29794	2.73924	-4.28878
C	-0.36932	2.89593	-2.75529
H	-1.71072	1.17114	-2.46098
H	0.42720	4.93795	-2.80203
C	0.97697	2.41253	-2.12617
H	-1.34642	4.83807	-2.89013
C	-0.45128	4.40860	-2.42707
C	-1.63689	2.22779	-2.19990
O	1.99361	3.04152	-2.41023
H	-2.51827	2.73204	-2.61257
H	-0.51829	4.57872	-1.34676
H	-1.68895	2.31336	-1.10967

TS1_1,5 Brook

C	2.93006	1.47290	4.63046
C	1.62669	1.16138	4.17563
C	0.97045	0.01179	4.58821
C	1.60703	-0.85805	5.47613
C	3.57445	0.67277	5.62355
C	2.85203	-0.47736	5.96431
N	3.51700	2.56147	3.98461
C	4.80271	2.76223	4.09075
C	5.40718	3.77612	3.08432
C	4.68700	5.13261	3.23655
C	6.91612	3.97653	3.28474
C	5.16824	3.23301	1.65649
O	5.62073	2.15778	4.90648
H	1.17005	1.83241	3.45335
H	-0.01597	-0.23160	4.20404
H	1.14725	-1.79336	5.76854
H	4.84769	5.55895	4.23339
H	3.61004	5.02339	3.08551
H	5.06853	5.85037	2.50049
H	7.14517	4.40970	4.26131
H	7.29954	4.65958	2.51778
H	7.46214	3.03283	3.19788
H	4.10171	3.09500	1.46153
H	5.66609	2.26655	1.51330
H	5.57036	3.92972	0.91142
Si	5.23262	1.27301	6.61414
C	4.16265	1.32581	8.27378
C	6.57113	2.63797	7.34241
C	6.35895	-0.26532	6.49650
O	3.52646	-1.42648	6.83703
S	2.93889	-1.96480	8.23668
O	4.03661	-2.02442	9.19301
O	1.65464	-1.36587	8.58146
C	2.61630	-3.74799	7.76507
F	1.60853	-3.83223	6.89589
F	2.29559	-4.41235	8.87604
F	3.71319	-4.27692	7.22809
C	6.03129	4.07709	7.22785
C	7.93399	2.55958	6.62037
C	6.88208	2.38408	8.83747
H	5.81697	-1.17778	6.23998
H	6.89013	-0.44792	7.43846
H	7.11897	-0.10290	5.72387
H	3.97464	2.37026	8.55675
H	4.68277	0.86533	9.12035
H	3.17877	0.85805	8.19581
H	7.83794	2.66330	5.54032
H	8.44148	1.60799	6.82049
H	8.61033	3.35422	6.97823
H	6.04316	2.61770	9.49816
H	7.72260	3.02009	9.15924
H	7.17931	1.34640	9.03443
H	5.87418	4.37424	6.18716
H	6.73128	4.80485	7.67124
H	5.07494	4.20126	7.75272

TS2

C	-0.88918	0.78395	-1.63847
C	-2.03692	0.17401	-2.17850
C	-2.59353	-0.93079	-1.53478
C	-1.99925	-1.43723	-0.38259
C	-0.28900	0.39474	-0.40609
C	-0.89421	-0.73852	0.10599
N	-0.22584	1.81769	-2.36756
C	-0.55942	2.42458	-3.54731
C	0.46461	3.45367	-4.10045
C	0.22811	3.59509	-5.61444
C	0.19022	4.81127	-3.41324
C	1.92774	3.02978	-3.86351
O	-1.61968	2.21649	-4.14457
H	-2.46453	0.55207	-3.09643
H	-3.47123	-1.41761	-1.95081
H	-2.38945	-2.32836	0.09078
H	-0.80417	3.87965	-5.82820
H	0.43188	2.65488	-6.13834
H	0.89466	4.36202	-6.02289
H	-0.84239	5.13462	-3.58347
H	0.85687	5.57936	-3.82134
H	0.35744	4.75880	-2.33310
H	2.12468	2.02764	-4.25952
H	2.20869	3.04174	-2.80558
H	2.59785	3.72963	-4.37444
Si	1.36123	1.78829	1.24762
C	1.00863	3.33237	0.20002
C	2.85831	0.61326	0.77286
C	-0.01360	1.46650	2.51877
O	-0.17736	-1.34742	1.26275
S	-0.85426	-1.95440	2.57427
O	0.07651	-1.76689	3.68277
O	-2.26558	-1.60648	2.70905
C	-0.76068	-3.79376	2.22007
F	-1.63943	-4.15451	1.28276
F	-1.04090	-4.44250	3.35221
F	0.46898	-4.11136	1.81800
C	2.68606	-0.37047	-0.39339
C	3.22768	-0.19738	2.03666
C	4.05252	1.53434	0.41787
H	-0.95680	1.10707	2.10925
H	-0.20464	2.42599	3.01726
H	0.31998	0.77052	3.29386
H	1.72526	3.45801	-0.61905
H	1.11603	4.21026	0.84864
H	0.00033	3.34581	-0.21743
H	0.65528	2.08068	-1.95201
H	2.42632	-0.88705	2.32233
H	3.42756	0.45661	2.89078
H	4.13218	-0.79787	1.85896
H	3.85969	2.11718	-0.49144
H	4.95231	0.93137	0.22808
H	4.28254	2.23673	1.22202
H	1.91470	-1.11578	-0.19487
H	3.63398	-0.90409	-0.56530
H	2.42366	0.13507	-1.32856
F	2.32271	2.60576	2.41371

TS3

C	-0.81876	0.44508	-1.90851
C	-2.12983	0.04840	-2.25385
C	-2.73515	-1.04144	-1.60854
C	-2.07040	-1.77432	-0.60662
C	-0.12353	-0.27341	-0.90767
C	-0.82304	-1.23368	-0.42014
N	-0.14733	1.53407	-2.51555
C	-0.57448	2.39171	-3.49164
C	0.41872	3.49381	-3.93794
C	0.67369	3.28216	-5.44582
C	-0.28570	4.84947	-3.71818
C	1.76085	3.48864	-3.18710
O	-1.68903	2.31729	-4.01461
H	-2.66476	0.59152	-3.02106
H	-3.74531	-1.33074	-1.88740
H	-2.50422	-2.61583	-0.08431
H	-0.26778	3.27793	-6.00136
H	1.18497	2.33072	-5.63153
H	1.30492	4.08719	-5.83714
H	-1.24686	4.87566	-4.23797
H	0.33911	5.66395	-4.10006
H	-0.46745	5.03324	-2.65313
H	2.31361	2.55408	-3.33596
H	1.63237	3.65330	-2.11113
H	2.39169	4.29971	-3.56518
O	0.12154	-2.48738	0.95674
S	-0.00476	-2.29923	2.45034
O	1.23871	-2.61384	3.17723
O	-0.72656	-1.08112	2.85837
C	-1.15761	-3.68366	2.91723
F	-2.34312	-3.55112	2.29502
F	-1.38161	-3.68250	4.24102
F	-0.63943	-4.87413	2.57917
H	0.78318	1.67226	-2.14670

TS4

C	-0.93355	0.67448	-1.87992
C	-1.35704	-0.54709	-2.50964
C	-2.09609	-1.55821	-1.87671
C	-2.50982	-1.48251	-0.52658
C	-1.41117	0.62225	-0.54929
C	-2.07114	-0.27961	0.01695
N	-0.12226	1.59878	-2.47641
C	-0.65216	2.57777	-3.21929
C	0.36951	3.54532	-3.88859
C	0.17921	3.43373	-5.41557
C	0.02352	4.97977	-3.44120
C	1.83101	3.23714	-3.53291
O	-1.87187	2.79020	-3.42903
H	-1.06392	-0.68208	-3.54904
H	-2.36074	-2.44295	-2.45399
H	-3.07847	-2.26370	-0.03481
H	-0.86373	3.62353	-5.68454

H	0.44202	2.43051	-5.77381
H	0.81334	4.15422	-5.94684
H	-1.02515	5.20191	-3.65667
H	0.65114	5.71693	-3.95702
H	0.17937	5.10607	-2.36248
H	2.11705	2.22836	-3.84640
H	2.00544	3.30685	-2.45444
H	2.50333	3.94871	-4.02994

TS3_Ag

C	3.25951	0.19724	3.79162
C	2.47363	-0.54832	2.88253
C	2.04327	-1.84508	3.18422
C	2.36892	-2.47837	4.40491
C	3.59288	-0.41901	5.01472
C	3.12097	-1.61194	5.14399
C	4.39176	2.44268	4.04170
C	4.62749	3.77137	3.28771
C	4.01552	4.89511	4.15362
C	6.15608	3.96727	3.18311
C	4.01086	3.81833	1.87919
O	4.88133	2.31173	5.17631
H	2.19681	-0.10903	1.92772
H	1.44137	-2.38868	2.46104
H	2.05643	-3.47914	4.67098
H	4.44644	4.89467	5.15820
H	2.92966	4.77884	4.24383
H	4.21518	5.86731	3.69131
H	6.62207	3.95180	4.17156
H	6.37268	4.93185	2.71267
H	6.61503	3.18137	2.57283
H	2.91955	3.71774	1.90044
H	4.42656	3.04825	1.21926
H	4.23427	4.78800	1.42383
O	3.57571	-2.81786	6.89772
S	3.86654	-2.35218	8.29321
O	4.16180	-3.43334	9.24035
O	4.79406	-1.18179	8.36726
C	2.24659	-1.64495	8.86864
F	1.86983	-0.62403	8.08173
F	2.36351	-1.19395	10.12349
F	1.29332	-2.58315	8.83386
N	3.65120	1.49445	3.40265
H	3.31766	1.75691	2.48369
Ag	4.70780	0.51853	6.70867

TS4_Ag

C	-0.90573	0.55644	-2.10044
C	-2.26335	0.11653	-2.21972
C	-2.76791	-1.00625	-1.54577
C	-1.99432	-1.81514	-0.68484
C	-0.28899	-0.33934	-1.23197
C	-0.66195	-1.37869	-0.57844
N	-0.36445	1.63841	-2.72038
C	0.93555	1.81993	-2.44398
C	1.64395	3.03485	-3.08200
C	2.14127	3.94527	-1.93746
C	2.85529	2.51848	-3.88779
C	0.71457	3.83445	-4.00684
O	1.64355	1.07782	-1.69274
H	-2.91607	0.69539	-2.86870
H	-3.81361	-1.26517	-1.69895
H	-2.40796	-2.67782	-0.17438
H	2.80579	3.39802	-1.26275
H	1.30216	4.33346	-1.34813
H	2.69109	4.80189	-2.34373
H	3.53294	1.94351	-3.25064
H	3.41326	3.35871	-4.31648
H	2.53462	1.87228	-4.71348
H	-0.15297	4.22466	-3.46681
H	0.34333	3.22095	-4.83337
H	1.26070	4.68426	-4.43332
Ag	1.56348	-0.87972	-0.05618

HF

F	-4.32536	-0.64296	0.00000
H	-3.39445	-0.64296	0.00000

TBSF

Si	1.48839	1.91550	1.41057
C	0.90808	3.13821	0.11225
C	2.72936	0.64222	0.72875
C	0.03136	1.12390	2.28662
C	2.03760	-0.21429	-0.35369
C	3.21951	-0.27565	1.86837
C	3.93857	1.37110	0.10557
H	-0.55901	0.51160	1.59530
H	-0.63464	1.89335	2.69447
H	0.34876	0.48188	3.11513
H	1.74340	3.67386	-0.35122
H	0.23785	3.88242	0.55818
H	0.35174	2.63145	-0.68466
H	2.39567	-0.83353	2.32765
H	3.72335	0.28966	2.66094
H	3.93887	-1.01140	1.48271
H	3.64121	2.02065	-0.72546
H	4.66053	0.64301	-0.28962
H	4.46692	1.98925	0.84065
H	1.17747	-0.76494	0.04471
H	2.74086	-0.95592	-0.75716
H	1.68633	0.39250	-1.19637
F	2.29307	2.79508	2.55069

Triflate

O	0.11422	-2.47402	0.95575
S	0.00286	-2.30205	2.42317
O	1.23151	-2.61566	3.18981
O	-0.72091	-1.08599	2.86232
C	-1.15322	-3.67277	2.91441
F	-2.34372	-3.55387	2.29551

F	-1.38172	-3.66703	4.24197
F	-0.64588	-4.87870	2.59341

AgF

F	-4.10575	1.68039	0.00000
Ag	-2.04442	1.68039	0.00000

$[Ag(MeCN)_4]^+$

Ag	-0.06080	-0.02781	0.02714
C	1.33352	4.00823	2.62532
C	0.93003	2.83574	1.86749
N	0.60858	1.90098	1.26292
H	2.40432	3.95559	2.84161
H	0.77633	4.04742	3.56559
H	1.12551	4.91120	2.04430
C	4.02208	-1.50945	-2.44650
C	2.82140	-1.09026	-1.74298
N	1.86464	-0.75631	-1.18111
H	4.32505	-0.73110	-3.15246
H	3.82335	-2.43596	-2.99272
H	4.82858	-1.67902	-1.72754
H	-1.73108	-3.25087	4.08921
N	-0.85040	-1.71873	1.47874
C	-1.19034	-2.57099	2.18621
C	-1.61479	-3.64021	3.07383
H	-0.86349	-4.43529	3.07523
H	-2.57030	-4.04632	2.73022
H	-3.93795	0.30072	-3.78949
N	-1.77977	0.57244	-1.50867
C	-2.63926	0.83584	-2.23974
C	-3.71814	1.16482	-3.15604
H	-3.42184	2.00905	-3.78504
H	-4.61324	1.43368	-2.58787

MeCN

C	-2.73357	-4.17195	0.27705
C	-2.72287	-2.71600	0.27738
N	-2.71468	-1.55477	0.27761
H	-3.24684	-4.54064	1.16965
H	-1.70804	-4.55166	0.27273
H	-3.25414	-4.54015	-0.61153
